# IAP antagonist tolinapant (ASTX660) in combination with pembrolizumab in advanced solid tumors: a phase I trial

**DOI:** 10.1038/s41467-026-77552-7

**Published:** 2026-09-30

**Authors:** Crescens Tiu, Wing Yau, Tencho Tenev, Daniel Lionarons, Ching Leung, Victoria Sanchez-Perez, Giovanni Codacci-Pisanelli, Xiaoran Lai, Anna Zachariou, Aasia Hussain, Toby Prout, Mona Parmar, Bindu Baikady, Maria Goicoechea, Scott Layzell, Naomi Guppy, Ioannis Roxanis, Thomas Lund, Diego Carlos Dos Reis, Camila Pereira Almeida, Tatiany Luiza Silveira, Ana Ferreira, Bora Gurel, Claudia Bertan, Mateus Crespo, Penny Flohr, Ann-Marie Baker, Trevor Graham, Luis Zapata, Claudio Salas, Soham Mandal, Alan Dunlop, Martin Sims, George Ward, Tomoko Smyth, Simone Jueliger, Matthew Davis, Ilya Potapov, Aram Oganesian, Danna Chan, Mattias Leino, Subham Basu, Andre Samson, James M. Murphy, Nina Tunariu, Edward W. Johnston, Alistair Ring, Nick Turner, Andrew N. J. Tutt, Amanda Swain, Anna Minchom, Adam Sharp, Alec Paschalis, Udai Banerji, Johann de Bono, Bristi Basu, Christina Yap, Syed Haider, Juanita Lopez, Pascal Meier

**Affiliations:** 1 The Breast Cancer Now Toby Robins Research Centre, The Institute of Cancer Research, London, UK; 2 Drug Development Unit, The Institute of Cancer Research and The Royal Marsden NHS Foundation Trust, Sutton, UK; 3 Cambridge University Hospitals NHS Foundation Trust, Cambridge, UK; 4 Clinical Trials and Statistics Unit, The Institute of Cancer Research, Sutton, UK; 5 Integrated Pathology Unit, The Institute of Cancer Research and The Royal Marsden NHS Foundation Trust, Sutton, UK; 6 Cancer Biomarkers Group, The Institute of Cancer Research, Sutton, UK; 7 Centre for Evolution and Cancer, The Institute of Cancer Research, Sutton, UK; 8 Immunophenotyping Laboratory, The Royal Marsden NHS Foundation Trust, Sutton, UK; 9 Astex Pharmaceuticals, Cambridge, UK; 10 Taiho Oncology Inc., Pleasanton, CA USA; 11 Navinci Diagnostics, Uppsala, Sweden; 12 The Walter and Eliza Hall Institute of Medical Research, Parkville, VIC Australia; 13 Department of Medical Biology, University of Melbourne, Parkville, VIC Australia; 14 Drug Discovery Biology, Monash Institute of Pharmaceutical Sciences, Monash University, Parkville, VIC Australia; 15 The Royal Marsden NHS Foundation Trust, Sutton, UK; 16 Department of Radiotherapy and Imaging, The Institute of Cancer Research, London, UK; 17 Development and Cancer Group and Tumor Modeling Facility, The Institute of Cancer Research, London, UK; 18 University of Cambridge, Cambridge, UK

**Keywords:** Cancer therapy, Phase I trials

## Abstract

Successful cancer immunotherapy requires not only tumor cell killing but also the activation of innate and adaptive immune responses. The inhibitor of apoptosis (IAP) proteins cIAP1, cIAP2 and XIAP act as key suppressors of RIPK1-driven immunogenic cell death, thereby enabling tumor immune evasion and limiting the effectiveness of immune checkpoint blockade (ICB). ASTX660 (tolinapant) is a non-peptidomimetic antagonist with a balanced activity against all clinically relevant IAPs, designed to lower the TNF cytotoxicity threshold and sensitize tumors to T cell-mediated killing. Here, we present results from the phase I ASTEROID trial (NCT05082259), evaluating ASTX660 in combination with pembrolizumab in patients with advanced solid tumors. Primary endpoints were recommended phase 2 dose (RP2D) and safety, while secondary endpoints were anti-tumor activity and pharmacokinetics. The combination was well tolerated and demonstrated early evidence of efficacy. RP2D was ASTX660 150 mg (7-days-on/14-days-off) with pembrolizumab. Overall response rate was 33%, including responses in ER⁺ breast cancers typically resistant to ICB. Integrated translational analyses reveal that response is associated with a TNF/IFN-enriched inflammatory tumor microenvironment, increased tumor antigenicity and enhanced TCR clonal diversity, whereas resistance correlates with a CCR4/6/7/8-driven TAM/Treg-dominant niche. Together, these results provide clinical proof-of-concept that dual IAP and PD-1 blockade sensitize tumors, while identifying candidate biomarkers to guide patient stratification in future studies.

## Abstract (web summary)

The IAP antagonist ASTX660 has the potential to enhance cancer immunotherapy by sensitizing tumour cells to immune-mediated killing. Here, the authors report results from an open-label, multicentre phase I trial evaluating the safety, tolerability, pharmacokinetics, and pharmacodynamics of ASTX660 in combination with the anti-PD-1 antibody pembrolizumab.

## Introduction

Effective cancer therapy may, in some contexts, require not only direct tumor cell killing but also mobilization and activation of the immune system to achieve durable, systemic anti-tumor responses^1–3^. Consequently, stimulating anti-tumor immunity has emerged as a key principle of modern cancer therapy, sparking intense interest into improving current immunotherapy development.

The generation of anticancer immunity follows a multi-step process often described as the cancer-immunity cycle^4^. The death of antigen-bearing tumor cells provides the initiating event, enabling their capture by antigen-presenting cells (APCs), most prominently dendritic cells (DCs)^5^. These antigens are displayed via major histocompatibility complex (MHC) molecules, encoded in humans by the HLA locus, enabling T cell recognition. However, whether this process leads to immune priming or tolerance depends on contextual signals, including the nature of the tumor antigens, the release of adjuvants from dying cells such as damage-associated molecular patterns (DAMPs), and the cytokine and chemokine milieu^5^. These signals determine DC maturation and trafficking to lymph nodes, where they initiate adaptive immune responses^6^.

Although rewiring cell death pathways to favor immunogenic outcomes has emerged as a promising strategy to enhance cancer therapies, translating this strategy into clinical practice remains challenging^1,3,7,8^. Most physiological cell turnover occurs through intrinsic apoptosis, a process that is largely immunologically silent and maintains tissue homeostasis^7,9,10^. In contrast, inflammatory apoptosis, necroptosis and pyroptosis - death programs often triggered during infection - can generate danger signals that activate innate and adaptive immunity^2,3,7,11^. Redirecting tumor cell death toward these immunogenic pathways offers an attractive strategy to improve the efficacy of cancer therapies, but clinical translation requires overcoming key biological barriers.

Central to overcoming such barriers is the inhibitor of apoptosis (IAP) protein family, a group of Ubiquitin E3 ligases with critical immunoregulatory functions^2,7,11–15^. Among these, cIAP1, cIAP2 and XIAP act as essential gatekeepers of both cell death and inflammatory signaling. Alterations in IAP proteins are common across cancers, driving tumor survival, therapy resistance and poor prognosis^16,17^. Amplification of the 11q22.1 locus encoding cIAP1/2 occurs in cervical^18,19^, breast^20^, squamous^21,22^ and brain^23,24^ cancers, while overexpression of IAPs in other tumor types similarly correlates with adverse outcomes^25,26^. Beyond promoting tumor-intrinsic survival, cIAP1/2 also shields cancer cells from T cell–mediated bystander killing, a process initiated by CD8⁺ T cell release of interferon (IFN) and tumor necrosis factor (TNF) within the tumor microenvironment^27,28^. By elevating the cytotoxicity threshold of TNF, cIAPs reinforce immune resistance and undermine the effectiveness of immunotherapy.

At the core of these pathways lies receptor-interacting serine/threonine-protein kinase 1 (RIPK1), a stress sentinel downstream of death receptors (DR), cytokine receptors and pattern-recognition receptors (PRR)^11,12,29–33^. RIPK1 integrates inflammatory gene expression with inflammatory/immunogenic forms of cell death, including DR- and PRR-induced apoptosis, necroptosis and pyroptosis, through tightly regulated post-translational modifications. In healthy cells, RIPK1 kinase activity is actively restrained by ubiquitylation and Ubiquitin-dependent phosphorylation, preventing inappropriate immune activation^11,13,14,34–37^. Conversely, RIPK1 activation and ripoptosome assembly can drive inflammatory/immunogenic cell death that promotes cross-priming of CD8⁺ T cell responses^5,38–42^.

The clinically relevant IAPs, cIAP1, cIAP2 and XIAP, function as the main Ubiquitin E3 ligases for RIPK1^2,7,11–15^. By ubiquitylating RIPK1, they suppress its cytotoxic potential while reinforcing its scaffolding role in NF-κB activation and NF-κB–dependent induction of pro-survival genes^43,44^. This signaling axis can also fuel the induction of an immunosuppressive chemokine program that limits natural killer and CD8⁺ T cell infiltration, reduces local TNF superfamily ligands, and drives resistance to immune checkpoint blockade (ICB) in preclinical models^45–47^.

Small-molecule IAP antagonists, such as ASTX660^48,49^, were first developed to induce tumor apoptosis; however, growing evidence shows that their effects extend well beyond direct cytotoxicity, encompassing restoration of exhausted immune responses, remodeling of the tumor microenvironment, and unleashing RIPK1-driven inflammatory/immunogenic cell death^16,34,35,50,51^. ASTX660 is a non-peptidomimetic IAP antagonist with a balanced profile against cIAP1, cIAP2 and XIAP, with IC50s of 0.22 nM and 2.8 nM for cIAP1 and XIAP, respectively, distinguishing it from earlier SMAC mimetics^48,49^. As monotherapy, it has demonstrated anti-tumor activity in patients with T cell lymphoma, although limited efficacy was observed in patients with unselected advanced solid tumors (NCT02503423)^52–54^. By lowering the TNF cytotoxicity threshold and sensitizing tumor cells to CD8⁺ T cell–mediated bystander killing, ASTX660 may trigger RIPK1-dependent inflammatory cell death and promote adaptive immune responses that could be further potentiated by ICB.

Here we report the investigator-initiated, open-label, multi-center ASTEROID Phase I trial (NCT05082259), evaluating safety, recommended Phase 2 dose, pharmacokinetics, pharmacodynamics and preliminary immunologic activity of ASTX660 in combination with the anti-PD-1 antibody pembrolizumab in patients with advanced solid tumors. We show that ASTX660 is well tolerated, with evidence of immune engagement and early signs of efficacy. Integrated translational analyses suggest that benefit arises in tumors with a TNF/IFN-driven inflammatory microenvironment, increased tumor antigenicity and enhanced TCR clonal diversity, whereas resistance is associated with a CCR4/6/7/8-driven TAM/Treg-dominant niche. These features highlight candidate biomarkers for patient stratification and provide a foundation for rational development of IAP antagonists in combination with ICB.

## Results

### Dose escalation and baseline patient characteristics

**Fig. 1:**
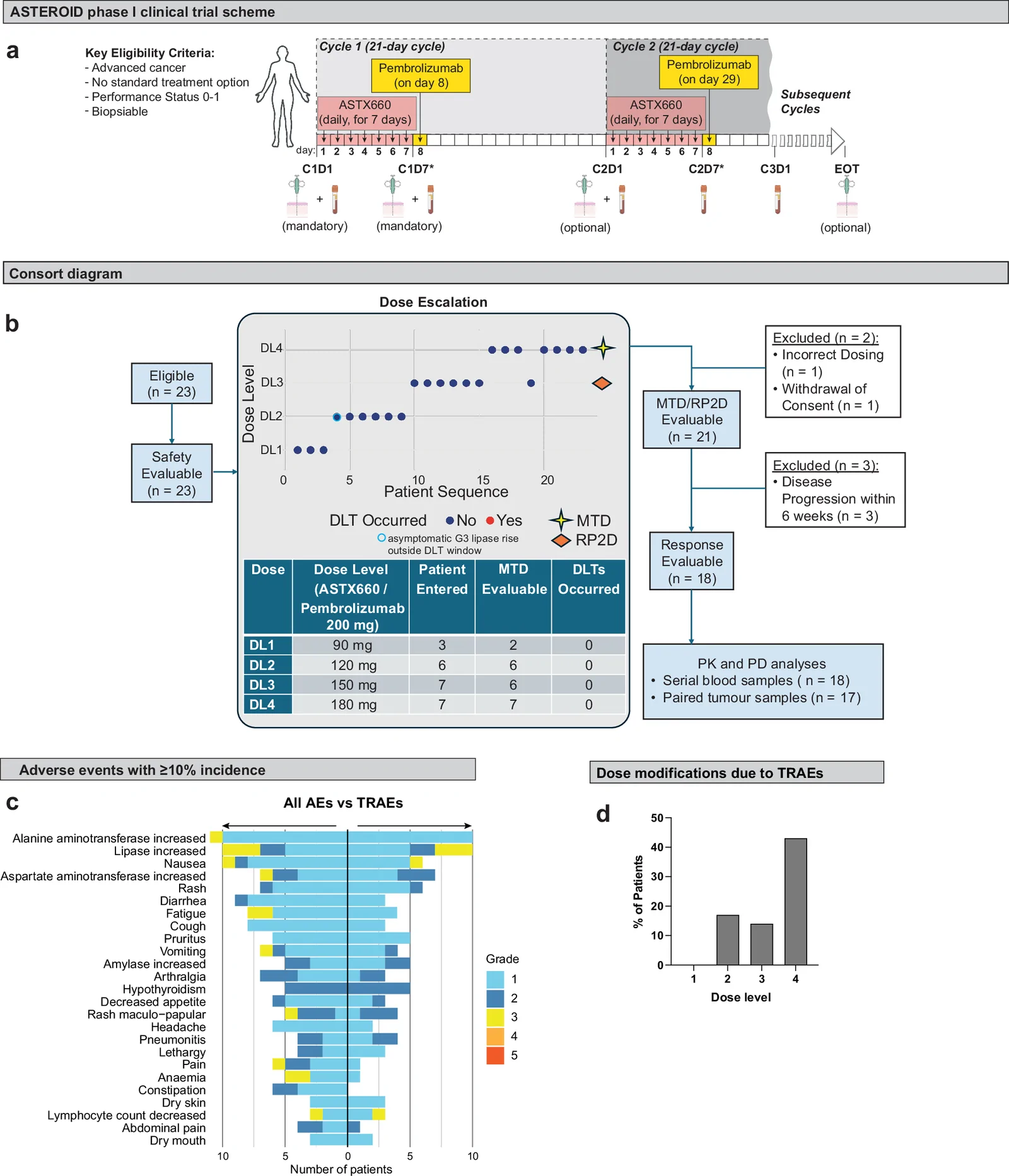
ASTEROID trial design and safety data. **a** Patients with advanced cancers who had exhausted standard treatment options were enrolled. Eligible patients received ASTX660 orally once daily for 7 days, followed by intravenous pembrolizumab on day 8, in 21-day cycles. Dose-limiting toxicities (DLTs) were assessed during the initial 6-week period. Core needle biopsies and blood samples were taken at the specified time points for pharmacokinetic (PK) and pharmacodynamic (PD) analyses: C1D1 and C2D1 (pre-ASTX660) and C1D7 and C2D7 (post-ASTX660/pre-pembrolizumab). *For plasma protein analysis, post-ASTX660 samples were taken at C1D8 and C2D8, pre-pembrolizumab. C2D1 and End-of-Trial (EOT) biopsies were optional. **b** Consort diagram showing treatment allocation of eligible patients (*n* = 23), including their evaluability for safety, tolerability, response and PK/PD analyses. Patients were treated at 4 dose levels (DL) of ASTX660 with a fixed dose of pembrolizumab (200 mg). No dose-limiting toxicity (DLTs) occurred. One patient at dose level 2 (DL2) had asymptomatic Grade 3 lipase rise outside the DLT window. Patients must have received at least two cycles ( ≥6 weeks) of combination therapy to be evaluable for response by imRECIST, to ensure adequate drug exposure, as per the pre-specified trial protocol. **c** Volcano plot depicting the number of patients who experienced adverse events (AEs) of any cause versus treatment-related adverse events (TRAEs). The most frequently observed AEs ( ≥10% incidence) are indicated. **d** Bar plot showing the incidence of dose modifications (interruptions, delays or reductions) due to TRAEs across different dose levels. Source data is provided as a Source data file for Fig. 1c, d.

Twenty-three patients were enrolled in Part A of the Phase I ASTEROID trial between March 2022 to November 2023. The data presented here are based on a cut-off date of October 24, 2025. Part A was a dose-escalation, proof-of-concept study in which ASTX660 was administered orally once daily for seven days, followed by intravenous pembrolizumab (200 mg) on day eight, in 21-day cycles until unacceptable toxicity or disease progression (Fig. 1a; “Methods”). Median follow-up was 17 months [95% confidence interval (CI): 6.0 – not estimable (NE)].

Dose escalation, planned for up to 18 response-evaluable patients, was guided by a 2-stage Bayesian time-to-event continual reassessment method (TITE-CRM)^55^. Treatment began at 90 mg ASTX660 daily, the minimum anticipated biological effect level, and increased to 180 mg daily, in line with the recommended Phase 2 dose (RP2D) determined in a prior monotherapy study (NCT02503423)^52^, given on a 7-days on, 14-days off schedule. Of 23 eligible patients, 21 were evaluable for determination of the maximal tolerated dose (MTD) (Fig. 1b). Eighteen patients completed at least two cycles (six weeks) of treatment and were evaluable for clinical response. Paired tumor biopsies taken on Cycle 1 Day 1 (C1D1, pre-treatment) and Cycle 1 Day 7 (C1D7, post-ASTX660 treatment) were available from 17 response-evaluable and 5 non-evaluable patients for pharmacodynamic analyses (Fig. 1b and Supplementary Data 1). Median age was 55 years [interquartile range (IQR) 45–73], and female:male ratio was 18:5. The median number of prior lines of systemic treatment for metastatic disease was 5 (IQR 2-6). Four (17.4%) had prior immune checkpoint inhibitor treatment (Table 1). Patients were enrolled with the following tumor types: breast [ER^+^HER2^-^ (*n* = 12), triple-negative breast cancer (TNBC, 1), ER^-^HER2^+^ (1)], cervical (3), cholangiocarcinoma (1), head and neck squamous cell carcinoma (HNSCC, 2), hepatocellular carcinoma (HCC, 1), melanoma (1), and pancreatic (1). Full patient characteristics and dosing are shown in Table 2. Prediction Analysis of Microarray 50 (PAM50)^56^ gene expression classification confirmed that nine ER^+^ breast cancer cases were luminal A or B subtypes (Supplementary Table 1).

**Table 1:** Patient demographics and baseline characteristics

Characteristic	Overall *N* = 23^1^
**Age (years)**	55.0 (37.0, 80.0)
**Sex**	
Female	18 (78.3%)
Male	5 (21.7%)
**Ethnicity**	
Any Other White Background	3 (13.0%)
Black/Black British: Caribbean	2 (8.7%)
White: British	15 (65.2%)
Not disclosed	2 (8.7%)
Missing	1 (4.3%)
**ECOG performance status at screening**	
0	5 (21.7%)
1	18 (78.3%)
2-4	0 (0.0%)
**Primary cancer diagnosis**	
Breast Cancer	14 (60.9%)
Cervical Cancer	3 (13.0%)
Cholangiocarcinoma	1 (4.3%)
Head & Neck Cancer	2 (8.7%)
Hepatocellular	1 (4.3%)
Melanoma	1 (4.3%)
Pancreatic Cancer	1 (4.3%)
**Metastatic disease present at diagnosis**	7 (30.4%)
**Number of prior systemic anti-cancer treatments**	5.0 (1.0, 12.0)
**Prior checkpoint inhibitor treatment**	4 (17.4%)

^1^Median (Min, Max); n (%).

**Table 2:** Patient disease characteristics and ASTX660 dose level

ID no	Sex	Tumor type	PAM50	Germline BRCA	PD-L1 (H-score)	TMB (mut/MB)	Other markers	Prior Treatment Lines for Metastatic Disease	Best response	Dose level (mg)
101-001	F	Breast ER-HER2 +	NA	wt	NA	NA		5	NE^a^	DL1 (90)
101-002	F	Breast ER +	LumA/B	wt	0	8.1		7	SD	
101-003	F	Breast ER +	LumB	wt	0	8.4		12	SD	
101-004	F	Breast ER +	LumB	wt	0	24.1*	ATM mutant	3	PR	DL2 (120)
101-010	F	Breast ER +	NA	wt	225	NA	MMR proficient, high TILs	5	PR	
101-008	F	Breast ER +	LumA	NA	0	3.0	Somatic BRCA2, ESR1, TP53 mutant; no MSI-H	5	PD	
101-007	F	Cervix SCC		NA	0-5	4.0		1	PD	
102-001	F	Cholangiocarcinoma		NA	0	2.1		2	PD	
101-009	F	Breast ER +	NA	wt	0	NA	PIK3CA mutant	5	NE^c^	
101-011	F	Cervix adenocarcinoma		NA	0	5.3		2	PR	DL3 (150)
102-003	M	HNSCC		NA	5–35	39.2*	PMS2 mutant; high blood TMB(34 muts/MB); no MSI-H	3 (ICI-refractory)	PR	
101-015	F	Breast ER +	LumB	wt	0	NA		11	PD	
101-016	F	Breast ER +	LumB	NA	1–5	7.2	AKT1, BAP1, ESR1, FGF10/4, GATA3, IRF2, MYC, PIK3CA mutant; no MSI-H	5	PD	
101-020	F	Breast ER +	LumB	gBRCA2	0	8.8		4	PD	
101-013	M	Breast ER +	LumB	NA	0	NA	Somatic BRCA2 mutant	4	NE^c^	
101-012	F	Cervix		NA	55–75	NA		4 (ICI-refractory)	NE^b^	
101-021	F	Breast TN	NA	wt	0	NA	PIK3CA, TP53, KMT2D, AKT1,MAP2K4 mutant	5	PR	DL4 (180)
101-017	F	Breast ER +	LumA	gBRCA2	0	NA	IDH1 mutant; no MSI-H	7	PD	
102-005	M	HCC		NA	0	3.2		1	SD	
102-006	M	Melanoma		NA	2	NA		1 (ICI-refractory)	PD	
102-007	M	HNSCC		NA	0	NA		6 (ICI-refractory)	NE^c^	
101-018	F	Breast ER +	NA	NA	0	2.9		8	SD	
102-008	F	Pancreas adenocarcinoma		NA	3-4	NA	Unknown MSI/MMR status	2	PR	

*DL *dose level, *ER* estrogen receptor, *F* female, *HNSCC* head and neck squamous cell carcinoma, *HER*2 human epidermal growth factor receptor 2, *HCC* hepatocellular carcinoma, *ICI* immune checkpoint inhibitor, M male, muts/MB mutations per megabase, *MSI-H* microsatellite instability-high, *NA* not assessed, *SCC* squamous cell carcinoma, *TILs* tumor infiltrating lymphocytes, *TN* triple-negative, *TMB* tumor mutational burden, *high ≥ 10 mut/MB, *wt* wildtype, *NE* Not evaluable, *PR* partial response, *PD* progressive disease, *SD* stable disease.

^a^in correct dosing.

^b^withdrew consent .

^c^rapid PD < 6weeks.

### ASTX660-pembrolizumab combination is safe and well tolerated

We first assessed the safety and tolerability profile of ASTX660 in combination with pembrolizumab. Overall, this treatment was well tolerated, with no Grade 4 or 5 treatment-related adverse events (TRAEs) observed (Fig. 1c). The most frequent TRAEs were Grade 1-2 rash (43%), alanine aminotransferase (ALT) increase (43%), lipase increase (30%), aspartate aminotransferase (AST) increase (30%), and nausea (26%). Grade 3 events included lipase elevation (13%) and single cases (4% each) of nausea, eosinophilia and lymphopenia, which were all transient. No dose-limiting toxicities (DLTs) were identified within the 6-week DLT-observation period. The MTD per the Bayesian TITE-CRM model was therefore determined as ASTX660 180 mg (dose level 4, DL4) orally for seven consecutive days, combined with pembrolizumab 200 mg given on day 8 of a 21-day cycle (Fig. 1b). DLT rate at this dose level was 0.030 (90% credible interval: 0-0.222) (Supplementary Fig. 1).

Dose modifications (dose interruption, delay or reduction) due to TRAEs were required in 5 of 23 patients (22%), with increased frequency at the higher dose levels [43%; 3 of 7 patients at DL4] (Fig. 1d). Elevation in lipase, with or without concurrent amylase increase, occurred in 10 patients (43%). All cases were asymptomatic with no radiological or clinical evidence of pancreatitis. Only one patient required corticosteroid treatment (oral prednisolone 10 mg daily for 7 days), while the remaining cases resolved with dose adjustments/interruptions or no intervention. One patient at dose level 2 (DL2) developed asymptomatic Grade 3 lipase elevation at cycle 3 day 8 (C3D8) outside the DLT period, prompting a 6-week treatment interruption; lipase levels normalized without any supportive medication or sequelae. Subsequently, the protocol was amended to allow the use of low-dose oral prednisolone as supportive medication for patients who developed asymptomatic lipase elevations. Although the role of steroids in managing asymptomatic lipase elevations remains unclear and no specific treatment guidelines exist^57^, protocol amendment was guided by patient safety considerations in the context of this experimental drug combination. The approach aimed to facilitate early detection and mitigation of potentially severe immune-related adverse events, minimize treatment interruptions, and limit steroid exposure to the lowest effective dose whenever possible.

Treatment-related Grade 1-2 rash was observed in ten patients (43%), all emerging within the first two treatment cycles, most commonly following the first dose of pembrolizumab (C1D8), consistent with immune-related skin reactions. All resolved with oral antihistamines and a short pulse of topical or oral steroids. Pneumonitis (Grade 1–2) occurred in four patients (17%), with two cases at DL3 (emerging during Cycle 11 and 12, respectively) and two at DL4 (one presenting during Cycle 3, the other in Cycle 14) (Supplementary Fig. 2). All improved spontaneously or with the use of systemic steroids and dose modifications. There was one event each of immune arthritis and oral mucositis requiring pulsed oral steroids, and six immune-related endocrine events (1 hyperthyroidism, 5 hypothyroidism) requiring supportive treatment (Supplementary Table 2).

### Rapid absorption and dose-dependent exposure of ASTX660

**Fig. 2:**
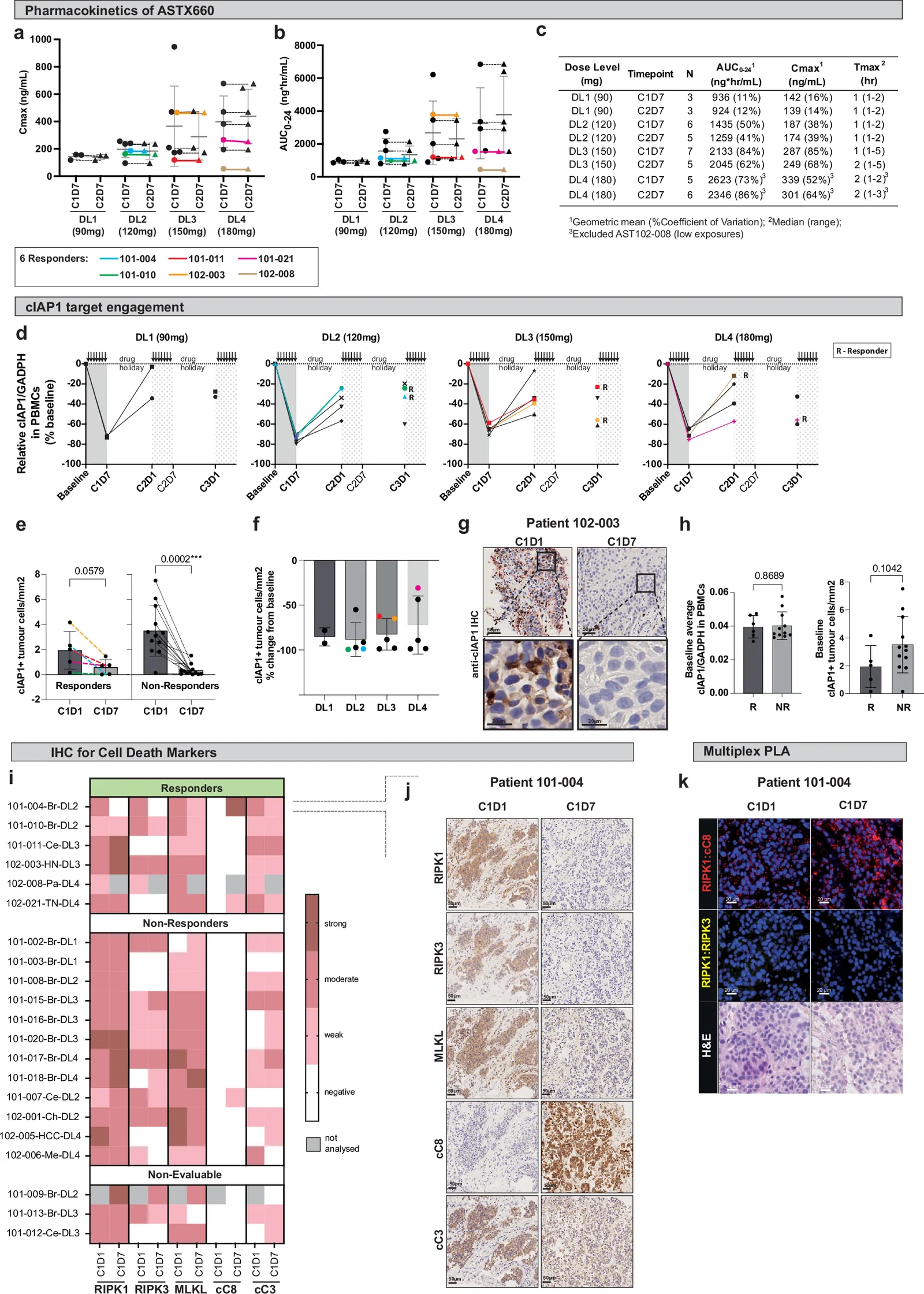
PK and PD cIAP1 on-target drug activity of ASTX660. **a** Maximum plasma concentrations (C_max_) of ASTX660 at C1D7 (dot) and C2D7 (triangle). **b** Area under the plasma concentration-time curve for 0–24 h (AUC0-24) at C1D7 and C2D7. For Fig. 2a, b, each data point represents a plasma sample taken from an individual patient at timepoints C1D7 (dot) and C2D7 (triangle). Six responders are color-coded. ASTX660 DL1: C1D7 and C2D7 (*n* = 3 each); DL2: C1D7 (*n* = 6), C2D7 (*n* = 5); DL3: C1D7 (*n* = 7), C2D7 (*n* = 5); DL4: C1D7 (*n *= 6), C2D7 (*n* = 7). Error bars indicate mean ± SD. **c** Table showing average PK parameters of oral ASTX660. Patient 102-008 who had the lowest C_max_ and AUC, was excluded from statistical analysis. **d** cIAP1 levels in PBMCs following ASTX660 treatment. At C1D7, cIAP1 levels decreased from baseline across all four dose levels (mean change: − 72.4% (DL1), − 62.1% (DL2), − 65.1% (DL3), − 69.5% (DL4)). After the 14-day drug holiday, cIAP1 levels remained reduced by an average of 32.1% (range − 3.0 to − 57%) at C2D1, and 40.0% (− 19.7 to 60.2%) at C3D1. Each data point represents a PBMC sample taken from an individual patient at timepoints: baseline, C1D7, C2D1 and C3D1. ASTX660 DL1: each timepoint (*n* = 2); DL2 and DL3: baseline, C1D7 and C2D1 (*n* = 5 each), C3D1 (*n* = 4); DL4: baseline and C1D7 (*n* = 5 each), C2D1 (*n* = 4), C3D1 (*n* = 3). **e**,** f** Densities of cIAP1^+^ tumor cells decrease with ASTX660 treatment from C1D1 to C1D7. Responders (*n* = 5), non-responders (*n* = 12). DL1 (*n* = 2), DL2 (*n* = 5), DL3 (*n* = 5), DL4 (*n *= 5). Error bars indicate mean ± SD; *p*-values were determined using a two-tailed paired *t* test. **g** Representative cIAP1 immunohistochemistry (IHC) staining in tumor biopsies from patient 102-003 taken pre- (C1D1) and post- (C1D7) ASTX660 treatment. **h** Baseline (C1D1) cIAP1 levels in PBMCs (left) and tumor tissue (right) comparing responders (R) and non-responders (NR). In PBMCs, R (*n* = 6), NR (*n* = 11); in tumor, R (*n* = 5), NR (*n* = 12). Error bars indicate mean ± SD; *p*-values were determined using a two-tailed unpaired *t* test. **i** Heat map of IHC staining for five cell death components [RIPK1, RIPK3, MLKL, cleaved caspase 8 (cC8) and cleaved caspase 3 (cC3)] in paired tumor biopsies. Expression intensities were graded by pixH-score (strong: 200.1–300; moderate: 100.1–200; weak: 50.1–100; negative 0–50). **j** Representative images of IHC of cell death components in paired tumor biopsies from patient 101-004. **k** Multiplex in situ proximity ligation assay (isPLA) showing protein:protein proximity in paired tumor samples from representative patient 101-004. Shown are RIPK1:cC8 and RIPK1:RIPK3 ‘interactions’, with corresponding H&E images. Source data for Fig. 2a–f, 2h–j are provided as a Source Data file.

To characterize the pharmacokinetic (PK) profile of oral ASTX660, blood samples were collected at defined time points after seven days of dosing in the first two cycles (C1D7 and C2D7). PK data were available for 22 patients (Fig. 2a–c). Across all dose levels, ASTX660 was rapidly absorbed, with a median time to maximal concentration (T_max_) in plasma of 1–2 h. Drug exposure, as measured by maximum plasma concentration (C_max_) and the area under the plasma concentration-time curve for 0–24 h (AUC_0-24_), increased in a dose-dependent manner (Fig. 2c). Average values were consistent with PK data from a previous ASTX660 monotherapy trial^52^.

### ASTX660 results in robust cIAP1 target engagement and downstream activation of cell death machinery

To evaluate target engagement, we assessed cIAP1 protein levels in peripheral blood mononuclear cells (PBMCs) and tumor biopsies from patients treated with ASTX660. cIAP1 was efficiently degraded in PBMCs by day 7 of cycle 1 (C1D7). At the higher dose levels (DL2-4), cIAP1 levels did not fully recover and remained suppressed (− 3 to − 57%) during the 14-day interval off ASTX660 (Fig. 2d). This sustained depletion likely reflects the ongoing pharmacodynamic effect of cIAP1 degradation rather than prolonged drug exposure, as the mean half-life of ASTX660 at the highest dose level (DL4, 180 mg) is 18 h^52^. Depletion of cIAP1 compared to baseline was additionally confirmed in tumor cells using immunohistochemistry (IHC) across all patients (Fig. 2e–g). Although we could not confirm target engagement of cIAP2 and XIAP due to suboptimal validation of assays in human blood and tissue samples, prior preclinical work has shown depletion of cIAP2 and antagonism of XIAP at doses that effectively deplete cIAP1^58^. These findings confirm that oral ASTX660 achieves systemic bioavailability in solid tumor patients at all dose levels explored, with sustained pharmacodynamic activity of IAP blockade at DL2 and higher.

Neither baseline cIAP1 expression nor the extent of its depletion in PBMCs or tumor samples correlated with clinical response (Fig. 2h). Downstream effects of target engagement were explored by examining five key mediators of immunogenic cell death by IHC: RIPK1, RIPK3, MLKL and cleaved caspase-8 (cC8). We also monitored cleaved caspase-3 (cC3), an executioner of apoptosis. All 20 available cycle 1 day 1 (C1D1) pre-treatment samples stained positive for RIPK1. RIPK3 was detected in 13 of 20 (65%) and MLKL in 19 of 20 (95%) cases, indicating that most patients carried the molecular machinery for ripoptosome-mediated inflammatory/immunogenic cell death (Fig. 2i and Supplementary Fig. 3).

In a responding patient with ER-positive breast cancer (patient 101-004) treated at DL2, ASTX660 induced changes in cell death proteins (Fig. 2j). ASTX660 triggered prominent activation of caspase-8 in tumor cells (cC8), consistent with activation of the ripoptosome/complex-II, while levels of RIPK1, RIPK3, and, to a lesser extent, MLKL, were reduced. Multiplex in situ proximity ligation assay^59^ (isPLA) confirmed a pronounced increase in RIPK1:cC8 PLA signals, suggesting ripoptosome assembly. Decrease in RIPK3 and MLKL levels, as well as absence of RIPK1:RIPK3 interactions, suggested that cell death proceeded predominantly via RIPK1/caspase-8–mediated inflammatory apoptosis (Fig. 2k and Supplementary Fig. 4). Treatment with ASTX660 did not significantly alter Ki67 expression, and baseline Ki67 levels were not associated with treatment response (Supplementary Fig. 5).

### Durable responses with ASTX660-pembrolizumab combination

**Fig. 3:**
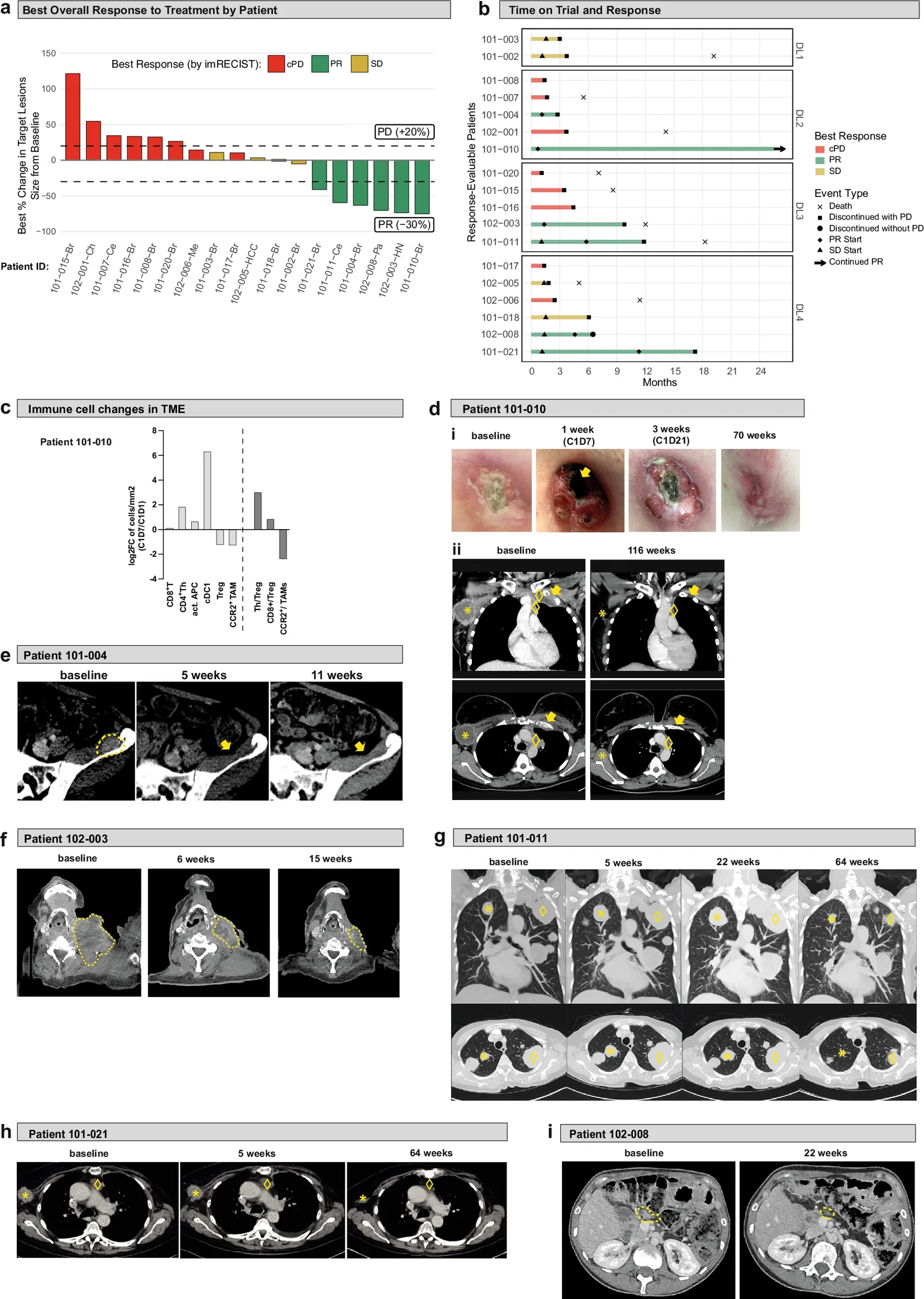
Anti-tumor activity of ASTX660 with pembrolizumab. **a** Waterfall plot showing the best percentage change in the sum of target lesion diameters from baseline for each patient. Bars are color-coded according to best overall response by immune-modified Response Evaluation Criteria in Solid Tumors (imRECIST): confirmed progressive disease (cPD), partial response (PR), or stable disease (SD). Cancer types are indicated for each patient: breast (Br), cholangiocarcinoma (Ch), cervical (Ce), melanoma (Me), hepatocellular carcinoma (HCC), pancreatic (Pa), head and neck (HN). **b** Swimmer plot of response-evaluable patients showing time on trial, color-coded by best response per imRECIST. Patients 101-010 had ongoing partial response at data cut-off. Patient 102-008 withdrew from the trial and was lost to follow-up. Figures 3a and 3b exclude three patients who had rapid PD in <6 weeks and were therefore not evaluable for response as per the pre-specified trial protocol. The objective response rate (ORR) in the protocol-specified evaluable population was 6/18 (33.3%), and 6/21 (28.6%) for the intention-to-treat (ITT) population. **c** Changes in immune cell populations within the tumor microenvironment (TME) based on multiplex immunofluorescence (mIF) analysis of paired tumor biopsies collected pre- (C1D1) and post- (C1D7) ASTX660. T cells (T), helper T cells (Th), activated antigen-presenting cells (APC), classical dendritic cells type 1 (cDC1), Treg (regulatory T cells), tumor-associated macrophages (TAM). See Supplementary Fig. 3b for mIF images. **d** Exceptional response in patient 101-010: i, Serial photographs of the sternal metastasis showing early tumor necrosis after one week of ASTX660 (arrow) and complete wound healing by 70 weeks (baseline and week 70 images courtesy of the patient). ii: Serial coronal and axial contrast-enhanced CT images demonstrating near-complete resolution of a large, predominantly necrotic right axillary node (asterisks), marked regression of left mediastinal pleural disease with minimal residual abnormality (diamonds), and healing of lytic lesions in the medial aspect of the first and second left ribs (arrows). **e** Response in patient 101-004. Axial contrast-enhanced CT images of the pelvis showing maintained complete resolution of a soft tissue nodule (dotted outline and arrows) associated with a left iliac bone metastasis at 5 and 11 weeks (confirmed on MRI, not shown). **f** Response in patient 102-003. Axial contrast-enhanced CT images showing a significant reduction in the extent of the large ulcerative left supraclavicular nodal mass (dotted outline) after 6 and 15 weeks on treatment. **g** Response in patient 101-011. Axial and coronal contrast-enhanced CT images (lung windows) showing an initial increase ( +20 to +30%) in the bilateral upper lobe metastases (asterisks and diamonds) followed by a substantial reduction (−30 to −50%) at 64 weeks, with resolution of several smaller lesions (not shown). **h** Response in patient 101-021. Axial contrast-enhanced CT images demonstrating near-complete resolution of right axillary (asterisks) and anterior mediastinal lymphadenopathy (diamonds) after 64 weeks of treatment. **i** Response in Patient 102-008. Axial contrast-enhanced CT images showing decreased size and enhancement of a locally recurrent pancreatic lesion (dotted outline) after 22 weeks of treatment. Source data for Fig. 3a–c are provided as a Source Data file.

Clinical activity was observed across dose levels 2-4 in patients with advanced, treatment-refractory cancers that are typically unresponsive to ICB. Confirmed responses were observed in three patients with breast cancer (two ER^+^/HER2^-^, one PD-L1 negative triple-negative), one patient with cervical adenocarcinoma, one patient with oral squamous cell carcinoma refractory to nivolumab, and one patient with pancreatic cancer, highlighting the broad potential for clinical benefit with ASTX660-pembrolizumab (Fig. 3). Across 18 response-evaluable patients, six partial responses by Immune-Modified Response Evaluation Criteria in Solid Tumors (imRECIST) yielded a preliminary overall response rate (ORR) of 33% (6/18) (Fig. 3a). Responders demonstrated marked tumor shrinkage exceeding the − 30% threshold, with some responses lasting beyond one year (Fig. 3b). As indicated above, paired biopsies from an ER^+^ breast cancer responder (patient 101-004, DL2) provided evidence of ripoptosome- and caspase-8-dependent tumor cell death, while RNA-seq principal component analysis revealed transcriptomic changes between C1D1 and C1D7, in contrast to minimal changes in patients with non-responding ER^+^ breast cancers (Supplementary Fig. 6). Immune pharmacodynamic profiling in an ER^+^ breast cancer responder (patient 101-010, DL2) revealed ASTX660-induced remodeling of the tumor microenvironment (TME), with enrichment of CD4^+^ helper T cells (Th), activated antigen-presenting cells [APCs, particularly classical dendritic cell type 1 (cDC1)], and reductions in regulatory T cells (Treg) and CCR2^+^ tumor-associated macrophages (TAM), shifting the TME toward an immune-activated state (Fig. 3c and Supplementary Fig. 7a). Notably, this tumor also exhibited strong PD-L1 expression and the highest baseline TIL content among all patients (Supplementary Figs. 7b, 8), features that may have contributed to the observed response.

Detailed clinical profiles of patients who obtained benefit from ASTX660/pembrolizumab are presented below.

**Patient 101-010 (DL2)** presented with advanced ER^+^/HER2^-^, BRCA1/2 wild-type breast cancer, with metastases to lymph nodes and chest wall, and a tumor microenvironment characterized by high PD-L1 expression and abundant TIL content ( >50%). She had received extensive prior treatment, including five previous regimens: ribociclib/letrozole, exemestane/everolimus, capecitabine, paclitaxel, and tamoxifen. Notably, the chest wall lesion developed marked inflammation and necrosis following seven days of ASTX660 alone, suggesting that ASTX660 primed tumor immunogenicity for subsequent ICI response. The lesion subsequently granulated and closed by week three, after initiating pembrolizumab. As of data cut-off, she has remained on study for >24 months with ongoing deepening of response (−75%) and complete wound healing (Fig. 3d-i, ii). TRAEs were mild (G1 fever, G1 rash, G1 nausea, G1 lipase rise and G2 hypothyroidism) and resolved.

**Patient 101-004 (DL2)** had advanced ER^+^/HER2^-^, BRCA1/2 wild-type breast cancer with metastases to the liver, left iliac wing soft tissue lesion, and T7 epidural region. She previously had received letrozole, paclitaxel with maintenance fulvestrant, and capecitabine for metastatic disease. Following two cycles (5 weeks), scans demonstrated a partial response (PR) of − 63%. Treatment was interrupted at cycle 3 day 8 due to asymptomatic G3 lipase elevation, which improved without requiring steroids. During this period of interruption, the patient developed back pain. As a result, staging scans were brought forward (week 11) to investigate the cause of pain and assess overall disease status prior to recommencing Cycle 4. Although imaging continued to show target lesions meeting imRECIST criteria for PR, non-measurable epidural disease was identified at the site corresponding to the patient’s pain. At that time, it was unclear whether the symptoms were due to true disease progression, tumor flare or pseudoprogression. The Safety Review Committee (SRC) determined that the patient met protocol-specified response criteria with respect to improvement in target lesions. However, considering the clinical scenario, including the need for radiotherapy to the symptomatic site and the prolonged treatment interruption, the decision was made to discontinue the patient from the trial (Fig. 3e). Further tumor profiling revealed high tumor mutational burden (TMB, 24 mut/Mb) (Table 2).

**Patient 102-003 (DL3)** had oral squamous cell carcinoma that was refractory to the ICB, nivolumab (anti-PD1), despite molecular profiling suggesting mismatch repair deficiency (PMS2 mutation) and high TMB (39 mut/Mb) (Table 2). Disease sites included left supraclavicular mass, left axillary and subpectoral nodes, and anterior right rib (6th) metastasis. Prior lines of treatment included cisplatin/5-fluorouracil/cetuximab; nivolumab (anti-PD-1); surgery and radiotherapy to the neck. He developed G1 rash and G1 pneumonitis, which resolved without steroid intervention. The patient achieved a −74% reduction in supraclavicular nodes with a progression-free survival (PFS) of 9.7 months (Fig. 3f).

**Patient 101-011 (DL3)** had cervical mesonephric adenocarcinoma and lung metastases, previously treated with carboplatin/paclitaxel/bevacizumab and cisplatin. Initial scans showed apparent progression, followed by a delayed PR (− 59%), consistent with pseudoprogression (Fig. 3g). Immune-related adverse events (irAE) included G1-2 flare of pre-existing arthritis, G2 hypothyroidism, G1 rash, and G2 pneumonitis. She was unable to restart trial treatment due to complications from the steroids used for treatment of pneumonitis, but remained on surveillance. Her PFS was 11.7 months, and at the time of study discontinuation, imaging showed ongoing maintained response to therapy in lung and nodal disease, but the emergence of a new bony rib lesion.

**Patient 101-021 (DL4)** had TNBC, BRCA wild-type, PD-L1 negative, with metastases to lymph nodes, pleura, and spine. Previous treatment for early breast cancer included neoadjuvant epirubicin/cyclophosphamide/paclitaxel and adjuvant capecitabine. Treatment for metastatic disease were carboplatin/gemcitabine, paclitaxel, carboplatin (rechallenge), eribulin, docetaxel, and palliative radiotherapy to T12. At week 14, CT scans showed a PR (−31%) of target lesions, however new leptomeningeal disease at T4 and cauda equina were noted on MRI. Given the absence of neurological symptoms and a lumbar puncture, which showed only inflammatory cells in cerebrospinal fluid (CSF) without malignant disease, this was considered pseudoprogression and treatment continued alongside close surveillance. At week 32, MRI scans showed further enlargement of meningeal lesions with ongoing extracranial response. Given the overall clinical benefit of the trial treatment, she received palliative radiotherapy (20 Gray) to the spine with tapering steroids to reduce the risk of neurological compromise. At Cycle 14, scans showed signs of pneumonitis, which was asymptomatic and resolved without requiring steroids. Scans at 11.3 months (49 weeks) confirmed a partial response of −41%, which was ongoing at 14.7 months (64 weeks, Fig. 3h). PFS was 17 months.

**Patient 102-008 (DL4)** had peritoneal metastases from pancreatic adenocarcinoma, previously treated with adjuvant modified FOLFIRINOX (folinic acid/5-FU/irinotecan/oxaliplatin) and gemcitabine/nab-paclitaxel for metastatic disease. She achieved a radiological PR of −70% and a sharp decline in CA19-9 from baseline of 2600 down to 35 U/mL (Fig. 3i**;** Supplementary Fig. 9). G2 pneumonitis, prompted sequential dose reductions at Cycle 4 (to DL3) and Cycle 5 (to DL2). The patient withdrew consent after five cycles. PFS was 6.3 months. Paired tumor samples were not available for this patient due to safety concerns at the time of the scheduled biopsy.

The expansion cohorts were established by the SRC through an integrated assessment of safety, pharmacokinetic, pharmacodynamic and preliminary efficacy data of these cases. Doses at or above DL2 were considered to be *biologically active*, based on: (i) effective cIAP1 depletion in blood and tumor with sustained suppression across the 7-days-on/14-days-off treatment cycle; (ii) downstream pharmacodynamic evidence of ripoptosome assembly and caspase-8 activation in tumor cells; and (iii) consistent clinical activity across DL2-DL4, with two partial responders at each dose level, accompanied by immune activation in the TME in responding patients (see below). While the Bayesian model identified DL4 as the MTD, frequent dose modifications at this level limited dosing intensity (Fig. 1d and Supplementary Fig. 2). Balancing biological activity, clinical efficacy, and tolerability, DL3 was selected as the RP2D to be further evaluated in the expansion cohorts.

### Immune signatures associated with response and resistance

**Fig. 4:**
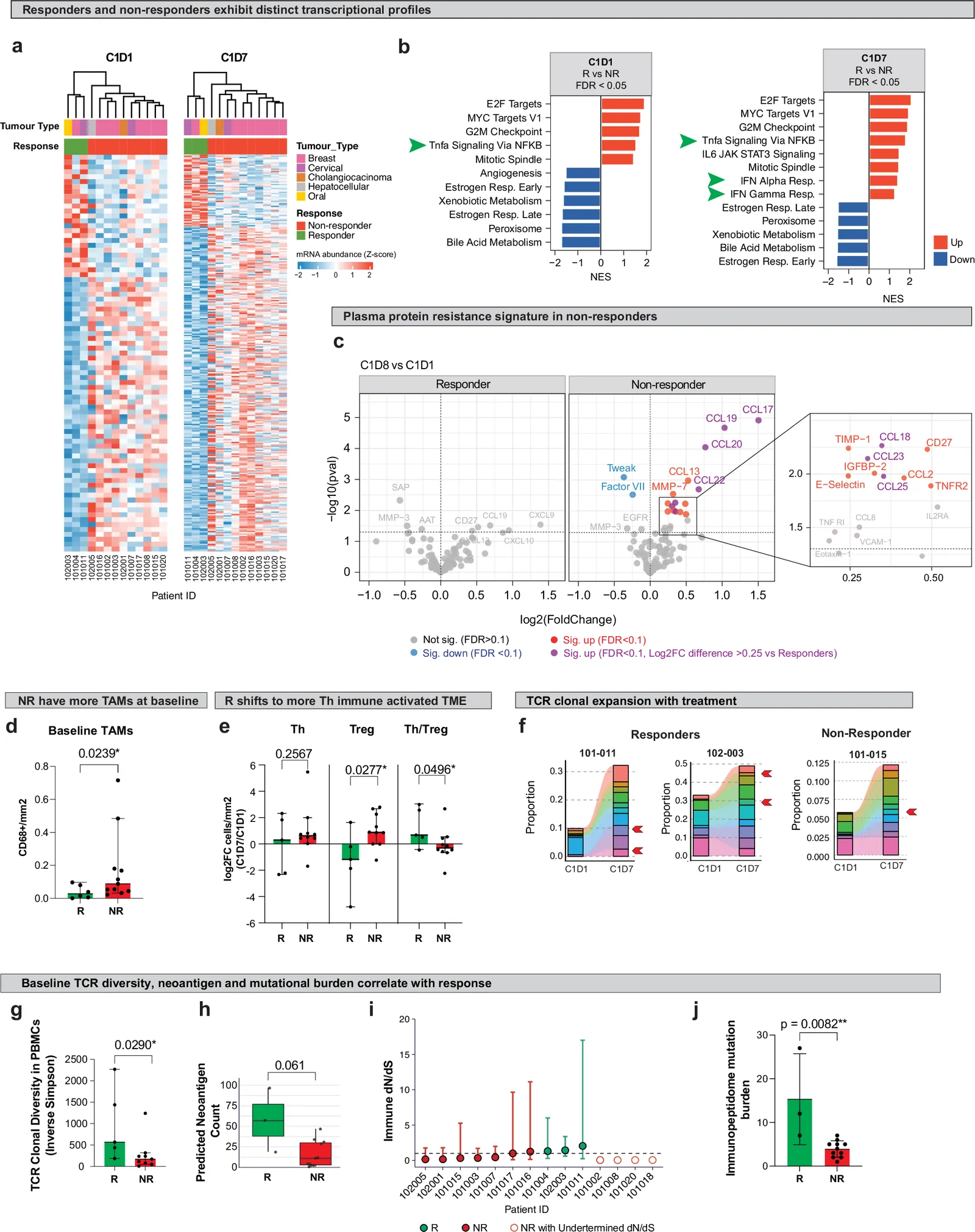
Immune and inflammatory pathway changes with ASTX660. **a** Heat maps showing differential gene expression between responders (R) and non-responders (NR) at baseline (C1D1) and on-treatment (C1D7) following ASTX660, determined by bulk RNA sequencing of tumor biopsies. False discovery rate (FDR) < 0.05. Scale bar indicates mRNA abundance standardized per gene (Z-score). Details are provided in Supplementary Data 2a, b. **b** Gene set enrichment analysis (GSEA) of RNA-seq data using MSigDB Hallmark collection showing pathways differentially enriched in responders (R) versus non-responders (NR) at baseline (C1D1) and following ASTX660 treatment (C1D7). NES, normalized enrichment score. See Supplementary Fig. 6a, b and Supplementary Data 2c, d for full gene set data. **c** Volcano plots of log2 fold change (log_2_FC) of plasma protein concentrations comparing post- (C1D8) versus pre- (C1D1) ASTX660 treatment plotted against -log10 (*p*-value). Proteins with FDR < 0.1 were considered significant; those with log_2_FC difference of > 0.25 between groups (NR minus R) were considered upregulated (purple). Statistical calculations were performed using a linear mixed-effects model (see “Methods” for details). **d** Baseline density of tumor-associated macrophages (TAM) (cells/mm2). Comparisons between R (*n* = 6) and NR (*n* = 11) groups were calculated using a one-tailed Mann-Whitney test, with *p* < 0.05 considered significant; error bars represent median ± 95% CI. **e** Log_2_FC of helper T cells (Th), regulatory T cells (Treg) and Th/Treg ratio from C1D1 to C1D7. Comparisons between R (*n* = 5) and NR (*n* = 10) groups were calculated using a one-tailed Mann-Whitney test; error bars represent median ± 95% CI. **f** Tracking of the ten most frequent T-cell clonotypes detected in the C1D7 tumor sample to their presence in the respective C1D1 tumor sample. Red arrows indicate emergence of new clonotypes absent at baseline. Additional TCR clonotype tracking across tumor and PBMC samples are shown in Supplementary Fig. 14a. **g** Baseline (C1D1 PBMC sample) TCR repertoire diversity, measured by inverse Simpson index. Two germline *BRCA2* mutation carriers and one patient with a *PMS2* mutation (Lynch syndrome) carrier were excluded. R (*n* = 5), NR (*n* = 9); *p*-value calculated using two-tailed Mann-Whitney test; error bars represent median ± 95% CI. **h** Neoantigen burden stratified by response status. Box plots show the median (horizontal line), interquartile range (IQR, box), and 1.5 x IQR (whiskers); R (*n* = 3), NR (*n* = 11); *p*-value was determined by two-tailed Mann-Whitney test. **i** Immune dN/dS (ratio of non-synonymous to synonymous mutations). Each point is the immune dN/dS estimate for an individual tumor; error bars show the 95% CI. The horizontal dashed line marks the neutral evolution threshold (dN/dS = 1). Four non-responders had undetermined dN/dS values due to the absence of synonymous mutations (dS = 0). R (*n* = 7), NR (*n* = 3). **j** Immunopeptidome mutation burden stratified by response status. R (*n* = 3), NR (*n* = 11). Error bars show median ± 95% CI; *p*-value was determined by two-tailed Mann-Whitney test. Source data for Fig. 4c–e, g–j are provided as a Source Data file.

Analysis of 50 hallmark gene sets^60^ revealed that, at baseline, responders were enriched for proliferative and inflammatory pathways, most notably TNF signaling via NFκB [normalized enrichment score (NES) = 1.546, false discovery rate (FDR) adjusted p-value (adj. p) = 0.010] These data support the hypothesis that IAP inhibition is most effective in a TNF-rich TME, providing a potential predictor of response to the combination treatment. By C1D7, enrichment in responders broadened to include IFNα and IFNγ responses as well as IL6 >JAK >STAT3 signaling (Fig. 4a, b; Supplementary Fig. 10a and Supplementary Data 2c, d). Although IFN signaling increased in both responder and non-responder groups after ASTX660 treatment, responders tended to already have higher baseline signaling, which further increased upon treatment (Supplementary Fig. 10b–d). Notably, the convergence of TNF and interferon signaling renders cancer cells particularly susceptible to CD8⁺ T cell-mediated bystander killing when IAPs are antagonized^27,28,61^, providing a mechanistic rationale for the observed transcriptional profile in responders.

To extend these observations, we profiled patient plasma using a 130-analyte biomarker panel (Human ExplorerMap v.1.0). No baseline differences were detected (Supplementary Fig. 11). However, ASTX660 treatment induced a consistent increase in 15 plasma proteins in non-responders (FDR adj. *p* <0.1) (Fig. 4c and Supplementary Data 3). Among these, seven chemokines were specifically elevated in non-responders when compared to responders [log_2_ fold change (log_2_FC) difference >0.25]: CCL17 and CCL22, CCR4 ligands that attract FOXP3⁺ Tregs and Th2 cells^62,63^; CCL18, known to be secreted by M2-like TAMs to promote Treg and cancer-associated fibroblast induction through receptors PITPNM3/CCR8/CCR6^64–66^; CCL23, a CCR1 ligand that recruits monocytes/myeloid-derived suppressor cells^66,67^; CCL20, a CCR6 ligand that draws Tregs and Th17 cells^68–71^; CCL19, a CCR7 ligand directing lymphocyte trafficking into lymphoid rather than tumor sites^72,73^; and CCL25, a CCR9 ligand implicated in apoptosis resistance, migration and immunosuppression^72,74^. Collectively, this chemokine program suggests a microenvironment enriched in TAMs and Tregs, with reduced effector T cell recruitment, providing a putative mechanistic explanation for immune evasion and ICB resistance. Interestingly, in an independent ASTX660 monotherapy trial in peripheral T cell lymphoma (ASTX660-01, NCT02503423^52^), CCL18 (ligand for PITPNM3/CCR6/8), CCL19 (ligand for CCR7), CCL20 (ligand for CCR6), CCL17 and CCL22 (ligands for CCR4) were likewise selectively elevated in non-responders (log_2_FC difference >0.25, Supplementary Fig. 12 and Supplementary Data 3), reinforcing that CCR4/6/7/8-driven recruitment of immunosuppressive TAMs and Tregs represents a plausible mechanism of resistance to IAP antagonism.

Consistent with this notion, we found that non-responders exhibited significantly higher baseline CD68^+^ TAM density compared to responders (*p* = 0.0239; Fig. 4d), indicative of a more immunosuppressive TME. Following ASTX660, total TAM levels tended to increase in non-responders, although this did not reach statistical significance, while responders showed a trend towards reduced proportions of CCR2⁺ TAMs – a subset associated with inflammatory and pro-tumorigenic activity (Supplementary Fig. 13a).

Further, Treg density tended to rise in non-responders but declined in responders, who also demonstrated corresponding increases in both Th/Treg (Fig. 4e) and CD8^+^/Treg ratios (Supplementary Fig. 13b). These findings suggest that ASTX660 can remodel the TME from an immunosuppressive to a more immune-activated state, thereby enhancing susceptibility to ICB. This effect was most evident in the exceptional responder (Patient 101-010) described above. Conversely, high baseline TAM infiltration and failure to reduce CCR2^+^ TAMs and Tregs may contribute to resistance to therapy.

We next examined T cell receptor (TCR) repertoire dynamics in response to ASTX660. FUME-TCR sequencing of paired tumor and PBMC samples revealed expansion of pre-existing clonotypes as well as the emergence of new ones in both responders and non-responders (Fig. 4f and Supplementary Fig. 14a). However, responders displayed significantly greater baseline TCR diversity and reduced clonal dominance in PBMCs compared with non-responders (Fig. 4g). Intratumoral TCR diversity was comparable in responders and non-responders (Supplementary Fig. 14b). Moreover, whole exome sequencing showed that responders (*n* = 3) carried the maximum total number of unique HLA class I alleles (*n* = 6) (Supplementary Fig. 15) with some evidence of higher neoantigen burden in responders compared to non-responders (median neoantigen count 57.7 vs 16.2, *p *= 0.061) (Fig. 4h). Notably, the two patients with highest neoantigen burden also had high TMB (≥ 10 mut/Mb) and both responded to treatment (patients 01-004 and 102-003; Table 2). Together, these data suggest that patients with broader systemic immune surveillance capacity and increased tumor antigenicity favor therapeutic benefit from ASTX660 combined with ICB.

To assess tumor immunogenicity, we calculated the ratio of non-synonymous to synonymous mutations (dN/dS) within the immunopeptidome^75^. Responders had immune dN/dS >1, consistent with escape from immune selection and increased antigenicity, a feature previously linked to ICB sensitivity^76^. (Fig. 4i). This is supported by significantly higher immunopeptidome mutation burden in responders vs non-responders (median mutation count, 12 vs 4, *p* = 0.0082) (Fig. 4j and Supplementary Fig. 16). Finally, flow cytometry of patient PBMCs showed no significant treatment-induced changes in dendritic cell or T cell populations between groups. (Supplementary Fig. 17).

Together, our data suggest that patients with broader TCR repertoire diversity, maximal HLA class I heterozygosity, and higher predicted neoantigen and immunopeptidome mutation burden are more likely to benefit from ASTX660 in combination with ICB. While increased tumor antigenicity (immune dN/dS >1) was enriched in responders, its presence in a non-responder indicates that antigenicity alone is insufficient, and additional factors such as TAM/Treg-driven suppression likely determine outcome. Thus, effective response appears to require both tumor immunogenicity and a permissive immune microenvironment.

## Discussion

Here, we assessed the safety and preliminary clinical activity of ASTX660 in combination with pembrolizumab. The regimen was generally well tolerated and demonstrated encouraging anti-tumor activity, with partial responses observed in six patients whose tumors are typically unresponsive to pembrolizumab alone. Notably, two of ten patients with ER^+^ breast cancer achieved a partial response at DL2. We observed no DLTs across all dose levels. TRAEs at ≥DL2, such as elevated pancreatic enzymes, rash and pneumonitis, were mitigated through proactive measures, including enhanced monitoring and the use of steroids. The MTD was established at 180 mg (DL4), although this dose level was associated with more frequent dose modifications.

Notably, pneumonitis occurred in four patients treated at DL3 and DL4, all of whom experienced clinical benefit. While our study was not powered to mechanistically dissect this relationship, several plausible explanations can be considered. First, pneumonitis may represent an on-target manifestation of heightened systemic immune activation in patients mounting the most robust anti-tumor immune responses. In this context, ASTX660 may enhance tumor immunogenicity and inflammatory signaling, while pembrolizumab may further amplify activated T-cell responses, together increasing both anti-tumor activity and the risk of immune-mediated tissue inflammation. Second, although patients were screened and monitored for concomitant inflammatory or infectious conditions, it is possible that patients who developed pneumonitis had a pre-existing inflammatory condition, predisposing both to response and to immune-related toxicity. This would be consistent with the broader concept that efficacy and immune-related adverse events can, in some cases, arise from overlapping biology. Notably, three of these cases occurred in patients with predisposing risk factors, such as extensive lung metastases (patient 101-011), prior radiotherapy to the chest (patient 102-003), and radiotherapy to the T12 metastasis (patient 101-021). However, areas of pneumonitis do not radiologically overlap with sites of metastases or prior treatment fields. Third, given the mechanism of IAP antagonism, a contributory role for enhanced non-canonical NF-κB-driven inflammatory signaling may also be plausible. Such effects could lower the threshold for inflammatory damage in normal tissues such as the lung. Larger studies with longitudinal immune profiling, including analyses of circulating cytokines, T-cell activation states, and tissue-specific immune signatures, will be needed to clarify whether pneumonitis and response are biologically linked in this setting.

Patient 101-011 developed irAEs aside from pneumonitis, including persistent grade 1-2 arthritic flare and grade 1 rash, consistent with reports linking irAE to improved response to ICB^77^. A comparable rate of lipase elevation (35%) was observed in a Phase 2 ASTX660 monotherapy trial using the same dose (180 mg) but on a more intensive schedule (7 days on/7 days off). However, that study reported lower rates of rash (24%) and pneumonitis (3%)^78,79^. By contrast, pneumonitis rates for pembrolizumab monotherapy are typically at 1–5%, with slightly higher frequencies (6%) in patients with prior thoracic irradiation^80^. Taken together, these data indicate that the combination of ASTX660 and pembrolizumab enhances anti-tumor activity but also increases the incidence of immune-mediated toxicities, particularly rash and pneumonitis, relative to monotherapy with either agent. Importantly, despite this elevated risk, adverse events remained manageable and reversible under appropriate supportive care. Considering biological activity, clinical benefit and tolerability, DL3 (150 mg) was selected as the RP2D for evaluation in the expansion cohorts.

The potential impact of the use of corticosteroids for TRAEs on immune checkpoint inhibitor (ICI) efficacy is currently under investigation. High peak doses of prednisone ( >1.0 mg/kg) are associated with worse overall survival, whereas low-dose steroids (10 mg/day) do not appear to compromise anti-tumor efficacy^81,82^. In our cohort, only one patient with an asymptomatic lipase increase received corticosteroids (oral prednisolone 10 mg daily for 7 days). Given the low dose and short duration of corticosteroid use, a negative impact on ICI efficacy is unlikely. Although asymptomatic lipase elevations can resolve spontaneously, this must be weighed against the risk of disease progression from prolonged treatment interruptions. In selected cases, a short course of low-dose corticosteroids may be a reasonable strategy to facilitate treatment continuation. All TRAEs treated with corticosteroids received prednisone-equivalent doses of <1 mg/kg, except for patient 101-011, who had 2 mg/kg methylprednisolone (2.5 mg/kg prednisone equivalent) for pneumonitis (Supplementary Table 2). Notably, this patient achieved a durable response of 11.7 months, suggesting that corticosteroid use, when clinically indicated, does not necessarily preclude meaningful anti-tumor activity. Importantly, no patients received concomitant corticosteroids during the pharmacodynamic sampling window (C1D1 to C1D7), except for one patient on long-term physiologic dose of hydrocortisone for adrenal insufficiency, indicating that these analyses are unlikely to be confounded by corticosteroid exposure.

By using multiplex proximity ligation assay, we were able to interrogate how ASTX660 shapes cell death pathways in patient tumor samples. In an ER^+^ breast cancer patient (101-004), ASTX660 promoted the interaction of RIPK1 with caspase-8 and caspase-8 activation, consistent with assembly of the ripoptosome^40^. This occurred in the absence of RIPK1:RIPK3 interactions, pointing towards caspase-8-driven inflammatory apoptosis rather than necroptosis. Consistently, IHC indicated increased levels of cleaved, active caspase-8 alongside loss of RIPK1, RIPK3 and MLKL in this patient. This observation supports the notion that necroptosis may only occur when caspase-8 activity is blocked. While ASTX660 triggered ripoptosome assembly in this case, similar profiles were not apparent in other responders at day seven of cycle one, pointing to temporal, spatial (sampling) or patient-specific heterogeneity in how IAP antagonism rewires cell death programs. Particularly, in some cases, the pre- and post-treatment samples were necrotic, leading to their exclusion from the analysis and complicating direct comparisons between timepoints.

We observed evidence of ‘pseudoprogression’ in several patients, a phenomenon in which immune infiltration can transiently increase lesion size. This was most apparent in lung metastases of cervical cancer patient 101-011, and may also have occurred in TNBC patient 101-021, who presented with enlarging meningeal lesions despite non-malignant CSF. Patient 101-004, who showed an early treatment response, may likewise have experienced symptoms attributable to pseudoprogression rather than true disease progression. These cases highlight the importance of carefully weighing the risks and benefits when deciding whether to continue therapy in patients with suspected pseudoprogression. Incorporating advanced imaging modalities or biomarker-based assessments may help distinguish true progression from immune-related inflammatory changes, thereby guiding more informed treatment decisions.

Among all patients, patient 101-010 was the only case with a “hot” TME, defined by abundant TILs and PD-L1-positive disease, which is atypical for ER^+^ disease. She achieved an exceptional response lasting more than two years and elected to move to surveillance upon completion of 2 years of therapy. Owing to the single-arm design of the study, we cannot formally distinguish whether the clinical benefit in this patient was driven by ASTX660, pembrolizumab, or their combination. Trial biopsies contained insufficient tumor content to repeat hormone receptor IHC staining or perform sequencing analyses, and therefore, microsatellite instability, TMB, and PAM50 status could not be assessed to evaluate for an immune-sensitive or more basal-like phenotype. Nevertheless, several observations support the view that ASTX660 made an important biological contribution and may have enhanced responsiveness to PD-1 blockade. Most notably, during the 7-day ASTX660 lead-in period, before pembrolizumab was introduced, there was clear clinical evidence of tumor inflammation and necrosis. In parallel, tumor analyses after ASTX660 monotherapy showed remodeling of the tumor microenvironment towards a more inflamed, immune-active state, consistent with the hypothesis that ASTX660 increased tumor immunogenicity and thereby primed the lesion for subsequent checkpoint inhibition. At the same time, the baseline presence of high TILs and PD-L1 expression suggests that this tumor may already have possessed features associated with sensitivity to pembrolizumab. Taken together, we believe the most appropriate interpretation is that the observed response likely reflects a favorable immune contexture that was further augmented by ASTX660, rather than being attributable with confidence to either agent alone.

Although PD-L1 is not a consistent predictor of immunotherapy benefit, in this setting, pembrolizumab treatment may have supported a durable response by enabling immune cell recruitment and fostering a TNF- and IFN-enriched milieu that favors bystander killing. Such a gene expression signature was consistently seen in tumors of responders after seven days of ASTX660 but absent in non-responders, suggesting that IAP inhibition is most effective in TNF/IFN-rich TMEs and, therefore, may predict response to ICB. In contrast, non-responders displayed a chemokine program enriched for CCL17, CCL18, CCL19, CCL20, CCL22, CCL23, and CCL25, collectively shaping a suppressive TME dominated by Tregs, and TAMs with limited effector T cell recruitment. Notably, upregulation of CCL17, CCL18, CCL19, CCL20 and CCL22 was also detected in an independent trial, reinforcing CCR4/6/7/8-mediated Treg recruitment as a likely recurrent resistance mechanism.

Together, these findings from samples taken after ASTX660 (but before pembrolizumab) suggest that responders are defined by a TNF/IFN-driven inflammatory TME, whereas non-responders are characterized by a CCR4/6/7/8-mediated, TAM/Treg-dominant suppressive niche. According to this scenario, IAP inhibition acts upstream to lower the threshold for inflammatory tumor cell death and immune activation, thereby generating a TME in which pembrolizumab is more likely to be effective. Importantly, enhanced blood TCR clonal diversity and increased tumor antigenicity at baseline further mark responders, underscoring that durable benefit from ASTX660 combined with ICB arises when inflammatory signaling, antigenicity, and T cell diversity converge to overcome tumor immune evasion. These features may therefore serve as candidate biomarkers to guide patient stratification and optimize the clinical application of IAP antagonists in combination with immunotherapy, although their predictive value will require further validation in larger cohorts.

In contrast to other IAP antagonists, ASTX660 is a non-peptidomimetic IAP antagonist with a relatively balanced cIAP/XIAP inhibitory profile, contrasting with several SMAC mimetics that preferentially target cIAPs. While the specific contribution of XIAP inhibition to immune priming in patients remains to be fully defined, dual cIAP/XIAP antagonism may reduce functional redundancy within the IAP network and thereby amplify immunogenic signaling downstream of TNF-family pathways and regulated cell death. Consistent with this broader therapeutic rationale, other early-phase studies have combined IAP antagonists with PD-1 blockade (e.g., APG-1387 + toripalimab; BI-891065 + ezabenlimab), generally reporting manageable safety with modest objective activity and pharmacodynamic immune readouts that are variable across patients. Importantly, direct cross-trial efficacy comparisons are not appropriate given differences in tumor types, dose/schedule, and the proportion of ICI-naïve versus ICI-refractory populations; nonetheless, taken together, these studies support the feasibility of IAP antagonism as an immunomodulatory partner for checkpoint blockade and reinforce the need for biomarker-led patient stratification to identify the contexts in which IAP inhibition most effectively converts tumors into immunotherapy-responsive disease.

Given the relatively small and heterogeneous cohort typical of a Phase I all-comers trial, some observations are inevitably influenced by individual patient trajectories. Accordingly, the correlative analyses are primarily descriptive and hypothesis-generating. While longitudinal sampling provides insight into treatment-associated changes, the study design does not allow definitive attribution of observed immune features to clinical outcomes. Despite these limitations, our findings are consistent with prior multi-omics studies of ICI across tumor types, which have linked IFN signaling, TCR repertoire dynamics, tumor myeloid/lymphoid composition, and mutational burden with treatment response^76,83–87^. In this context, our results align with established immune correlates of ICI response and extend these observations by suggesting that IAP inhibition may induce similar immune features prior to pembrolizumab exposure. Overall, these findings should be interpreted with appropriate caution and warrant further investigation in larger, prospectively designed studies.

In conclusion, Part A of the ASTEROID trial established that the combination of ASTX660 and Pembrolizumab is safe and biologically active at ≥ DL2, with signs of immune engagement. PK analysis revealed overlap between DL3 and DL4, and based on the integrated safety, efficacy, PK, and PD data, DL3 (150 mg) was selected as the initial RP2D. Recruitment for the Part B expansion phase at this dose started in February 2024, with flexibility to refine the RP2D as additional safety and efficacy data emerge. Importantly, the expansion cohorts will provide the opportunity to validate the candidate biomarkers of response and resistance identified here, with the ultimate goal of improving patient stratification and converting cold tumors into immunotherapy-responsive disease.

## Methods

### Compliance with reporting guidelines

The CONSORT-DEFINE^88^ checklist for early phase dose-finding trials, alongside the study protocol are provided in the Supplementary Information to enhance transparency and ensure accurate reporting of the study’s findings.

### Study overview

ASTEROID is a prospective, open-label, multi-center phase 1 non-randomized adaptive platform study of ASTX660 (tolinapant) in combination with pembrolizumab, consisting of an initial dose escalation phase followed by expansion cohorts in patients with advanced solid tumors (ClinicalTrials.gov Identifier NCT05082259). Data shown are from Part A (dose escalation), which enrolled 23 patients from March 2022 to November 2023, as of the cut-off date, October 24, 2025. Part B (dose expansion) is currently enrolling patients into exploratory cohorts. The trial was conducted across the Drug Development Unit at the Royal Marsden and Cambridge University Hospitals NHS Foundations Trusts, UK.

### Ethics

The protocol, patient information sheet, consent form and all relevant study documentation were approved by the Institute of Cancer Research (ICR)/Royal Marsden Hospital (RMH) Joint Committee for Clinical Research, the local institutional Research Ethics Committee, the Health Research Authority and Medicines and Healthcare products Regulatory Agency. The study was carried out in compliance with the International Conference on Harmonization Good Clinical Practice Guidelines and requirements of the UK Clinical Trials regulations (SI 2004/1031 and SI 2006/1928 as amended). Written informed consent was obtained from all participants, which included the reporting and sharing of individual-level data. Patient IDs were generated for this study. No participant compensation was provided. The study was sponsored and monitored by the ICR.

### Study objectives

The primary objectives of Part A were to propose a recommended dose for Phase II (RP2D) evaluation by establishing the maximum tolerated dose (MTD) of ASTX660 in combination with pembrolizumab and to assess the safety and tolerability of the combination. The secondary objectives were to evaluate the preliminary anti-tumor activity of the combination and characterize the pharmacokinetics (PK) of ASTX660. Tertiary objectives were to characterize the pharmacodynamics (PD) profile of single-agent ASTX660 and the combination with pembrolizumab on the host immune response using blood and tumor samples; to assess putative predictive biomarkers of response and resistance to the doublet combination; and to evaluate the effect of ASTX660 on the tumor microenvironment, including evaluation of inflammatory cytokines downstream of NF-kB.

### Study design

Part A was a proof-of-concept dose exploration trial of oral ASTX660 given once daily for 7 days, followed by intravenous pembrolizumab 200 mg on day eight, in 21-day cycles, until unacceptable toxicity or disease progression. A dose escalation design for up to 18 evaluable patients to determine the MTD of the combination was based on a modified Bayesian Time-to-Event Continual Reassessment Method (TITE-CRM) model^55^. Dose escalation commenced at the minimum anticipated biological effect level–defined starting dose of ASTX660 (90 mg) and progressed up to 180 mg, based on RP2D of prior monotherapy trial (NCT02503423^52^). Core needle biopsies and blood samples were taken at different timepoints for PK and PD analyses. The study scheme is shown in Fig. 1a.

### Patient eligibility

In Part A, adult patients aged ≥18 years must have histologically or cytologically confirmed malignant advanced solid cancer, refractory to conventional treatment, or for which no conventional therapy exists or is declined by the patient. Key inclusion criteria also required that patients had World Health Organization functional status 0 or 1, adequate organ function, and a disease amenable to serial biopsies. Key exclusion criteria were autoimmune disorders requiring immunosuppression. The full list of eligibility criteria can be found in the Supplementary Methods 1.

### Safety evaluation and MTD determination

The incidence and severity of adverse events (AEs) were assessed continuously for all patients using Common Terminology Criteria for Adverse Events (CTCAE) version 5.0. Attribution of a final diagnosis for AEs was per investigator determination based on overall assessments and review of clinical symptoms. All patients receiving at least one dose of investigational medicinal product (IMP), ASTX660 or pembrolizumab, were evaluable for safety. Treatment-related adverse events (TRAEs) were defined as AEs that per investigator review, had a reasonable possibility of being caused by IMP up to 28 days after the last dose. Dose-limiting toxicities (DLTs) were assessed during the first 2 cycles (42 days). A DLT was defined as any event that fulfilled the criteria as listed in Supplementary Methods 1. This included any Grade (G) 3 or greater laboratory changes, except isolated changes of any grade without clinical sequelae or significance that resolves to <G2 within 7 days; any clinically significant AE that results in disruption of the dosing schedule of more than 14 days; and any other toxicity which the investigators deemed to be a DLT at any time. Serious AEs were defined as any AE that met at least one of the following criteria: resulted in death, was immediately life-threatening, required inpatient hospitalization or prolongation of existing hospitalization, resulted in persistent or significant disability/incapacity, or other medically important serious event.

Dose escalation was based on a 2-stage TITE-CRM model. The MTD was defined as the dose with an estimated DLT rate close to a target of 25%. Patients were recruited in cohorts of two starting at Dose Level (DL) 1, with at least two weeks between each patient. When each patient had at least three weeks of assessment without any DLT occurring, patients could be recruited to the next higher DL dose. If no DLTs were observed for the first eight patients (at DLs 1, 2, 3 and 4), subsequent patients would be recruited continuously at the highest dose (DL4). Once the first DLT occurred, the TITE-CRM model would be used by the Safety Review Committee (SRC) to recommend the best dose for subsequent patients with all the information accumulated so far.

Patients who did not complete the 6-week DLT assessment period, for reasons other than toxicity (e.g., withdrawal of consent, rapid clinical deterioration) were replaced. The target sample size was 18 evaluable patients to enable adequate collection of paired evaluable tumor and blood samples for PD analyses and was anticipated to be sufficient to determine the MTD as per the TITE-CRM design.

### Efficacy evaluation

All patients who met the eligibility criteria, received at least two cycles of combination treatment and had a baseline assessment of disease were evaluable for response. Efficacy analysis included assessment of objective response (OR) by immune-modified Response Evaluation Criteria in Solid Tumors (imRECIST) criteria^89,90^. Tumor burden assessments by computed tomography (CT) and/or magnetic resonance imaging (MRI) were performed after Cycle 2, then after every 3 cycles (9 weeks) or more frequently, when clinically indicated. After 6 cycles, assessments could be reduced to every 4 cycles at the investigator’s discretion. Patient narratives were provided by the recruiting principal investigators.

### Recommended phase 2 dose (RP2D) determination

SRC meetings were held monthly to review all available clinical data, the TITE-CRM’s recommended dose, and make decisions on the appropriate dose for the next cohort of patients. The RP2D was determined by considering the safety and tolerability profile, alongside available efficacy, PK and PD data. Part B has commenced recruitment at RP2D of 150 mg, which could still be adjusted if necessary, according to emerging safety data.

### Blood sample collection and processing

#### PK Studies

Whole blood was collected at the following timepoints:

- 3 samples on C1D7: (1) within 30 min pre-dose; (2) at 1 h ± 30 min post-dose; (3) at 4 h ± 30 min post-dose
- 3 samples on C2D7: (1) within 30 minutes pre-dose; (2) at 2 h ± 30 min post-dose; (3) at 6 h ± 30 min post-dose

ASTX660 levels in plasma were analyzed as previously reported^52^ using a validated LC/MS-MS method with a dynamic range of 1–500 ng/mL. PK parameters were estimated with standard noncompartmental methods using Phoenix WinNonlin 6.4 (Pharsight Corporation). Statistical analyses were performed via R version 3.5.1.

#### Flow cytometry

Whole blood was collected by venepuncture into EDTA blood collection tubes, pre-dose at the following timepoints: C1D1, C1D7, CXD1 (day 1 of each subsequent cycle) and at the end of the trial. Samples underwent flow cytometry to identify DC and T cell subpopulations using the following commercially available kits:

1. T cell panel of 10 markers (DURAclone IM, Beckman Colter): CD45RA, CD197 (CCR7), CD28, CD279 (PD1), CD27, CD4, CD8, CD3, CD57, CD45
2. DC panel of 12 markers (DURAclone IM, Beckman Colter):
  DC population markers: CD1c, CD11c, CD16, CD45, CD123, C-type lectin domain containing 9 A (CLEC9A), human leukocyte antigen-DR isotype (HLA-DR)
  Lineage exclusion markers: CD3, CD14, CD19, CD20, CD56

Samples were incubated in the IM T Cell and DC Subsets tubes provided with the kits to stain the different cell populations. Erythrocytes were removed by lysis, and leukocytes are acquired on the Beckman Colter DX Flex according to standard procedures. Data was analyzed using Beckman Colter Kaluza C software. Results were reported as a percentage of positive cells. Statistical significance of changes between C1D1 and C1D7 was determined using a paired *t* test, with a *p*-value <0.05 considered statistically significant. See Supplementary Fig. 18 for gating strategies.

#### Plasma and PBMC preparation from whole blood

Whole blood was collected by venepuncture into a BD® Vacutainer^TM^ CPT tube and centrifuged within 2–4 h at room temperature in a horizontal rotor (swing-out head) for a minimum of 20 min at 1500-1800 x *g*, without brake (or low brake – deceleration set to 1). The top clear plasma layer was pipetted off without disturbing the mononuclear layer and transferred into 5 × 2 mL cryovial tubes. Plasma sample aliquots were stored at −80 °C. Next, the PBMC cell layer was pipetted into a 15 mL conical centrifuge tube and mixed with PBS to bring the volume up to 10 mL. This was centrifuged at room temperature for 10 mins at 300 x *g*, with brake. The supernatant was discarded, and the cell pellet resuspended in 1 mL of chilled Synth-A-Freeze® Cryopreservation Medium, which was then transferred into a cryovial. The vial was placed in a CoolCell® LX freezing container and kept at − 80 °C for at least 4 h before transferring into a sample storage box.

#### Nucleic Acid Extraction from PBMCs

PBMCs from whole blood taken at C1D1, C1D7, and C2D7 were separated from plasma as described above. 0.4 mL of PBMCs in medium was centrifuged at room temperature for 2 min at 1200 x *g*. RNA extraction was performed using the RNeasy® Mini Kit (Qiagen), and subsequent genomic DNA extraction using QIAmp DNA Mini Kit (Qiagen) as per the manufacturer’s instructions.

#### cIAP1 protein analysis in PBMCs

PBMCs from whole blood collected at C1D1, C1D7, C2D1 and C3D1 were shipped frozen to Astex Pharmaceuticals, UK, for cIAP1 protein analysis. Protein lysates were quantified using a bicinchoninic acid assay (Thermo Fisher). cIAP1 protein levels were measured relative to glyceraldehyde-3-phosphate dehydrogenase (GADPH) using a custom-made Meso Scale Discovery Prototype Duplex Assay plate on the MESO™ Sector S 600MM reader.

#### Plasma protein profiling

Plasma samples from C1D1, C1D8, C2D1, and C2D8 were analyzed by Rules-Based Medicine (Q2 Solutions, Austin, TX, USA). Plasma protein concentrations were quantified using a 130-protein panel assay (Human ExplorerMap v1.0).

#### Plasma protein data pre-processing

The original data were processed to deal with batch effects, missing values, and values outside the detection limits. Measurements reported as ‘<X’ or ‘>X’ were converted to ‘X’ (‘ <X’: X is equal to Lower Limit of Quantification). Analytes with a zero coefficient of variation were removed.

For ASTX660-01 plasma protein data, to remove any association of Response and the detection limits specific to the analysis batch, measurements outside the dynamical range were set to the maximum/minimum lower/upper limit of detection of the entire cohort, if the measurement was below/above the limit. If these substitutions were made in more than 90% of the cases, the corresponding analyte was removed from the downstream analysis. All samples with more than 90% of missing values were removed. Likewise, low-quality samples were removed. Otherwise, the missing values were imputed as the median of all other samples per analyte. Paired samples from 62 patients with peripheral T-cell lymphoma (PTCL), in the first treatment cycle, were analyzed. Samples collected before treatment or within 2 h after dosing were classified as “early” and designated as baseline (C1W1Early). “Late” samples were collected on day 2 (one day after the first dose), day 7, day 8, or day 9, corresponding to the end of seven consecutive daily doses in cycle 1 (C1W1Late). When multiple samples from the same subject met the inclusion criteria for a timepoint, the sample closest to day 1 (for C1W1Early) or day 8 (for C1W1Late) was selected. If two samples had comparable proximity to the target day, the one with fewer missing values was prioritized. Finally, within the ASTX660-01 study, subjects with Complete (CR) or Partial (PR) Response, and Stable Disease (SD) for over 6 months were considered responders, whereas subjects with Progressive Disease (PD) and SD for less than 6 months were considered non-responders. The number of responders was 22. The number of non-responders was 40.

#### Statistical analysis of plasma protein data

Linear Mixed-Effect Models were used to account for time-dependent subject profiles of individual proteins (i.e., subject IDs were used as random intercepts). In addition, the dose of ASTX660 was used as a confounder in the model. Other confounders (such as Age, Sex, Tumor type) were excluded as an independent Type III Sum of Squares ANOVA demonstrated their inability to reduce unexplained variance (F-test) in a significantly large proportion of protein models. Protein levels for responders and non-responders were conditioned separately on the four time-points. Proteins with more than 50% non-varying values per time-point, as well as proteins with model fits producing singular solutions, were removed. Namely, each protein concentration was modeled using the “formula” (using R programming language notation): log_2_(Concentration) ~ Response[Binary] * Time[2 levels] + ASTX660Dose + (1|Patient).

For the ASTX660-01 analysis, the same approach was used. The confirmed confounders were Age, Subtype, and other higher-order terms: log_2_(Concentration) ~ Response[Binary] * Time[4 levels] + Age + PTCLSubtype[7 levels] + Time:Age + Response:Age:Sex + (1|Patient). Given the higher-order terms, all population extrapolations derived from the differential expression results are to be assumed for the average age.

The subject demographic data and best clinical response, used for the ASTX660-01 plasma protein analysis, was retrieved from the active clinical study database on 16^th^ May 2023.

#### Software and reproducibility

R version 4.3.3 was used for statistical analysis. Type III Sum of Squares ANOVA analysis was performed using R package *afex* (version 1.3_1), *lme4* (version 1.1_35), *lmerTest* (version 3.1_3). The pre-processing was performed in Python (3.12.8) using *pandas* (2.2.3). Volcano plots were produced using the R package *ggplot2* (3.5.1).

### Tumor biopsy collection

Mandatory core needle biopsies, [gauge size 14–18], were taken under ultrasound guidance on C1D1 and C1D7. C2D1 and end-of-trial (EOT) biopsies were optional. All efforts were made to have the paired biopsies taken by the same radiologist and from the same site of metastasis. At each timepoint, patients could have up to seven biopsies in the sequence listed below, dependent on their consent, tolerance and safety for the procedure.

***Sample 1: Fixed in formalin and paraffin embedded (FFPE) blocks for hematoxylin and eosin (H&E), IHC and mIF staining****.* Core biopsy samples were immediately fixed in 10% neutral buffered formalin (NBF) at room temperature for 16–24 h then processed through paraffin wax. FFPE blocks were used for IHC and mIF staining.

***Sample 2: Fresh frozen for DNA and RNA sequencing****.* Biopsies were carefully placed flat in cryomolds filled with Optimal Cutting Temperature compound and immersed in isopentane. Isopentane was pre-cooled in a metal container placed in dry ice at least 15 min prior. After freezing, samples were wrapped in labeled foil, transferred in dry ice and stored at −80 °C until use. An initial 4 µm-thick longitudinal section of the fresh frozen biopsy was cut using a cryostat, stained with H&E, and visually analyzed for tumor content by a pathologist (B.G.). Only biopsies with sufficient tumor content ( >20%) were further sectioned and collected for nucleic acid extraction. Genomic DNA and total RNA were purified using AllPrep DNA/RNA Mini Kit (Qiagen) according to the manufacturer’s instructions. DNA/RNA quantity and quality were confirmed using the Agilent 4200 TapeStation system. RNA samples were aliquoted for bulk RNAseq and FFPE-suitable Unique Molecular idEntifier-based-T Cell Receptor sequencing (FUME-TCRSeq) according to minimum requirements set out by Genewiz (quantity required ≥ 250 ng; RNA Integrity Number (RIN) >6.0) and FUME-TCRseq protocol (quantity required ≥ 5 ng)^91^. DNA samples were sent to Novogene (Cambridge, UK) for whole exome sequencing.

***Samples 3 and 4:*** Additional fresh biopsy samples were taken from breast cancer patients for patient-derived xenograft (PDX), patient-derived organoid (PDO) generation, and ex vivo assays.

***Samples 5 and 6:*** Additional biopsies from breast cancer patients were fixed for novel exploratory analyses, including proximity ligation assay and additional molecular characterization.

***Sample 7: Snap frozen for cytokine analyses***. Biopsies were collected into a Bijou® tube and immediately placed in liquid nitrogen or an alcohol dry ice bath, then stored at − 80 °C. It was later decided not to proceed with this analysis as most patients did not have this last sample taken.

### Transcriptomics analyses

#### RNA next generation sequencing (NGS)

Paired tumor RNA samples were sent to Genewiz (Azenta Life Science). Sequencing was performed using the Illumina NovaSeq platform with a 2 × 150 base pair configuration, generating 100 million paired-end reads per sample after ribodepletion.

#### Molecular subtyping of breast cancer patients

Breast cancer patients were classified into molecular subtypes using the PAM50 gene expression classifier^56^ on the RNA sequencing data from their respective tumor samples. These molecular subtypes were then compared to the tumor histopathological characteristics.

#### Principal component analysis (PCA) of pre- and post-treatment samples

PCA was performed on RNA sequencing data to capture gene expression differences between patient samples collected before and after treatment. Analyses were restricted to patients whose biopsies had a tumor content >85% to enable accurate interpretation of clustering by minimizing the confounding effects of tumor purity.

#### Gene set enrichment analyses (GSEA)

GSEA^92^ was conducted to explore differential gene expression profiles before and after ASTX660 treatment, as well as between responders and non-responders. Gene sets were obtained from the Molecular Signatures Database (MSigDB) hallmark gene set collection^60^. Hallmark pathway enrichment was assessed using preranked gene set enrichment analysis implemented in the R package fgsea. Genes were ranked by log_2_FC × −log_10_(unadjusted differential-expression P-value). Differential gene expression was compared between responders and non-responders at pre-treatment C1D1 and post-treatment C1D7, respectively. These were unpaired comparisons between independent patient groups, with the response group included as the model factor (Supplementary Data 2c, d). Differential gene expression was compared between post-treatment C1D7 and pre-treatment C1D1 samples within responders and non-responders, respectively. These were paired longitudinal comparisons, with patient identity included as a blocking factor to account for repeated sampling from the same individuals (Supplementary Data 2e and f). All gene-level differential-expression comparisons were performed using two-sided Wald tests implemented in the R package DESeq2. Gene-level *P*-values were adjusted for multiple comparisons within each differential-expression analysis using the Benjamini–Hochberg false-discovery-rate procedure. For the Hallmark analyses, direction-specific enrichment *P*-values were estimated using the adaptive multilevel split Monte Carlo procedure implemented in fgsea. *P*-values were adjusted for multiple comparisons across the Hallmark gene sets tested within each analysis using the Benjamini–Hochberg procedure.

#### FUME-TCRseq

FUME-TCRseq was performed on RNA from tumor and PBMCs as previously described^91^. TCRseq analysis was performed using the Immunarch R package^93^. For diversity analysis, PBMC repertoires were randomly subsampled to 3000 clonotypes, and tumor repertoires subsampled to 385 clonotypes. Three samples with negligible tumor repertoires were excluded. Two patients with germline BRCA mutations and 1 patient with a PMS2 mutation (Lynch syndrome) were excluded from analysis.

### Immunogenomics analyses

#### Genomic DNA whole exome sequencing (WES)

Tumor genomic DNA extracted from C1D1 biopsies were sent to Novogene (Cambridge, UK) for quality control checks and WES at 150X depth. C1D7 DNA sample was sent instead if a patient’s C1D1 sample did not meet the minimum requirements. In addition, DNA from PBMCs taken on C1D1 were sequenced at 50X depth, enabling the identification of somatic variants through comparison to germline DNA. Most analyses were performed on the ALMA high-performance computing (HPC) cluster. All immunogenic analyses were conducted using R (v4.3.1).

#### Somatic variant calling and annotation

Raw FASTQ files were processed using the nf-core/Sarek pipeline (v3.4.4, commit g5cc3049)^94^, executed via Nextflow (v24.04.2). Variant calling was carried out with Mutect2 (GATK v4 4.6.1.0)^95^ for somatic single-nucleotide variants (SNVs) and indels, and CNVkit^96^ for copy number variation analysis. Functional annotation was performed using the Variant Effect Predictor (VEP, v110)^97^.

The analysis was conducted against the GRCh38 reference genome using resources hosted on s3://ngi-igenomes/. Exome capture regions were defined by the Agilent SureSelect Human All Exon V6 r2 Covered Targets BED file (S07604514_hg38.bed).

#### Variant filtering

High-confidence somatic variants were retained by filtering Mutect2 VCFs using BCFtools (v1.16) to extract only records flagged as PASS. These filtered VCFs were used for downstream analyses, including TMB, immune selection, and neoantigen prediction.

#### HLA Typing

HLA class I genotypes (HLA-A, HLA-B, HLA-C) were inferred using the nf-core/hlatyping pipeline (v2.0.0), employing OptiType^98^ as the HLA prediction engine. The inferred alleles were used for SOPRANO and pVACseq v7.1.2.

#### Immune selection analysis

Immune selection on somatic variants was assessed using SOPRANO (Selection On PRotein ANnotated regiOns), a web-based tool for dN/dS estimation in immune-exposed versus neutral genomic regions^76^. Filtered and VEP-annotated Mutect2 VCFs and sample-specific HLA genotypes were submitted via the SOPRANO portal (https://software.icr.ac.uk/app/soprano). Immune-focused selection was quantified with SOPRANO using the immunopeptidome (“ON”) model. For each tumor (one per patient), SOPRANO returned the patient-level immune dN/dS estimate (ON_dNdS), its 95% confidence interval (ON_Low_CI, ON_High_CI), and the number of coding mutations mapping to the immunopeptidome (ON_Mutations). SOPRANO reports an undetermined dN/dS when immunopeptidome counts are too low (synonymous = 0). These samples were displayed as “und.” in the bar plot (with the corresponding ON_Mutations value shown) and were excluded from CI calculations and any summaries of dN/dS point estimates; they were retained for mutation-burden analyses.

#### Neoantigen prediction

Candidate neoantigens were predicted using pVACseq, part of the pVACtools suite (version 5.4.2)^99^. Mutect2 VCFs were re-annotated using VEP (v114) with neoantigen-specific plugins (Frameshift, Wildtype) and flags: --pick, --symbol, --terms SO, --hgvs, --biotype, --tsl. The re-annotated VCFs and corresponding HLA genotypes were used as input for pVACseq. pVACseq was run with epitope lengths of 8–11 amino acids, using NetMHCpan and MHCflurry for binding predictions. Binding affinity thresholds were set at 500 nM. Cleavage site prediction was enabled using NetChop (cterm method). Neoantigens were prioritized based on predicted binding affinity, mutational impact, and transcript support when available.

TMB was calculated as the number of PASS-filtered somatic mutations per megabase of targeted sequence, based on the Agilent SureSelect Human All Exon V6 capture region (60.5 Mb).

#### Software and reproducibility

All workflows were executed on the ALMA HPC cluster under SLURM scheduling using containerized tools via Singularity. Key tools included nf-core/Sarek, nf-core/hlatyping, GATK, Strelka2, Manta, CNVkit, VEP (v110/v114), BCFtools (v1.16), pVACtools, SOPRANO, and Java JDK 15. Data processing and figure generation were performed in R (v4.3.1).

### Cell death markers immunohistochemistry

IHC was used to analyze changes in cIAP1, and five cell death markers: RIPK1, RIPK3 and MLKL, cleaved caspase 8 (cC8), and cleaved caspase 3 (cC3). Additionally, changes of the proliferation marker Ki67 was also analyzed.

Primary antibodies for cIAP1, RIPK1, RIPK3 and MLKL, which have not previously been used in clinical samples, were first validated in human cell and tissue blocks. The HT29 (human colorectal adenocarcinoma) cell line was used as a positive control for RIPK1, RIPK3 and MLKL, based on the strong expression of these proteins, as determined by Western blot in vitro. Accordingly, negative controls used were HT29 cells treated with a RIPK1 proteolysis targeting chimera (PROTAC) degrader (R1-ICR-5)^100^, HT29 RIPK3 knockout cells, and NCI-H522 cells (MLKL-negative human lung adenocarcinoma cell line). MDA-MB-231 cells treated with DMSO and SM164 (a SMAC mimetic) were used as positive and negative controls for cIAP1, respectively.

Single-plex IHC was performed on FFPE control tissue sections for each of the markers individually to ascertain the conditions of heat-induced epitope retrieval (HIER), primary antibody incubation time, primary antibody concentration and the requirement of secondary amplification for each target before transfer of these conditions to the BOND RX using the BOND Polymer Refine Detection System (Leica Biosystems). Briefly, the slides were deparaffinized, rehydrated and subjected to microwave HIER in both citrate buffer solution at pH 6.0 or EDTA at pH 9.0 (Invitrogen), first for 1 min and 30 s at 1000 watts and then 15 min at 100 watts, to establish optimal HIER. This was followed by peroxidase [3% hydrogen peroxide (H_2_O_2_)] and protein block [serum-free protein block (DAKO)], then primary antibody incubation for 1 h at room temperature using serial dilutions [in serum-free protein block (Leica)] to establish the optimal one. SignalStain Boost IHC. Horseradish peroxidase (HRP) [mouse (#8125) or rabbit (#8114)] (Cell Signaling Technology) was used according to the primary antibody, and Leica Novolink polymer was added if the reaction required additional amplification (for mIF optimization only). 3,3′-diaminobenzidine (DAB) solution (DAB kit Leica Novolink) was applied in a dark chamber, slides were rinsed in deionized water, counterstained with hematoxylin, dehydrated and mounted with Cytoseal 60 (Fisher Scientific, UK). After drying overnight, they were scanned at 20X with NanoZoomer S60 Hamamatsu Scanner (Hamamatsu Photonics, Japan) and evaluated to establish the working conditions for BOND staining. Primary antibodies and staining conditions are detailed in Supplementary Information.

Following manual optimization for each marker, control slides were stained at the BOND to confirm the staining conditions before proceeding with the cohort samples. The cohort slides were baked at 62 ^o^C for an hour and then loaded into a BOND rack for the staining protocol, underwent deparaffinization (Leica BOND Dewax Solution), rehydration and subjected to HIER with the optimized Leica BOND Epitope Retrieval Solution for each primary antibody - ER1 [citrate (pH 6.0)] or ER2 [EDTA (pH 9.0)]. Primary antibodies were diluted in Leica BOND primary antibody diluent (AR9352, Leica), incubated for 15 min followed by post-primary, polymer reagent, DAB chromogen and hematoxylin counterstain. Slides were then removed from the BOND and submitted to dehydration and mounting with Cytoseal 60 (Fisher Scientific). After drying overnight, they were scanned at 20 x using a Nano Zoomer S60 Hamamatsu Scanner. Digital image analysis was performed using QuPath^101^.

Tumor cell areas were manually annotated (avoiding necrotic areas), and cell detection was performed using QuPath (version 0.5.0) with the built-in *Cell Detection* command based on the Watershed cell detection algorithm. The following parameters were used for the detection: detection image = Hematoxylin optical density (OD), requested pixel size = 0.5 μm, background radius = 8 μm, median filter radius = 0 μm, sigma = 1.5 μm, minimum cell area = 10 μm², maximum cell area = 400 μm², threshold = 0.1, and cell expansion = 2.5 μm.

Cell death marker expression was quantified by assigning each sample a pixelwise H-score (pixH) of 0–300 as previously described^102^. The following DAB thresholds were set for each biomarker, classifying positive pixels as low (1 + ), medium (2 + ) or high (3 +):

**Table:** 

**CASP3:**	DABthresholds = [0.14,0.25,0.55] //1 + , 2 + , 3 + DAB
**CASP8:**	DABthresholds = [0.14,0.25,0.55] //1 + , 2 + , 3 + DAB
**MLKL:**	DABthresholds = [0.12,0.22,0.45] //1 + , 2 + , 3 + DAB
**RIPK1:**	DABthresholds = [0.14,0.25,0.55] //1 + , 2 + , 3 + DAB
**RIPK3:**	DABthresholds = [0.14,0.25,0.55] //1 + , 2 + , 3 + DAB

The total area of the DAB high (Hp), DAB medium (Mp), DAB low (Lp) and DAM negative (Hp) pixels were input into Eq. (1) to give the final pixH score.

1 $${{{\rm{pixH}}}}=100\frac{3{{{\rm{Hp}}}}+2{{{\rm{Mp}}}}+{{{\rm{Lp}}}}}{{{{\rm{Hp}}}}+{{{\rm{Mp}}}}+{{{\rm{Lp}}}}+{{{\rm{Np}}}}}$$

Each sample was then categorized into negative (pixH = 0–50), weak (50–100), moderate (100–200) and strong (200–300) expression.

Detected cells were further analyzed for DAB signal intensity to identify cIAP1-positive cells. To classify cells as positive or negative for cIAP1 expression, an object classifier was created within QuPath using the Cell DAB optimal OD mean feature. A threshold of 0.07 was applied, with cells showing values above this threshold classified as cIAP1-positive.

Ki67 expression was assessed as positive nucleus detected in tumor cells areas manually annotated and expressed as percentage of tumor cells positive for Ki67 as described previously^103,104^.

### PD-L1 Immunohistochemistry

IHC was performed on 4 μm FFPE tissue sections using a rabbit monoclonal anti-PD-L1 antibody (#13684; clone E1L3N; Cell Signaling Technology) on the BOND RX automated staining platform (Leica Biosystems). This antibody clone has been validated and shown to be comparable with other FDA-approved IHC assays^105^. HIER was carried out using BOND Epitope Retrieval Solution 2 (pH 9.0; #AR9640, Leica Biosystems) for 10 min, followed by incubation with the anti-PD-L1 antibody at a 1:250 dilution for 30 min at room temperature. Detection was performed using the BOND Polymer Refine Detection Kit (#DS9800, Leica Biosystems). PD-L1 IHC-stained slides were scanned at high resolution (200 ×) using the VS200 digital slide scanner (Olympus, Tokyo, Japan). All cases were evaluated by a pathologist (BG). Membranous and cytoplasmic PD-L1 quantification for each sample was determined using modified H-scores to determine the overall percentage of membranous and cytoplasmic PD-L1 positivity across the entire stained tumor sample, yielding a range from 0 to 300, according to the equation below:

2 $${{{\rm{Modified}}}} \; {{{\rm{H}}}}-{{{\rm{score}}}}=	[( \,\%\, {{{\rm{of}}}} \; {{{\rm{weak}}}}\; {{{\rm{staining}}}}) \times 1]+[( \,\%\, {{{\rm{of}}}} \; {{{\rm{moderate}}}} \; {{{\rm{staining}}}}) \times 2] \\ 	+ [( \,\%\, {{{\rm{of}}}} \; {{{\rm{strong}}}} \; {{{\rm{staining}}}}) \times 3]$$

### Multiplex immunofluorescence

To assess changes in immune cell profiles, we designed 3 mIF panels of 6 biomarkers each [5 unique and 1 tumor marker: pan-cytokeratin (panCK)], plus 4′,6-diamidino-2-phenylindole (DAPI), for interrogation by the PhenoImager HT (Akoya) platform.

**Panel 1 (T cell panel):** CD4, CD8, FOXP3, Granzyme B, PD-1, panCK

**Panel 2 (DC panel):** CD11c, CD141, CD123, CD28, CD86, panCK

**Panel 3 (B cell/vasculature/myeloid panel):** CD20, CD31, CD68, CD206, CCR2, panCK

Single-plex IHC was performed on FFPE tissue sections for each unique target individually as per the protocol above before panel optimization. To establish each primary antibody position in the panel, a single IHC was performed with 3 control slides at the optimized conditions for the primary antibody, with the following modifications in HIER:

- One slide to evaluate antibody position 1, submitted to 1 cycle of HIER
- One slide to evaluate antibody position 3, submitted to 3 cycles of HIER
- One slide to evaluate antibody position 5, submitted to 5 cycles of HIER

After the 5^th^ HIER cycle, the IHC was finished as per the optimized manual protocol, and the slides were evaluated to access in which position the primary antibody should be located in the panel to ensure optimal staining. This process was repeated for all primary antibodies used for mIF, and the designed panels were tested until the best combination of antibodies position and Opal pairing was achieved.

FFPE tissue sections underwent deparaffinization, rehydration, and HIER in the BOND RX. The primary antibodies were incubated for 30 minutes, followed by Opal Polymer HRP secondary antibodies and the respective paired Opal fluorophore from the Opal 7-Plex system (Akoya Bioscience). After Opal deposition, the slides underwent another round of HIER to remove the primary/secondary antibody complexes, preparing them for the next marker. This process was repeated until all markers were labeled. Finally, spectral DAPI (Akoya Bioscience) was added for 5 min at room temperature, and the slides were mounted in X6 Immu-Mount (Fisher Scientific), then cured in the dark at room temperature for 1 h.

Slides were scanned by the Perkin Elmer Vectra Polaris scanner (Akoya Bioscience) using PhenoImager HT 2.0 software. Digital image analysis was performed in HALO Image Analysis Platform version 3.6.4134 (Indica Labs) using the Highplex FL v4.2.14 module for cell segmentation, mark up, phenotyping and quantification. Briefly, the mIF images were manually annotated to remove necrotic areas or other artifacts such as folding and detached fragments. The thresholds for cell detection and segmentation, such as minimum nuclear size, nuclear roundness, DAPI intensity, cytoplasm radius and membrane segmentation aggressiveness, were established according to the samples’ morphology features to be as representative as possible. For cell phenotyping, the positivity of each panel marker was adjusted and reviewed by the IPU pathology team based on the intensity signal/pattern of staining of the markers, followed by setting up all cell phenotypes of interest using different combinations of the panel markers positivity/negativity. Cell quantification was performed according to previously established markers thresholds/cell phenotypes, and results were reported as the number of positive cells for each of them.

Full list of antibodies for IHC and mIF is provided in Supplementary Table 3.

### Tumor-infiltrating lymphocyte (TIL) assessment

H&E samples were visually assessed by a pathologist (T.L.S.) who was blinded to the patient profiles. Percentages of area infiltrated by lymphocytes within the tumor and adjacent stroma were classified as cold ( <10%), moderate (10–50%), or hot ( >50%), as previously described^106^.

### In situ proximity ligation assay (PLA)

FFPE slides were initially baked for 30 min at 60 °C, followed by dewaxing using the standard Leica Bond dewaxing protocol on the Leica BOND RX automated staining system. Next, antigen retrieval was carried out using a standard Leica Bond antigen retrieval protocol, heating the samples to 100 °C for 40 min in ER2 solution. Following antigen retrieval, the tissue samples were encircled using an ImmEdge Hydrophobic Barrier PAP Pen (Vector laboratories). For all incubations, the samples were placed within a humidity chamber to avoid evaporation. Samples were then blocked for 1 h at 37 °C using Naveni Block (Navinci Diagnostics). Following blocking, the samples were incubated with Naveni Plex Omni conjugates targeting CD3ε (CD3ε.F1.10152), Zap70 (ZAP70.F2.10292), PD1 (PD1.D1.10157), PD-L2 (PD-L2.D2.10158), Lck (LCK.B1.10271), Zap70 (ZAP70.B2.10306), cleaved caspase-8 (cleaved Caspase-8.C1:10372), RIPK1 (RIP.C2:10373), PD1 (PD1.A1.10034), SHP2 (SHP2.A2.10304), P-Tyrosine (P-Tyrosine.G1.10291), STAT3 (STAT3.G2.10355), PD1 (PD1.H1.10030), PD-L1 (PD-L1.H2.10033), RIPK3 (RIPK3.I1:10376), RIPK1 (RIP.I2:10377) diluted in Naveni Diluent (Navinci) overnight at 4 °C. Slides were then briefly rinsed using 37 °C TBS-T (Thermo Fisher Scientific) twice, followed by a 15 min wash with 37 °C 1X TBS-T. The samples were then incubated Reaction 1 mixture (Naveni Plex: Omni Buffer 1 / A-I (1x), Naveni Enzyme 1 (1x) (Navinci)) for 30 min at 37 °C. After the incubation, the slides were briefly rinsed and then washed for 5 min in 1X TBS-T. Next, the samples were incubated with Reaction 2 mixture (Naveni Reaction buffer 2 (1x), Naveni Enzyme 2 (1x) (Navinci) for 90 min at 37 °C. After the incubation, the Reaction 2 mixture was discarded, replaced with Post-block mixture (Naveni post-block (1x) diluted in Naveni diluent (Navinci)) and incubated for 30 min at 37 °C. The Post-block mixture was then discarded and replaced with Naveni Plex: Omni Detection Mixture ABC (Naveni Plex: Omni Detection Mix / ABC (1x) (Navinci), DAPI (0,4 µg/ml) (Fisher Scientific)) and incubated for 30 min at 37 °C. The excess detection mix was removed by washing the slides twice in 1X TBS-T for 5 min and once in 0.1 X TBS-T. Slides were then dried using a slide centrifuge and mounted using Fluoroshield (Sigma-Aldrich).

#### Detection oligonucleotide cycling

The signals generated from isPLA were visualized over three cycles using three separate detection mixes (Naveni Plex: Omni Detection Mix / ABC (5x), Naveni Plex: Omni Detection Mix / DEF (5x), Naveni Plex: Omni Detection Mix / GHI (5x)). After imaging, the slides were demounted by incubation in 1x TBS-T at 37 °C until the coverslip was released. Following coverslip removal, the detection oligonucleotides were removed from the samples using the removal buffer (Navinci). Next, the samples were incubated with the next detection mixture (Naveni Detection Mix (1x) (Navinci), DAPI (0,4 µg/ml) (Fisher Scientific) and incubated for 30 min at 37 °C. The excess detection mix was removed by washing the slides twice in 1X TBS-T for 5 min and once in 0.1X TBS-T. Slides were then dried using a slide centrifuge and mounted using Fluoroshield (Sigma-Aldrich). The process was done once for each detection mix.

#### Controls

For evaluation of cell death protein interactions, fixed cell blocks of HT29 cells treated for 4 h with DMSO or T/A/E [combination human TNF (100 ng/mL), ASTX660 (1 µM) and Emricasan (5 µM)] were used as negative and positive controls, respectively.

#### Imaging

All images were acquired using an Olympus VS200 scanner controlled by the Olympus SlideView software. The camera used was a Hamamatsu Orca FUSION Camera, and samples were illuminated using an XCITE Novem light source. The objective used was a UPLXAPO 20x / 0,80. Each sample was imaged in four cycles, each time with DAPI, FITC, CY3 and CY5 channels. The first three cycles correspond to the three Detection mixes; the fourth cycle images autofluorescence. Following image acquisition, the four separate layers were merged into a single-layered image using the ‘Combine Channels of Multi-Layer Image’ function in Olympus VS200 Desktop software. Images were visualized on QuPath with the following adjusted channel (min, max) settings: DAPI (0, 29190), RIPK1:cC8 (0.163, 3716), RIPK1:RIPK3 (11.1, 2600); gamma = 1 for all channels.

### Statistics and reproducibility

For Part A of the ASTEROID trial, a target sample size of 18 evaluable patients was determined to enable adequate collection of paired evaluable tumor and blood samples for pharmacodynamic analyses, and this target sample size was anticipated to be sufficient to determine the MTD as per the trial design. Dose-finding decisions were based on TITE-CRM, implemented using the dfcrm (version 0.2) and dtpcrm (version 0.1) packages in R (version 4.4.1, R Foundation for Statistical Computing). Survival analyses were performed using the survival package (version 3.8) with Kaplan-Meier curves generated via ggsurv. Descriptive summaries were produced using gtsummary (version 2.2.0), and additional figures were created using ggplot2 (version 3.5.2). No novel computational code or algorithms were developed for this study. Details of the parameters for the TITE-CRM model are included in the study protocol and Statistical Analysis Plan v1.0. No patients were excluded from safety evaluation (total *n* = 23); 2 patients were excluded from MTD/RP2D evaluation due to incorrect dosing and withdrawal of consent. 3 patients were excluded from response evaluation due to rapid disease progression (within 6 weeks) since there was not enough time for combination drug exposure to confidently assess treatment response. (Fig. 1b).

PK parameters were estimated with standard noncompartmental methods using Phoenix WinNonlin 6.4 (Pharsight Corporation). Statistical analyses were performed using R version 3.5.1.

Certain tumor samples were excluded from pharmacodynamic analyses due to reasons summarized in Supplementary Data 1, e.g., poor tumor content, failed quantity and quality control. IHC analysis of cell death markers was performed using QuPath (version 0.5.0). Multiplex immunofluorescence digital image analysis was performed in HALO Image Analysis Platform version 3.6.4134 (Indica Labs) using the Highplex FL v4.2.14 module for cell segmentation, mark up, phenotyping and quantification. Plasma protein statistical analysis was performed using R version 4.3.3. Type III Sum of Squares ANOVA analysis was performed using R package afex (version 1.3_1), lme4 (version 1.1_35), lmerTest (version 3.1_3). The pre-processing was performed in Python (3.12.8) using pandas (2.2.3). Volcano plots were produced using the R package ggplot2 (3.5.1). RNAseq analysis was performed using R version 4.4.2. TCRseq analysis was performed using the Immunarch R package [Nazarov V, T. V., et al. immunarch: Bioinformatics Analysis of T-Cell and B-Cell Immune Repertoires. (2023)]. All workflows for WES analysis were executed on the ALMA HPC cluster under SLURM scheduling using containerized tools via Singularity. Key tools included nf-core/Sarek, nf-core/hlatyping, GATK, Strelka2, Manta, CNVkit, VEP (v110/v114), BCFtools (v1.16), pVACtools, SOPRANO, and Java JDK 15. Data processing and figure generation were performed in R (v4.3.1). Statistical analyses of cIAP1 levels, mIF, and flow cytometry were performed using GraphPad Prism 10.

### Reporting summary

Further information on research design is available in the Nature Portfolio Reporting Summary linked to this article.

## Supplementary information

Supplementary Information

Description of Additional Supplementary File

Supplementary Data 1

Supplementary Data 2

Supplementary Data 3

Reporting Summary

Transparent Peer Review file

## Source data

Source Data

## Data Availability

The full study protocol and Statistical Analysis Plan are provided as Supplementary Notes 1 and 2. De-identified sequencing data have been deposited at the European Genome-phenome Archive (EGA), which is hosted by the EBI and the CRG, under accession numbers EGAS50000002043EGAS50000002044EGAS50000002059. Requests for sequencing data access must be made through EGA and will be reviewed by the ASTEROID Data Access Committee (EGAC50000001005). Further information about EGA can be found at https://ega-archive.org and The European Genome-phenome Archive in 2021: https://doi.org/10.1093/nar/gkab1059. Requests for the clinical and translational data from patient samples must provide a clinically relevant rationale in adherence with the intent of the study and patients’ consent. These will be reviewed by the corresponding authors and the ICR to determine whether the request is subject to any intellectual property, ethical and/or confidentiality considerations. A prompt response will be provided to such requests. Patient identifiers or information that may reveal the patient’s identity will not be shared. Source data are provided in this paper.
