# Supplementary material for: IAP antagonist tolinapant (ASTX660) in combination with pembrolizumab in advanced solid tumors: a phase I trial
Source: Nat Commun. 2026 Sep 30;17:11255. doi: 10.1038/s41467-026-77552-7 (PMC13720124; doi:10.1038/s41467-026-77552-7)
Supplement: Supplementary file 1 [file 41467_2026_77552_MOESM1_ESM.pdf]

IAP inhibitor ASTX660 in combination with anti-PD-1 pembrolizumab in advanced solid tumors: a phase I trial

Supplementary Information File

Contents:

Supplementary Tables

Supplementary Table 1. Receptor status and PAM50 classification of breast cancer patient biopsies
Supplementary Table 2. Corticosteroid use for treatment-related adverse events
Supplementary Table 3. List of antibodies and BOND working conditions used for IHC and mIF panels on ASTEROID samples

Supplementary Figures

Supplementary Fig. 1. Dose-toxicity relationship.
Supplementary Fig. 2. Timeline of immune-related adverse events (irAEs)
Supplementary Fig. 3. Changes in cell death marker expression by IHC pre- (C1D1) and post-ASTX660 (C1D7) in responders (R) and non-responders (NR).
Supplementary Fig 4. isPLA controls.
Supplementary Fig. 5. Ki67 expression in tumor tissue assessed by IHC.
Supplementary Fig. 6. Principal component analysis (PCA) of transcriptomic profiles from pre- (C1D1) and post-treatment (C1D7) biopsies of ER+ breast cancer patients treated with ASTX660.
Supplementary Fig. 7. Immune cell profiling and PD-L1 expression in paired biopsies from patient 101-010.
Supplementary Fig. 8. Heat map illustrating levels of tumor-infiltrating lymphocytes (TILs) on H&E stained paired tumor samples.
Supplementary Fig. 9. CA-19-9 tumor marker response in patient with pancreati cancer (102-008)
Supplementary Fig. 10. GSEA of tumor RNA.
Supplementary Fig. 11. Baseline plasma protein levels by treatment response.
Supplementary Fig. 12. Changes in plasma protein concentrations in ASTX660-01 monotherapy trial.
Supplementary Fig. 13. Changes in immune cell populations in the tumor microenvironment (TME).
Supplementary Fig. 14. FUME-TCR sequencing of tumor and PBMC samples.
Supplementary Fig. 15. Association between TCR clonal diversity and the number of unique HLA class I alleles per patient.
Supplementary Fig. 16. Immunopeptidome mutation burden per patient.
Supplementary Fig. 17. Flow cytometry analysis of paired blood samples.
Supplementary Fig. 18. Gating strategies for flow cytometry of PBMCs

Supplementary Methods

1. Supplementary Trial Information

Supplementary Notes

1. ASTEROID Protocol Final Version 4.0
2. Statistical Analysis Plan
3. CONSORT-DEFINE checklist

Supplementary Tables

Supplementary Table 1. Receptor status and PAM50 classification of breast cancer patient biopsies

ID	ER IHC	PR IHC	HER2 IHC (all ISH -)	Both biopsies from same site	C1D1 PAM50	C1D7 PAM50
101-002	ER+ (8/8)	PR- (0/8)	2+	Yes	Luminal A	Luminal B
101-003	ER+ (7/8)	PR+ (4/8)	2+	Yes	Luminal B	Luminal B
101-004	ER+ (7/8)	PR- (0/8)	0	Yes	Luminal B	Luminal B
101-008	ER+	PR+	0	Yes	Luminal A	Luminal A
101-013	ER+ (4/8)	PR- (0/8)	1+	No	Luminal B	Luminal B
101-015	ER+ (7/8)	PR+ (8/8)	1+	Yes	Luminal B	Luminal B
101-016	ER+ (8/8)	PR- (0/8)	2+	Yes	Luminal B	Luminal B
101-017	ER+ (8/8)	PR+ (7/8)	0	No	Normal[a]	Luminal A
101-020	ER+ (8/8)	PR+ (5/8)	2+	Yes	Luminal B	Luminal B
101-010	ER+ (?)	?	0	Yes	(not done)[b]	(not done)[b]
101-018	ER+ (8/8)	PR+ (7/8)		Yes	(not done)[b]	(not done)[b]
101-021	ER-	PR-	0	Yes	(not done)[c]	(not done)[c]

[a]Normal subtyping probably due to low tumor content of biopsy sample (30%)

[b]PAM50 typing was not performed for patients 101-010 and 101-018 due to biopsies having insufficient tumor content for RNA extraction.

[c]PAM50 typing was not performed for patient 101-021 due to samples not reaching our cut-off date for sample processing.

Supplementary Table 2. Corticosteroid use for treatment-related adverse events

ID	Indication	Corticosteroid	Route	Dose and Frequency
101-004	rash	mometasone	topical	0.1% BD for 3 days
	rash	clobetasol	topical	0.05% BD for 2 days
101-007	rash	hydrocortisone	topical	1% BD for 3 days
101-008	rash	mometasone	topical	0.1% OD for 5 days
	oral mucositis	prednisolone	oral	5 mg BD for 8 days
101-010	eczema flare	mometasone	topical	0.1% OD alternate days and PRN for duration of trial
		clobetasol	topical	0.1% BD PRN
	rash	clobetasol	topical	0.05% OD for 28 days
		clotrimazole/ betamethasone	topical	1%/0.064% BD PRN
101-011	arthritis flare (1st episode)	prednisolone	oral	30 mg OD for 4 days
	arthritis flare (2nd episode)	prednisolone	oral	30 mg OD for 2 days
	pneumonitis	methylprednisolone	iv	110 mg (2.5mg/kg) OD for 5 days, then switched to prednisolone
		prednisolone	oral	125 mg OD for 3 days then weaned off over total 45 days
101-012	rash	mometasone	topical	0.1% OD for 17 days
101-016	rash	betamethasone	topical	0.025% BD for duration of trial
101-018	rash	clobetasol	topical	0.05% PRN
		prednisolone	oral	30 mg OD for 8 days then weaned off over total 17 days
	amylase/lipase derangement	prednisolone	oral	10 mg OD for 7 days
102-008	pneumonitis (1st episode)	prednisolone	oral	50 mg OD for 11 days then weaned off over total 33 days
	pneumonitis (2nd episode)	prednisolone	oral	50 mg OD for 7 days then weaned off over total 34 days
	dermatitis	hydrocortisone	topical	PRN

Abbreviations: intravenous (iv); twice a day (BD), once a day (OD), PRN (as needed)

Supplementary Table 3. List of antibodies and BOND working conditions used for IHC and mIF panels on ASTEROID samples

Primaries antibodies (clones)	Host	Vendor/ Catalogue No.	Working dilutions (polymer use)	HIER solution	Positive controls	Negative controls
Cell death marker IHC panel						
RIPK1 (E8S7U)	Rabbit	CST 73271	1:400	ER2	HT29 cells; Normal human colon; Human colorectal cancer	HT29 cells treated with RIPK1 PROTAC degrader
RIPK3 (E7A7F)	Rabbit	CST 10188	1:300	ER2	HT29 cells; Human normal colon	HT29 RIPK3 knockout cells
MLKL (E7V4W)	Mouse	CST 26539	1:800	ER2	HT29 cells; Human colorectal cancer	NCI-H522 cells
Cleaved caspase 3 (Asp175 - 5A1E)	Rabbit	CST 9664	1:300	ER2	Human tonsil; Human colorectal cancer	Human tonsil (no primary antibody control)
Cleaved caspase 8 (D374) (18C8)	Rabbit	CST 9496	1:300	ER2	Human tonsil; Human colorectal cancer	Human tonsil (no primary antibody control)
Ki67 (SP6)	Rabbit	AB16667	1:300	ER2	Human tonsil; Human lymph node	Human tonsil (no primary antibody control)
mIF Panel 1						
CD4 (EPR6855)	Rabbit	AB133616	1:200	ER2	Human tonsil	
CD8 (C8/144B)	Mouse	M7103	1:500	ER2		
FoxP3 (236A/E7)	Mouse	AB20034	1:300	ER1		
Granzyme B (EPR8260)	Rabbit	AB134933	1:250	ER2		
PD1 (EPR4877(2))	Rabbit	AB137132	1:500	ER1		
PanCK (AE1/AE3)	Mouse	M3515	1:200	ER1		
mIF Panel 2						
CD11c (EP1347Y)	Rabbit	AB52632	1:250	ER2	Human tonsil; Human appendix	
CD141 (EPR4051)	Rabbit	AB109189	1:100	ER2		
CD123 (BR4MS)	Mouse	CST23797S	1:300 (with polymer)	ER2		
CD28 (D274E)	Rabbit	CST38774	1:200	ER2		
CD86 (E2G8P)	Rabbit	CST91882	1:400 (with polymer)	ER2		
PanCK (AE1/AE3)	Mouse	Dako M3515	1:200	ER1		
mIF Panel 3						
CD68 (PG-M1)	Mouse	Dako M0814	1:150	ER2	Human tonsil	
CD206 (E2L9N)	Rabbit	CST91992	1:300	ER1		
CCR2 (SN707)	Rabbit	ThermoFisher MA5-41175	1:500	ER2		
CD31 (JC70A)	Mouse	Dako M0823	1:250	ER2		
CD20 (L26)	Mouse	AB9475	1:250	ER1		
PanCK (AE1/AE3)	Mouse	Dako M3515	1:200	ER1		
Other IHC						
cIAP1 (EPR4673)	Rabbit	AB108361	1:200	ER1	Human appendix; MDA-MB-231 cells	MDA-MB-231 cells treated with SM164
PD-L1 (E1L3N)	Rabbit	CST 13684	1:250	ER2	Human Placenta; LNCaP cells	Human Ovary; LNCaP cells treated with siRNA against PD-L1

Supplementary Figures

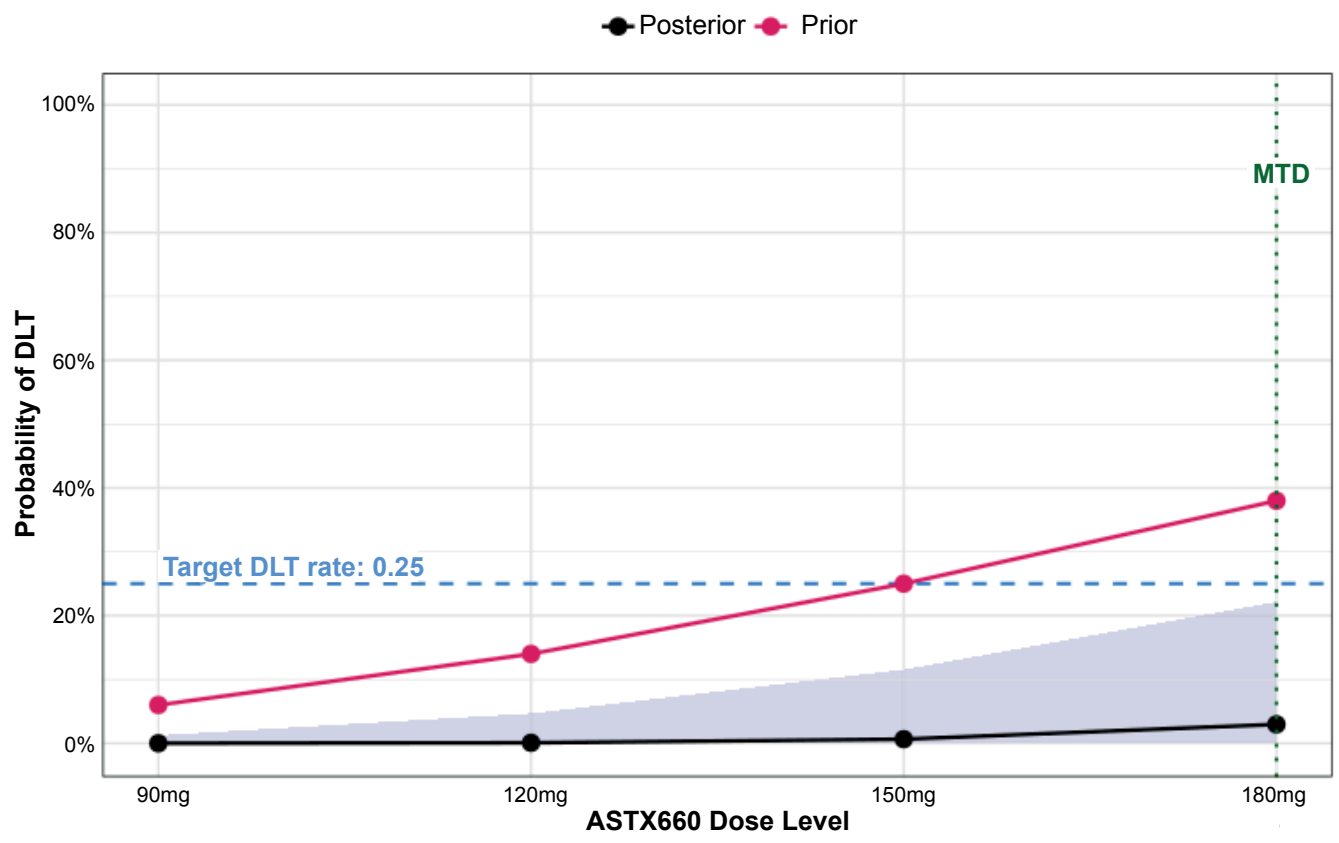

Supplementary Fig. 1. Dose-toxicity relationship. a, Posterior DLT probability estimates with 90% credible intervals (CrI). The dashed horizontal line indicates the target DLT rate, and the dotted vertical line marks the estimated MTD. Source data is provided as a Source Data file.

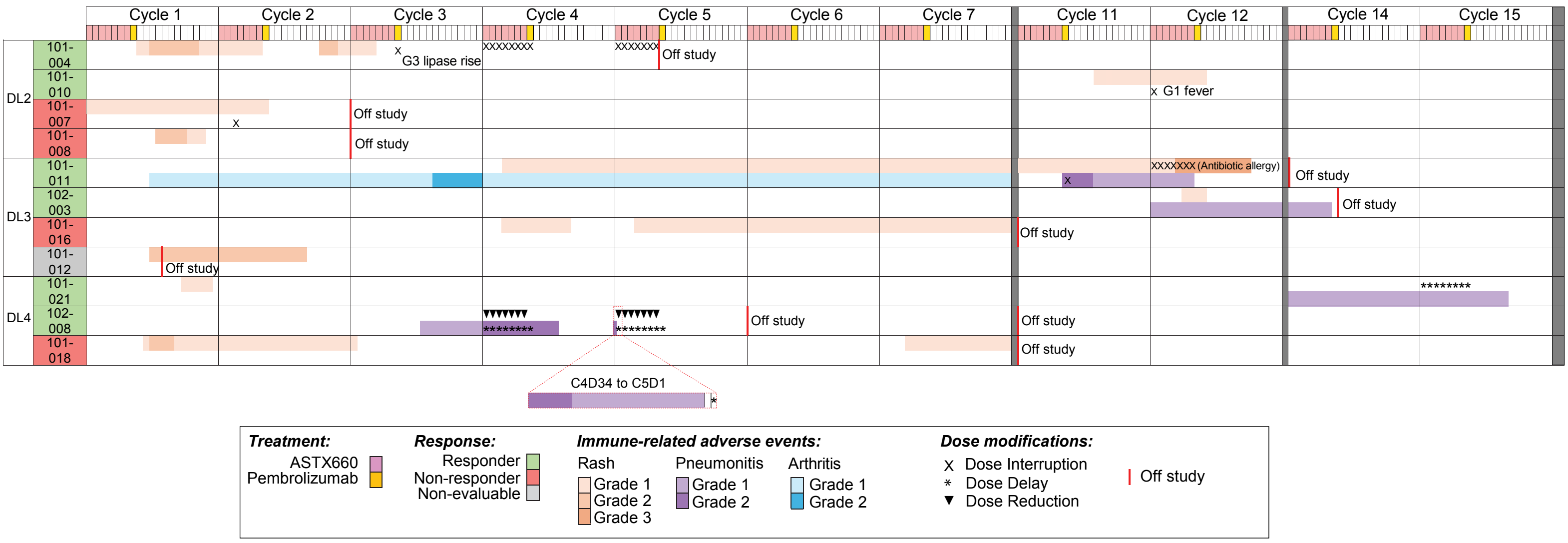

Supplementary Fig. 2. Timeline of immune-related adverse events (irAEs). 11 patients at dose levels (DL) 2-4 experienced immune-related rash, pneumonitis and arthritis. Colored bars indicate incidence of irAEs coded according to type and severity. Dose modifications are labeled. Patient 101-021 developed pneumonitis leading to Cycle 15 being delayed for 31 days. Patient 102-008 had two episodes of pneumonitis, requiring two dose reductions (from DL4 to DL2) with delay in Cycle 4 for 32 days and delay in Cycle 5 for 45 days.

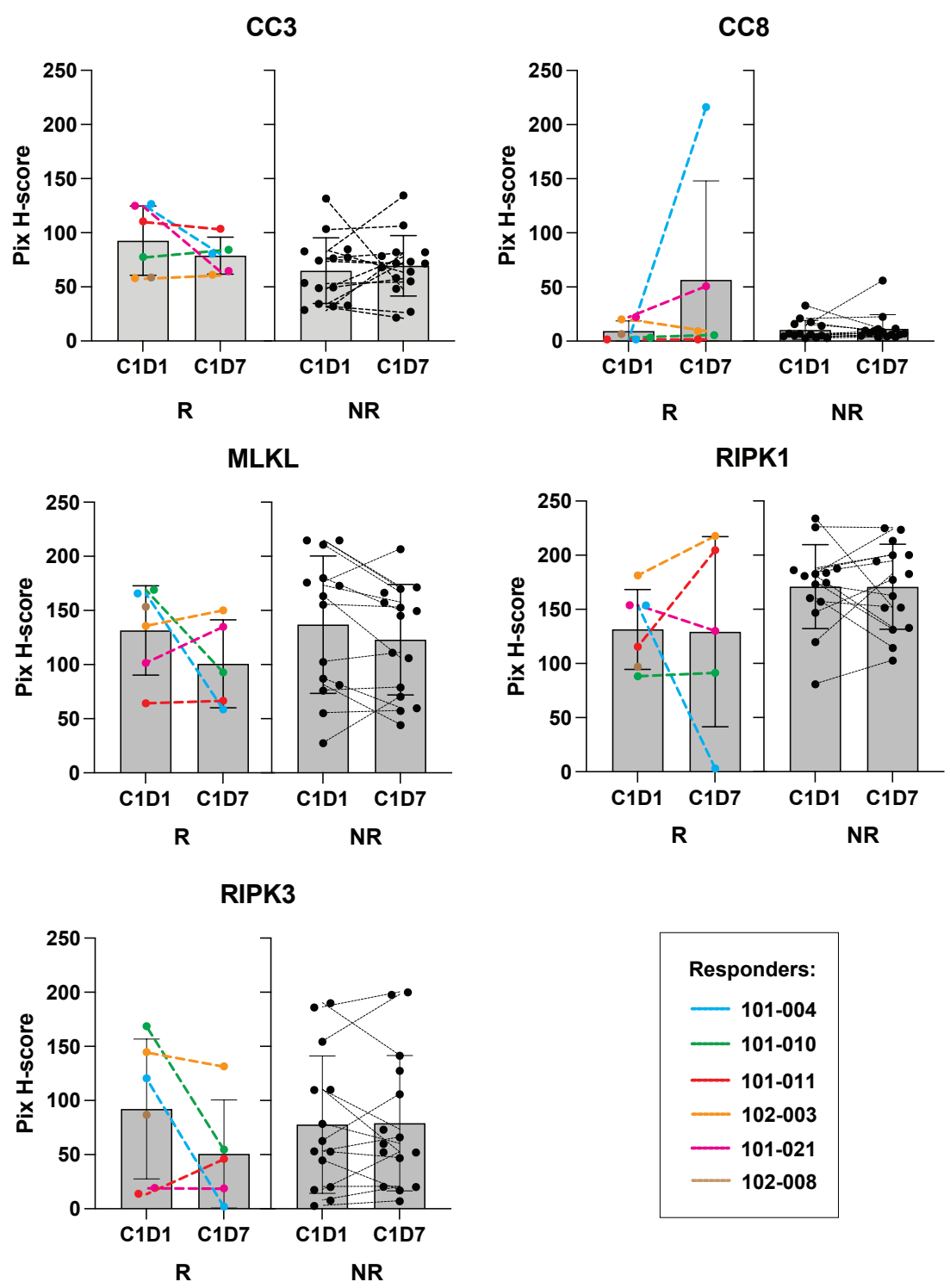

Supplementary Fig. 3. Changes in cell death marker expression by IHC pre- (C1D1) and post-ASTX660 (C1D7) in responders (R) and non-responders (NR). Source data is provided as a Source Data file.

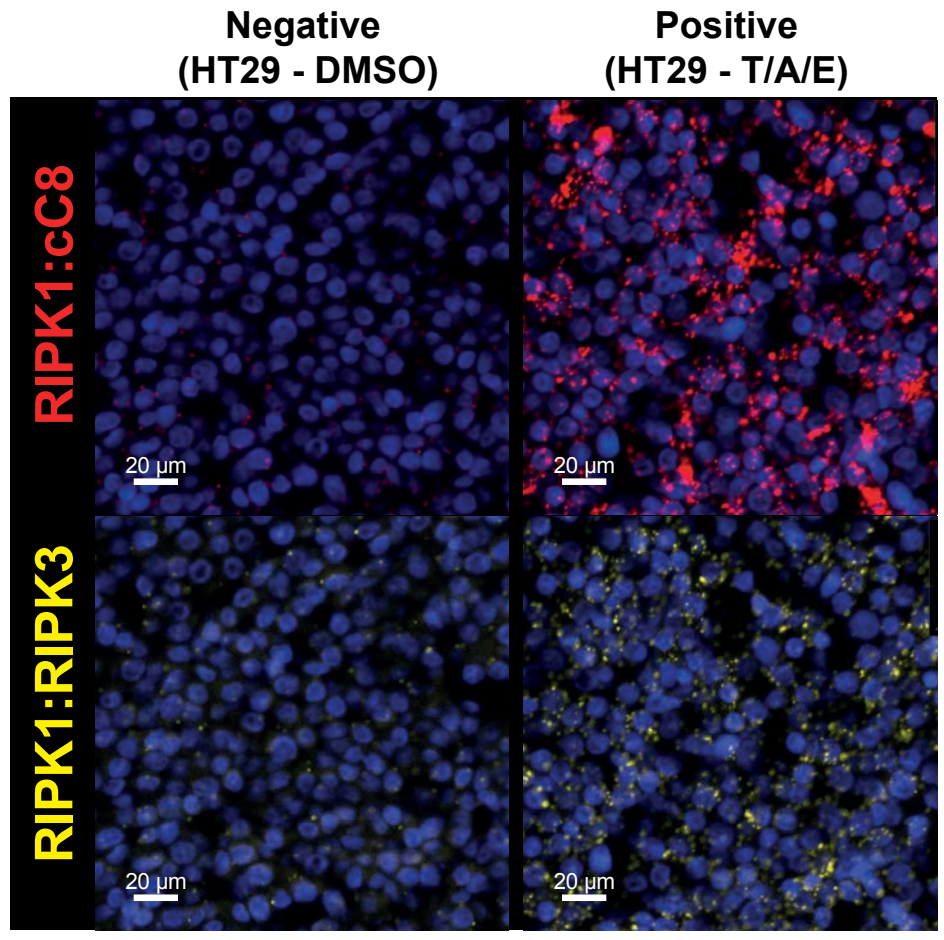

Supplementary Fig. 4. isPLA controls. HT29 cells treated with DMSO or the combination of TNF, ASTX660 and Emricasan (TSE) were used as negative and positive controls, respectively, to validate the in situ proximity ligation assay (isPLA). (n=1).

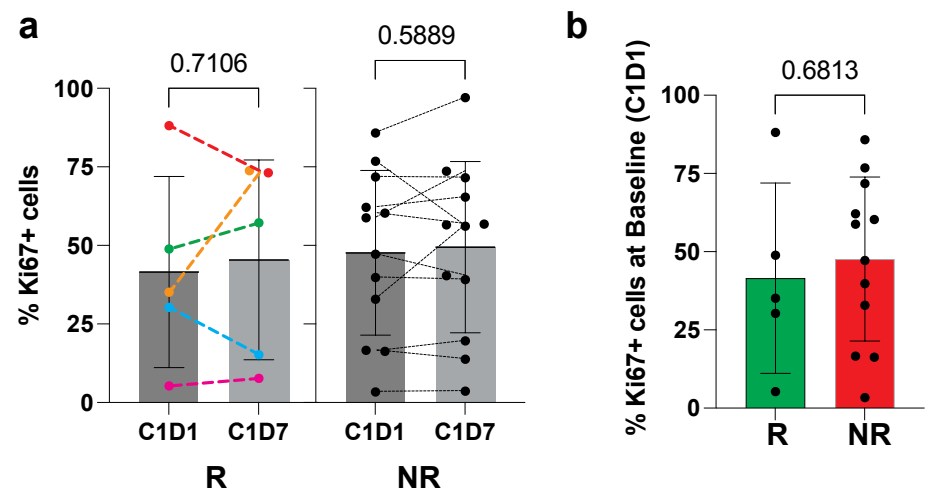

Supplementary Fig. 5. Ki67 expression in tumor tissue assessed by IHC. **a,** Change in percentage of Ki67+ tumor cells pre- and post-ASTX660 treatment in responders (R) and non-responders (NR). **b,** Baseline % of Ki67+ tumor cells comparing R vs NR. p-values were calculated using two-tailed paired and unpaired t-tests, for (a) and (b), respectively. Source data is provided as a Source Data file.

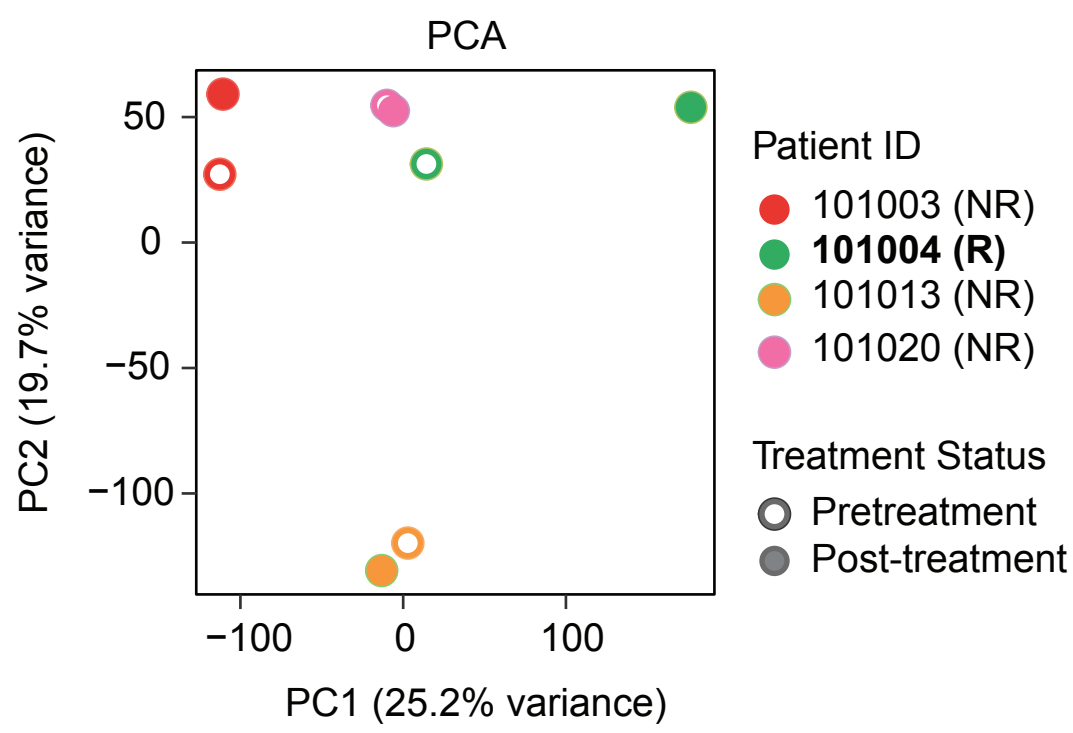

Supplementary Fig. 6. Principal component analysis (PCA) of transcriptomic profiles from pre- (C1D1) and post-treatment (C1D7) biopsies of ER+ breast cancer patients treated with ASTX660. Analyses were restricted to samples with tumor content >85% to enable accurate clustering by minimizing confounding effects of tumor purity. R, responders; NR, non-responders. Source data is provided as a Source Data file.

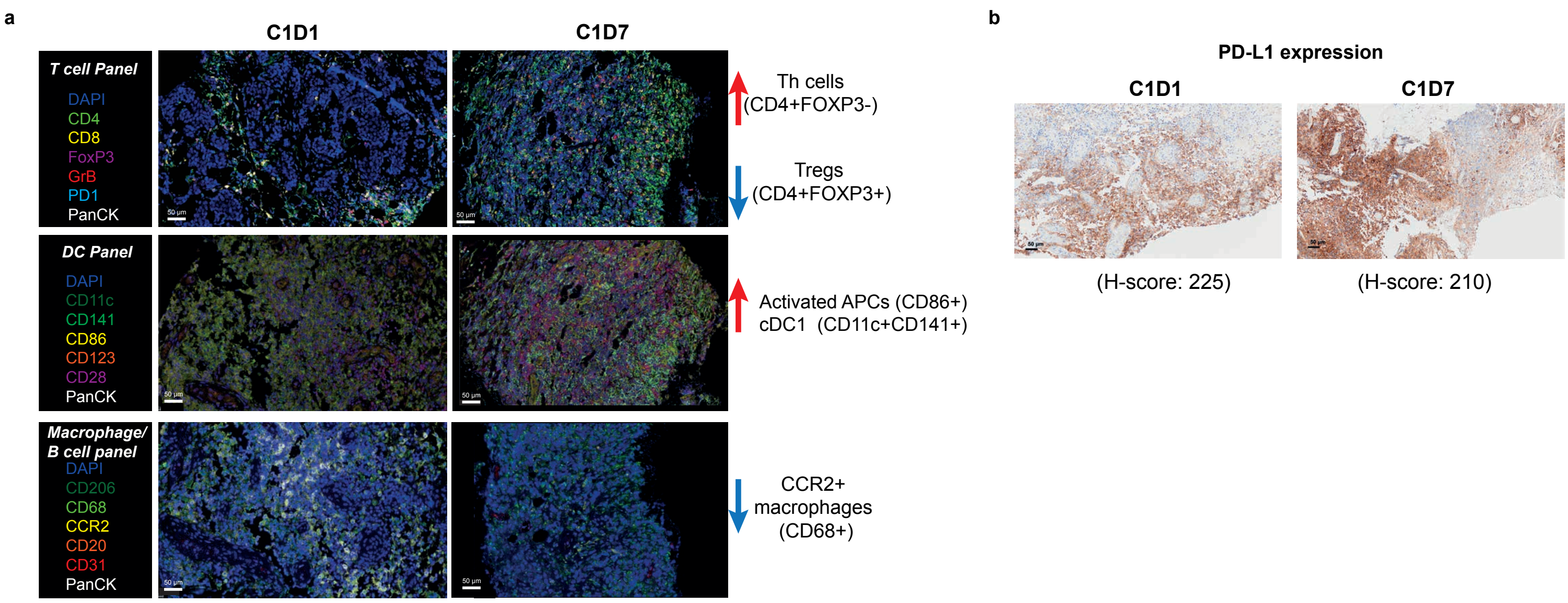

Supplementary Fig. 7. Immune cell profiling and PD-L1 expression in paired biopsies from patient 101-010. a, Multiplex immunofluorescence (mIF) images showing changes in immune cell populations following ASTX660 treatment. Helper T cell (Th, CD4+FOXP3-), regulatory T cell (Treg, CD4+FOXP3+), activated antigen presenting cells (APC, CD86 +), classical dendritic cell type 1 (cDC1, CD11c+CD141+), and CCR2+ macrophages (CCR2+CD68+). **b,** Images of pre- (C1D1) and post- (C1D7) ASTX660 biopsies showing PD-L1 expression by IHC. H-scores are indicated. (n=1).

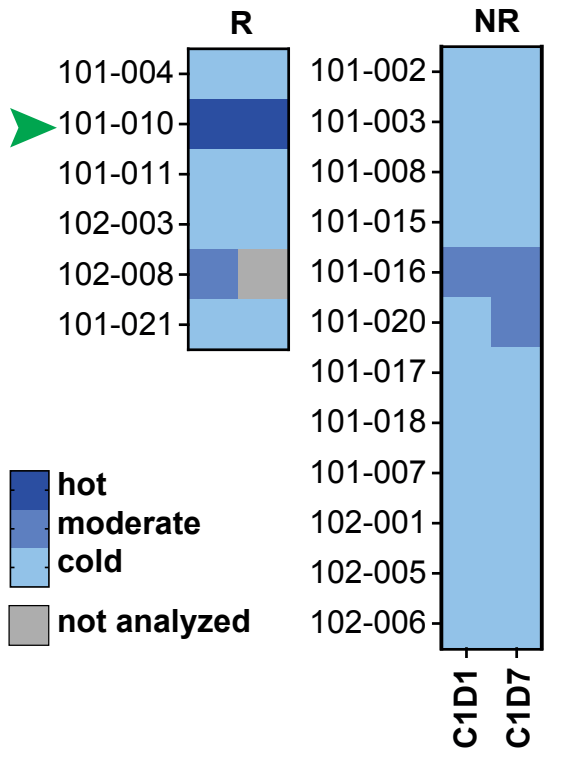

Supplementary Fig. 8. Heat map illustrating levels of tumor-infiltrating lymphocytes (TILs) on H&E stained paired tumor samples. Samples are classifed as hot (TILs >50% of tumor area), moderate (10-50%), or cold (0-10%), according to pathologist's visual assessment. Source data is provided as a Source Data file.

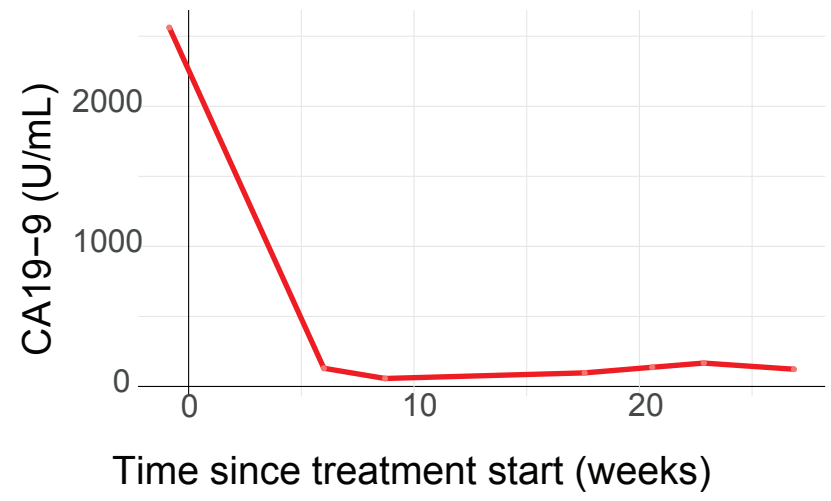

Supplementary Fig. 9. CA19-9 tumor marker response in patient with pancreatic cancer (102-008). Source data is provided as a Source data file.

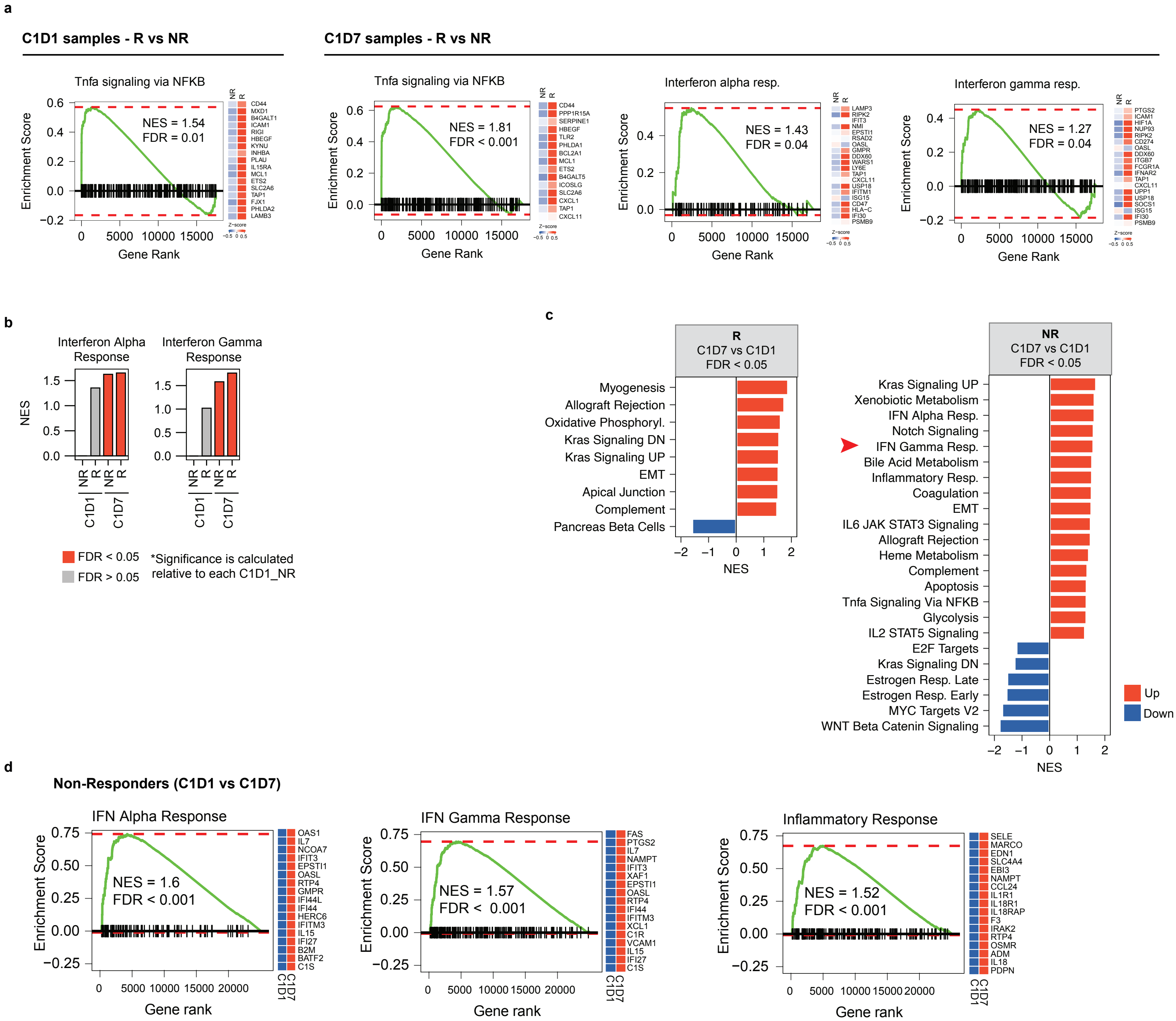

Supplementary Fig. 10. GSEA of tumor RNA. a, Hallmark gene sets that were significantly upregulated [false discovery rate (FDR) <0.05] in responders versus non-responders at baseline (C1D1) and on-treatment (C1D7); (see Fig. 4b). The top 17 differentially expressed genes for each gene set are listed. FDR, false discovery rate; NES, normalized enrichment score. **b,** Normalized enrichment scores (NES) for selected MSigDB Hallmark gene sets across responders (R) and non-responders (NR) before (C1D1) and after (C1D7) ASTX660 treatment, relative to C1D1 non-responders. **c, d,** GSEA of the MSigDB Hallmark collection showing pathways differentially enriched between C1D7 and C1D1 samples. Results are shown independently for responders and non-responders. Top 17 differentially expressed genes for each gene set are listed. See Suppl. Data 2e-f for detailed data.

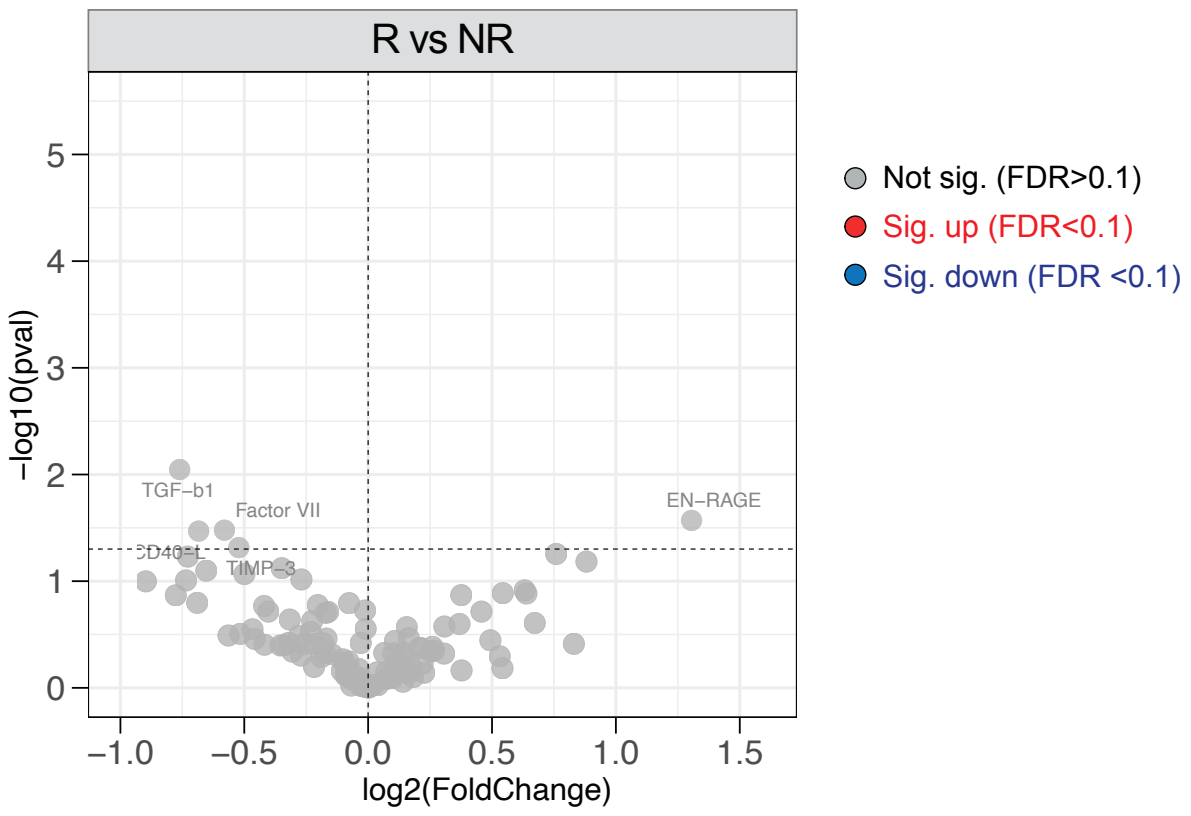

Supplementary Fig. 11. Baseline plasma protein levels by treatment response. Volcano plot of log2 of baseline (C1D1) plasma protein concentrations from responders versus non-responders, plotted against -log10 (p-value). There were no significant differences between both groups, with FDR <0.1 considered significant. Source data is provided as a Source Data file.

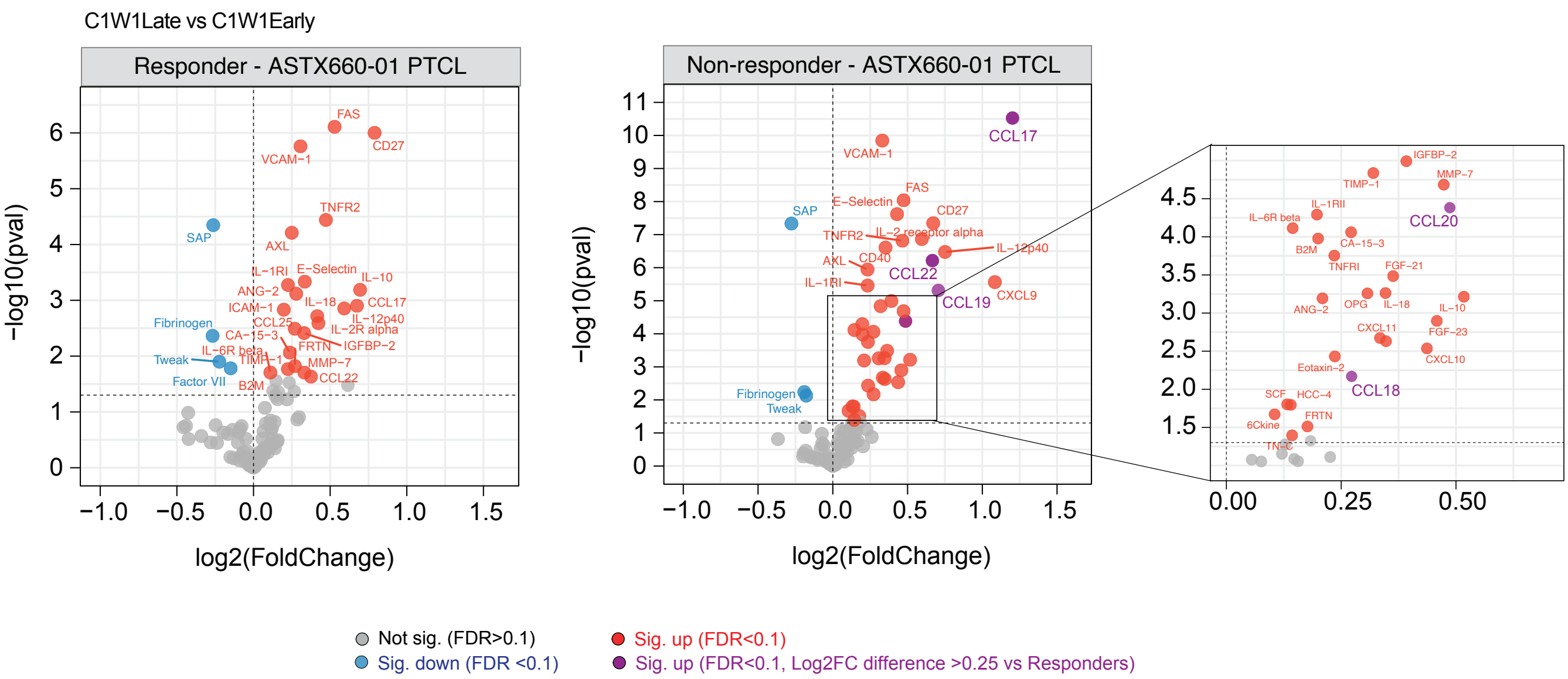

Supplementary Fig. 12. Changes in plasma protein concentrations in ASTX660-01 monotherapy trial. Volcano plots of log2 fold change (log2FC) of plasma protein concentrations taken at "late" (C1W1Late) over "early" (C1W1Early) timepoints, against -log10 (p-value), with FDR (C1W1Late-C1W1Early) <0.1 as significant. (See Methods for "early" and "late" timepoints criteria and statistical analysis). Consistent with the ASTEROID dataset, CCL17, CCL18, CCL19, CCL20 and CCL22 were significantly upregulated in non-responders compared to responders [FDR (C1W1Late-C1W1Early) <0.1 and log2FC difference (NR-R) >0.25]. Source data is provided as a Source Data file.

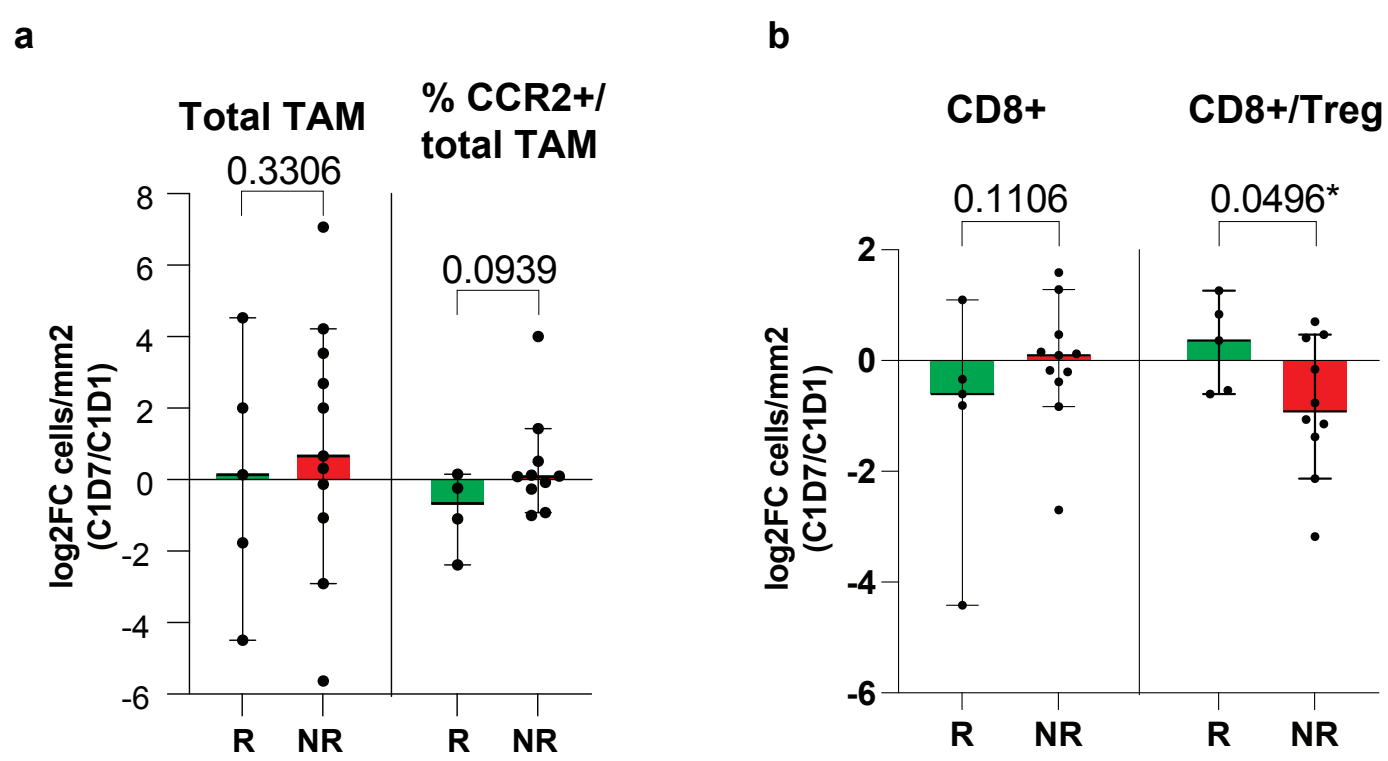

Supplementary Fig. 13. Changes in immune cell populations in the tumor microenvironment (TME). a, Log2FC of TAMs and percentage of CCR2+ TAMs from C1D1 to C1D7. **b,** CD8+ T-cells/mm2 and CD8+/Treg ratio from C1D1 to C1D7. p-values were calculated using one-tailed Mann-Whitney test, with <0.05 as significant. Error bars show median ± 95% CI. For total TAM and CD8+ calculations, responders (R, n=5) and non-responders (NR, n=11) were analyzed; for %CCR2/total TAM, R (n=4) and NR (n=10); for CD8+/Treg, R (n=5) and NR (n=10). Source data is provided as a Source data file.

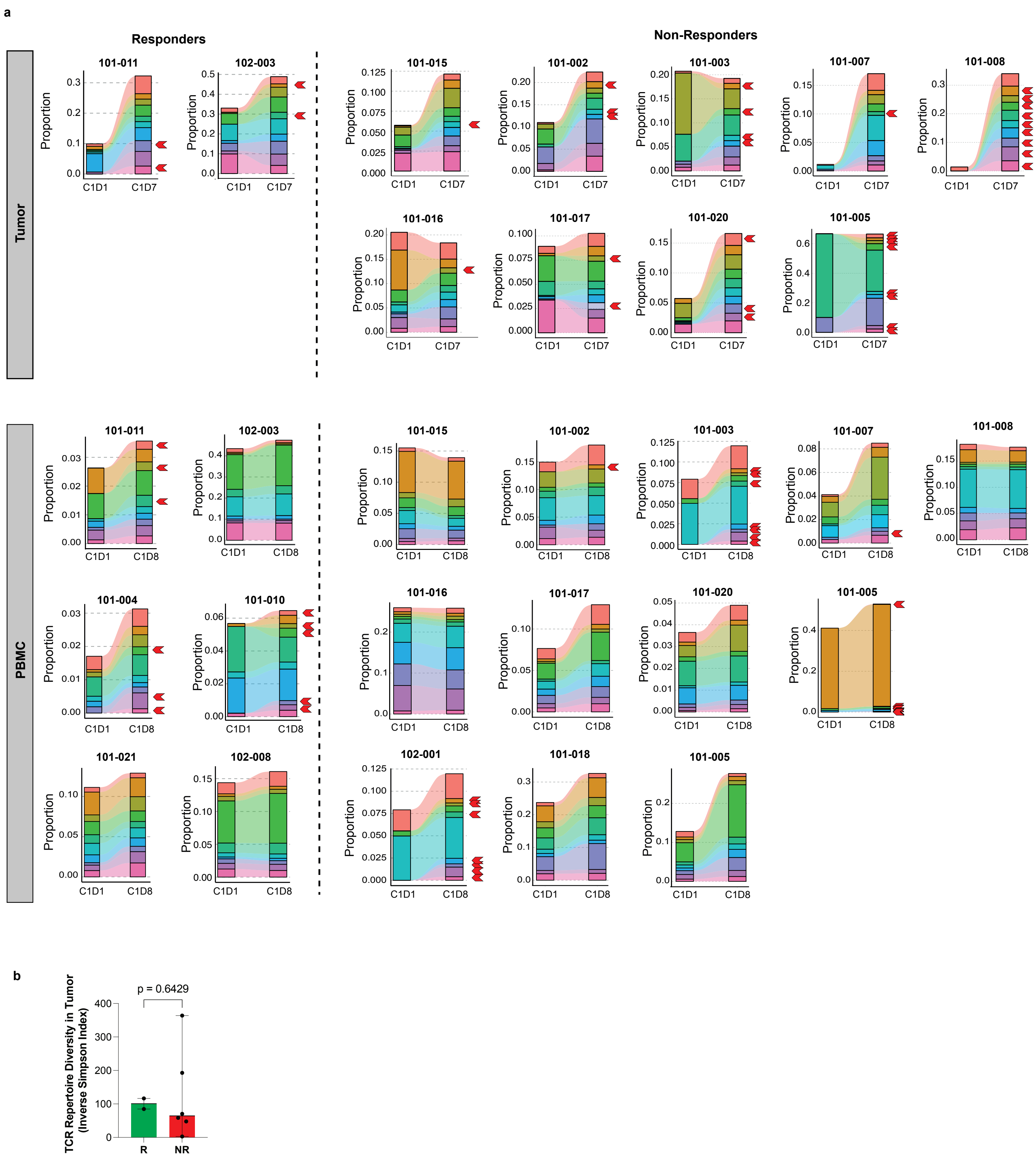

Supplementary Fig. 14. FUME-TCR sequencing of tumor and PBMC samples. a, TCR clonotype tracking in tumor and PBMC samples. Frequencies of the then most common clonotypes detected at C1D7 samples were tracked to C1D1. Red arrows indicate emergent clonotypes absent at C1D1. **b,** Baseline (C1D1 tumor sample) TCR clonal repertoire diversity, measured by inverse Simpson index. Two germline BRCA2 mutation carriers and one patient with PMS2 mutation carrier (Lynch syndrome) were excluded. R (n=2), NR (n=6); p-value calculated using two-tailed Mann-Whitney test; error bars represent median ± 95% CI. Source data is provided as a Source data file.

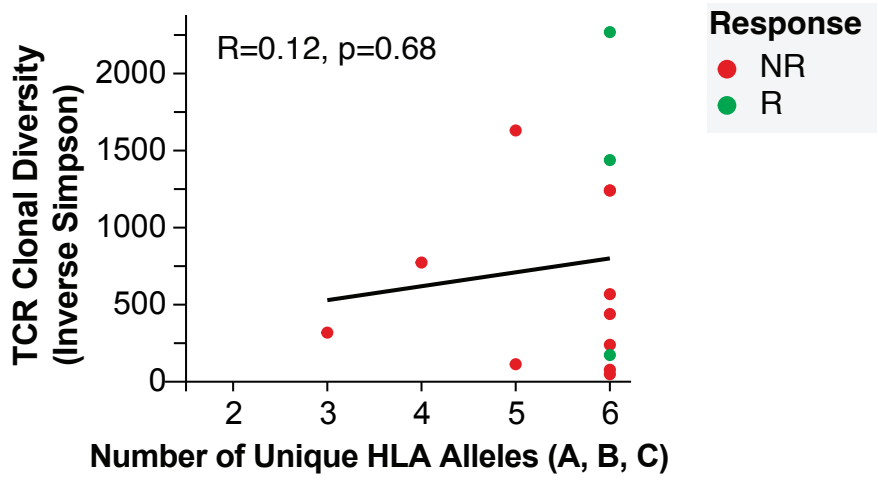

Supplementary Fig. 15. Association between TCR clonal diversity and the number of unique HLA class I alleles per patient. TCR clonal diversity was measured by inverse Simpson index. The total number of unique HLA-A, -B, and -C alleles (range: 1–6) per patient was determined using Optitype. Each point represents an individual from the ASTEROID trial. Responders (R, n = 3) carried the maximum number of HLA alleles (6); one responder displayed low TCR diversity (~200), whereas two others exhibited high diversity (~1500 and >2000). Spearman correlation R = 0.12, p=0.68. Source data is provided as a Source data file.

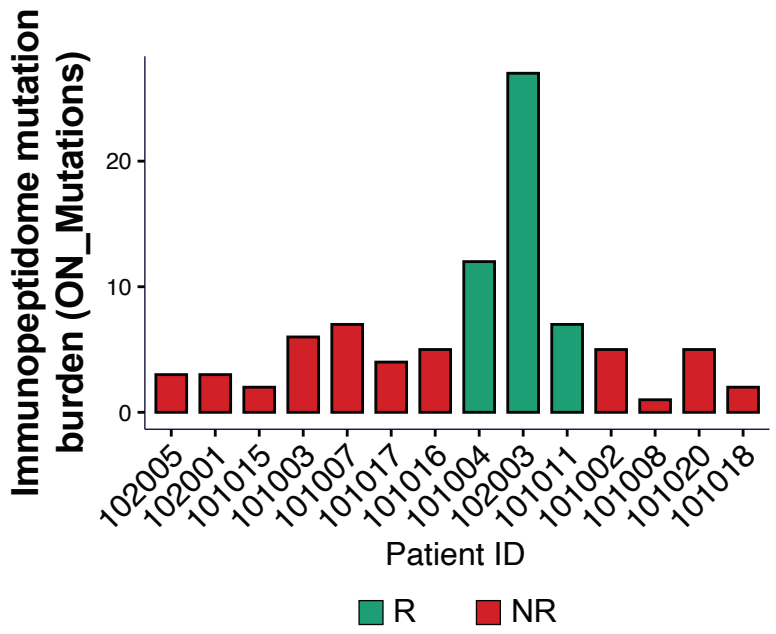

Supplemetary Fig. 16. Immunopeptidome mutation burden per patient. Bars represent number of ON_Mutations and are coloured by response group (NR or R). Source data is provided as a Source data file.

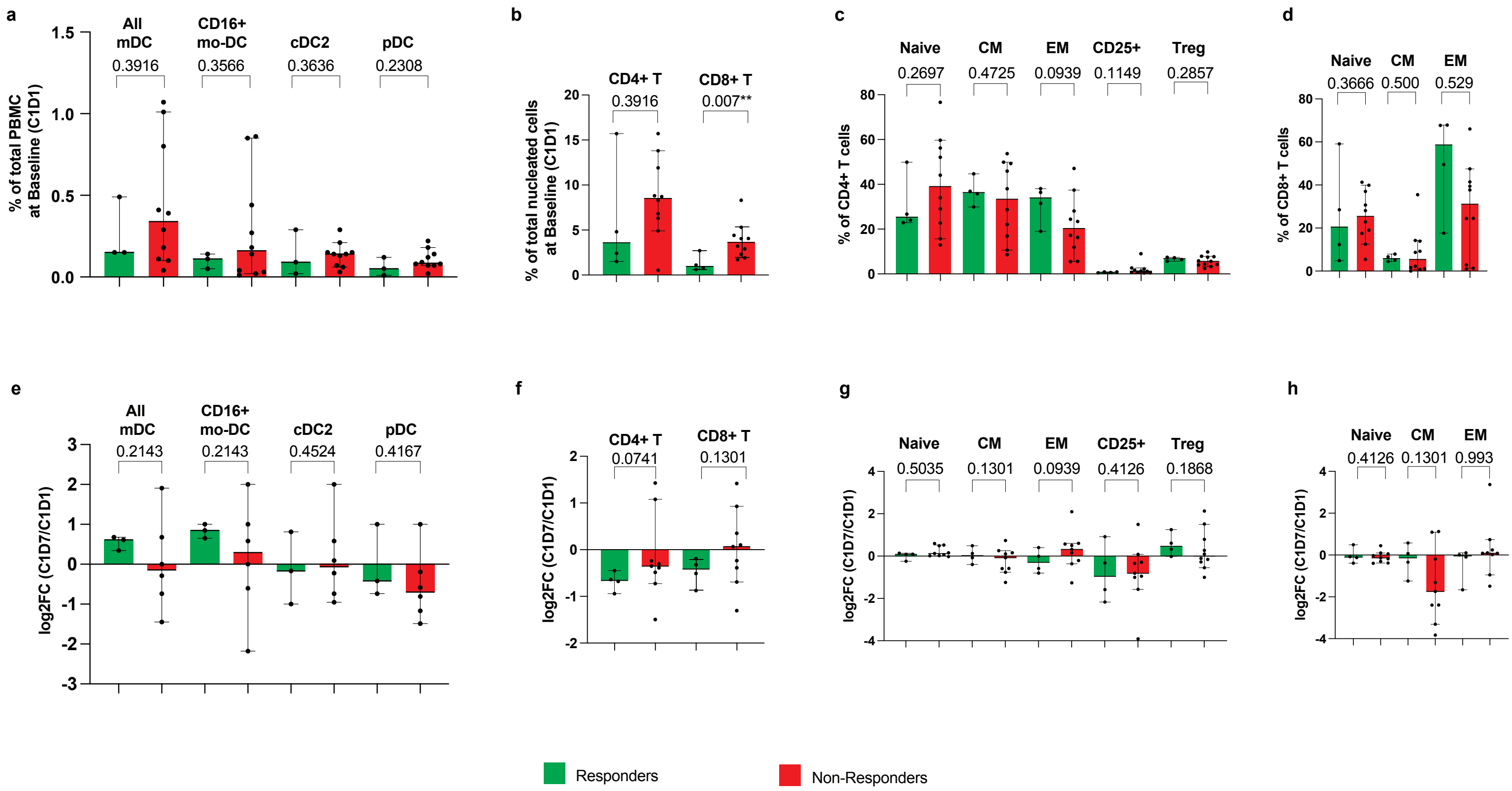

Supplementary Fig. 17. Flow cytometry analysis of paired blood samples. Baseline (C1D1) levels of **(a)** dendritic cells (DC), and **(b)** CD4+ and CD8+ T-cells (as % of total PBMC). Baseline levels of **(c)** CD4+ and **(d)** CD8+ T-cell subpopulations. Log2FC from C1D1 to C1D7 of **(e)** DCs, **(f)** CD4+ and CD8+ T-cells, **(g)** CD4+ subpopulations and (h) CD8+ subpopulations. Statistical comparisons between responders (green) and non-responders (red) were calculated using one-tailed Mann-Whitney test; error bars show median ± 95% CI. For (a), Responders (R, n=3) and Non-responders (NR, n=10) were analyzed; for (b, c, d), R (n=4), NR (n=10); for (e), R (n=3), NR (n=6); for (f, g, h), R (n=4), NR (n=9). DC markers: myeloid DC (mDC, HLA-DR+CD11c+); CD16+ monocytic DC (mo-DC, HLA-DR+CD11c+CD16+); classical DC type 2 (cDC2, HLA-DR+CD11c+CD1c+); plasmacytoid DC (pDC, HLA-DR+CD11c-CD123+). T-cell markers: Naive (CD45RA+CCR7+); central memory (CM, CD45RA-CCR7+); effector memory (EM, CD45RA-CCR7-); regulatory T-cell (Treg, CD4+CD25+CD127low/-); activated CD25+ T-cell (CD4+CD25+). See Supplementary Table 3 for gating strategies. Source data is provided as a Source data file.

a **T cell panel**

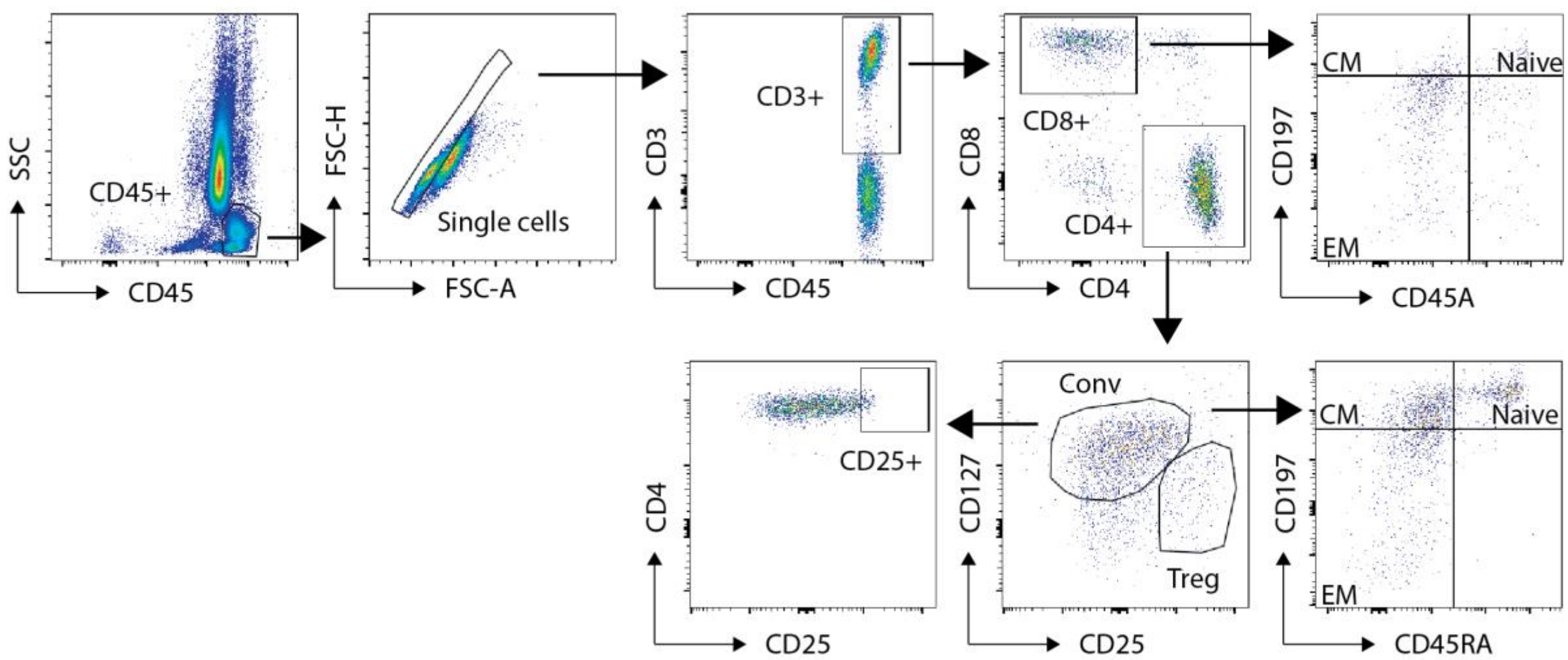

b **Dendritic cell panel**

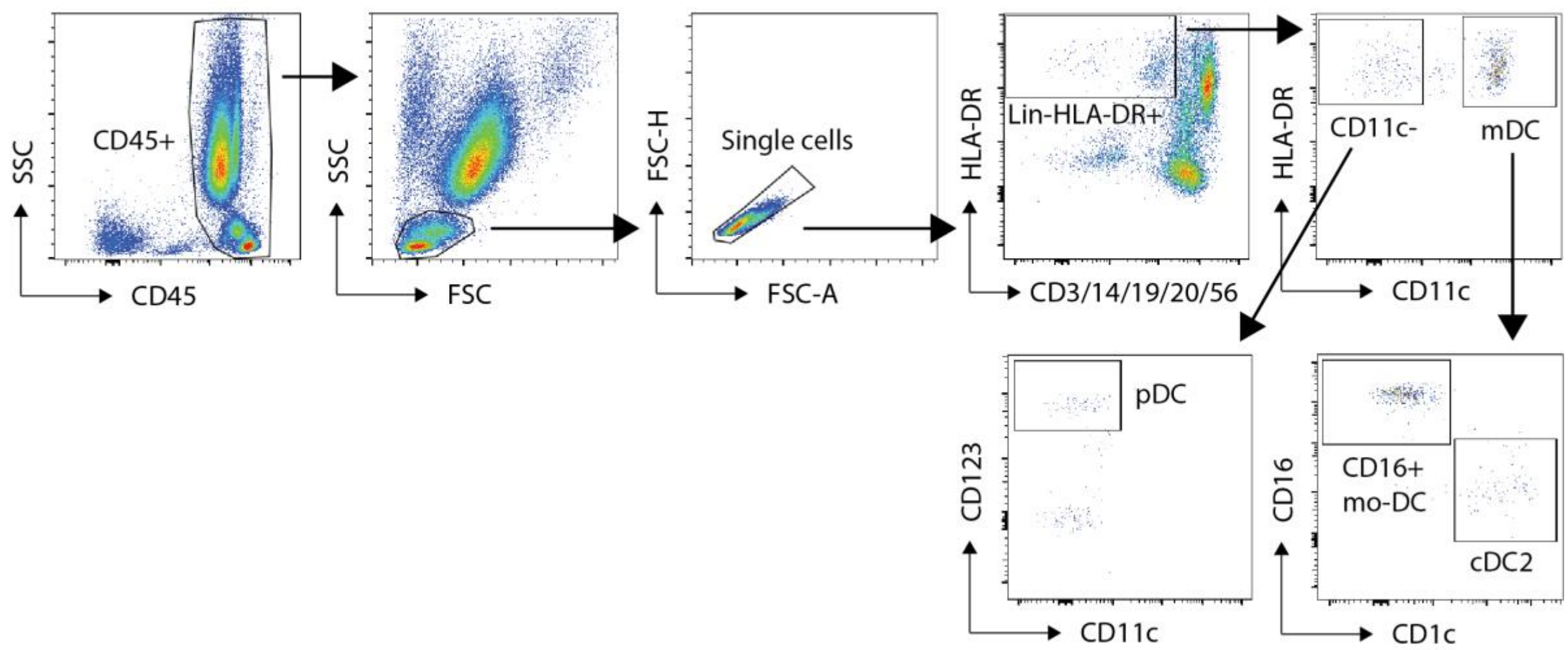

Supplementary Fig. 18. Gating strategies for flow cytometry of PBMCs. a, T cell gating strategy corresponding to Supp. Figures 17b-d and 17f-h**;** **b,** Dendritic cell gating strategy corresponding to Supp. Figures 17a and 17e.

SUPPLEMENTARY METHODS

1. Supplementary Trial Information

Full Title:

ASTEROID: A Phase I trial of tolinapant (ASTX660) in combination with Pembrolizumab in patients with solid tumours: utilizing triple IAP blockade as a strategy to maximize Immunogenic cell Death and the generation of an efficient adaptive immune response

Short title:

ASTEROID: Phase I trial of ASTX660 in combination with Pembrolizumab in solid tumours

Registration:

ClinicalTrials.gov NCT05082259

Patient Population

Up to 18 evaluable patients with advanced solid tumours were planned to be enrolled onto the dose escalation part of the study (Part A). Four dose levels of ASTX660 were explored. The dose escalation utilized a flexible 2 stage Time to event Continual Reassessment Method (TITE-CRM) including time-to-toxicity analysis to account for dose-limiting toxicities that may arise outside the DLT period. The aim of the escalation phase was to obtain the MTD of ASTX660 in combination with a fixed dose of Pembrolizumab associated with a dose limiting toxicity (DLT) set at a target of 25%. A target sample size of 18 evaluable patients was recruited to Part A to enable adequate collection of paired evaluable tumour and blood samples for pharmacodynamic analyses, and this target sample size was anticipated to be sufficient to determine the MTD as per the TITE-CRM design. Once the RP2D had been established, patients will be recruited to the expansion cohort, Part B.

Eligibility Criteria for Part A:

Inclusion criteria:

1. Patients with histologically or cytologically confirmed malignant advanced solid tumours, refractory to conventional treatment, or for which no conventional therapy exists or is declined by the patient.
2. Measurable disease as assessed by imRECIST.
3. All patients with advanced solid tumours enrolled onto Part A must be willing and able to have fresh paired tissue biopsies for biomarker analysis.
4. Life expectancy of at least 12 weeks.
5. World Health Organisation (WHO) performance status of 0 or 1.

6. Haematological and biochemical indices within the ranges shown below. These measurements must be performed within one week (Day -7 to Day 1) prior to the patient’s first dose of IMP.

**Laboratory Test**	**Value required**
Haemoglobin (Hb)	≥ 9.0 g/dL
Absolute neutrophil count	≥ 1.5 x 109/L
Lymphocyte count	>0.5 x 109/L
Platelet count	≥ 100 x 109/L
Total Serum bilirubin	≤ 1.5 x upper limit of normal (ULN)
Alanine aminotransferase (ALT)	≤ 2.5 x (ULN) unless raised due to known metastatic liver disease in which case ≤ 5 x ULN is permissible
Aspartate aminotransferase (AST)	≤ 2.5 x (ULN) unless raised due to known metastatic liver disease in which case ≤ 5 x ULN is permissible
Either:<br>Calculated creatinine clearance<br>Or:<br>Creatinine	≥ 50 mL/min (uncorrected value)<br>Or<br>< 1.5 x upper limit of normal (ULN)
Albumin	>28 g/L
LDH	<3 x ULN
Amylase	≤ ULN
Lipase	≤ ULN
Coagulation	INR <1.5<br>APTT <1.5 x ULN

7. 18 years or over.
8. Written (signed and dated) informed consent and be capable of co-operating with treatment and follow-up.
9. Female patients with reproductive potential must have a negative urine or serum pregnancy test performed within 72 hours of first dose.

Exclusion criteria:

1. Radiotherapy (except for palliative reasons), endocrine therapy, immunotherapy including Pembrolizumab or chemotherapy during the previous four weeks (six weeks for nitrosoureas, Mitomycin-C) and four weeks for investigational medicinal products, except for hormonal therapy with luteinizing hormone-releasing hormone (LHRH) analogues for medical castration in patients with castrate resistant prostate cancer or ovarian suppression in pre- or peri-menopausal women with endocrine-driven breast cancer, which are permitted, and bisphosphonates or RANK ligand antagonists that are permitted for the management of bone metastases.

2. Current malignancies of other types, with the exception of adequately treated cone biopsied in situ carcinoma of the cervix uteri and basal or squamous cell carcinoma of the skin. Cancer survivors, who have undergone potentially curative therapy for a prior malignancy, have no evidence of that disease for five years or more and are deemed at negligible risk for recurrence, are eligible for the trial.
3. Ongoing Grade 2 or greater toxicities from pre-existing conditions or previous treatments. Exceptions to this are alopecia and ongoing anticoagulation therapy due to prior thromboembolic episodes.
4. Ability to become pregnant (or already pregnant or lactating). However, those female patients who have a negative serum or urine pregnancy test before enrolment and agree to use two highly effective forms of contraception (oral, injected or implanted hormonal contraception and condom, have an intra-uterine device and condom, diaphragm with spermicidal gel and condom) for four weeks before entering the trial, during the trial and for six months afterwards are considered eligible. NB. Abstinence is only considered to be an acceptable method of contraception when this is in line with the preferred and usual lifestyle of the subject. Periodic abstinence (e.g., calendar, ovulation, symptothermal, post-ovulation methods) and withdrawal are not acceptable methods of contraception
5. Male patients with partners of child-bearing potential (unless they agree to take measures not to father children by using one form of highly effective contraception [condom plus spermicide] and not to donate sperm during the trial and for six months afterwards). Men with pregnant or lactating partners should be advised to use barrier method contraception (for example, condom plus spermicidal gel) to prevent exposure to the foetus or neonate.
6. Known untreated or active central nervous system (CNS) metastases (progressing or requiring corticosteroids for symptomatic control). Patients with a history of treated CNS metastases are eligible, provided they meet all of the following criteria:
    - Evaluable or measurable disease outside the CNS is present.
    - Radiographic demonstration of improvement or stability upon the completion of CNS-directed therapy at least 4 weeks after completion of CNS directed therapy and no evidence of interim progression between the completion of CNS-directed therapy and the baseline disease assessment.
    - Not requiring corticosteroids.
7. Major surgery within four weeks of the first dose of study treatment.
8. History of malabsorption syndrome or other condition that would interfere with enteral absorption.
9. At high medical risk because of non-malignant systemic disease including active uncontrolled infection.
10. History of (non-infectious) pneumonitis/interstitial lung disease that required steroids, or current pneumonitis/interstitial lung disease.

11. Known to be serologically positive for hepatitis B, hepatitis C or human immunodeficiency virus (HIV).
12. Has an active autoimmune disease that has required systemic treatment in past 3 months (i.e. with use of disease modifying agents, corticosteroids or immunosuppressive drugs). Replacement therapy (e.g., thyroxine, insulin, or physiologic corticosteroid replacement therapy for adrenal or pituitary insufficiency, etc.) is not considered a form of systemic treatment. Patients with Sjogren's syndrome will not be excluded from the study. In addition, patients that experienced a Grade 3 or higher immune-related AE on treatment with immunotherapy will be excluded from the study. Patients that have experience a prior G2 immune-related AE on treatment will need case-by-case discussion with the CI. Patients with inactive autoimmune disease which has previously required systemic therapy, may be considered on a case-by-case basis after discussion with the sponsor.
13. Has a diagnosis of immunodeficiency or is receiving systemic steroid therapy or any other form of immunosuppressive therapy within 14 days prior to the first dose of trial treatment. The use of physiologic doses of corticosteroids may be approved after consultation with the chief Investigator. Stable use (i.e., no change in dose within 1 month prior to Day 1 of Cycle 1) of inhaled corticosteroids is allowed. Patients for cohort B4 who are on a stable dose of corticosteroids not exceeding 2mg Dexamethasone for tumour associated oedema or symptoms will be permitted to enrol on the study.
14. Has received a live vaccine within 30 days of planned start of study therapy. Note: The killed virus vaccines used for seasonal influenza vaccines for injection are allowed; however intranasal influenza vaccines (e.g. FluMist®) are live attenuated vaccines and are not allowed.
15. Any of the following cardiac criteria:
    a. Mean resting corrected QT interval (QTcF) > 470 msec obtained from an electrocardiogram (ECG). Known congenital QT syndrome or history of torsades de pointes.
    b. Left ventricular ejection fraction of <50% on echocardiogram.
    c. Any clinically significant abnormalities in rhythm, conduction or morphology of resting ECG, e.g. complete left bundle branch block, third degree heart block. Controlled atrial fibrillation is allowed.
    d. Experience of any of the following procedures or conditions in the preceding 6 months: coronary artery bypass graft, angioplasty, vascular stent, myocardial infarction, angina pectoris, congestive heart failure New York Heart Association [NYHA Grade 2 or above], severe valvular disease, uncontrolled hypertension despite optimal therapy.
16. Prior bone marrow transplant or have had extensive radiotherapy to greater than 25% of bone marrow within eight weeks.

17. Participates or plans to participate in another interventional clinical trial, whilst taking part in this Phase I study of ASTX660 and Pembrolizumab. Participation in an observational trial would be acceptable.
18. Patients with prior exposure to an IAP antagonist (Smac mimetic) will be excluded from this study. Patients with prior exposure to immunotherapy (either CTLA-4, PD-1/PD-L1 inhibitor/cellular therapy) will be permitted to enrol as long as they did not experience any immune-adverse event toxicity while on their prior immunotherapy as described in exclusion criteria 12.
19. History or current evidence of any condition, therapy, or laboratory abnormality that might confound the results of the study, interfere with the patient's participation for the full duration of the study, or is not in the best interest of the patient to participate, in the Investigator's opinion.
20. Severe hypersensitivity (≥ Grade 3) to any of the IMPs and/or any of their excipients.
21. Known psychiatric or substance abuse disorders that would interfere with cooperation with the requirements of the trial.
22. History of an allogenic tissue/ solid organ transplant.
23. Symptoms of COVID-19 and/or documented COVID-19 infection.

Definition of dose limiting toxicity

The dose limiting toxicity (DLT) and maximum tolerated dose (MTD) are defined using the National Cancer Institute (NCI) Common Terminology Criteria for Adverse Events (CTCAE) Version 5.0. DLTs will be primarily assessed during the 6 week period consisting of Cycle 1 and Cycle 2 in Part A, but later onset toxicities will also be taken into account. A DLT is defined as any event that is at least possibly related to the study drugs (ASTX660 and/or Pembrolizumab)- and meets at least one of the following criteria:

- Neutropenia Grade 4 for five days or more *see note;
- Febrile neutropenia (fever of unknown origin without clinically or microbiologically documented infection) with Grade 3 or 4 neutropenia (absolute neutrophil count [ANC] <1.0 x 10^{9}/L and fever 38.5^{o}C);
- Infection (documented clinically or microbiologically) with Grade 3 or 4 neutropenia (ANC <1.0 x 109/L);
- Thrombocytopenia:: a) Asymptomatic Grade 4 for five days or more *see note, or b) >Grade 3 associated with clinically significant bleeding, or c) requiring platelet transfusion;
- Grade 4 anemia
- Any Grade 3 (or greater) elevation of hepatic transaminases (ALT or AST) OR total bilirubin lasting >48 hours will be considered a DLT with the following exceptions:
    - For patients with elevated hepatic transaminase at baseline due to documented liver metastasis (ie ≤5xULN), hepatic transaminase >8x ULN for > 48 hours will be considered a DLT.

- Any other Grade 3 (or greater) laboratory changes, with the exception of isolated laboratory changes of any grade without clinical sequelae or clinical significance that resolves to <Grade 2 within 7 days.
- Asymptomatic rises in amylase / lipase will only be considered a DLT if no improvement to <G2 following optimal supportive treatment including oral steroids.
- Rash will be considered a DLT if no improvement to <G2 following optimal supportive treatment including oral steroids.
- Any other Grade 3 (or greater) AE of clinical significance EXCLUDING:
    - Grade 3 or 4 nausea, vomiting or Grade 3 diarrhoea in patients who have not received optimal treatment with anti-emetics;
    - Alopecia of any grade
    - Grade 3 fatigue which resolves to <Grade 2 within 72 hours
    - Grade 3 rashes that resolve to < Grade 2 within 7 days with appropriate management
- Any Grade 3 (or greater) cytokine release syndrome that does not resolve to grade 2 or less within 6 hours with optimal medical management with immunosuppression (eg, corticosteroid dosing) will be considered a DLT.
- Any Grade 3 (or greater) immune-related adverse event with the exception of those that resolve to Grade 1 within 7 days of initiating corticosteroids.
- Any other toxicity that is greater than that at baseline, is clinically significant and/or unacceptable, does not respond to supportive care and results in a disruption of dosing schedule of more than 14 days.
- Any other toxicity which in the view of the investigators is considered to be a DLT at any time
- Death

* Note: In the event of a Grade 4 neutropenia or Grade 4 thrombocytopenia, a full blood count must be performed at least on Day 5 after the onset of the event to determine if a DLT has occurred. Continue to monitor the patient closely until resolution to Grade 3 or less.

Definition of Maximum Tolerated Dose and Recommended Phase 2 Dose

The MTD was defined as the dose of ASTX660 in combination with a fixed dose of Pembrolizumab with estimated DLT rate close to a target DLT rate of 25%, as recommended by the TITE-CRM model. This dose was determined following review of all the relevant toxicity data by the Safety Review Committee. The recommended Phase 2 Dose (RP2D) would be either the MTD or a dose below as recommended by the SRC taking into account the toxicity profile seen as well as the emerging pharamacokinetic and pharamacodynamic data.

The Institute of Cancer Research

ASTEROID: A Phase I trial of tolinapant (ASTX660) in combination with Pembrolizumab (MK-3475) in patients with solid tumours: utilizing triple IAP blockade as a strategy to maximize Immunogenic cell Death and the generation of an efficient adaptive immune response

Sponsor protocol number: CCR5199

EudraCT number: 2020-004648-28

Chief Investigator:

Dr Juanita Lopez
Consultant Medical Oncologist
The Institute of Cancer Research and Royal Marsden NHS Foundation Trust
Drug Development Unit
Downs Road, Sutton
Surrey, SM2 5PT
Tel: +44 (0)20 86613539
Email: juanita.lopez@icr.ac.uk

28/7/25

Sponsor:

The Institute of Cancer Research
15 Cotswold Road
Sutton
SM2 5NG

CONFIDENTIAL

ASTEROID: A Phase I trial of tolinapant (ASTX660) in combination with Pembrolizumab (MK-3475) in patients with solid tumours: utilizing triple IAP blockade as a strategy to maximize Immunogenic cell Death and the generation of an efficient adaptive immune response

Sponsor protocol number:	CCR5199
EudraCT number:	2020-004648-28
**Chief Investigator:**	Dr Juanita Lopez<br>Consultant Medical Oncologist<br>The Institute of Cancer Research and Royal Marsden NHS Foundation Trust<br>Drug Development Unit<br>Downs Road, Sutton<br>Surrey, SM2 5PT<br>Tel: +44 (0)20 86613539<br>Email: juanita.lopez@icr.ac.uk
**Sponsor:**	The Institute of Cancer Research<br>15 Cotswold Road<br>Sutton<br>SM2 5NG

PARTICIPATING INVESTIGATORS AND CENTRES:

Participating centre: Royal Marsden Hospital, Drug Development Unit
Participating address: Downs Road, Sutton
Surrey, SM2 5PT

Principal Investigator: Dr Juanita Lopez
Tel: +44 (0)20 86613539
Email: juanita.lopez@icr.ac.uk

Participating centre: Cambridge University Hospitals NHS Trust
Participating address: Department of Oncology
PO Box 193
Cambridge Biomedical Campus
Hills Road, Cambridge
CB2 0QQ

Principal Investigator: Dr Bristi Basu
Tel: +44 (0)1223 769310
Email: bristi.basu@cruk.cam.ac.uk

Participating centre: Royal Marsden Hospital, Breast Unit
Participating address: Downs Road, Sutton
Surrey, SM2 5PT
&
Fulham Road
London, SW3 6JJ

Principal Investigator: Dr Alistair Ring
Email: Alistair.Ring@rmh.nhs.uk

Participating centre: The Clatterbridge Cancer Centre NHS Foundation Trust
Participating address: 65 Pembroke Place L7 8YA

Principal Investigator: Dr Shaveta Mehta
Email: shaveta.mehta1@nhs.net

Participating centre: Leeds Teaching Hospitals NHS Trust
Participating address: St James University Hospital
Leeds
LS9 7TF

Principal Investigator: Dr Fiona Collinson
Email: fiona.collinson@nhs.net

PROTOCOL CONTRIBUTORS AND STATISTICIAN:

Protocol Contributors	Dr Alison Turner<br>Head of Investigator Initiated Research<br>The Institute of Cancer Research<br>Drug Development Unit<br>15 Cotswold Road, Sutton<br>Surrey, SM2 5NG<br>Tel: 44 (0) 20 87224303<br>Email: alison.turner@icr.ac.uk
	Dr Anna Zachariou<br>Clinical Trial Programme Manager<br>The Institute of Cancer Research<br>Drug Development Unit<br>15 Cotswold Road, Sutton<br>Surrey, SM2 5NG<br>Tel: 44 (0) 20 87224605<br>Email: anna.zachariou@icr.ac.uk
	Dr Crescens Tiu<br>Clinical Fellow in Medical Oncology<br>The Institute of Cancer Research & Royal Marsden NHS Foundation Trust<br>Drug Development Unit<br>15 Cotswold Road, Sutton<br>Surrey, SM2 5NG<br>Email: crescens.tiu@icr.ac.uk
ICR-CTSU Scientific Lead	Professor Christina Yap<br>Professor of Clinical Trial Biostatistics<br>The Institute of Cancer Research<br>Clinical Trials and Statistics Unit<br>15 Cotswold Road, Sutton<br>Surrey, SM2 5NG<br>Tel: 44 (0) 20 87224022<br>Email: christina.yap@icr.ac.uk
Trial Statistician	Xiaoran Lai<br>Medical Statistician<br>The Institute of Cancer Research<br>Clinical Trials and Statistics Unit<br>15 Cotswold Road, Sutton<br>Surrey, SM2 5NG<br>Tel: 44 (0) 20 34377202<br>Email: xiaoran.lai@icr.ac.uk

VERSION HISTORY:

Version No.	Date of issue	Reason for update
1.0	04-Aug-2021	Initial version submitted for Regulatory and Ethics approval
1.1	21-Oct-2021	Initial version approved by MHRA, HRA & REC
2.0	28-Feb-2022	IB Updates & correction of minor typographical errors
3.0	08-Jul-2022	Clarifications around dose escalation decisions
4.0	22-Sep-2023	Incorporation of two additional cohorts, B4 & B5, in the expansion phase (Part B). Addition of four new sites. Pembrolizumab IB update.

TABLE OF CONTENTS

TITLE PAGE
TABLE OF CONTENTS
LIST OF ABBREVIATIONS AND DEFINITION OF TERMS
PROTOCOL SIGNATURES

1 PROTOCOL SUMMARY 11
1.1 Full title: 11
1.1.1 Short title: 11
1.2 Clinical trial primary objectives and endpoints 11
1.3 Design 11
1.3.1 Study treatment: 11
1.3.2 Patient Population 12
2 INTRODUCTION 13
2.1 Background 13
2.1.1 IAPs at the cross roads of cell-death and cancer immunity 13
2.1.2 Smac mimetics in combination with PDL-1 inhibitors 13
2.2 Investigational medicinal product 14
2.2.1 Structure of ASTX660 14
2.2.2 Mechanism of action of ASTX660 14
2.2.3 Non-clinical anti-tumour activity 15
2.2.4 Animal pharmacology, toxicology and pharmacokinetics/pharmacodynamics 16
2.3 Clinical experience 17
2.4 Background on combination agent-Pembrolizumab 19
2.4.1 Structure of Pembrolizumab 19
2.4.2 Mechanism of action of Pembrolizumab 19
2.4.3 Non-clinical anti-tumour activity 19
2.4.4 Animal pharmacology, toxicology and pharmacokinetics / pharmacodynamics 19
2.4.5 Clinical experience with Pembrolizumab 20
2.5 Rationale for the proposed trial 21
3 TRIAL DESIGN 22
3.1 Clinical trial objectives and endpoints 22
3.1.1 Primary objectives and endpoints 22
3.1.2 Secondary objectives and endpoints 22
3.1.3 Tertiary objectives and endpoints 22
3.2 Design of the clinical trial 23
3.3 Definition of dose limiting toxicity 27
3.4 Definition of maximum tolerated dose and Recommended Phase 2 Dose 28
3.5 Patient evaluability 28
3.5.1 Safety 28
3.5.2 Dose Escalation 28
3.5.3 Response 29
4 PATIENT SELECTION 30
4.1 Eligibility criteria 30
4.1.1 Inclusion criteria: 30
4.1.2 Exclusion criteria: 32
4.2 Patient enrolment 35
5 TREATMENT 36
5.1 Selection of the Phase I starting dose and schedule 36

5.2 Dosing schedule/treatment schedule ... 36
5.3 Safety Review Committee ... 37
5.4 Dose escalation scheme ... 40
5.4.1 Intra-patient dose escalations ... 40
5.5 Dose expansion scheme ... 41
5.6 Dose modifications ... 42
5.6.1 Dose reductions ... 42
5.6.2 Dose delays ... 42
5.6.3 Dose interruptions due to COVID-19 ... 43
5.7 Toxicity management guidelines ... 43
5.7.1 Management of pembrolizumab-related toxicity ... 44
5.7.2 Management guidelines for toxicity related to both ASTX660 and pembrolizumab ... 47
5.7.3 Management of infusion reactions ... 50
5.8 Duration of treatment ... 51
5.8.1 Replacement of patients ... 52
5.9 Concomitant medication and treatment ... 52

6 PHARMACEUTICAL INFORMATION ... 54

6.1 Supply of ASTX660 and pembrolizumab ... 54
6.2 Pharmaceutical data ... 54
6.2.1 Formulation of ASTX660 ... 54
6.2.2 Storage conditions for ASTX660 ... 54
6.2.3 ASTX660 administration ... 54
6.2.4 Formulation of Pembrolizumab ... 54
6.2.5 Storage conditions for Pembrolizumab ... 55
6.2.6 Pembrolizumab administration ... 55
6.3 ASTX660 and Pembrolizumab accountability ... 55

7 INVESTIGATIONS SCHEDULE ... 56

7.1 Pre-treatment evaluations ... 56
7.1.1 Obtaining written informed consent ... 56
7.1.2 Evaluations within four weeks (28 days) ... 57
7.1.3 Evaluations within two weeks (14 days) ... 57
7.1.4 Evaluations within one week (seven days) ... 57
7.2 Evaluations during the trial ... 58
7.3 Evaluations at ‘off-study’ visit ... 59
7.4 Patient-reported outcomes ... 59
7.5 Follow-up ... 60
7.6 Schedule of events ... 61

8 PHARMACOKINETIC AND PHARMACODYNAMIC ASSESSMENTS ... 67

8.1 Pharmacokinetic Assays ... 67
8.2 Pharmacodynamic assays ... 67
8.2.1 Collection of archival tumour specimens ... 67
8.2.2 Collection of tumour biopsy material for pharmacodynamic analysis ... 67
8.2.3 Collection of blood samples for pharmacodynamic analysis ... 68
8.2.4 Schedule of research sample collection ... 69

9 ASSESSMENT OF SAFETY ... 72

9.1 Adverse event definitions ... 72
9.1.1 Adverse event ... 72
9.1.2 Serious adverse events (SAEs) ... 73
9.1.3 Determining adverse event causality ... 74
9.1.4 Expectedness ... 74
9.1.5 Suspected, unexpected, serious, adverse reactions (SUSARs) ... 74
9.2 Expedited reporting of serious adverse events to the Pharmacovigilance Officer, DDU ... 74
9.2.1 Events exempt from being reported as SAEs ... 75

9.3 Recording of adverse events and serious adverse events in eCRFs 75
9.3.1 Recording of anaemia and hypokalaemia 76
9.4 Follow-up of adverse events 76
9.5 Urgent safety measures 76
9.6 Pregnancy 77

10 ASSESSMENT OF EFFICACY 78

10.1 Measurement of disease 78
10.2 Timing and type of tumour assessments 78
10.2.1 Immune-modified RECIST 78
10.2.2 RANO / iRANO Criteria 79
10.2.3 Evaluations during and at 'off-study' 79
10.3 Tumour response 79
10.3.1 Recording of response in the eCRF 80

11 DISCONTINUATION OF TREATMENT/ WITHDRAWAL FROM TRIAL 81

12 DEFINING THE END OF TRIAL 82

13 DATA ANALYSIS AND STATISTICAL CONSIDERATIONS 83

13.1 Statistical considerations and analysis populations 83
13.1.1 Escalation phase 83
13.1.2 Expansion phase 83
13.2 Presentation of data 85
13.3 Safety 86
13.4 Pharmacokinetics 86
13.5 Pharmacodynamics 86
13.6 Anti-tumour activity 86

14 ADMINISTRATION 87

14.1 Protocol deviations and amendments 87
14.2 Completion of the case report form (CRF) 87
14.3 Trial performance and monitoring 87
14.3.1 Management of the study 87
14.3.2 Source document verification 88
14.4 Clinical study report 88
14.5 Record retention 88
14.6 Ethical considerations 89
14.7 Indemnity 89
14.8 Publication policy and press releases 89

15 REFERENCES 90

16 APPENDICES 93

16.1 APPENDIX 1: WHO PERFORMANCE SCALE 93
16.2 APPENDIX 2: DECLARATION OF HELSINKI 94
16.3 APPENDIX 3: MEASUREMENT OF DISEASE 97
16.4 APPENDIX 4: NEW YORK HEART ASSOCIATION (NYHA) SCALE 105
16.5 APPENDIX 5: CONTRACEPTIVE GUIDANCE AND PREGNANCY TESTING 106
16.6 APPENDIX 6: ADVERSE EVENTS: DEFINITIONS AND PROCEDURES FOR RECORDING, EVALUATING, FOLLOW UP AND REPORTING 109
16.6.1 Definition of AE 109
16.6.2 Definition of SAE 110
16.6.3 Additional Events Reported in the Same Manner as SAE 111
16.6.4 Recording AE and SAE 111
16.6.5 Reporting of AEs, SAEs, and Other Reportable Safety Events to Merck and Astex 114
16.7 APPENDIX 7: RANO CRITRIA FOR GLIOBLASTOMAS AND HIGH GRADE GLIOMAS 115
16.8 APPENDIX 8: iRANO CRITERIA 117

LIST OF ABBREVIATIONS AND DEFINITION OF TERMS

	Abbreviation	Definition
**A**	ABPI	Association of the British Pharmaceutical Industry
	AE	adverse event
	alk phos	alkaline phosphatase
	ALT	alanine aminotransferase
	ANC	absolute neutrophil count
	AST	aspartate aminotransferase
	AUC	area under the curve
**B**	BAD	biologically active dose
	BP	blood pressure
	BSA	body surface area
**C**	^{0}C	degrees Celsius
	CI	Chief Investigator
	CLT	total body clearance
	C_{max}	maximum observed plasma concentration
	CR	complete response
	CRA	Clinical Research Associate
	CRF	case report form
	CT	computerised tomography
	CTA	clinical trial authorisation
	CTCAE	Common Terminology Criteria for Adverse Events
	CTM	Clinical Trial Manager
**D**	Day	calendar day
	DCF	data clarification form
	DDU	Drug Development Unit
	DLT	dose limiting toxicity
**E**	ECG	electrocardiogram
	ECMC	Experimental Cancer Medicine Centres
	EDTA	ethylene diamine tetra-acetic acid
	EORTC	European Organisation for Research and Treatment of Cancer
	EPD	early progressive disease
**F**	FDG	fluorodeoxyglucose
	FU	Formulation Unit
	GCP	Good Clinical Practice
	g/dL	gram(s) per decilitre
	GMP	Good Manufacturing Practice
	GTAC	Gene Therapy Advisory Committee
**H**	Hb	haemoglobin
	HR	Hazard Ratio
	HCG	human chorionic gonadotropin
	HIV	human immunodeficiency virus
**I**	ICH GCP	International Conference on Harmonisation of Good Clinical Practice
	IHC	Immunohistochemistry
	IMP	investigational medicinal product
	ITF	Investigator Trial File
**L**	LVEF	left ventricular ejection fraction
**M**	MAD	maximum administered dose
	mg/m^2	milligram per square metre
	MHRA	Medicines and Healthcare products Regulations Agency
	MRI	magnetic resonance imaging

LIST OF ABBREVIATIONS AND DEFINITION OF TERMS

	Abbreviation	Definition
	MRT	mean residence time
	MTD	maximum tolerated dose
**N**	NCI	National Cancer Institute
**P**	PBMC	Peripheral blood mononuclear cells
	PD	progressive disease
	PET	positron emission tomography
	PI	Principal Investigator
	PK	pharmacokinetic
	PR	partial response
	PRP	Platelet-rich plasma
**Q**	QC	quality control
	QP	Qualified Person
**R**	REC	Research Ethics Committee
	RECIST	Response Evaluation Criteria in Solid Tumours
	RPM	revolutions per minute
**S**	SAE	serious adverse event
	SD	stable disease
	SGDCF	site generated data clarification form
	SDV	source data verification
	SOP	standard operating procedure
	SPC	Summary of Product Characteristics
	SUSAR	suspected unexpected serious adverse (drug) reaction
**T**	$T_{1/2}$	terminal elimination half-life
	TITE-CRM	Time to Event-Continual Reassessment Method
	T_{max}	time to reach C_{max}
**U**	ULN	upper limit of normal
	USM	urgent safety measure
**V**	V_{SS}	steady state volume of distribution
**W**	WFI	Water for Injection
	WBC	white blood cell
	WHO	World Health Organisation

PROTOCOL SIGNATURES

Investigator Signature:

I have read and agree to the protocol, as detailed in this document. I am aware of my responsibilities as an Investigator under the UK Clinical Trials Regulations[1], the guidelines of Good Clinical Practice (GCP)[2], the Declaration of Helsinki (Appendix 2), the applicable regulations of the relevant NHS Trusts and the trial protocol. I agree to conduct the trial according to these regulations and guidelines and to appropriately direct and assist the staff under my control, who will be involved in the trial, and ensure that all staff members are aware of their clinical trial responsibilities.

INVESTIGATOR'S NAME: ______________________________

SIGNATURE: ______________________________

DATE: ______________________________

[1] The Medicines for Human Use (Clinical Trials) Regulations (S.I. 2004/1031) and any subsequent amendments to it.

[2] ICH Harmonised Tripartite Guideline E6: Note for Guidance on Good Clinical Practice (CPMP/ICH/135/95) Step 5, adopted by CPMP July 1996.

1 PROTOCOL SUMMARY

1.1 Full title:

ASTEROID: A Phase I trial of tolinapant (ASTX660) in combination with Pembrolizumab in patients with solid tumours: utilizing triple IAP blockade as a strategy to maximize Immunogenic cell Death and the generation of an efficient adaptive immune response

1.1.1 Short title:

ASTEROID: Phase I trial of ASTX660 in combination with Pembrolizumab in solid tumours

1.2 Clinical trial primary objectives and endpoints

Primary objectives	Endpoints
To propose a recommended dose for Phase II (RP2D) evaluation by establishing the maximum tolerated dose of ASTX660 in combination with Pembrolizumab,	Determining the Maximum Tolerated Dose (MTD) based on dose-limiting toxicities (DLTs) up to 6 weeks from start of treatment (as defined in Section 3. 3 of the protocol) and the RP2D.
To assessing the safety and tolerability of ASTX660 and Pembrolizumab when given in combination.	Determining the frequency, severity and causality of each adverse event to the combination of ASTX660 and Pembrolizumab according to the National Cancer Institute (NCI) Common Terminology Criteria for Adverse Events (CTCAE) Version 5.0.
Part B only<br>To evaluate the preliminary anti-tumour activity of ASTX660 in combination with Pembrolizumab.	Disease response by imRECIST criteria, clinical benefit rate, best changes in tumour size, progression-free survival and duration of response.

Full details of secondary and tertiary objectives can be found in Section 3.1.

1.3 Design

This is prospective, open label, multi-centre phase I non-randomised TITE-CRM study, consisting of an initial dose escalation phase and followed by an expansion phase.

1.3.1 Study treatment:

In both Part A (escalation) and Part B (expansion) ASTX660 will be administered daily, 7 days on / 14 days off. Pembrolizumab will be administered on Day 8 of every cycle. The starting dose of ASTX660 will be 90mg given orally. Pembrolizumab will be administered as an intravenous infusion, at a fixed dose of 200mg throughout the study. Dose escalation will utilise a flexible 2-stage time to event Continual Reassessment Method (TITE-CRM) design to obtain the MTD and RP2D of ASTX660 in combination with a fixed dose of Pembrolizumab.

1.3.2 Patient Population

Part A: Up to 18 evaluable patients with advanced solid tumours are planned to be enrolled onto the dose escalation part of the study.

Part B: In the expansion phase, a total of up to 50 patients will be enrolled in five separate groups; patients with Immune checkpoint refractory tumours (n=10), patients with cervical cancer (n=10), patients with triple negative breast cancer (n=10), patients with relapsed glioblastoma (n=10) and patients with ER+ HER2- breast cancer (n=10).

2 INTRODUCTION

2.1 Background

2.1.1 IAPs at the cross roads of cell-death and cancer immunity

Cellular inhibitor of apoptosis protein 1/2 (cIAP1/2) and X-linked inhibitor of apoptosis protein (XIAP) are key members of the inhibitor of apoptosis (IAP) protein family that function as important gatekeepers of cell death and pro-survival inflammatory and immunogenic signalling pathways (1). Although best known for their ability to regulate caspases, IAPs also influence ubiquitin-dependent pathways regulating the 'Ripoptosome' – a receptor-interacting protein kinase 1 (RIPK1)-dependent cytosolic signalling platform responsible for the integration of cellular stress, innate immune signalling pathways as well as cell death (2-5). This leads to the orchestration of inflammatory signalling including NF-κB activation which are critical for eliciting efficient T cell cross-priming (6).

In response to TNFα, cIAPs ubiquitinate RIPK1, promoting the formation of complexes which activate survival signalling through the canonical NF-κB pathway. Simultaneously, formation of the death-inducing signalling complex (DISC), which drives apoptosis, is prevented. Removal of cIAP1/2 allows the DISC to form, leading overall to a switch in TNFα signalling from pro-survival to proapoptotic. Once loss of cIAP1/2 is combined with release of the XIAP-mediated block on caspases, which is essential for full activation of apoptosis, a sustained proapoptotic effect in the presence of TNFα is obtained. Antagonism and subsequent degradation of the cIAP1/2 also leads to the stabilization of NIK (NF-κB-inducing kinase), which activates the non-canonical NF-κB pathway, resulting in enhancement of T-cell co-stimulation (7).

2.1.2 Smac mimetics in combination with PDL-1 inhibitors

Small-molecule antagonists of the IAPs, known as Smac mimetic compounds, bind to IAPs, preventing their binding to caspases, but also inducing the auto-ubiquitination and subsequent proteasomal-mediated degradation of these IAPs. Smac-mediated antagonism of IAPs sensitizes cancer cells to death ligands originating from the immune system through the formation of the ripoptosome (2). In parallel, Smac mimetic mediated depletion of IAPs in immune cells leads to the activation of the NF-κB pathway which is postulated to produce co-stimulatory signals that stimulate dendritic cell maturation (8) and efficient cross-priming of effector T cells (6) (Figure 1).

Preclinical models testing first generation Smac mimetics in combination with anti-PD-1 have shown significant efficacy with durable cures in syngeneic mouse models of cancer (9).

ASTX660 is a potent, non-peptidomimetic, antagonist of cIAP1, cIAP2 and XIAP. Combined antagonism of cIAP1/2 and XIAP may offer improved efficacy over first-generation Smac mimetics that are more cIAP1/2 selective. ASTX660 has been shown to have tumor growth inhibitory activity and potent proapoptotic activity in nonclinical models (see Section 4.1 of IB). ASTX660 is being developed in a first-in-human (FIH) phase I-II study for the treatment of patients with advanced solid tumors or lymphoma and for whom standard life-prolonging measures are not available (NCT02503423).

As such, it is postulated that ASTX660 may more efficiently push cancer cells into dying by necroptosis or inflammatory apoptosis; and equally more efficiently initiate an adaptive immune response that could be augmented by immune-checkpoint blockade.

ASTX660 has been combined in vivo with anti-PD-L1 in a murine syngeneic tumour model CT26. Significant tumour regression and enhanced survival was seen with the combination regardless of single agent ASTX660 or anti-PD-L1 activity (Astex, in house data). Combination trials of first generation cIAP1/2 selective antagonists together with immune checkpoint inhibitors are ongoing (NCT03270176, NCT02890069).

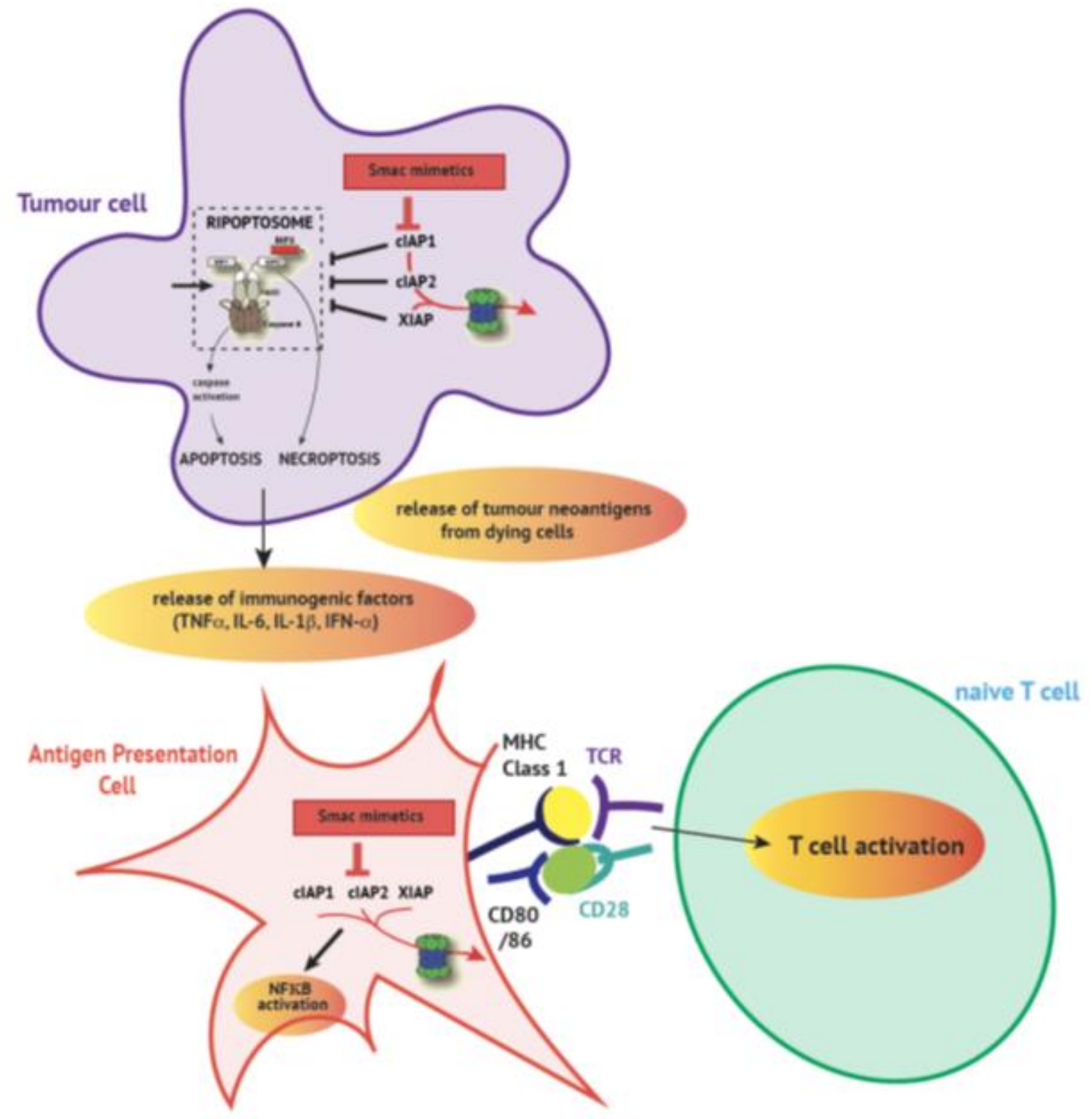

Figure 1 - (top) Redundancy of cIAP1/2 and XIAP in 'guarding' the Ripoptosome. Inhibition of cIAP1/cIAP2 as well as XIAP by ASTX660 is hypothesized to increase tumour immunogenic cell death by more efficient activation of the ripoptosome. Together with the activation of NfKB, this promotes dendritic cell activation enahncing antigen presentation and the generation of an efficient immune response.

2.2 Investigational medicinal product

ASTX660 is a potent, non-peptidomimetic antagonist of the cellular and X-linked inhibitors of apoptosis proteins (cIAP1/2andXIAP), which is currently being tested in a first in human phase I-II study in patients with advanced solid tumors and lymphomas (NCT02503423, AbstractPS1073).

2.2.1 Structure of ASTX660

ASTX660 is a synthetic small molecule. The drug substance is L-(+)-lactic acid salt of ASTX660: $C_{30}H_{42}FN_5O_3{\cdot}C_3H_6O_3$.

2.2.2 Mechanism of action of ASTX660

ASTX660 is a dual antagonist of cellular inhibitor of apoptosis protein 1 (cIAP1) and X-linked inhibitor of apoptosis protein (XIAP).

2.2.3 Non-clinical anti-tumour activity

The anti-proliferative effect of ASTX660 and its dependence on TNF-α were investigated in a panel of cell lines, including breast, melanoma, and lung.

ASTX660 potently inhibited the proliferation of EVSA-T and MDA-MB-231 breast cancer cell lines with IC50 values of 0.43 nM and 1.8 nM, respectively. These cell lines produce sufficient levels of TNF-α to allow IAP antagonists to demonstrate single-agent activity (TNF-α blockade was shown to eliminate this activity). Proliferation of the cell lines (HCC1806, A375, SK-MEL-28, Calu-6, NCI-H2122, NCI-H647, and SW900) was also inhibited by ASTX660, at different levels of potency, but only in the presence of 1 ng/mL TNF-α. In the absence of exogenous TNF-α, the proliferation of these cells was unaffected by ASTX660 treatment since these cells do not produce sufficient TNF-α themselves to elicit an apoptotic effect, indicating that the antiproliferative effects observed were due to antagonism of IAPs rather than off-target activity.

A proportion of the breast cancer and lung cancer cell lines tested were insensitive to ASTX660 with or without TNF-α (HCC-1937, A549, Calu-1, NCI-H1155).

Work with an early lead, tested in a cell line panel, suggested a high level of sensitivity to IAP antagonism in melanoma and leukaemia cell lines in the presence of TNF-α. The mechanism of this sensitivity was investigated further in melanoma and lymphoma using ASTX660. Induction of apoptosis by ASTX660 was investigated in detail in the melanoma cell lines A375 and SK-MEL-28, and the lymphoma cell line, WSU-DLCL2. In melanoma cell lines, treatment with 1 μM ASTX660 led to a degradation of both cIAP1 and cIAP2; apoptosis was only observed when treatment was combined with TNF-α, as demonstrated by an increase in the levels of cleaved poly-ADP ribose polymerase (PARP), caspase-3, and caspase-9. In contrast, caspase-3 cleavage was observed in ASTX660-treated WSU-DLCL2 lymphoma cells and this apoptotic response was independent of TNF-α.

A number of studies were carried out where MDA-MB-231 tumour-bearing mice were treated with single oral doses of ASTX660 (10, 20, and 30 mg/kg). Tumours were removed at various time points from 0.5 hour up to 7 days after dosing, and the levels of cIAP1 in tumours were measured by western blot and Meso Scale Discovery (MSD). Levels of cIAP were rapidly reduced to ~30% of the control levels at 1 hour after dosing with 20 mg/kg ASTX660 and remained at around this level for up to 72 hours, after which they returned to control levels by 7 days after ASTX660 treatment. This indicates that antagonism by ASTX660 resulted in degradation of cIAP1. The duration of this effect was related to the treatment dose, with levels of cIAP1 recovering more quickly after a lower dose of 10 mg/kg ASTX660. In addition, an increase in 2 apoptosis markers (cleaved PARP and cleaved caspase-3) was detected at 6, 24, and 72 hours after dosing, indicating that ASTX660 induced apoptosis in these tumours.

The effect of ASTX660 on XIAP binding to SMAC or caspase-9 was measured in mice bearing tumours derived from the engineered HEK293 cells overexpressing FLAG-tagged XIAP and caspase-9. These mice were treated with single oral doses of ASTX660 at 20 mg/kg and tumours were removed at time points from 1 day up to 7 days after dosing (data not shown; BIOL_2014_009). Binding of XIAP to SMAC and to caspase-9 was reduced to below control levels for up to 3 days after ASTX660 treatment, indicating that ASTX660 was antagonizing the binding of XIAP to both SMAC and caspase-9.

The effect of ASTX660 on XIAP binding to SMAC was also measured in MDA-MB-231 tumour-bearing mice. After a single 20-mg/kg dose of ASTX660, tumours were removed at time points from 0.5 hour up to 7 days after dosing. XIAP binding to SMAC in tumours was rapidly reduced to below 10% of control levels at 1 hour after dosing, remaining at these low levels for up to 6 hours after dosing. Levels began to increase again by 24 hours after dosing, but remained below control levels for up to 5 days after ASTX660 treatment. These data indicate that ASTX660 was interfering with the direct binding of XIAP to SMAC.

These data demonstrate that treatment with a single dose of ASTX660 antagonizes both cIAP1 and XIAP in tumours. The initial effects of this antagonism can be rapidly observed for both targets (inhibition of binding of XIAP to SMAC and cIAP1 degradation), but XIAP:SMAC binding recovers more rapidly

than cIAP1 levels, probably because the antagonism of cIAP leads to an actual loss of protein through degradation. These doses resulted in measurable levels of ASTX660 in tumour tissue.

2.2.4 Animal pharmacology, toxicology and pharmacokinetics/pharmacodynamics

ASTX660 binds to the isolated baculovirus inhibitor of apoptosis protein [IAP] repeat-3 (BIR3) domains of both XIAP and cIAP1 in vitro with nanomolar potency. Dual antagonism of XIAP and cIAP by ASTX660 has been demonstrated in cell lines and in vivo (in xenograft tissue), by measuring the association of XIAP with caspase-9 or second mitochondria-derived activator of caspase (SMAC) and the degradation of cIAP, respectively. Modulation of cIAP1 has also been demonstrated in peripheral blood mononuclear cells (PBMCs) from rats as a surrogate tissue. Tumour growth was inhibited in human cancer xenograft mouse models, including MDA-MB-231 and A375, in response to oral treatment with ASTX660, over a range of doses and schedules, including an intermittent dosing schedule of 7 days on/7 days off/7 days on/7 days off.

Several toxicology studies were completed with ASTX660 in 2 species (rat and cynomolgus monkey) to evaluate the nonclinical safety profile of orally administered ASTX660. Clinical evaluation of ASTX660 is supported by 2 GLP toxicology studies of ASTX660: one conducted in rats (a 28-day repeat-dose study with continuous daily dosing) and the other in cynomolgus monkeys (a 21-day repeat-dose study with an intermittent dosing schedule of daily dosing for 7 days on/7 days off/7 days on, referred to as “intermittent 7+7+7 dosing regimen,” similar to that proposed for the first-in-human study). The dose of ASTX660 that was severely toxic in 10% of rodents (STD10) with up to 28 consecutive days of daily oral dosing in the GLP rat study was 15 mg/kg/day; ASTX660 was not tolerated at 22.5 mg/kg/day in this continuous-type dosing regimen in the rat. The highest nonseverely toxic dose (HNSTD) in the GLP monkey study using the intermittent 7+7+7 dosing regimen with a 4-week recovery period was 30 mg/kg/day. In the 28-day repeat dose GLP monkey study, drug accumulation during the second 7-day dosing period was consistent with that seen in the first 7-day dosing period.

In a recent 92-day repeat dose GLP subchronic toxicity evaluation in monkeys, using an intermittent dosing regimen followed by a 4-week recovery period, the no observable adverse effect level was 12.5 mg/kg/day, and the HNSTD was 30 mg/kg/day. Overall, the toxicology findings in rats and monkey were consistent with and presumed to be secondary to systemic proinflammatory changes caused by the exaggerated pharmacological action of ASTX660 (IAP antagonism). ASTX660 did not show any evidence of genotoxicity or induction of chromosome damage in the bacterial reverse mutation ("Ames") test, the human peripheral blood lymphocyte (PBL) in vitro test, or the rat micronucleus test, when tested in accordance with regulatory guidelines.

Pharmacokinetic (PK) parameters of ASTX660 were analysed in the mouse, rat, and monkey; toxicokinetic studies were conducted in the rat and monkey. Following IV administration, ASTX660 had plasma clearance that approximated half liver blood flow in all species. ASTX660 appeared to readily distribute throughout the body, having a moderate to large V_{ss} ranging from 2.0 to 9.3 L/kg (in mouse and rat, respectively). The half-life of ASTX660 ranged from 1.9 in rodents up to 8.9 hours in monkeys. Following oral administration, ASTX660 was bioavailable in all species evaluated. There was evidence of supraproportional PK with increasing dose in all species, manifested by an increase in bioavailability. Mild-to-moderate (2- to 3-fold) drug accumulation was observed in rat and monkey after repeat dosing.

ASTX660 was primarily metabolised by human CYP3A4 in vitro and was a weak-to-moderate inhibitor of CYP3A4. ASTX660 showed a low potential for inhibition of the other major human CYPs and underwent minimal metabolism by these CYPs. There is potential for a clinical drug-drug interaction through the modulation of ASTX660 metabolism by a co-administered potent CYP3A4 inhibitor or inducer. There was no evidence of CYP3A induction in human plasma samples. Exposure of cultured primary human hepatocytes to ASTX660 did not result in induction of mRNA expression or enzyme activity for CYP isoforms 1A2, 2B6, and 3A4. ASTX660 was eliminated by multiple mechanisms following IV and oral administration in rats. A significant proportion of the dose was excreted in the urine

as unchanged parent compound (34%) in the first 24 hours following IV administration, with a further 6.5% eliminated in the bile. The remainder of the dose is assumed to be eliminated by metabolism. This suggests both renal elimination and hepatic metabolism are important routes of ASTX660 clearance in vivo.

Four apparently major metabolites of ASTX660 (M1-M4) were identified in both rats and monkeys following single-dose oral administration. All 4 metabolites were found in rat/monkey bile; only M3 was found in plasma. Preliminary characterization of metabolites have identified 14 putative metabolites of ASTX660 in postdose human plasma samples, including 2 potentially major metabolites pending further confirmation. Results of an in vitro study of the interaction of ASTX660 with human ABC and uptake transporters indicate that ASTX660 may act as a potent inhibitor of efflux transporter MDR1 and uptake transporters OCT1, OCT2, and MATE1.

For additional information concerning ASTX660, refer to the Investigator Brochure.

2.3 Clinical experience

The First In Human clinical trial (ASTX660-01) is in progress. ASTX660-01 is a Phase 1-2, open-label study of ASTX660 in subjects with advanced solid tumours or lymphoma and for whom standard life-prolonging measures are not available. Objectives of the Phase 1 portion of the study were to assess the safety and PK parameters of orally administered ASTX660, determine the maximum tolerated dose (MTD) and recommended Phase 2 dose (RP2D), and obtain preliminary efficacy and target engagement data. Phase 1 enrolment is complete and all Phase 1 subjects have discontinued study treatment. The MTD and RP2D have been determined (210 mg/day and 180 mg/day, respectively). The recommended intermittent dosing regimen (fixed dose) is once a day for 7 consecutive days every other week of each 28-day cycle (ie, [7 days on/7 days off] ×2; daily dosing on Days 1-7 and 15-21).

The primary objective of the Phase 2 portion of the study is to explore the single-agent antitumor activity of ASTX660 in several specific tumour types that have potential biomarkers characterized by a molecular feature that may confer sensitivity to ASTX660. Phase 2 enrolment is in progress; all subjects receive ASTX660 at the RP2D (180 mg/day).

As of the data cut-off date (04 June 2020, 193 subjects have received at least 1 dose of study treatment in the ASTX660-01 study, as follows:

- 45 subjects in Phase 1, including 37 in the dose-escalation stage of the study (Part 1) and 8 in the dose-expansion stage (Part 2). Overall in Phase 1, 78% of subjects received 2 or more cycles of study treatment (mean ± SD=2.4±2.3 cycles).
- The MTD and recommended Phase 2 dose were determined at 210 mg/day and 180 mg/day (fixed dose), respectively.
- A total of 148 subjects have been treated with ASTX660 at a starting dose of 180 mg/day in the ongoing Phase 2 part of the study. As of the reporting date, across all cohorts, the median number of cycles received was 3.7 (range from 1 to 24). Forty subjects (27.6%) received ≥1 dose reduced cycle; 29 (20.0%) subjects required a delayed treatment cycle.

In Part 1 (dose escalation) of Phase 1, the dose of ASTX660 was escalated from 15 mg/day to 270 mg/day (fixed dosing) based on a standard 3+3 study design. ASTX660 administered orally using an intermittent dosing schedule (ie, 7 days on/7 days off) demonstrated a manageable safety profile. DLTs included asymptomatic Grade 3 lipase increased or hyperlipasaemia with or without Grade 3 amylase increase in 1 subject at the 210 mg/day dose level and in 3 subjects at the 270 mg/day dose level. Most DLTs of Grade 3 increased lipase resolved or lessened in severity and the subjects were able to resume dosing with ASTX660 after dose interruption and/or reduction.

ASTX660 at 210 mg/day was determined to be the MTD. After reviewing all available data from Phase 1 Part 1 of the study, the Data Safety Review Committee (DSRC) recommended 180 mg/day as the recommended Phase 2 dose (RP2D). To confirm the RP2D, enrolment was expanded at the 180-

mg/day dose level in Phase 1 Part 2 (dose expansion). A total of 17 subjects received ASTX660 at the 180-mg/day dose level in Phase 1 (dose escalation and dose expansion combined). No DLTs, and no treatment-related AEs of increased lipase, hyperlipasaemia, or increased amylase (of any severity grade), were reported among the 17 subjects who received ASTX660 at the 180-mg/day dose level in Phase 1.

Overall in Phase 1, the most common study treatment-related AEs (≥10%) were nausea (22%), pruritus (18%), vomiting (18%), and fatigue (13%). In addition, rash (including rash maculopapular) was reported in >10% of subjects. The most common Grade 3 AE related to study treatment was lipase increased (3 subjects; 7%); all other Grade 3 AEs occurred in 1 subject (2%) each, including hyponatraemia and hypophosphataemia (both in the same subject), hyperlipasaemia, lymphopoenia, amylase increased, and anaemia. No subjects in Phase 1 in any dose group had Grade 4 or 5 AEs related to study treatment or SAEs related to study treatment.

In Phase 2, up to 04 June 2020, the most common treatment-related AEs (≥15%) were lipase increased/hyperlipasaemia (N=43; 29.6%), rash /rash maculo-papular (N=42; 28.9%), amylase increased (N=38; 26.2%), alanine aminotransferase increased (N=25; 17.2%), and fatigue (N=25; 17.2%). The most common (≥5%) Grade ≥3 treatment-related AEs included lipase increased/hyperlipasaemia (N=19; 13.1%), rash /rash maculo-papular (N=19; 13.1%), and amylase increased (N=11; 7.6%). A total of 70 (48.3%) subjects experienced SAEs (regardless of relationship with study drug) in Phase 2, with the most common events including pneumonitis (N=5 [3.4%]), and pneumonia, pyrexia, and tumour pain (N=4; 2.8% for all 3 event terms). Serious adverse reactions (SARs) were observed in 27 (18.6%) subjects. The most common SARs included pneumonitis (N=5; 3.4%), rash maculo-papular (N=3; 2.1%), and pancreatitis (N=2; 1.4%); all other SARs were observed in 1 subject (0.9%) each. As of the data cutoff, 16 (11.0%%) subjects discontinued treatment due to an AE.

Overall, 4 subjects have died due to SAEs. Fatal SAEs considered related to ASTX660 by the investigator include sepsis and pneumonia (N=1; 0.5% each). A single life-threatening SAE considered related to ASTX660 by the investigator was observed: intestinal perforation (N=1; 0.5%).

Of 45 subjects enrolled in Phase 1, one subject with cutaneous T-cell lymphoma had a clinically meaningful improvement in skin lesions, as reported by the investigator. In Phase 2, as of the data cut-off for the latest IB update, the objective response rates (ORRs) in Cohort 3 (peripheral T cell lymphoma [PTCL]) and Cohort 4 (cutaneous T cell lymphoma [CTCL]) were 19.2% and 20.7%, respectively. One partial response was reported in a subject with metastatic melanoma enrolled in Cohort 5 after 12 cycles of treatment. No objective responses were reported in the head and neck squamous cell carcinoma (HNSCC), diffuse large B-cell lymphoma (DLBCL), and cervical cohorts.

Overall, PK parameters were assessed in Cycle 1 and 2, and were similar between cycles. As such, only Cycle 1 PK data are presented here. ASTX660 was detected at all doses tested in this study and was characterized by fast absorption with a median time to maximum measured concentration in plasma [Cmax] (Tmax) of approximately 1 hour for both powder in bottle (PiB) and Capsule formulations. Mean plasma concentrations of ASTX660 exhibited a biphasic profile with a secondary peak for some cohorts more prominent on Day 7 than Day 1. The cause of the secondary peak is currently unknown. There was an approximately 2-fold accumulation for AUC from time 0 to 24 hours after dosing (AUC0-24) across doses on Day 7 vs Day 1, but minimal to no accumulation for Cmax. No accumulation in AUC0-24 or Cmax exposure was evident from cycle to cycle. ASTX660 AUC0-24 and C_{max} exposures increased in a dose-proportional manner in the range of 15 to 180 mg/day and supraproportionally at 180 to 270 mg/day dose ranges. At the RP2D (180 mg/day Capsule), exposure levels reached the target therapeutic AUC exposure range established in mouse xenograft models.

At the time of data cutoff, for Phase 2, evaluable preliminary PK data were available for 55 and 104 subjects on C1D1 and C1D7, respectively. The exposures were highest for the DLBCL cohort and lowest for the molecularly selected (ie, other solid tumors with a molecular rationale) cohort. In addition, CTCL and PTCL cohorts had the 2nd and 3rd lowest exposures.

Changes in cIAP1 protein levels were measured from PBMC extracts, at Baseline and during treatment. A rapid and robust degradation of cIAP1 was observed in Cycle 1 at all ASTX660 doses for 37 subjects with evaluable samples and was maintained through Cycle 2 at or above 180 mg/day for 30 subjects with evaluable samples.

Cytokines and other inflammatory markers such as TNF-α, IL-6, IL-8, and C-reactive protein (CRP) were measured in samples from 42 subjects. Many cytokines and other inflammatory markers were undetectable (below the limit of quantitation of the assay) in plasma and, of those detected, no consistent trend or substantial change from baseline was observed with increasing dose levels of ASTX660.

2.4 Background on combination agent-Pembrolizumab

2.4.1 Structure of Pembrolizumab

Pembrolizumab is a potent humanized immunoglobulin G4 (IgG4) monoclonal antibody (mAb) with high specificity of binding to the PD-1 receptor, thus inhibiting its interaction with PD-L1 and PD-L2.

2.4.2 Mechanism of action of Pembrolizumab

The PD-1 receptor is a negative regulator of T-cell activity that has been shown to be involved in the control of T-cell immune responses. Pembrolizumab potentiates T-cell responses, including anti-tumour responses, through blockade of PD-1 binding to PD-L1 and PD-L2, which are expressed in antigen presenting cells and may be expressed by tumours or other cells in the tumour microenvironment.

2.4.3 Non-clinical anti-tumour activity

Based on preclinical in vitro data, pembrolizumab has high affinity and potent receptor blocking activity for PD-1.

Therapeutic studies in mouse models have shown that administration of antibodies blocking the PD-1/PD-L1 interaction enhances infiltration of tumors by CD8+ T-cells and leads ultimately to tumor rejection. Anti-mouse PD-1 or anti-mouse PD-L1 antibodies have demonstrated anti-tumor responses as a monotherapy in murine models of squamous cell carcinoma, pancreatic carcinoma, melanoma and CRC. Blockade of the PD-1 pathway effectively promoted CD8+ T-cell infiltration into the tumor and the presence of IFN-γ, granzyme B and perforin, indicating that the mechanism of action involved local infiltration and activation of effector T-cell function in vivo (10) (11) (12) (13) (14) (15). Experiments have confirmed the in vivo efficacy of PD-1 blockade as a monotherapy as well as in combination with chemotherapy in syngeneic mouse tumor models (refer to the IB).

2.4.4 Animal pharmacology, toxicology and pharmacokinetics / pharmacodynamics

The safety of pembrolizumab was evaluated in a 1-month and a 6-month repeat-dose toxicity study in Cynomolgus monkeys administered intravenous doses of 6, 40 or 200 mg/kg once a week in the 1-month study and once every two weeks in the 6-month study, followed by a 4-month treatment-free period. No findings of toxicological significance were observed and the no observed adverse effect level (NOAEL) in both studies was ≥ 200 mg/kg, which produced exposure multiples of 19 and 94 times the exposure in humans at doses of 10 and 2 mg/kg, respectively. The exposure multiple between the NOAEL and a human dose of 200 mg was 74.

Animal reproduction studies have not been conducted with pembrolizumab. The PD-1/PD-L1 pathway is thought to be involved in maintaining tolerance to the foetus throughout pregnancy. Blockade of PD-L1 signaling has been shown in murine models of pregnancy to disrupt tolerance to the foetus and to result in an increase in foetal loss.

Animal fertility studies have not been conducted with pembrolizumab. In 1 month and 6 month repeat-dose toxicology studies in monkeys, there were no notable effects in the male and female reproductive organs; however, many animals in these studies were not sexually mature.

PK data analysis of pembrolizumab administered Q2W and Q3W showed slow systemic clearance, limited volume of distribution, and a long half-life (refer to IB). Pharmacodynamic data (IL-2 release assay) suggested that peripheral target engagement is durable (>21 days). This early PK and pharmacodynamic data provides scientific rationale for testing a Q2W and Q3W dosing schedule.

A population pharmacokinetic analysis has been performed using serum concentration time data from 476 patients. Within the resulting population PK model, clearance and volume parameters of pembrolizumab were found to be dependent on body weight. The relationship between clearance and body weight, with an allometric exponent of 0.59, is within the range observed for other antibodies and would support both body weight normalized dosing or a fixed dose across all body weights.

Pembrolizumab has been found to have a wide therapeutic range based on the melanoma indication. The differences in exposure for a 200 mg fixed dose regimen relative to a 2 mg/kg Q3W body weight based regimen are anticipated to remain well within the established exposure margins of 0.5 – 5.0 for pembrolizumab in the melanoma indication. The exposure margins are based on the notion of similar efficacy and safety in melanoma at 10 mg/kg Q3W vs. the proposed dose regimen of 2 mg/kg Q3W (i.e. 5-fold higher dose and exposure). The population PK evaluation revealed that there was no significant impact of tumour burden on exposure. In addition, exposure was similar between the NSCLC and melanoma indications. Therefore, there are no anticipated changes in exposure between different indication settings.

2.4.5 Clinical experience with Pembrolizumab

Pembrolizumab is in clinical development as an intravenous (IV) immunotherapy for advanced malignancies. Pembrolizumab is indicated for the treatment of patients across a number of indications.

In the initial clinical trial using pembrolizumab (KEYNOTE 001, KN001), 173 subjects with unresectable or metastatic melanoma with disease progression within 24 weeks of the last dose of ipilimumab or, if BRAF V600 mutation positive, prior treatment with a BRAF inhibitor, were randomized to receive pembrolizumab 2 mg/kg (n=89) or 10 mg/kg (n=84) IV once every 3 weeks until disease progression or unacceptable toxicity. The ORR was 24% in the 2 mg/kg arm, consisting of one CR and 20 PR, and similar ORR results were observed in the 10 mg/kg arm. Based on these results, in September, 2014, the United States Food and Drug Administration (FDA) granted accelerated approval to pembrolizumab for the treatment of subjects with unresectable or metastatic melanoma and disease progression following ipilimumab or, if BRAF V600 mutation-positive, a BRAF inhibitor.

Ongoing clinical trials using pembrolizumab are being conducted in advanced melanoma, non-small cell lung cancer (NSCLC), bladder cancer, hematologic malignancies, and in a number of other advanced solid tumour indications. These studies are detailed in the IB. In the KN028 trial (Clinicaltrials.gov: NCT02054806), the safety, tolerability, and antitumor activity of pembrolizumab is being assessed in subjects with a variety of malignancies, including the ten specific tumor types (Groups A-J) that will be evaluated in the trial described in this protocol. Of note, enrolment in KN028 is limited to subjects with tumor specimens with at least 1% of cells expressing PD-L1, as assessed by IHC using a prototype assay. The initial results from KN028 suggest that pembrolizumab is well tolerated in subjects with the ten specified malignancies (Groups A-J) included in the trial described in this protocol. In addition, as discussed below, the data from KN028 are being analysed to inform on the prevalence,

coexpression, and predictive value of tumor PD-L1 expression, GEP score, and MSI-H status as biomarkers predictive of pembrolizumab response.

2.5 Rationale for the proposed trial

ASTX660 has been shown to have tumour growth inhibitory activity and potent proapoptotic activity in nonclinical models, with a more balanced inhibitory profile for both compared with other first-generation Smac mimetics that are more cIAP1/2 selective (Refer to IB Section 4.1) It is therefore expected to exhibit more efficient tumour cell death by necroptosis or inflammatory apoptosis; and equally more efficiently initiate an adaptive immune response that could be augmented by combining with anti-PD-1 agent pembrolizumab. This trial will investigate more in depth the mechanisms by which ASTX660 elicits its therapeutic effects both on tumour and on the host immune system. This will be critical to determine the best strategy to pursue in future later stage tumour specific trials of IAP antagonists in combination with immunotherapy, and to ensure appropriate molecular stratification biomarkers for the greatest benefit to patients.

The 7 day on / 14 day off schedule was chosen on the basis of pharmacodynamic data from the Phase I single agent study showing that ASTX660 treatment was able to induce a robust and sustained degradation of cIAP1 and cIAP2 at doses above 180mg/day. The appropriateness of this dosing schedule will be regularly assessed on the basis of emerging PK/PD data by the SRC.

3 TRIAL DESIGN

3.1 Clinical trial objectives and endpoints

3.1.1 Primary objectives and endpoints

Primary objectives	Endpoints	Part
To propose a recommended dose for Phase II (RP2D) evaluation by establishing the maximum tolerated dose (MTD) of ASTX660 in combination with Pembrolizumab.	Determining the Maximum Tolerated Dose (MTD) based on dose-limiting toxicities (DLTs) up to 6 weeks from start of treatment (as defined in Section 3. 3 of the protocol) and the RP2D.	A
To assess the safety and tolerability of ASTX660 and Pembrolizumab when given in combination.	Determining the frequency, severity and causality of each adverse event to the combination of ASTX660 and Pembrolizumab according to the National Cancer Institute (NCI) Common Terminology Criteria for Adverse Events (CTCAE) Version 5.0.	A and B
Part B only<br>To evaluate the preliminary anti-tumour activity of ASTX660 in combination with Pembrolizumab.	Disease response by imRECIST criteria, clinical benefit rate, best changes in tumour size, progression-free survival and duration of response.	B

3.1.2 Secondary objectives and endpoints

Secondary objectives	Endpoints	Part
Part A only<br>To evaluate the preliminary anti-tumour activity of ASTX660 in combination with Pembrolizumab.	Disease response by imRECIST criteria, clinical benefit rate, best changes in tumour size, progression-free survival and duration of response.	A
To characterise the pharmacokinetics (PK) of ASTX660 in the escalation phase.	Determination of the plasma levels of ASTX660 using validated assays.	A

3.1.3 Tertiary objectives and endpoints

Tertiary objectives	Endpoints	Part
To characterise the pharmacodynamics (PD) profile of single agent ASTX660 and the combination of ASTX660 and pembrolizumab on the host immune response, during the escalation phase, using paired blood and plasma samples.	Determination of changes in immune cell population (using FACS) and host immune cell cytokine profile with single agent ASTX660 and the combination of ASTX660 and Pembrolizumab.	A
To characterise the	Determination of changes in the	A and B

Tertiary objectives	Endpoints	Part
pharmacodynamics profile of single agent ASTX660 and the combination of ASTX660 and Pembrolizumab in pre- and post-treatment tumour biopsies.	tumour microenvironment including changes in tumour infiltrating lymphocytes (TILs), myeloid derived suppressor cells (MDSCs) and other immune cells.	
To assess putative predictive biomarkers of response and resistance to the doublet combination.	Determination of changes in exploratory biomarkers including but not limited to immune transcriptome profile changes, PD-L1 expression and tumour lysate cytokine profiling in pre- and post-treatment tumour biopsies.	A and B
To evaluate the effect of ASTX660 on the tumour microenvironment including the evaluation of inflammatory cytokines downstream of NF-κB in paired tumour biopsies.	Determination of changes in exploratory biomarkers including but not limited to immune transcriptome profile changes, PD-L1 expression and tumour lysate cytokine profiling in pre- and post-treatment tumour biopsies.	A and B
To evaluate changes in patient reported outcomes from baseline using the EORTCQLQ-C30.	HRQoL will be assessed using the global score of the EORTC.	A and B
To estimate the overall survival (OS) in patients receiving the combination of pembrolizumab and ASTX660.	OS will be measured from the date of addition of ASTX660 to pembrolizumab to the date of death.	A and B

3.2 Design of the clinical trial

This is a prospective, open label, multi-centre Phase I non-randomised adaptive platform study consisting of an initial dose escalation phase followed by expansion cohorts in patients with advanced solid tumours.

Approximately 18 to ~~48~~ 68 patients with solid tumours will be entered into this trial. The final number will depend on the number of dose escalations required to reach DLT. The trial will consist of two parts: Dose Escalation (Part A) and Dose Expansion (Part B). Part B is contingent on further funding. Please refer to figure 2 for schematic of study design. Once proof of concept has been established, an additional 10 patient cohort (B4) will be opened to include patients with glioblastoma.

It is expected that the trial will have a duration of 42 months.

Emerging evidence from non-clinical models and translational data generated from this study may lead to improved hypotheses which could be tested via the addition of future exploratory cohorts in this study.

Part A: Dose Escalation

The aim of the first part of this study is to establish the maximum tolerated dose (MTD) of ASTX660 and Pembrolizumab when given in combination in patients with advanced solid tumours.

In Part A, escalating doses of ASTX660 will be investigated in combination with a fixed dose of Pembrolizumab (200mg). ASTX660 administration will follow a 7 day on / 14 day off pattern (ie dosing for 7 consecutive days leading up to administration of the immune check-point inhibitors). Therefore in Cycle 1, ASTX660 will be given alone from Day 1 to Day 7, followed by a 14 day break, then taken again in Cycle 2 . Each cycle will run for a period of 21 days, with ASTX660 taken always on Days 1 to 7 and Pembrolizumab given always on Day 8. Paired tumour biopsies and blood samples will be collected for analysis of pharmacodynamic biomarkers.

Up to 4 dose levels of ASTX660 will be explored. The dose escalation will utilize a flexible 2 stage Time to event Continual Reassessment Method (TITE-CRM) including time-to-toxicity analysis to account for dose-limiting toxicities that may arise outside the DLT period. The aim of the escalation phase will be to obtain the MTD of ASTX660 in combination with a fixed dose of Pembrolizumab associated with a dose limiting toxicity (DLT) set at a target of 25%. A target sample size of 18 evaluable patients will be recruited to Part A to enable adequate collection of paired evaluable tumour and blood samples for pharmacodynamic analyses, and this target sample size is anticipated to be sufficient to determine the MTD as per the TITE-CRM design.

Once the RP2D has been established, patients will be recruited to the expansion cohort, Part B.

Part B: Dose Expansion

Patients will be accrued to the expansion cohorts in Part B in order to further assess the safety and tolerability of the combination and to obtain a preliminary signal of efficacy. Patients in Part B will be treated at the RP2D as determined by the SRC after the initial dose escalation (Part A) has been completed. Safety, tolerability and occurrence of DLT will continually be monitored and the RP2D will be adjusted if necessary. The expansion cohorts have statistically justified sample sizes and predefined criteria to inform a three-outcome decision of go, no-go or inconclusive to guide further testing of the combinational therapy.

The treatment schedule will be the same as that for Part A, i.e. each cycle will be 21 days-long. Pembrolizumab will always be administered on D8 of every cycle. ASTX660 will be taken daily on Days 1-7.

Part B will consist of the following 10 patient exploratory tumour cohorts:

- **Cohort B1:** Patients with Immune-checkpoint refractory tumours (n = 10) including melanoma, renal cell cancer, lung cancer and head and neck cancer. Other tumour types where immune-checkpoint inhibitors are licensed as standard of care treatment can be considered upon discussion with the Chief Investigators.

  Immune-checkpoint refractory tumours are defined as having progressed within 6 months of commencement of immune checkpoint inhibitors.

- **Cohort B2:** Patients with Cervical Cancer (n = 10)

  Patients with PD-L1 positive tumours who have progressed on prior IO will go straight onto combination therapy. Patients with PD-L1 negative tumours, or those who are IO-naïve, will have a run-in with Pembrolizumab and switch to combination if they show no response or at disease progression.

- **Cohort B3:** Patients with Triple Negative Breast Cancer (n=10)

  Patients who have progressed on prior IO will go straight onto combination therapy. Patients who are IO-naïve will have a run-in with Pembrolizumab and switch to combination if they show no response or at disease progression.

- **Cohort B4:** Patients with relapsed Glioblastoma (n=10)

- **Cohort B5:** Patients with ER positive HER2 negative breast cancer (n=10)

Selected patients (minimum of 3) with tumour types of interest in each cohort (with the exception of the glioblastoma B4 cohort) will have paired tumour biopsies taken at baseline and on C3D1. Blood samples will also be collected at baseline and C2D8 for paired TCR repertoire and/or transcriptomic analysis.

Additional tumour specific indication cohorts may be added in the expansion phase if deemed of scientific value to the study.

Figure 2: Study Design

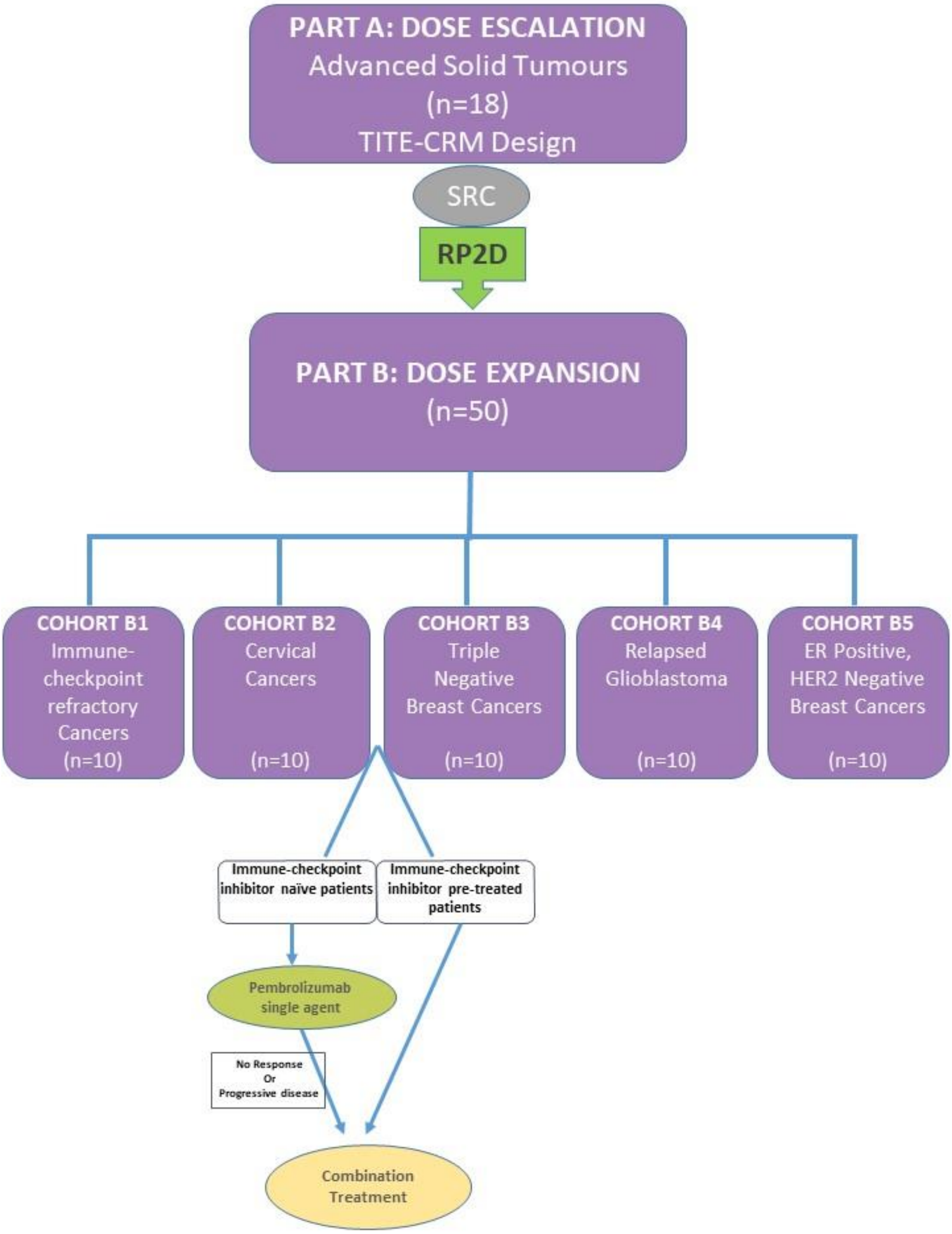

3.3 Definition of dose limiting toxicity

The dose limiting toxicity (DLT) and maximum tolerated dose (MTD) are defined using the National Cancer Institute (NCI) Common Terminology Criteria for Adverse Events (CTCAE) Version 5.0.

DLTs will be primarily assessed during the 6 week period consisting of Cycle 1 and Cycle 2 in Part A, but later onset toxicities will also be taken into account. A DLT is defined as any event that is at least possibly related to the study drugs (ASTX660 and/or Pembrolizumab)- and meets at least one of the following criteria:

- Neutropenia Grade 4 for five days or more *see note;
- Febrile neutropenia (fever of unknown origin without clinically or microbiologically documented infection) with Grade 3 or 4 neutropenia (absolute neutrophil count [ANC] <1.0 x 10^9/L and fever ≥38.5^{o}C);
- Infection (documented clinically or microbiologically) with Grade 3 or 4 neutropenia (ANC <1.0 x 10^9/L);
- Thrombocytopenia:: a) Asymptomatic Grade 4 for five days or more *see note, or b) >Grade 3 associated with clinically significant bleeding, or c) requiring platelet transfusion;
- Grade 4 anaemia
- Any Grade 3 (or greater) elevation of hepatic transaminases (ALT or AST) OR total bilirubin lasting >48 hours will be considered a DLT with the following exceptions:

  For patients with elevated hepatic transaminase at baseline due to documented liver metastasis (ie ≤5xULN), hepatic transaminase >8x ULN for > 48 hours will be considered a DLT.

- Any other Grade 3 (or greater) laboratory changes, with the exception of isolated laboratory changes of any grade without clinical sequelae or clinical significance that resolves to <Grade 2 within 7 days.
- Asymptomatic rises in amylase / lipase will only be considered a DLT if no improvement to <G2 following optimal supportive treatment including oral steroids.
- Rash will be considered a DLT if no improvement to <G2 following optimal supportive treatment including oral steroids.
- Any other Grade 3 (or greater) AE of clinical significance EXCLUDING:
  - Grade 3 or 4 nausea, vomiting or Grade 3 diarrhoea in patients who have not received optimal treatment with anti-emetics;
  - Alopecia of any grade
  - Grade 3 fatigue which resolves to <Grade 2 within 72 hours
  - Grade 3 rashes that resolve to < Grade 2 within 7 days with appropriate management
- Any Grade 3 (or greater) cytokine release syndrome that does not resolve to grade 2 or less within 6 hours with optimal medical management with immunosuppression (eg, corticosteroid dosing) will be considered a DLT.
- Any Grade 3 (or greater) immune-related adverse event with the exception of those that resolve to Grade 1 within 7 days of initiating corticosteroids.
- Any other toxicity that is greater than that at baseline, is clinically significant and/or unacceptable, does not respond to supportive care and results in a disruption of dosing schedule of more than 14 days.
- Any other toxicity, which in the view of the investigators is considered to be a DLT at any time.
- Death

*** Note: In the event of a Grade 4 neutropenia or Grade 4 thrombocytopenia, a full blood count must be performed at least on Day 5 after the onset of the event to determine if a DLT has occurred. Continue to monitor the patient closely until resolution to Grade 3 or less.**

Any patient in whom a DLT occurs during any cycle will have his or her treatment held until toxicity resolves to Grade 1 or better. Upon resolution, therapy may be re-initiated at the next lower dose level when clinically indicated and in consultation with the Chief Investigator.

If any ≥ Grade 3 or clinically significant toxicities are observed outside the DLT period, these will need to be discussed with the external, senior ECMC clinician in order to decide whether the toxicities are treatment-related and whether those should be incorporated within the TITE-CRM model.

Should any change be made to the grade or causality of an AE during the trial that may alter its DLT status, the Drug Development Unit (DDU) must be informed immediately as this may affect dose escalation decisions.

3.4 Definition of maximum tolerated dose and Recommended Phase 2 Dose

The MTD is defined as the dose of ASTX660 in combination with a fixed dose of Pembrolizumab with estimated DLT rate close to a target DLT rate of 25%, as recommended by the TITE-CRM model.

This dose will be determined following review of all the relevant toxicity data by the Safety Review Committee.

The recommended Phase 2 Dose (RP2D) will be either the MTD or a dose below as recommended by the SRC taking into account the toxicity profile seen as well as the emerging pharamacokinetic and pharamacodynamic data.

3.5 Patient evaluability

3.5.1 Safety

All patients receiving at least one administration of the IMP, ASTX660 or Pembrolizumab will be evaluable for safety.

3.5.2 Dose Escalation

Part A:

In order for patients in Part A to be evaluable for dose escalation decisions, they must be eligible and fulfil one of the following:

(1) experience a DLT within the DLT period.

OR

(2) have completed the DLT period or at least 2 weeks of DLT follow up and are still being observed for DLT. Patients who are still being monitored for DLT or who have come off within the DLT period for any reason other than toxicity will be included in the probability calculation with a weight equal to the proportion of the full DLT assessment period using the TITE-CRM design.

Patients who have experienced a DLT within the DLT period or have received at least 75% of the specified dose of ASTX660 (i.e. 5 doses of ASTX660) and 1 dose of Pembrolizumab during the first cycle will be evaluable for determination of MTD (initially at the end of Part A as well as the end of Part B).

If patients experience dose interruptions due to one or both IMPS such that Cycle 2 cannot commence within 2 weeks of planned initial dose, this will be considered a DLT. If dose interruptions occur due to unrelated events (e.g.COVID-19 infection) or due to disease-related events, patients will be evaluable for DLT as long as there is an adequate follow up period as per the TITE-CRM model.

Patients who are not evaluable can be replaced, in consultation with the SRC.

If patients are not able to proceed with both pre- and post-treatment tumour biopsies, on C1D1 and C1D7 respectively, for whatever reason (patient or physician decision; or progressive disease), additional patients will be recruited to achieve 18 pairs of evaluable biopsies. We will endeavor to achieve paired biopsies from all patients in Part A. If a patient is replaced for not achieving the paired biopsies, they will still be considered in dose escalation decisions, provided the right criteria are met.

Part B:

Patients who do not complete one cycle of combination treatment in Part B for reasons other than toxicity or progressive disease will be replaced.

Patients enrolled to B1, B2, B3 and B5 cohorts who are not able to proceed with both pre- and post-treatment tumour biopsies for whatever reason (patient or physician decision; or progressive disease) will be replaced such that a minimum of 3 patients in each cohort undergo paired biopsies.

3.5.3 Response

All patients who meet the eligibility criteria, receive at least two cycles of combination treatment and have a baseline assessment of disease will be evaluable for response by imRECIST. To be assigned a status of complete response (CR) or partial response (PR), changes in tumour measurements must be confirmed by repeat measurements performed no less than four weeks after the response criteria are met. To be assigned a status of stable disease (SD), follow-up measurements must have met the SD criteria at least once and at least six weeks after the initial dose of the investigational medicinal product (IMP) ASTX660 or Pembrolizumab is given.

For patients in cohorts B2 and B3 (IO naïve), the scan prior to the start of combination treatment will be used as the baseline scan.

Further details of response and evaluability criteria will be specified in the statistical analysis plan (SAP).

4 PATIENT SELECTION

4.1 Eligibility criteria

The patient must fulfil the eligibility criteria (listed in Sections 4.1.1 and 4.1.2).

4.1.1 Inclusion criteria:

1. PART A: Patients with histologically or cytologically confirmed malignant advanced solid tumours, refractory to conventional treatment, or for which no conventional therapy exists or is declined by the patient.

   PART B1: Patients with histologically or cytologically confirmed malignant advanced solid tumours (including melanoma, renal cell cancer, non-small cell lung cancer and head and neck squamous cell cancer), refractory to immune checkpoint inhibitors and for which no conventional therapy exists or is declined by the patient. Patients with other tumour types where immune checkpoint inhibitors are licensed can be considered upon discussion with the Chief Investigator.

   PART B2: Patients with histologically or cytologically confirmed cervical cancer, refractory to conventional treatment, or for which no conventional therapy exists or is declined by the patient.

   PART B3: Patients with histologically or cytologically confirmed triple negative breast cancer, refractory to conventional treatment, or for which no conventional therapy exists or is declined by the patient.

   PART B4: Patients with histologically confirmed relapsed glioblastoma (GBM). Diagnosis of GBM will be based on WHO classification of CNS tumours, 5th edition (2021):
   IDH wild type diffuse astrocytic glioma with microvascular proliferation, OR necrosis, OR one or more of the following molecular features of GBM:
   - TERT promoter mutation,
   - EGFR gene amplification,
   - 7 gain/10 loss chromosome copy number changes.

   PART B5: Patients with histologically or cytologically confirmed ER positive HER2 negative breast cancer who have progressed on CDK4/6 inhibitor therapy.

   For Immune Checkpoint inhibitor *refractory* tumours, participants must have progressed on treatment with an anti-PD-1/L1 mAb administered either as monotherapy or in combination with other checkpoint inhibitors or other therapies. PD-1 treatment progression is defined by meeting all of the following criteria:
   a. Has received at least 2 doses of an approved anti-PD-1/PD-L1 mAb.
   b. Has demonstrated disease progression after anti-PD-1/PD-L1 as defined by RECIST v1.1. The initial evidence of PD is to be confirmed by a second assessment no less than 4 weeks from the date of the first documented disease progression, in the absence of rapid clinical progression (as defined in c below).
   c. Progressive disease has been documented within 24 weeks from commencing anti-PD-1/PD-L1 and no later than 12 weeks from the last dose of anti-PD-1/PD-L1 mAb.
      i) Progressive disease is determined according to iRECIST.
      ii) This determination is made by the investigator. Once disease progression is confirmed, the initial date of disease progression documentation will be considered the date of disease progression.

2. Parts A, B1, and B2: Measurable disease as assessed by imRECIST.

Parts B3 and B5 : Measurable disease as assessed by imRECIST OR Evaluable disease as assessed by whole body MRI.
Part B4: Measurable disease as assessed by RANO.

3. Patients in the ER positive HER2 negative cohort (cohort B5) must not have had more than two prior systemic lines of chemotherapy in the metastatic setting. There is no limitation on number of prior lines of hormonal or targeted therapies.
4. All patients with advanced solid tumours enrolled onto Part A must be willing and able to have fresh paired tissue biopsies for biomarker analysis.
5. Life expectancy of at least 12 weeks.
6. World Health Organisation (WHO) performance status of 0 or 1.
7. Haematological and biochemical indices within the ranges shown below. These measurements must be performed within one week (Day -7 to Day 1) prior to the patient's first dose of IMP.

**Laboratory Test**	**Value required**
Haemoglobin (Hb)	≥ 9.0 g/dL
Absolute neutrophil count	≥ 1.5 x 10^9/L
Lymphocyte count	>0.5 x 10^9/L
Platelet count	≥ 100 x 10^9/L
Total Serum bilirubin	≤ 1.5 x upper limit of normal (ULN)
Alanine aminotransferase (ALT)	≤ 2.5 x (ULN) unless raised due to known metastatic liver disease in which case ≤ 5 x ULN is permissible
Aspartate aminotransferase (AST)	≤ 2.5 x (ULN) unless raised due to to known metastatic liver disease in which case ≤ 5 x ULN is permissible
Either:<br>Calculated creatinine clearance<br><br>Or:<br>Creatinine	 ≥ 50 mL/min (uncorrected value)<br><br>Or<br>< 1.5 x upper limit of normal (ULN)
Albumin	>28 g/L
LDH	<3 x ULN
Amylase	≤ ULN
Lipase	≤ ULN
Coagulation	INR <1.5<br>APTT <1.5 x ULN

8. 18 years or over
9. Written (signed and dated) informed consent and be capable of co-operating with treatment and follow-up
10. Female patients with reproductive potential must have a negative urine or serum pregnancy test performed within 72 hours of first dose

4.1.2 Exclusion criteria:

1. Radiotherapy (except for palliative reasons), endocrine therapy, immunotherapy including Pembrolizumab or chemotherapy during the previous four weeks (six weeks for nitrosoureas, Mitomycin-C) and four weeks for investigational medicinal products, except for hormonal therapy with luteinizing hormone-releasing hormone (LHRH) analogues for medical castration in patients with castrate resistant prostate cancer or ovarian suppression in pre- or peri-menopausal women with endocrine-driven breast cancer, which are permitted, and bisphosphonates or RANK ligand antagonists that are permitted for the management of bone metastases.
2. Current malignancies of other types, with the exception of adequately treated cone biopsied in situ carcinoma of the cervix uteri and basal or squamous cell carcinoma of the skin. Cancer survivors, who have undergone potentially curative therapy for a prior malignancy, have no evidence of that disease for five years or more and are deemed at negligible risk for recurrence, are eligible for the trial.
3. Ongoing Grade 2 or greater toxicities from pre-existing conditions or previous treatments. Exceptions to this are alopecia and ongoing anticoagulation therapy due to prior thromboembolic episodes.
4. Ability to become pregnant (or already pregnant or lactating). However, those female patients who have a negative serum or urine pregnancy test before enrolment and agree to use two highly effective forms of contraception (oral, injected or implanted hormonal contraception and condom, have an intra-uterine device and condom, diaphragm with spermicidal gel and condom) for four weeks before entering the trial, during the trial and for six months afterwards are considered eligible. NB. Abstinence is only considered to be an acceptable method of contraception when this is in line with the preferred and usual lifestyle of the subject. Periodic abstinence (e.g., calendar, ovulation, symptothermal, post-ovulation methods) and withdrawal are not acceptable methods of contraception
5. Male patients with partners of child-bearing potential (unless they agree to take measures not to father children by using one form of highly effective contraception [condom plus spermicide] and not to donate sperm during the trial and for six months afterwards). Men with pregnant or lactating partners should be advised to use barrier method contraception (for example, condom plus spermicidal gel) to prevent exposure to the foetus or neonate.
6. For cohorts A, B1, B2, B3 and B5 only - Known untreated or active central nervous system (CNS) metastases (progressing or requiring corticosteroids for symptomatic control). Patients with a history of treated CNS metastases are eligible, provided they meet all of the following criteria:
   - Evaluable or measurable disease outside the CNS is present.
   - Radiographic demonstration of improvement or stability upon the completion of CNS-directed therapy at least 4 weeks after completion of CNS directed therapy and no evidence of interim progression between the completion of CNS-directed therapy and the baseline disease assessment.
   - Not requiring corticosteroids.
7. For Cohort B4 only:

   - Participants with infratentorial tumors and tumors primarily located in or close to critical structures (e.g., brain stem). Patients with leptomeningeal disease.
   - Patients with evidence of raised intracranial pressure or mass effect on imaging
   - Patients unable to tolerate contrast enhanced MRI.
8. Major surgery within four weeks of the first dose of study treatment.
9. History of malabsorption syndrome or other condition that would interfere with enteral absorption.
10. At high medical risk because of non-malignant systemic disease including active uncontrolled infection.

11. History of (non-infectious) pneumonitis/interstitial lung disease that required steroids, or current pneumonitis/interstitial lung disease.

12. Known to be serologically positive for hepatitis B, hepatitis C or human immunodeficiency virus (HIV).
13. Has an active autoimmune disease that has required systemic treatment in past 3 months (i.e. with use of disease modifying agents, corticosteroids or immunosuppressive drugs). Replacement therapy (e.g., thyroxine, insulin, or physiologic corticosteroid replacement therapy for adrenal or pituitary insufficiency, etc.) is not considered a form of systemic treatment. Patients with Sjogren’s syndrome will not be excluded from the study. In addition, patients that experienced a Grade 3 or higher immune-related AE on treatment with immunotherapy will be excluded from the study. Patients that have experience a prior G2 immune-related AE on treatment will need case-by-case discussion with the CI. Patients with inactive autoimmune disease which has previously required systemic therapy, may be considered on a case-by-case basis after discussion with the sponsor.
14. Has a diagnosis of immunodeficiency or is receiving systemic steroid therapy or any other form of immunosuppressive therapy within 14 days prior to the first dose of trial treatment. The use of physiologic doses of corticosteroids may be approved after consultation with the chief Investigator. Stable use (i.e., no change in dose within 1 month prior to Day 1 of Cycle 1) of inhaled corticosteroids is allowed. Patients for cohort B4 who are on a stable dose of corticosteroids not exceeding 2mg Dexamethasone for tumour associated oedema or symptoms will be permitted to enrol on the study.
15. Has received a live vaccine within 30 days of planned start of study therapy. Note: The killed virus vaccines used for seasonal influenza vaccines for injection are allowed; however intranasal influenza vaccines (e.g. FluMist®) are live attenuated vaccines and are not allowed.
16. Any of the following cardiac criteria:
    a) Mean resting corrected QT interval (QTcF) > 470 msec obtained from an electrocardiogram (ECG). Known congenital QT syndrome or history of torsades de pointes.
    b) Left ventricular ejection fraction of <50% on echocardiogram.
    c) Any clinically significant abnormalities in rhythm, conduction or morphology of resting ECG, e.g. complete left bundle branch block, third degree heart block. Controlled atrial fibrillation is allowed.
    d) Experience of any of the following procedures or conditions in the preceding 6 months: coronary artery bypass graft, angioplasty, vascular stent, myocardial infarction, angina pectoris, congestive heart failure New York Heart Association [NYHA Grade 2 or above], severe valvular disease, uncontrolled hypertension despite optimal therapy.
17. Prior bone marrow transplant or have had extensive radiotherapy to greater than 25% of bone marrow within eight weeks.

18. Participates or plans to participate in another interventional clinical trial, whilst taking part in this Phase I study of ASTX660 and Pembrolizumab. Participation in an observational trial would be acceptable.
19. Patients with prior exposure to an IAP antagonist (Smac mimetic) will be excluded from this study. Patients with prior exposure to immunotherapy (either CTLA-4, PD-1/PD-L1 inhibitor/cellular therapy) will be permitted to enrol as long as they did not experience any immune-adverse event toxicity while on their prior immunotherapy as described in exclusion criteria 12.
20. History or current evidence of any condition, therapy, or laboratory abnormality that might confound the results of the study, interfere with the patient’s participation for the full duration of the study, or is not in the best interest of the patient to participate, in the Investigator’s opinion.

21. Severe hypersensitivity (≥ Grade 3) to any of the IMPs and/or any of their excipients.
22. Known psychiatric or substance abuse disorders that would interfere with cooperation with the requirements of the trial.
23. History of an allogenic tissue/ solid organ transplant.

24. Symptoms of COVID-19 and/or documented COVID-19 infection.

4.2 Patient enrolment

All patients who provide informed consent including any screen failures must be entered onto the patient enrolment log provided by the DDU to the investigational site.

Before enrolling the patient in the trial, the Investigator or designated representative should confirm the patient is eligible to enter the trial. An enrolment form should be completed prior to enrolment. To enrol a patient, email the completed enrolment form (see below). A trial number and dose level allocation will be allocated. The original enrolment form should be filed within the Investigator Trial File.

To enrol a patient please email the completed eligibility and enrolment form to:<br>**asteroid@icr.ac.uk (Monday – Friday 09:00-17:00)**

5 TREATMENT

5.1 Selection of the Phase I starting dose and schedule

The starting dose of ASTX660 is 90mg orally once a day, which is the dose shown to have pharmacodynamics effects in depleting IAPs in the Phase I single agent study. ASTX660 will be given in a 7 day on/14 day off pattern, in combination with 200mg flat dose Pembrolizumab administered intravenously on D8 of each cycle. This schedule was chosen on the basis of pharmacodynamic data from the phase I single agent study showing that ASTX660 treatment was able to induce a robust and sustained degradation of cIAP1 and cIAP2 at doses above 180mg/day. The appropriateness of this dosing schedule will be regularly assessed on the basis of emerging PK/PD data by the SRC (Mita M. et al.).

Pembrolizumab will be administered at the FDA approved dose of 200mg.

5.2 Dosing schedule/treatment schedule

Part A:

One treatment cycle is 21 days, with oral daily administration of ASTX660 on days 1 to 7 and intravenous infusions of Pembrolizumab on Day 8 of every cycle. Paired blood and tumour biopsies will be taken on C1D1 and C1D7.

For DLT evaluation, the DLT period is inclusive of Cycles 1 and Cycle 2, i.e. 42 days.

Up to 4 dose levels of ASTX660 will be explored (Table 1). The dose escalation will utilize a TITE CRM method including time-to-toxicity analysis to account for dose-limiting toxicities that may occur over an extended period of 42 days. The DLT period may be extended upon advice from the SRC if emerging safety data reveal toxicities occurring later than expected. 18 patients are anticipated to be sufficient to determine the MTD as per TITE CRM design.

Table 1: Schema for dose escalation

Dose Level	ASTX660	Pembrolizumab
1	90mg	200mg
2	120mg	200mg
3	150mg	200mg
4	180mg	200mg

Once the MTD or biologically active dose has been established, patients will be recruited to the expansion cohort, Part B.

Part B:

Patients will be accrued to the expansion cohorts in Part B in order to further assess the safety and tolerability of the combination and to obtain a preliminary signal of efficacy. Patients in Part B will be treated at the RP2D as determined by the SRC during the dose escalation. More than one dose could

be explored in Part B as part of dose optimisation as per project Optimus (27) if recommended by the SRC to further evaluate safety and tolerability at a given dose.

The treatment schedule will be the same as that for Part A, i.e. each cycle will be 21 days-long. Pembrolizumab will always be administered on D8 of every cycle. ASTX660 will be taken daily on Days 1-7.

Part B will consist of the following 10 patient exploratory tumour cohorts:

- **Cohort B1:** Patients with Immune-checkpoint refractory tumours (n = 10), including melanoma, renal cell cancer, lung cancer and head and neck cancer. Other tumour types where immune-checkpoint inhibitors are licenced as standard of care treatment can be considered upon discussion with the Chief Investigators.

Immune-checkpoint refractory tumours are defined as having progressed within 6 months of commencement of immune-checkpoint inhibitors.

- **Cohort B2:** Patients with Cervical Cancer (n = 10)

  Patients with PD-L1 positive tumours who have progressed on prior IO will go straight onto combination therapy. Patients with PD-L1 negative tumours, or those who are IO-naïve will have a run-in with Pembrolizumab and switch to combination if they show no response or at disease progression.

- **Cohort B3:** Patients with Triple Negative Breast Cancer (n=10)

  Patients who have progressed on prior IO will go straight onto combination therapy. Patients who are IO-naïve will have a run-in with Pembrolizumab and switch to combination if they show no response or at disease progression.

- **Cohort B4:** Patients with relapsed Glioblastoma (n=10)
- **Cohort B5:** Patients with ER positive HER2 negative breast cancer (n=10) who have progressed on CDK4/6 inhibitor therapy

5.3 Safety Review Committee

The escalation phase of the study will be split into two stages:

<u>Stage One:</u> Patients will be recruited in cohorts of two with a starting dose at Dose level 1. If no DLT occurs, escalation to the next higher dose may take place once each patient has at least 3 weeks of DLT assessment period. If we observe no DLT for the first 8 patients (at Dose levels 1, 2, 3 and 4), subsequent patients will be recruited continuously at the highest dose (Dose level 4). Once the first DLT occurs, stage one ends and we will proceed to the second stage, where the TITE-CRM model, as described in section 5.4, will be used to recommend the best dose for subsequent patients with all the information accumulated so far.

<u>Stage Two:</u> The TITE-CRM model will be updated in line with set monthly SRC meetings if no further DLTs have occurred since the previous update. If a patient experiences a DLT, this will trigger an earlier SRC meeting, and the model will be updated to allow for potential dose de-escalation. An SRC meeting may be cancelled if, since the previous model update, no DLTs have occurred and there has been no further patient follow-up within the 6-week DLT assessment period (see figure 3).

The SRC will consist of:

- Chief Investigator (CI) (or delegated DDU consultant)
- Principal Investigators (PI) from each participating site (or delegated clinician)
- DDU Clinical Trials Manager (CTM) managing the study
- Trial Statistician(s)
- Observer from the Pharmaceutical Company providing the IMP (optional)
- DDU Pharmacovigilance Officer
- External, senior ECMC clinician who is independent of the study team

Further internal or external experts may be consulted by the SRC as necessary.

The SRC will review and assess all DLT and available safety data, the TITE-CRM's recommended dose, together with available pharmacokinetic and pharmacodynamic data to make a decision on the appropriate dose for the next cohort of patients. Any dose interruptions and reductions will be taken into account.

The decision may be to:

- Proceed with dose escalation
- Expand the cohort to further explore that dose level (to a maximum of 6 evaluable patients)
- De-escalate the dose: to a previous lower dose level (to a maximum of 6 evaluable patients)
- Explore different dosing schedules
- Stop the dose escalation phase of the study
- De-escalate the dose during the dose expansion

The decisions and decision-making of the SRC on the next dose level will be documented and provided to the investigators prior to dosing any new patients.

During the expansion phase, the SRC will review accumulated data and the model updates from the TITE-CRM (which will be conducted approximately after every 5 to10evaluable patients).

A schema illustrating the expected trial flow for model updates and SRC meetings in both stage one and stage two is provided in Figure 3.

Figure 3: TITE-CRM model updates and SRC schema

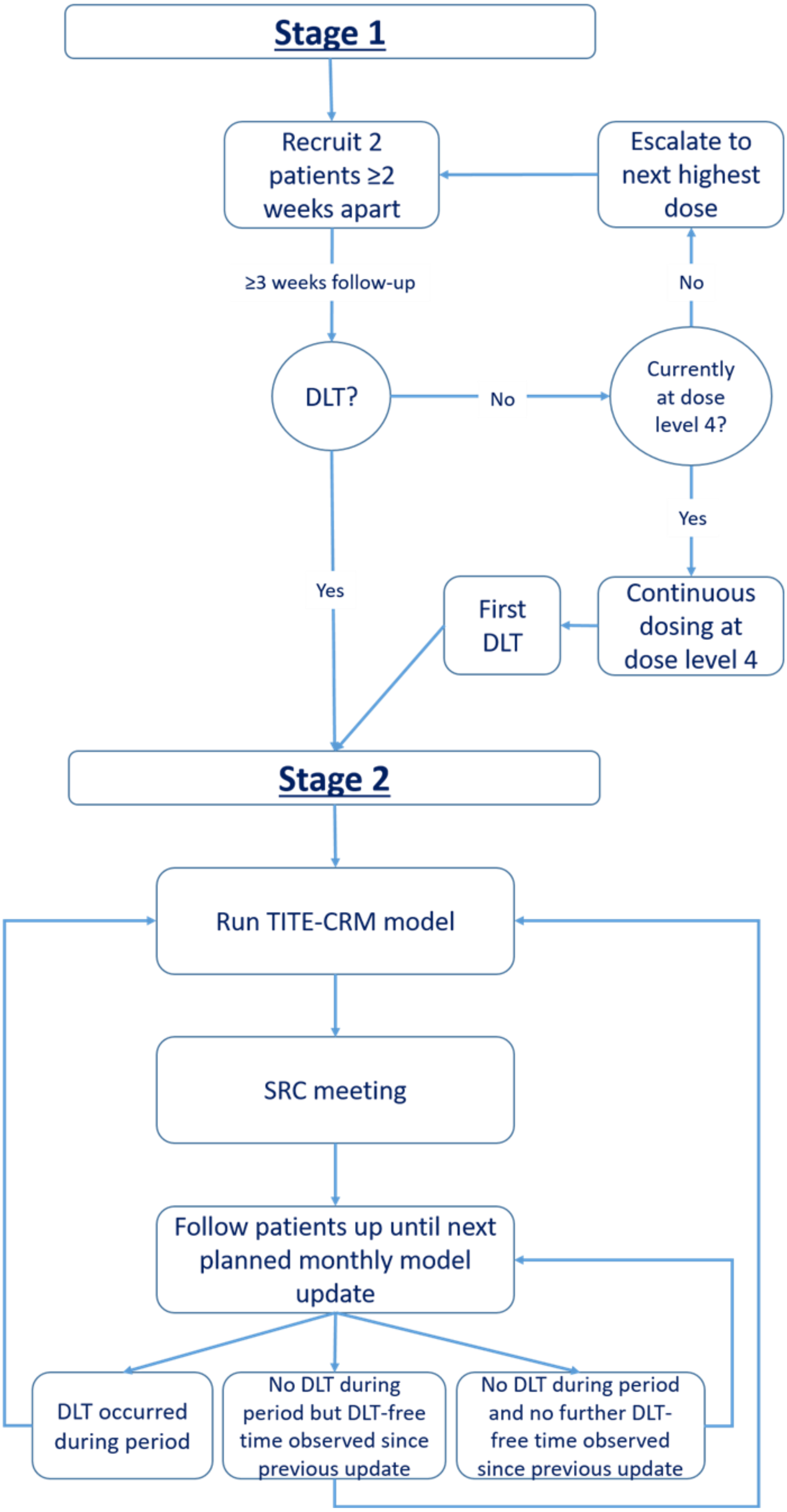

5.4 Dose escalation scheme

Up to 4 dose levels of ASTX660 will be explored. The dose escalation will utilize a flexible 2-stage Time to event Continual Reassessment Method (TITE-CRM) design to obtain the MTD of ASTX660 in combination with a fixed dose of pembrolizumab. The TITE-CRM is more time-efficient than the CRM or rule-based designs such as the 3+3 and hence allow a shorter overall trial duration. In addition, it has comparable accuracy in MTD determination as the CRM and both designs are much superior compared to rule-based designs.

The TITE-CRM design uses a weighted measure based on DLT follow-up period, and has the attractive feature of not requiring (or minimising) enrolment suspension while patients are still being observed for DLT (Cheung & Chappell 2000). It is particularly useful to account for potential late onset toxicities where the DLT may occur beyond the typical 21-28 days cycle period. This design not only allows for a full DLT assessment period (like the CRM) but it also allows the flexibility of dose decisions once patients have been followed-up for a minimum assessment period. In the TITE-CRM paradigm, patients who have started treatment but have not experienced DLT will be included in the probability calculation with a weigh equal to the proportion of the full DLT assessment period. This feature is applicable for this trial, where we would like to capture DLT occurrence up to 6 weeks from start of treatment. For instance, a patient who has not experienced DLT at 4 weeks will be included in the model as having a weight of 4/6 of no DLT. Patients who experience DLT or completed the 6-week DLT assessment period without DLT will be assigned full weight. A further advantage of this design is that it will be able to include partial information on patients who could not be formally evaluated for DLTs due to withdrawal, treatment discontinuation or death that is unrelated to treatment within the DLT assessment period.

In the dose escalation phase, the initial MTD will be obtained using up to 18 evaluable patients for dose escalation (as defined in Section 3.5.2). Once the RP2D is determined, this is followed by dose expansion in 5 specific cohorts (see below). Safety will continually be monitored and the RP2D may be adjusted if necessary.

The starting dose of ASTX660 is 90mg orally once a day, which is the dose shown to have pharmacodynamic effects in depleting IAPs in the Phase I single agent study. ASTX660 will be given in a 7 day on / 14day off pattern in combination with 200mg flat dose of Pembrolizumab administered intravenously on Day 8 of each cycle.

The starting dose of Pembrolizumab is the FDA approved dose of 200mg.

In the first stage of the TITE-CRM design, patients will be recruited in cohorts of two with a starting dose at Dose level 1. If no DLT occurs, escalation to the next higher dose may take place once each patient has at least 3 weeks of DLT assessment period. If we observe no DLT for the first 8 patients (at Dose levels 1, 2, 3 and 4), subsequent patients will be recruited continuously at the highest dose (Dose level 4). Once the first DLT occurs, we will proceed to the second stage, where the TITE-CRM model, as described in section 5.4, will be used to recommend the best dose for subsequent patients with all the information accumulated so far. A minimum DLT follow-up of 2 weeks will be imposed between patients, but with an expected recruitment rate of 1 patient every 3 weeks, we are expecting recruitment to be almost continuous in this second stage.

Once the RP2D has been established, patients will be recruited to the expansion cohort, Part B.

5.4.1 Intra-patient dose escalations

Intra-patient dose escalation for Part A is not allowed. The SRC may recommend intra-patient dose escalations in Part B, but not exceeding the MTD.

5.5 Dose expansion scheme

A Bayesian decision framework with a three-outcome design (No Go, Inconclusive and Go) based on the primary activity outcome measure of clinical benefit , which includes Complete Response, Partial Response assessed by imRECIST or durable stable disease (of 6 months or longer) will be used. Based on previous trials using single agent of IO (including Pembrolizumab), the clinical benefit rate is expected to be <5% for the patient population to be recruited into this trial (cohorts B1-B4).

Firstly, for patients with immune-checkpoint inhibitor refractory patients (cohort B1), outcome data with salvage immunotherapy is scarce; the only published data in melanoma which typifies an immune-responsive cancer, suggests a response rate to salvage immunotherapy in melanoma patients who had progressed on immune check-point inhibitors was only 10% (16). As such it is not expected that this is likely to be much lower in other tumour types.

Secondly, considering patients with cervical cancer, the response rate to patients with cervical cancer unselected for histological subtype or PD-L1 status is as yet unclear given the trials specifically selected only for squamous cell subtype and PD-L1 expression but it expected to be around 10% (17); cohort B2 will specifically explore outcomes in patients with cervical cancer who had progressed on immune-checkpoint inhibitor therapy – patients who had progressed on immunotherapy would be recruited directly onto combination therapy, and patients who were immunotherapy naïve would have single agent Pembrolizumab and only progress onto combination when deemed not to have a benefit on single agent. Outcome measures will be measured from time of start of combination to clearly answer this question.

Similarly, for patients with TNBC recruited to cohort B3 where there is a single digit response rate to single agent immunotherapy, (18, 19), patients who had progressed on prior immunotherapy would be recruited directly onto combination while patients who were immunotherapy naïve would have single agent Pembrolizumab and only progress onto combination when deemed not to have benefited from single agent.

For cohorts B4 and B5, given the very low response rates to single agent immunotherapy (20), patients will be recruited directly onto combination therapy.

The decision criteria will be based on the posterior probability that the clinical benefit rate in the combination therapy is greater than 5%:

(A) A high probability (≥90%) that the clinical benefit rate is greater than 5% will indicate that there is a clear signal that the treatment is active → GO

or

(B) A low probability (≤12%) will provide a clear signal that the treatment is not active → NO GO

or otherwise

(C) There is no clear evidence (12% < Prob < 90%) to conclude if it is active or not active → INCONCLUSIVE

This is analogous to the three-outcome design in the frequentist settings for Phase II single arm clinical trials proposed by Sargent et al 2001 (21), and randomised Phase II screened selection design by Yap et al 2013 (22). We will however explore this in a Bayesian framework here, which is particularly useful as the sample size in each expansion cohort is small and is not fixed (ranging from 10 to 15 patients). The exact number is dependent on the number of evaluable patients dosed at the RP2D in the dose-escalation phase. Those patients will contribute to the activity evaluation in the expansion phase. Simulation results indicate a favourable performance of this design even with a small sample size of n=10

5.6 Dose modifications

In the event of significant drug-related toxicity, ASTX660 or pembrolizumab dosing may be interrupted or delayed and/or reduced, as described below. In the event of multiple toxicities, dose modification should be based on the worst toxicity observed. Patients are to be instructed to notify investigators at the first occurrence of any adverse sign or symptom.

Dose modifications may occur in three ways:

- Within a cycle: dosing interruption until adequate recovery followed by dose reduction, if required;

- Between cycles: next cycle administration may be delayed due to persisting toxicity when a new cycle is due to start;

- At start of the new cycle: dose reduction may be required in a subsequent cycle based on toxicity experienced in the previous cycle.

If despite appropriate dose reductions and/or delays, the patient experiences unacceptable toxicities specifically attributed to either ASTX660 or pembrolizumab, then the patient may continue with single agent treatment without coming off study providing they have completed their first cycle with combination treatment.

5.6.1 Dose reductions

There will be no dose reductions for pembrolizumab in this study. However, study treatment may be interrupted as described in section 5.6.2 below.

A dose reduction (one dose level below) of ASTX660 is recommended if the patient reports Grade ≥ 3 toxicity probably related to ASTX660 or Grade 2 toxicity lasting >3 weeks despite appropriate supportive treatment. This does not apply to clinically insignificant laboratory abnormalities. For the management of asymptomatic pancreatic enzyme elevation, please refer to the table in section 5.7.2 under pancreatic events.

Once the patient is re-treated after recovery, if the same Grade ≥ 3 toxicity occurs at the new reduced dose level, further dose reduction of ASTX660 should be considered. The level of dose reduction will determined by the Chief Investigator and the SRC members.

5.6.2 Dose delays

Adverse events possibly due to pembrolizumab and/or ASTX660 may be managed independently according to their respective risk profile. They may be temporarily suspended in patients experiencing toxicity considered to be related to the respective study drug.

Adverse events (both non-serious and serious) associated with pembrolizumab exposure may represent an immunologic aetiology. These adverse events may occur shortly after the first dose or several months after the last dose of treatment. See Section 5.7 for further guidelines for management of specific toxicity, including use of corticosteroids.

For pembrolizumab-related toxicities, if corticosteroids have been initiated, they must be tapered over ≥ 1 month to ≤ 10 mg/day oral prednisone or equivalent before pembrolizumab can be resumed. Pembrolizumab may be withheld for a period of time of up to 12 weeks to allow for corticosteroids to be reduced to ≤ 10 mg/day oral prednisone or equivalent. Pembrolizumab must be permanently discontinued if the adverse event does not resolve or the corticosteroid dose is not ≤10 mg/day within 12 weeks of the last pembrolizumab treatment, unless otherwise agreed with the Chief Investigator. Resumption of pembrolizumab may be considered in patients who are deriving benefit and have fully recovered from the immune-related event. Patients can be rechallenged with pembrolizumab only after

approval has been documented by both the investigator (or an appropriate delegate) and the Chief Investigator.

For ASTX660-related toxicities, patients who miss more than 12 weeks of scheduled treatment will be discontinued from study, except for those patients who have been specifically discussed with the Chief Investigator and the DDU trial management team, and deemed would likely benefit from continuing on single agent Pembrolizumab.

Patients who develop clinically significant infections (viral, bacterial or fungal) requiring systemic anti-infective therapy or medical intervention, should interrupt both IMPs until their infection has resolved.

Dosing interruptions are permitted outside of the DLT period after completion of Cycles 1 and 2 in the case of medical / surgical events or logistical reasons not related to study therapy (e.g., elective surgery, unrelated medical events, patient vacation, and/or holidays). Subjects should be placed back on study therapy within 3 weeks of the scheduled interruption, unless otherwise discussed with the DDU. The reason for interruption should be documented in the patient's study record.

5.6.3 Dose interruptions due to COVID-19

If a patient tests positive for COVID-19 during the study, treatment will be interrupted until the patient becomes asymptomatic, has tested negative for the virus and it is deemed safe for them to continue on study. During treatment interruption the patient will be followed up regularly by phone.

5.7 Toxicity management guidelines

As there is some overlapping toxicity (in particular rash, raised amylase/lipase and pancreatitis), establishing causality is important in guiding management. The following sections describe supporting care guidelines for pembrolizumab-related toxicity (5.7.1) and toxicity related to both ASTX660 and pembrolizumab (5.7.2).

Pembrolizumab treatment may be temporarily suspended in patients experiencing toxicity considered to be related to study treatment. If corticosteroids are initiated for treatment of the toxicity, they must be tapered over ≥ 1 month to ≤ 10 mg/day oral prednisone or equivalent before pembrolizumab can be resumed. If pembrolizumab is withheld for > 12 weeks, the patient will be discontinued from pembrolizumab. However, pembrolizumab may be withheld for > 12 weeks to allow for patients to taper off corticosteroids prior to resuming treatment. Pembrolizumab can be resumed after being withheld for > 12 weeks if the Chief Investigator agrees that the patient is likely to derive clinical benefit. Pembrolizumab treatment may be suspended for reasons other than toxicity (e.g., surgical procedures) with the Chief Investigator’s approval. The investigator and the Chief Investigator will determine the acceptable length of treatment interruption.

ASTX660 treatment may be temporarily suspended in patients experiencing toxicity considered to be related to study treatment. If corticosteroids are initiated for treatment of the toxicity, they must be tapered to ≤ 10 mg/day oral prednisone or equivalent before ASTX660 can be resumed, if clinically appropriate. If ASTX660 is withheld for > 28 days, the patient will be discontinued from ASTX660. If the investigator believes the patient is likely to derive clinical benefit and the Chief Investigator is in agreement, ASTX660 treatment can be resumed after being withheld for > 28 days.

If either pembrolizumab or ASTX660 is discontinued, the other drug can be continued if the patient is likely to derive clinical benefit, as determined by the investigator and following discussion with the Chief Investigator.

5.7.1 Management of pembrolizumab-related toxicity

Subjects should receive appropriate supportive care measures as deemed necessary by the treating investigator. Suggested supportive care measures for the management of adverse events with potential immunologic aetiology are outlined below. Where appropriate, these guidelines include the use of oral or intravenous treatment with corticosteroids as well as additional anti-inflammatory agents if symptoms do not improve with administration of corticosteroids. Note that several courses of steroid tapering may be necessary as symptoms may worsen when the steroid dose is decreased. For each disorder, attempts should be made to rule out other causes such as metastatic disease or bacterial or viral infection, which might require additional supportive care. The treatment guidelines are intended to be applied when the investigator determines the events to be related to pembrolizumab.

Note: if after the evaluation the event is determined not to be related, the investigator does not need to follow the treatment guidance (as outlined below). Refer to Section 5.6 for dose modification.
It may be necessary to perform conditional procedures such as bronchoscopy, endoscopy, or skin photography as part of evaluation of the event.

General Instructions:

1. Severe and life-threatening immune-related AEs should be treated with IV corticosteroids followed by oral steroids. Other immunosuppressive treatment should begin if the AEs are not controlled by corticosteroids.
2. The corticosteroid taper should begin when the irAE is ≤ Grade 1 and continue at least 4 weeks.
3. If pembrolizumab has been withheld, pembrolizumab may resume after the AE decreased to ≤ Grade 1 after corticosteroid taper.

5.7.1.1 Management of Diarrhoea / Colitis

Subjects should be carefully monitored for signs and symptoms of enterocolitis (such as diarrhoea, abdominal pain, blood or mucus in stool, with or without fever) and of bowel perforation (such as peritoneal signs and ileus).

CTCAE v5.0 Grade	Recommended Actions
Grade 2 or 3	• Withhold Pembrolizumab<br>• Monitor patients for signs and symptoms of enterocolitis (ie, diarrhoea, abdominal pain, blood or mucus in stool with or without fever) and of bowel perforation (ie, peritoneal signs and ileus)
Recurrent Grade 2 or Grade 3	• Permanently discontinue Pembrolizumab<br>• Administer corticosteroids (initial dose of 1-2 mg/kg prednisone or equivalent) followed by taper<br>• Patients with ≥Grade 2 diarrhoea suspecting colitis should consider GI consultation and performing endoscopy to rule out colitis<br>• Patients with diarrhoea/colitis should be advised to drink liberal quantities of clear fluids. If sufficient oral fluid intake is not feasible, fluid and electrolytes should be substituted via IV infusion

5.7.1.2 Management of Type 1 diabetes mellitus (T1DM) or Hyperglycaemia

CTCAE v5.0 Grade	Recommended Actions
New onset T1DM or<br>Grade 3 or 4 hyperglycaemia associated with evidence of β cell failure	• Withhold^ Pembrolizumab<br>• Initiate insulin replacement therapy for patients with T1DM<br>• Administer anti-hyperglycaemic in patients with hyperglycaemia<br>• Monitor participants for hyperglycaemia or other signs and symptoms of diabetes

^ The decision to withhold or permanently discontinue pembrolizumab is at the discretion of the investigator or treating physician. If control achieved or ≤ Grade 2, pembrolizumab may be resumed.

5.7.1.3 Management of Hypophysitis

CTCAE v5.0 Grade	Recommended Actions
Grade 2	• Withhold Pembrolizumab<br>• Monitor for signs and symptoms of hypophysitis (including hypopituitarism and adrenal insufficiency)
Grade 3 or 4	• Withhold or permanently discontinue Pembrolizumab<br>• Administer corticosteroids and initiate hormonal replacements as clinically indicated

5.7.1.4 Management of Hyperthyroidism and Hypothyroidism

CTCAE v5.0 Grade	Recommended Actions
Hyperthyroidism Grade 2	• Continue Pembrolizumab<br>• Treat with non-selective beta-blockers (eg, propranolol) or thionamides as appropriate<br>• Monitor for signs and symptoms of thyroid disorders
Hyperthyroidism Grade 3 or 4	• Withhold or permanently discontinue Pembrolizumab^<br>• Treat with non-selective beta-blockers (eg, propranolol) or thionamides as appropriate<br>• Monitor for signs and symptoms of thyroid disorders
Hypothyroidism Grade 2, 3 or 4	• Continue Pembrolizumab<br>• Initiate thyroid replacement hormones (eg, levothyroxine or liothyronine) per standard of care<br>• Monitor for signs and symptoms of thyroid disorders

^The decision to withhold or permanently discontinue pembrolizumab is at the discretion of the investigator or treating physician. If control achieved or ≤ Grade 2, pembrolizumab may be resumed.

5.7.1.5 Management of Hepatic Events

**CTCAE v5.0 Grade**	**Recommended Actions**
Grade 2^ AST or ALT elevation or Increased Bilirubin	• Withhold Pembrolizumab<br>• Administer corticosteroids (initial dose of 0.5 - 1 mg/kg prednisone or equivalent) followed by taper<br>• Monitor with liver function tests (consider weekly or more frequently until liver enzyme value returned to baseline or is stable)
Grade 3^^ or 4^^^ AST or ALT elevation or Increased Bilirubin	• Permanently discontinue Pembrolizumab<br>• Administer corticosteroids (initial dose of 1 - 2 mg/kg prednisone or equivalent) followed by taper<br>• Monitor with liver function tests (consider weekly or more frequently until liver enzyme value returned to baseline or is stable)

^AST/ALT: >3.0 - 5.0 x ULN if baseline normal; >3.0 5.0 x baseline, if baseline abnormal; bilirubin:>1.5 3.0 x ULN if baseline normal; >1.5 - 3.0 x baseline if baseline abnormal

^^AST/ALT: >5.0 to 20.0 x ULN, if baseline normal; >5.0 20.0 x baseline, if baseline abnormal; bilirubin:>3.0 10.0 x ULN if baseline normal; >3.0 - 10.0 x baseline if baseline abnormal

^^^AST/ALT: >20.0 x ULN, if baseline normal; >20.0 x baseline, if baseline abnormal; bilirubin: >10.0 x ULN if baseline normal; >10.0 x baseline if baseline abnormal

5.7.1.6 Management of Renal Failure or Nephritis

**CTCAE v5.0 Grade^**	**Recommended Actions**
Grade 2	• Withhold Pembrolizumab<br>• Administer corticosteroids (prednisone 1 – 2 mg/kg or equivalent) followed by taper<br>• Monitor changes of renal function
Grade 3 or 4	• Permanently discontinue Pembrolizumab<br>• Administer corticosteroids (prednisone 1 – 2 mg/kg or equivalent) followed by taper<br>• Monitor changes of renal function

^ Grading according to increased creatinine or acute kidney injury

5.7.1.7 Management of Myocarditis

**CTCAE v5.0 Grade^**	**Recommended Actions**
Grade 1 or 2	• Withhold Pembrolizumab<br>• Based on severity of AE administer corticosteroids<br>• Ensure adequate evaluation to confirm aetiology and/or exclude other causes
Grade 3 or 4	• Permanently discontinue Pembrolizumab<br>• Based on severity of AE administer corticosteroids<br>• Ensure adequate evaluation to confirm aetiology and/or exclude other causes

5.7.1.8 Management of all other Immune-Related AEs

CTCAE v5.0 Grade	Recommended Actions
Persistent Grade 2	• Withhold Pembrolizumab<br>• Based on severity of AE administer corticosteroids<br>• Ensure adequate evaluation to confirm aetiology or exclude other causes
Grade 3	• Withhold or discontinue Pembrolizumab based on the event^<br>• Based on severity of AE administer corticosteroids<br>• Ensure adequate evaluation to confirm aetiology or exclude other causes
Recurrent Grade 3 or Grade 4	• Permanently discontinue Pembrolizumab<br>• Based on severity of AE administer corticosteroids<br>• Ensure adequate evaluation to confirm aetiology or exclude other causes

^Events that require discontinuation include but are not limited to: Guillain-Barre Syndrome, encephalitis, myelitis, Stevens-Johnson Syndrome and toxic epidermal necrolysis.

5.7.2 Management guidelines for toxicity related to both ASTX660 and pembrolizumab

- **Dermatologic events**
  A dermatologist should evaluate persistent and/or severe rash or pruritus. A biopsy should be considered unless contraindicated.

  - For **Grade 1 events**, continue ASTX660 and pembrolizumab. Initiate treatment with topical corticosteroids and/or other symptomatic therapy (e.g., antihistamines). Monitor closely (eg within 48-72 hours). Consider treatment with 10mg/day oral prednisone or equivalent.
  - For **Grade 2 events**, withhold ASTX660 for up to 28 days but continue pembrolizumab. Continue symptomatic treatment eg with topical corticosteroids and/or other symptomatic therapy (e.g., antihistamines). Consider treatment with 10mg/day oral prednisone or equivalent; treatment with higher steroid dose may be necessary as clinically indicated.
    - If Grade 2 rash improves rapidly with holding ASTX660 and commencing topical treatments, and is therefore felt by the investigator to be attributable to ASTX660, dosing may be resumed at the same dose level or one dose level lower upon improvement to Grade ≤ 1. If steroid dose is ≤10 mg/day, pembrolizumab should be continued. Consider treatment with higher-potency topical corticosteroids if event does not improve
  - For **Grade 3 events**, withhold ASTX660 for up to 28 days and pembrolizumab for up to 12 weeks after event onset. Consider initiating treatment with 10 mg/day oral prednisone or equivalent, increasing dose to 1-2 mg/kg/day if event does not improve within 48-72 hours.
    - If event resolves to Grade 1 or better within 28 days, resume ASTX660 at one dose level lower. If not, permanently discontinue ASTX660. If event resolves to Grade 1 or better, resume pembrolizumab. Permanently discontinue ASTX660 for recurrent Grade ≥3 events after lowering the ASTX660 dose.
    - If event does not resolve to Grade 1 or better while withholding pembrolizumab, permanently discontinue ASTX660 and pembrolizumab and discuss with Chief Investigator.
  - For **Grade 4 events**, permanently discontinue ASTX660 and pembrolizumab and discuss with Chief Investigator. Arrange referral to dermatologist and confirmatory tests. Initiate treatment with 1-2 mg/kg/day intravenous methylprednisolone or equivalent and convert to 1-2 mg/kg/day oral prednisone or equivalent upon

improvement. If event does not improve within 48 hours after initiating corticosteroids, consider the addition of an immunosuppressive agent.

- **Pancreatic events:**
    - The differential diagnosis of acute abdominal pain should include pancreatitis. Appropriate workup should include an evaluation for ductal obstruction, as well as serum amylase and lipase tests. Patients with any elevation of amylase of lipase must be carefully monitored for any signs or symptoms that can be attributed to this (including but not limited to fatty stools, nausea, abdominal pain or bloating.
    - **For Any Grade pancreatitis**
        - Permanently discontinue ASTX660
    - **For Grade 2 or 3 immune-related pancreatitis**
        - Withhold pembrolizumab for up to 12 weeks and discontinue ASTX660.
        - Consider referral to gastrointestinal specialist.
        - Initiate treatment with 1-2 mg/kg/day intravenous methylprednisolone or equivalent and convert to 1-2 mg/kg/day oral prednisone or equivalent upon improvement.
        - If event resolves to Grade 1 or better resume pembrolizumab.
        - .
        - If event does not resolve to Grade 1 or better while withholding pembrolizumab, permanently discontinue pembrolizumab and discuss with Chief Investigator.
        - For recurrent events, permanently discontinue pembrolizumab and discuss with Chief Investigator
    - **For Grade 4 immune-related pancreatitis**
        - Permanently discontinue pembrolizumab and ASTX660 and discuss with Chief Investigator.
        - Refer patient to gastrointestinal specialist.
        - Initiate treatment with 1-2 mg/kg/day intravenous methylprednisolone or equivalent and convert to 1-2 mg/kg/day oral prednisone or equivalent upon improvement.
        - If event does not improve within 48 hours after initiating corticosteroids, consider adding an immunosuppressive agent.
        - If event resolves to Grade 1 or better, taper corticosteroids over ≥ 1 month.

Suggested guidelines regarding adjusting, withholding and discontinuing study treatment and the potential management of asymptomatic lipase and amylase enzyme elevation considered to be related to study treatment are provided in Table 2 below:

Table 2: Pancreatic Enzyme Elevation

CTCAE v5.0 Grade Lipase/Amylase Increase	Recommended Actions and Dose Modification
**Grade 1:**<br>**Lipase:** >ULN – 1.5 x ULN<br>**Amylase:** >ULN – 1.5 x ULN	Continue study treatment and monitor for change in severity
**Grade 2:**<br>**Lipase:** >1.5-2.0 x ULN and symptomatic;<br>>2.0-5.0 x ULN and asymptomatic<br><br>**Amylase:** >1.5-2.0 x ULN and symptomatic;<br>>2.0-5.0 x ULN and asymptomatic	Continue study treatment and monitor amylase and lipase at least weekly with close monitoring of any change in severity and changes in signs/symptoms<br><br>For prolonged elevation (e.g., > 3 weeks), consider treatment with 10 mg/day oral prednisone or equivalent”
**Grade 3:**<br>**Lipase:** >2.0-5.0 x ULN and symptomatic;<br>>5.0 x ULN and asymptomatic<br><br>**Amylase:** >2.0-5.0 x ULN and symptomatic;	Consider referral to gastrointestinal specialist and urgent imaging (CT scan)<br><br>Withhold pembrolizumab for up to 12 weeks and ASTX660 for up to 28 days after event onset.

>5.0 x ULN and asymptomatic<br><br>&<br><br>**Grade 4:**<br>**Lipase:** >5.0 x ULN symptomatic<br>**Amylase:** >5.0 x ULN symptomatic	Monitor amylase and lipase every other day.<br>If event resolves to Grade 1 or better with interruption of the ASTX660, this may be resumed at a lower dose level with monitoring at least twice a week[a]<br><br>If no improvement, consider treatment with 1-2 mg/kg/day oral prednisone or equivalent. If event resolves to Grade 1 or better within 28 days, ASTX660 may be resumed at a lower dose level with monitoring at least twice a week[a], pembrolizumab may be resumed upon discussion with the Chief Investigator once steroids are weaned .<br><br>If event does not resolve to Grade 1 or better within 28 days, permanently discontinue pembrolizumab and ASTX660 and discuss with Chief Investigator.<br>For recurrent events, permanently discontinue pembrolizumab and discuss with Chief Investigator.

[a] Escalation may be considered if no pancreatic enzyme elevation Grade ≥1 is evident for 4 weeks after restarting study treatment at a lower dose and with approval from the Chief Investigator.
[b] Approval of the Chief Investigator is required to restart study treatment after interruption of ≥21 days.

- **Pneumonitis:**

CTCAE v5.0 Grade	Recommended Actions
Grade 1	• Monitor for symptoms<br>• Study treatment can continue if it benefits the patient
Grade 2	• Withhold Pembrolizumab and ASTX660 until recovery to Grade ≤1<br>• Administer corticosteroids (initial dose of 1 - 2 mg/kg prednisone or equivalent) followed by taper. Steroid taper should be started and continued over no less than 4 weeks.<br>• Add prophylactic antibiotics for opportunistic infections<br>• Monitor patients for signs and symptoms of pneumonitis<br>• Evaluate patients with suspected pneumonitis with radiographic imaging and initiate corticosteroid treatment<br>• If recovery to Grade ≤1, treatment with ASTX660 may restart at one dose level lower.<br>• If not recovery to Grade ≤1 within 3 weeks, permanently discontinue study treatment
Recurrent Grade 2, Grade 3 or 4	• Permanently discontinue Pembrolizumab and ASTX660<br>• Administer corticosteroids (initial dose of 1 - 2 mg/kg prednisone or equivalent) followed by taper<br>• If IV steroids followed by high dose oral steroids does not reduce initial symptoms within 48 to 72 hours, consider additional anti-inflammatory measures with or without immunosuppression<br>• Add prophylactic antibiotics for opportunistic infections<br>• Monitor patients for signs and symptoms of pneumonitis<br>• Evaluate patients with suspected pneumonitis with radiographic imaging and initiate corticosteroid treatment

The investigator should consider the benefit-risk balance a given patient may be experiencing prior to further administration of pembrolizumab. For pembrolizumab -related toxicities, if corticosteroids have been initiated, they must be tapered over ≥ 1 month to ≤ 10 mg/day oral prednisone or equivalent before pembrolizumab can be resumed.

Pembrolizumab may be withheld for a period of time up to 12 weeks to allow for corticosteroids to be reduced to ≤ 10 mg/day oral prednisone or equivalent. Pembrolizumab must be permanently discontinued if the adverse event does not resolve or the corticosteroid dose is not ≤ 10 mg/day within 12 weeks of the last pembrolizumab treatment, unless otherwise agreed with the Chief Investigator. Resumption of pembrolizumab may be considered in patients who are deriving benefit and have fully recovered from the immune-related event. Patients can be rechallenged with pembrolizumab only after approval has been documented by both the investigator (or an appropriate delegate) and the Chief Investigator and DDU trial management team.

In addition, cytokine release syndrome or systemic immune activation is a potential risk associated with immune check point inhibitor combinations. Systemic immune activation is a rare condition characterised by an excessive immune response. Given the mechanism of action of pembrolizumab, systemic immune activation is considered a potential risk when given in combination with other immunomodulating agents. Systemic immune activation should be included in the differential diagnosis for patients who, in the absence of an alternative aetiology, develop a sepsis-like syndrome after administration of pembrolizumab. If systemic immune activation is suspected, contact the Chief Investigator for additional recommendations.

5.7.3 Management of infusion reactions

Treatment guidelines for subjects who experience an infusion reaction associated with administration of pembrolizumab are shown in the table below.

Table 3: Pembrolizumab Infusion Reaction Treatment Guidelines

**NCI CTCAE Grade**	**Treatment**	**Premedication at subsequent dosing**	**ASTX660 Action**
Grade 1:<br><br>Mild reaction; infusion interruption not indicated; intervention not indicated	Increase monitoring of vital signs as medically indicated until the subject is deemed medically stable in the opinion of the investigator.	None	Depending on dose schedule, consider re-challenge, dose delay or dose reduction.
Grade 2:<br><br>Requires infusion interruption but responds promptly to symptomatic treatment (e.g., antihistamines, NSAIDS, narcotics, IV fluids); prophylactic medications indicated for < =24 hrs	**Stop Infusion and monitor symptoms.**<br><br>Additional appropriate medical therapy may include but is not limited to:<br>IV fluids<br>Antihistamines<br>NSAIDS<br>Acetaminophen<br>Narcotics<br><br>Increase monitoring of vital signs as medically indicated until the subject is deemed medically stable in the opinion of the investigator.<br><br>If symptoms resolve within one	Subject may be premedicated 1.5h (± 30 minutes) prior to infusion of pembrolizumab with:<br><br>Diphenhydramine 50 mg PO (or equivalent dose of antihistamine).<br><br>Acetaminophen 500-1000 mg PO (or equivalent dose	Hold ASTX660 treatment and reduce next dose by 1 dose level (depending on dose schedule) for the next administration period.

	hour of stopping drug infusion, the infusion may be restarted at 50% of the original infusion rate (e.g. from 100 mL/hr to 50 mL/hr). Otherwise dosing will be held until symptoms resolve and the subject should be premedicated for the next scheduled dose.<br><br>**Subjects who develop Grade 2 toxicity despite adequate premedication should be permanently discontinued from further trial treatment administration.**	of antipyretic).	
Grade 3 & 4<br><br><u>Grade 3:</u><br>Prolonged (i.e., not rapidly responsive to symptomatic medication and/or brief interruption of infusion);<br>recurrence of symptoms following initial improvement; hospitalization indicated for other clinical sequelae (e.g., renal impairment, pulmonary infiltrates)<br><br><u>Grade 4:</u><br>Life-threatening; pressor or ventilatory support indicated	**Stop Infusion.**<br><br>Additional appropriate medical therapy may include but is not limited to:<br>IV fluids<br>Antihistamines<br>NSAIDS<br>Acetaminophen<br>Narcotics<br>Oxygen<br>Pressors<br>Corticosteroids<br>Epinephrine<br><br>Increase monitoring of vital signs as medically indicated until the subject is deemed medically stable in the opinion of the investigator.<br><br>Hospitalization may be indicated.<br><br>**Subject is permanently discontinued from further trial treatment administration.**	No subsequent dosing	Hold ASTX660 treatment during the diagnostic work-up.<br><br>In the absence of clear antitumour effect, permanently discontinue ASTX660.<br><br>In the presence of a clear antitumour effect, consider ASTX660 dose reduction, pretreatment with corticosteroids and antihistamines, and possible close observation for additional treatment cycles.
N.B. Appropriate resuscitation equipment should be available in the room and a physician readily available during the period of drug administration.			

MRI = magnetic resonance imaging; TSH = thyroid-stimulating hormone

5.8 Duration of treatment

Treatment should continue unless (a) the patient asks to be withdrawn, (b) there is evidence of disease progression or (c) the patient is experiencing unacceptable toxicity or for any of the reasons listed in

Section 11. Treatment with Pembrolizumab can continue for up to 2 years or 35 cycles. Treatment with ASTX660 can continue as long as the patient derives clinical benefit.

5.8.1 Replacement of patients

Patients who do not complete the DLT period in Part A, for reasons other than toxicity will be replaced.

5.9 Concomitant medication and treatment

Concomitant medication may be given as medically indicated. Details (including doses, frequency, route and start and stop dates) of the concomitant medication given must be recorded in the patient's medical records and the case report form (CRF).

Radiotherapy may be given concomitantly for the control of bone pain, however these irradiated lesions will not be evaluable for response.

The patient must not receive other anti-cancer therapy or investigational drugs while on the trial.

Careful consideration should be given to balancing the medical needs of the subject and the potential effect on ASTX660 before coadministering a drug that is known to be a potent CYP3A4 inhibitor or inducer. Potent inhibitors or inducers of CYP3A should be avoided within 2 weeks before the first dose of study treatment (3 weeks for St John's Wort and 5 weeks for phenobarbitone), and from the time of consent until 2 weeks after the last dose of study treatment. Chronic use of a strong CYP3A4 inhibitor or inducer, or sensitive CYP3A substrates with a narrow therapeutic window that are deemed not permissible by the Chief Investigator after enrolment should be avoided or be used with caution. Moderate inhibitors or inducers of CYP3A should be used with caution. Examples of CYP3A4 inhibitors and inducers may be found on the following websites; note that the published lists are not comprehensive. Refer to the specific product information for an intended concomitant drug for its effect on CYP3A4.

- Pharmacytimes.com: http://www.pharmacytimes.com/publications/issue/2008/ 2008-09/2008-09-8687#sthash.P1Lojc8O.dpuf.
- FDA.gov: http://www.fda.gov/drugs/developmentapprovalprocess/developmentresources/druginteractionslabeling/ucm093664.htm

Due to the risk of potential cIAP-related liver injury, statins and other medications that can affect liver function should be used with caution.

Medications known to increase risk of torsades des pointes are prohibited in this study. A list of such medications is provided at the following website and discussed in Drew et al 2010: https://www.crediblemeds.org/

As ASTX660 is an investigational drug and phototoxicity has not been completely ruled out, patients will be advised to use broad-spectrum sunscreen before exposure to the sun and to avoid excessive sun exposure.

Live vaccines within 30 days prior to the first dose of trial treatment and while participating in the trial. Examples of live vaccines include, but are not limited to, the following: measles, mumps, rubella, chicken pox, yellow fever, rabies, BCG, and typhoid (oral) vaccine. Seasonal influenza vaccines for injection are generally killed virus vaccines and are allowed; however intranasal influenza vaccines (e.g. Flu-Mist®) are live attenuated vaccines, and are not allowed.

Patients can receive the COVID-19 vaccine prior to screening or after the first two cycles of combination treatment, due to the unknown interactions between the vaccine and Pembrolizumab & ASTX660. If patients wish to be vaccinated before completing the first two cycles, this should be

discussed and agreed with the Chief Investigator. The schedule for continuing with trial treatment should be reviewed accordingly and filenoted. The COVID-19 vaccination can be considered outside of these times by the Principal Investigator following careful discussion and on a case-by-case basis. A file note is required to document any treatment breaks that are related to the COVID-19 vaccinations. No formal studies have been done on the effectiveness of vaccines while patients have been treated with Pembrolizumab or ASTX660. Site must clearly document in the medical notes that patients have been aware there is no data about the safety of trial medication during a COVID vaccination and any effects of the trial medication on the efficacy of the vaccine. This applies for all licensed COVID vaccines and to each dose of vaccine individually.

Glucocorticoids for any purpose other than to modulate symptoms from an event of clinical interest of suspected immunologic aetiology. The use of physiologic doses of corticosteroids may be approved after consultation with the Sponsor. Patients with glioblastoma recruited to cohort B4 will be permitted to receive a stable low dose steroids for symptom control as defined in section 4.1.2.

Subjects who, in the assessment by the investigator, require the use of any of the aforementioned treatments for clinical management should be removed from the trial. Subjects may receive other medications that the investigator deems to be medically necessary.

6 PHARMACEUTICAL INFORMATION

6.1 Supply of ASTX660 and pembrolizumab

A complete certificate of analysis and a Qualified Person (QP) certification must be provided with each batch of IMP and be retained in the Investigator Trial File (ITF)/Pharmacy File.

All study drug will be packaged and labelled in accordance with local regulations and Good Manufacturing Practice, stating that the drug is for clinical trial use only and should be kept out of the reach and sight of children. ASTX660 will be labelled, QP released and distributed by Astex Pharmaceuticals. Pembrolizumab will be labelled, QP released and distributed by MSD Sharpe and Dohme Limited.

ASTX660 and Pembrolizumab will only be dispatched to sites after receipt of confirmation that the regulatory checklist is complete.

For information on all IMP and re-ordering of supplies, contact the DDU Clinical Trials Manager responsible for the trial who will arrange further supplies.

6.2 Pharmaceutical data

6.2.1 Formulation of ASTX660

ASTX660 will be supplied as capsules. Each hydroxylpropyl methylcellulose (HPMC) capsule contains either 30 mg or 90 mg of ASTX600 free-base equivalents, suitable for oral administration. Capsules can be swallowed with water. The capsules are provided in induction-sealed bottles with child-resistant closures. Bottles are labelled with the capsule strength (ie, 30 mg or 90 mg), and are further distinguished by different-colored labels. Each bottle contains 14 capsules and an oxygen-absorber (not to be consumed). The formulation excipients are microcrystalline cellulose, mannitol, sodium starch glycolate, and magnesium stearate. These excipients are of compendial grade and are generally recognized as safe (GRAS).

6.2.2 Storage conditions for ASTX660

All supplies must be stored in a secure, limited access storage area.

Store ASTX660 capsules refrigerated at 2°C to 8°C.

6.2.3 ASTX660 administration

The first dose of ASTX660 should always be taken in clinic, in the presence of the trial nurse. ASTX660 can be taken with or without food, with the exception of PK days. In Part A of the study, on C1D7 and C2D7, when PK samples will be taken, patients should refrain from eating for at least 2 hours prior to ingesting ASTX660 and for 2 hours after. During the 4-hour fasting period, clear liquids (such as tea, coffee, or water) are allowed; milk, juice, and soup are **not** allowed. Capsules can be swallowed with water. Small amounts of sugar (but not dairy) can be added to coffee and tea. Grapefruit is never allowed. No fasting is required in Part B.

6.2.4 Formulation of Pembrolizumab

Pembrolizumab Solution for Infusion 100 mg/vial is a liquid drug product supplied as a clear to opalescent solution, essentially free of visible particles, in Type I glass vials and manufactured using the fully formulated drug substance with L-histidine as buffering agent, polysorbate 80 as surfactant, and sucrose as stabilizer/tonicity modifier.

Pembrolizumab Solution for Infusion can be further diluted with normal saline or 5% dextrose in the concentration range of 1 to 10 mg/mL in IV containers made of polyvinyl chloride (PVC) or non-PVC material.

6.2.5 Storage conditions for Pembrolizumab

Pembrolizumab Solution for Infusion is stored under refrigerated conditions.

Reconstituted vials should be used immediately to prepare the infusion solution in the IV bag, and the infusion solution should be administered immediately. If the diluted pembrolizumab solution is not used immediately, it may be stored at room temperature for no more than 6 hours from the time of dilution. This hold time includes room temperature storage of the diluted solution and the duration of infusion. The product can also be stored under refrigeration at 2°C to 8°C for no more than 96 hours from the time of dilution. If refrigerated, the diluted solution must be allowed to come to room temperature prior to administration. The solution must be discarded after 6 hours at room temperature or 96 hours under refrigeration.

For each individual trial, clinical supplies are to be stored in accordance with specific instructions on the label.

Labelling requirements for the reconstituted pembrolizumab can be found in the Pharmacy Manual.

6.2.6 Pembrolizumab administration

Administer infusion solution intravenously over 30 minutes through an intravenous line containing a sterile, non-pyrogenic, low-protein binding, and 0.2 micron to 5 micron in line or add-on filter. Do not co-administer other drugs through the same infusion line.

Labelling requirements for the reconstituted Pembrolizumab can be found in the Pharmacy Manual.

6.3 ASTX660 and Pembrolizumab accountability

Accurate records of all IMP shipments, vials dispensed, and all vials returned must be maintained. This inventory record must be available for inspection at any time by members of the Drug Development Unit (DDU). IMP supplies are to be used only in accordance with this protocol and under the supervision of the Investigator.

The Investigator delegates responsibility for IMP management to the DDU Specialist Pharmacist. The Specialist Pharmacist undertakes not to destroy any unused IMP unless directed to by the DDU. Any unused IMP must be destroyed according to hospital procedures and properly accounted for using the IMP Destruction Form and also on the IMP Accountability Record. During the course of the trial the Clinical Trials Manager will check the numbers of bottles of ASTX660 and vials of Pembrolizumab shipped to the centre, the number used and the number destroyed or returned. The pharmacy will give an account of any discrepancy.

7 INVESTIGATIONS SCHEDULE

In cases where a patient has investigations at a different hospital, for example weekly blood samples, then it is the Investigator's responsibility to ensure he/she receives and reviews the results. The results must be recorded on the eCRF and the reports from the other hospitals must be available for source data verification. Laboratory reference ranges, including effective dates, and evidence of laboratory accreditation must be obtained from all laboratories used.

7.1 Pre-treatment evaluations

Details of all evaluations/investigations for enrolled patients, including relevant dates, required by the protocol must be recorded in the medical records so that the eCRF can be checked against the source data.

Please also refer to the tabulated Schedule of Assessments in Section 7.5.

7.1.1 Obtaining written informed consent

Written informed consent must be obtained from the patient before any protocol-specific procedures are carried out and within four weeks before the patient's first dose of the IMP [ASTX660 in Parts A and B (IO-pre-treated patients) and Pembrolizumab in Part B (IO-naïve patients)]. Initial discussion and signing of the informed consent must not occur on the same day, as the patient must be given time to think about their commitment to the trial.

Only the Principal Investigator (PI) and those Sub-Investigator(s) delegated responsibility by the PI, and have signed the Study Site Delegation Log, are permitted to gain informed consent from patients and sign the consent form. All signatures must be obtained before the occurrence of any medical intervention required by the protocol (ICH GCP 4.8.8 and 8.3.1.2). The date of the signatures of both the patient and the PI/Sub-Investigator should be the same.

The PI or the Sub-Investigator must inform the patient about the background to, and present knowledge of the normal management of their disease. The PI or sub-investigator must also inform the patient about the IMPs (ASTX660 and Pembrolizumab).The patient should be aware of the following points.

- The known toxicity of the IMP and the possibility of experiencing side-effects.
- That IMP is new and that the exact degree of activity is at present unknown, but that treating him/her will contribute to further knowledge.
- The potential dangers of becoming pregnant (or the patient's partner becoming pregnant) and he/she has been given information about appropriate medically approved contraception. (refer to Section 9.6).
- That he/she may refuse treatment either before or at any time during the trial and that refusal to participate will involve no penalty or loss of benefits to which they are otherwise entitled.
- Whom to contact for answers to pertinent questions about the research and their rights, and also who to contact in the event of a research-related injury.

A copy of the consent form and patient information sheet must be given to the patient to keep and the original consent form and patient information sheet, must be filed in the Investigator Trial File (ITF) (unless otherwise agreed that the original consent form will be filed in the medical records and the copies kept in the ITF).

7.1.2 Evaluations within four weeks (28 days)

The following must be performed/obtained **within the four weeks before** the patient receives the first dose.

- Written informed consent (as detailed in Section 7.1.1)
- Demographic details
- Medical history including prior diagnosis, prior treatment, concomitant diseases and concomitant treatment
- Tumour serum markers (if applicable)
- Archival tumour tissue; a request for archival tumour tissue must be made prior to patient enrolment, however, delays in tissue transport should not preclude enrolment of the patient on trial.
- Radiological disease assessments: Computerised Tomography (CT) scan of thorax, abdomen and pelvis; or Magnetic Resonance Imaging (MRI) scan of abdomen and pelvis, with CT chest. Patients for cohort B4 will undergo MRI Brain and CT TAP. Patients for cohorts B3 and B5 who do not have measurable disease per CT will require WB-MRI with diffusion. Additional radiological tests may be performed. Radiological measurements must be performed within four weeks before the patient receives the first dose.

Note that all adverse events (AEs), including serious adverse events (SAEs), must be monitored and recorded in the eCRF from the time the patient consents to any protocol-specific procedure (see Section 9 for further details).

7.1.3 Evaluations within two weeks (14 days)

The following must be performed **within the two weeks before** the patient receives the first dose:

- Echocardiogram

7.1.4 Evaluations within one week (seven days)

The following must be performed **within one week before** the patient receives the first dose:

- clinical disease measurements, if applicable (i.e. patients with clinically assessable disease);
- complete physical examination: cardiovascular, respiratory, central and peripheral nervous system, dermatological, gastro-intestinal, and any other system which might be relevant to the patient's medical history;
- height, weight, body surface area (BSA), WHO performance status, temperature, blood pressure (BP) and pulse rate;
- electrocardiogram (ECG): Resting 12-lead ECG, with manual calculation of QT interval (QTcF);
- serum human chorionic gonadotropin (HCG) test to rule out pregnancy at trial entry, within 72 hours of first dose; results must be obtained and reviewed before the first dose of the IMP is administered, if applicable (i.e. women of child bearing potential).
- laboratory tests (blood/urine samples) to confirm eligibility:
    - Haematology – haemoglobin (Hb), white blood cells (WBC) with differential count (neutrophils and lymphocytes) and platelets
    - Coagulation – INR, APTT
    - Biochemistry – sodium, potassium, adjusted calcium, phosphate, urea, creatinine, total protein, albumin, bilirubin, glucose, alkaline phosphatase (alk phos), alanine

aminotransferase (ALT), aspartate aminotransferase (AST), amylase and lipase. In addition, the following should be determined:

- Thyroid function tests (TSH, T4, T3)
- LDH

o Urinalysis – pH, specific gravity, glucose, protein and blood

- Collection of blood samples for PD analysis:
  - o PBMCs

7.2 Evaluations during the trial

During the trial, safety assessments may be performed 24 hours prior to dosing.

- **Physical examination**: When clinically indicated, a symptom-directed physical examination will be performed on Days 1 and 8 of each cycle before Pembrolizumab or ASTX660 administration. Additionally, in Part B of the study, for IO-naïve patients, a physical examination will be conducted on Day 1 of every cycle of Pembrolizumab single agent administration.
- WHO performance status, temperature, pulse rate, BP, bodyweight must be repeated on Days 1 and 8 of each cycle before Pembrolizumab or ASTX660 administration. Additionally, in Part B of the study, for IO-naïve patients, these evaluations will be conducted on Day 1 of every cycle of Pembrolizumab single agent administration.
- **Adverse events and concomitant treatments**: At each visit, before each ASTX660 and Pembrolizumab administration, an assessment of any AE experienced since the previous visit must be made by the Investigator or Research Nurse and the start and stop dates of the AE together with the relationship of the event to treatment with ASTX660 and Pembrolizumab must be recorded in the medical records. All AEs must be graded according to NCI CTCAE Version 5.0. Any concomitant treatment must also be recorded in the medical records and in the eCRF. (See Section 9 for further details regarding AE reporting requirements.)
- **Laboratory tests**:

  Haematology and biochemistry: must be performed on Days 1 and 8 of each cycle before Pembrolizumab or ASTX660 administration. Additionally, in Part B of the study, for IO-naïve patients, laboratory tests will be conducted on Day 1 of every cycle of Pembrolizumab single agent administration. After 4 cycles of combination dosing, the frequency of haematology and biochemistry assessments may decrease at the discretion of the Investigator. At a minimum, the assessments must be repeated before dosing on Day 1 of each cycle. During the trial, safety assessments may be performed 24 hours prior to dosing.

  - o Haematology: haemoglobin (Hb), white blood cells (WBC) with differential count (neutrophils and lymphocytes) and platelets
  - o Biochemistry: sodium, potassium, adjusted calcium, phosphate, urea, creatinine, total protein, albumin, bilirubin, glucose, alkaline phosphatase (alk phos), alanine aminotransferase (ALT), aspartate aminotransferase (AST), amylase and lipase. In addition, the following should be determined:
    - Thyroid function tests (TSH, T4, T3) every 2 cycles; dosing can occur prior to review of these results on days of dosing
  - o Coagulation: repeated on Day1 of each cycle.
  - o Urinalysis: repeated on Day 1 of each cycle, as detailed in Section 7.1.4.
- **Tumour serum markers (if applicable):** must be performed at the end of cycle 2, then repeated every 3 cycles / 9 weeks.
- **Urine HCG pregnancy test:** must be performed on Day 1 of each cycle if applicable (i.e. women of child bearing potential).

- **<u>Electrocardiogram (ECG):</u>** resting 12-lead ECG, with manual calculation of QT interval (QTcF); to be performed on Day 1 of each cycle, if clinically indicated.
- **<u>Assessment of disease:</u>** This must be repeated every 3 cycles/9 weeks. After 6 cycles, the frequency of imaging may be reduced to up to every 4 cycles, at the Investigator's discretion (Refer to Section 10).
- **<u>Collection of blood samples for PK analysis (Part A only):</u>** PK samples will be collected on Day 7 of Cycle 1 and Cycle 2. Please refer to section 8 for details of the PK sample collection schedule.
- **<u>Collection of blood samples for PD analysis:</u>** Please refer to section 8 for details of the schedule of PD samples.

7.3 Evaluations at 'off-study' visit

Evaluations at the 'off-study' visit must be performed 28 days after the last dose of either ASTX660 or Pembrolizumab. The following investigations must be done:

- a symptom-directed physical examination including WHO performance status, temperature, pulse rate, BP and bodyweight;
- haematology tests: detailed in Section 7.1.4;
- biochemistry tests: detailed in Section 7.1.4;
- urinalysis: detailed in Section 7.1.4;
- ECG
- Blood sample for PD analysis (Part A only)
- assessment of tumour disease, unless assessment has been performed within the previous four weeks (28 days);
- assessment of AEs (also see Section 7.4); and
- assessment of concomitant treatments.

7.4 Patient-reported outcomes

Symptomatic improvement is considered a clinical benefit and accepted by health authorities. As part of the analyses for this study, participants will provide information regarding their health-related quality of life (HRQoL) using the European Organization for the Research and Treatment of Cancer (EORTC) Quality of Life Questionnaire-Core 30 (QLQ-C30). These measures are not pure efficacy or safety endpoints because they are affected by both disease progression and treatment tolerability.

The EORTC QLQ-C30 is the most widely used cancer-specific HRQoL instrument, which contains 30 items and measures 5 functional dimensions (physical, role, emotional, cognitive, and social), 3 symptom items (fatigue, nausea/vomiting, and pain), 6 single items (dyspnoea, sleep disturbance, appetite loss, constipation, diarrhoea, and financial impact), and a global health status/quality of life (QoL) scale (items 29 and 30) [Aaronson, N. K., et al 1993]. The EORTC QLQ-C30 is a psychometrically and clinically validated instrument appropriate for assessing QoL in oncology studies [Aaronson, N. K., et al 1993] and has been used in Phase 2 clinical trials of Pembrolizumab single agent, and so will provide useful information on the quality of life on patients on this combination trial.

Patients will be given this questionnaire at three points during the trial: baseline, during cycle 4 and at the end of study.

7.5 Follow-up

Patients will be followed up for 28 days after the last administration of ASTX660 and for up to 90 days from the last dose of Pembrolizumab. Patients will be encouraged to attend clinic for this follow up, however, a telephone call will suffice if attendance in person is considered inappropriate for the patient. If any AEs and SAEs are considered to have a highly probable, probable or possible causal relationship to ASTX660 and/or Pembrolizumab and are still present 90 days after the last dose or occur in the 90 days post administration, then the patient will be followed up monthly afterwards until resolution to baseline or stabilisation of these events.

Patients will be followed up for survival status every 3 months from the end of study visit and for up to two years or as clinically indicated.

7.6 Schedule of events

PART A: Dose escalation

**Observation/Investigation**	**Baseline / Pre-study**			**Cycle 1**			**Cycle 2**			**Cycle 3 onwards**		**Off study and Follow-up**		
	Within 4 weeks prior to first dose	Within 2 weeks prior to first dose	Within 1 week prior to first dose	C1D1	C1D7	C1D8	C2D1	C2D7	C2D8	Day 1	Day 8	Off study: within 28 days of last IMP dose	Follow-up: monthly	Follow-up for survival
Written informed consent	X													
Demographics	X													
Medical History	X													
Adverse event evaluation	X			Continually review								X	Until resolution (a)	
Concomitant treatments	X			Continually review								X		
Radiological (CT, MRI) disease assessment (b)	X									X		X		
Tumour serum markers (if applicable) (b)	X									X		X		
Clinical disease assessment (if applicable) (b)			X							X		X		
Pregnancy test (c)			X				X			X				
Physical examination			X	X		X	X		X	X	X	X		
Temperature, weight, blood pressure & pulse			X	X		X	X		X	X	X	X		
WHO performance status			X	X		X	X		X	X	X	X		
Laboratory tests: haematology & biochemistry (d)			X	X		X	X		X	X	X	X	Until resolution of drug-related lab AEs	
Coagulation			X	X			X			X				
Urinalysis			X	X			X			X		X		

Observation/Investigation	Baseline / Pre-study			Cycle 1			Cycle 2			Cycle 3 onwards		Off study and Follow-up		
	Within 4 weeks prior to first dose	Within 2 weeks prior to first dose	Within 1 week prior to first dose	C1D1	C1D7	C1D8	C2D1	C2D7	C2D8	Day 1	Day 8	Off study: within 28 days of last IMP dose	Follow-up: monthly	Follow-up for survival
Electrocardiogram (ECG)			X	If clinically indicated			If clinically indicated			If clinically indicated		X		
Echocardiogram		X												
Archival tumour sample	X													
Tumour Biopsy				X (pre-dose)	X		X*					X*		
Blood for PBMCs (I-exp)			X			X			X (optional)					
Blood for PBMCs (II-cIAP)			X		X		X			X (f)				
Blood for FACS analysis				X (pre-dose)	X (pre-dose)		X (pre-dose)			X (pre-dose)		X		
Blood for cytokine profiling				X (pre-dose)	X (pre-dose)		X (pre-dose)			X (pre-dose)		X		
Blood for TCR analysis				X (pre-dose)					X (pre-dose)					
Blood for pharmacokinetic (PK) assays (e)					X			X						
ASTX660 administration				X	X		X	X		X				
Pembrolizumab administration						X			X		X			
EORTC QLQ-C30			X							X (Cycle 4 only)		X		
Survival status (g)														X

1 cycle = 21 days; There will be no visit windows on any scheduled cycle visit other than for safety assessments. The cycle length must always remain 21 days.

* Tumour biopsies will be taken prior to dosing on C1D1 (-2/- 1 day) and on C1D7 (or prior to Pembrolizumab dosing on C1D8). A third and fourth optional biopsy may be taken prior to dosing on C2D1 (+/- 1 day) and at treatment discontinuation.

(a) Monthly follow-up required ONLY for those AEs and SAEs considered drug-related (highly probable, probable or possible) and present at time that patient comes off the trial. Monthly follow-up to continue until resolution, to baseline, stabilisation or patient starts another anti-cancer treatment.

(b) Disease assessment must be performed at baseline and at the end of cycle 2, then repeated every 3 cycle(s)/9 weeks of continuous treatment and at off study visit (unless assessment has been performed within the previous 4 weeks). For patients who continue on treatment after 6 cycles the frequency may be reduced. Clinical disease assessment and tumour markers should be assessed if applicable

(c) Pregnancy test only required if the patient is a woman of child bearing potential. Serum pregnancy test must be performed at baseline within 72 hours of first dose, then urine HCG pregnancy test must be performed on Day 1 of each cycle.

(d) During the trial haematology and biochemistry may be performed 24 hours prior to dosing. In the event of a Grade 4 neutropenia or Grade 4 thrombocytopenia a full blood count must be performed at least on Day 5 after the onset of the event to determine if a dose limiting toxicity has occurred. Continue close monitoring until resolution to Grade 3 or less.

(I-exp) PBMC samples I are exploratory and should be taken at baseline, C1D8 and C2D8

(II-cIAP) PBMC samples II are for the analysis of cIAP1 levels and should be taken at baseline, C1D7, C2D1 and C3D1

(e) On C1D7 PK samples should be taken pre-dose, 1h and 4h post dose. On C2D7 PK samples should be taken pre-doe, then at 2h and 6h post-dose. All pre-dose samples should be taken within 30 min prior to the ASTX660 dose. For the post-dose samples, a +/- 30min window is allowed. Please ensure patients have fasted for at least 2 hours prior to ingesting study drug and for 2 hours after.

(f) PBMC samples II to be taken on C3D1 but not on any subsequent cycles.(g) Patients will be followed up for survival status every 3 months from the end of study visit and for up to two years.

PART B: Dose expansion (Cohorts B1, B4 and B5; Cohorts B2 and B3 IO pre-treated patients)

Observation/Investigation	Baseline / Pre-study			Cycle 1			Cycle 2		Cycle 3 onwards		Off study and Follow-up		
	Within 4 weeks prior to first dose	Within 2 weeks prior to first dose	Within 1 week prior to first dose	C1D1	C1D7	C1D8	C2D1	C2D8	Day 1	Day 8	Off study: within 28 days of last IMP dose	Follow-up: monthly	Follow-up for survival
Written informed consent	X												
Demographics	X												
Medical History	X												
Adverse event evaluation		X					Continually review				X	Until resolution (a)	
Concomitant treatments		X					Continually review				X		
Radiological (CT, MRI) disease assessment (b)	X								X		X		
Tumour serum markers (if applicable) (b)	X								X		X		
Clinical disease assessment (if applicable) (b)			X						X		X		
Pregnancy test (c)			X				X		X				
Physical examination			X	X		X	X	X	X	X	X		
Temperature, weight, blood pressure & pulse			X	X		X	X	X	X	X	X		
WHO performance status			X	X		X	X	X	X	X	X		
Laboratory tests: haematology & biochemistry (d)			X	X		X	X	X	X	X	X	Until resolution of drug-related lab AEs	
Coagulation			X	X			X		X				
Urinalysis			X	X			X		X		X		

Observation/Investigation	Baseline / Pre-study			Cycle 1			Cycle 2		Cycle 3 onwards		Off study and Follow-up		
	Within 4 weeks prior to first dose	Within 2 weeks prior to first dose	Within 1 week prior to first dose	C1D1	C1D7	C1D8	C2D1	C2D8	Day 1	Day 8	Off study: within 28 days of last IMP dose	Follow-up: monthly	Follow-up for survival
Electrocardiogram (ECG)			X	If clinically indicated			If clinically indicated		If clinically indicated		X		
Echocardiogram		X											
Archival tumour sample	X												
Tumour Biopsy*				X (pre-dose)				X (pre-dose)					
Blood for PBMCs (I-exp)				X (pre-dose)		X (pre-dose)		X (pre-dose)					
Blood for PBMCs (II-cIAP)			X		X		X		X (f)				
ASTX660 administration				X	X		X		X				
Pembrolizumab administration						X		X		X			
EORTC QLQ-C30			X						X (Cycle 4 only)		X		
Survival status (g)													X

1 cycle = 21 days; There will be no visit windows on any scheduled cycle visit other than for safety assessments. The cycle length must always remain 21 days.

* Tumour biopsies will be taken prior to dosing on C1D1 (-1/- 2 days) and on C2D8 (+ 7 days). Patients on cohort B4 will not require biopsies.

(a) Monthly follow-up required ONLY for those AEs and SAEs considered drug-related (highly probable, probable or possible) and present at time that patient comes off the trial. Monthly follow-up to continue until resolution, to baseline, stabilisation or patient starts another anti-cancer treatment.

(b) Disease assessment must be performed at baseline and at the end of cycle 2, then repeated every 3 cycle(s)/9 weeks of continuous treatment and at off study visit (unless assessment has been performed within the previous 4 weeks). For patients who continue on treatment after 6 cycles the frequency may be reduced. Clinical disease assessment and tumour markers should be assessed if applicable. Patients on cohort B4 will only need a single CT TAP at baseline.

(c) Pregnancy test only required if the patient is a woman of child bearing potential. Serum pregnancy test must be performed at baseline within 72 hours of first dose, then urine HCG pregnancy test must be performed on Day 1 of each cycle.

(d) During the trial haematology and biochemistry may be performed 24 hours prior to dosing. In the event of a Grade 4 neutropenia or Grade 4 thrombocytopenia a full blood count must be performed at least on Day 5 after the onset of the event to determine if a dose limiting toxicity has occurred. Continue close monitoring until resolution to Grade 3 or less.

(I-exp) PBMC samples I are exploratory and should be taken at baseline, C1D8 and C2D8

(II-cIAP) PBMC samples II are for the analysis of cIAP1 levels and should be taken at baseline, C1D7, C2D1 and C3D1

(f) PBMC samples II to be taken on C3D1 but not on any subsequent cycles.

(g) Patients will be followed up for survival status every 3 months from the end of study visit and for up to two years.

PART B: Dose expansion (Cohorts B2 and B3; IO naive patients)

Observation/Investigation	Baseline / Pre-study			Pembrolizumab single agent (e)	Cycle 1			Cycle 2		Cycle 3 onwards		Off study and Follow-up		
	Within 4 weeks prior to first dose	Within 2 weeks prior to first dose	Within 1 week prior to first dose	Day 1	C1D1	C1D7	C1D8	C2D1	C2D8	Day 1	Day 8	Off study: within 28 days of last IMP dose	Follow-up: monthly	Follow-up for survival
Written informed consent	X													
Demographics	X													
Medical History	X													
Adverse event evaluation		X						Continually review				X	Until resolution (a)	
Concomitant treatments		X						Continually review				X		
Radiological (CT, MRI) disease assessment (b)	X			X						X		X		
Tumour serum markers (if applicable) (b)	X			X						X		X		
Clinical disease assessment (if applicable) (b)			X	X						X		X		
Pregnancy test (c)			X	X				X		X				
Physical examination			X	X	X		X	X	X	X	X	X		
Temperature, weight, blood pressure & pulse			X	X	X		X	X	X	X	X	X		
WHO performance status			X	X	X		X	X	X	X	X	X		
Laboratory tests: haematology & biochemistry (d)			X	X	X		X	X	X	X	X	X	Until resolution of drug-related lab AEs	
Coagulation			X	X	X			X		X				
Urinalysis			X	X	X			X		X		X		

**Observation/Investigation**	**Baseline / Pre-study**			**Pembrolizumab single agent (e)**	**Cycle 1**			**Cycle 2**		**Cycle 3 onwards**		**Off study and Follow-up**		
	Within 4 weeks prior to first dose	Within 2 weeks prior to first dose	Within 1 week prior to first dose	Day 1	C1D1	C1D7	C1D8	C2D1	C2D8	Day 1	Day 8	Off study: within 28 days of last IMP dose	Follow-up: monthly	Follow-up for survival
Electrocardiogram (ECG)			X	X	If clinically indicated			If clinically indicated		If clinically indicated		X		
Echocardiogram		X												
Archival tumour sample	X													
Tumour Biopsy*					X (pre-dose)				X (pre-dose)					
Blood for PBMCs (I-exp)					X (pre-dose)		X (pre-dose)		X (pre-dose)					
Blood for PBMCs (II-cIAP)			X			X		X		X (f)				
ASTX660 administration					X	X		X		X				
Pembrolizumab administration				X			X		X		X			
EORTC QLQ-C30			X							X (Cycle 4 only)		X		
Survival status (g)														X

1 cycle = 21 days; There will be no visit windows on any scheduled cycle visit other than for safety assessments. The cycle length must always remain 21 days.

* Tumour biopsies will be taken prior to dosing on C1D1 (-1/- 2 days) and on C2D8 (+ 7 days).

(a) Monthly follow-up required ONLY for those AEs and SAEs considered drug-related (highly probable, probable or possible) and present at time that patient comes off the trial. Monthly follow-up to continue until resolution, to baseline, stabilisation or patient starts another anti-cancer treatment.

(b) Disease assessment must be performed at baseline and at the end of cycle 2, then repeated every 3 cycle(s)/9 weeks of continuous treatment and at off study visit (unless assessment has been performed within the previous 4 weeks). For patients who continue on treatment after 6 cycles the frequency may be reduced. Clinical disease assessment and tumour markers should be assessed if applicable.

(c) Pregnancy test only required if the patient is a woman of child bearing potential. Serum pregnancy test must be performed at baseline within 72 hours of first dose, then urine HCG pregnancy test must be performed on Day 1 of each cycle.

(d) During the trial haematology and biochemistry may be performed 24 hours prior to dosing. In the event of a Grade 4 neutropenia or Grade 4 thrombocytopenia a full blood count must be performed at least on Day 5 after the onset of the event to determine if a dose limiting toxicity has occurred. Continue close monitoring until resolution to Grade 3 or less.

(e) Pembrolizumab will be administered as a single agent on Day 1 of a 21 day cycle until patients stop responding or until they show progressive disease and thereafter in combination with ASTX660.

(I-exp) PBMC samples I are exploratory and should be taken at baseline, C1D8 and C2D8

(II-cIAP) PBMC samples II are for the analysis of cIAP1 levels and should be taken at baseline, C1D7, C2D1 and C3D1

(f) PBMC samples II to be taken on C3D1 but not on any subsequent cycles.

(g) Patients will be followed up for survival status every 3 months from the end of study visit and for up to two years.

8 PHARMACOKINETIC AND PHARMACODYNAMIC ASSESSMENTS

8.1 Pharmacokinetic Assays

Only patients in Part A of the study will undergo pharmacokinetic assessments. Patients will have plasma samples collected for measurement of ASTX660 on Cycle 1 Day 7 and Cycle 2 Day 7. ASTX660 will be measured in plasma according to agreed SOPs and validated methods. The schedule of PK sample collection is outlined in section 8.2.4.

Please refer to the Study Laboratory Manual for handling, storage and shipment of samples.

8.2 Pharmacodynamic assays

8.2.1 Collection of archival tumour specimens

Archival tumour material will be collected for all patients enrolled into the study. The tumour samples will preferably be in the form of a formalin fixed paraffin embedded block (tissue derived from the diagnostic tumour or a metastatic site). DNA extracted from archival tumour material will be analysed for genetic mutations that may impact on how patients respond to this treatment (for research purposes only).

Archival tumour biopsies will be analysed at the laboratories of the Institute of Cancer Research. Please refer to the Study Laboratory Manual for handling, storage and shipment of samples.

8.2.2 Collection of tumour biopsy material for pharmacodynamic analysis

Patients with solid tumours whose tumours are easily and safely amenable to biopsy will be asked to consent for collection of up to four pre-and post- treatment biopsies. Core needle tumour biopsies will be taken according to local procedures and will be formalin fixed and embedded in paraffin (FFPE). If the tumour is inaccessible, the biopsy is not in the patient's best interest or if they refuse, biopsies will not be taken. The schedule of biopsy collection is outlined in section 8.2.4.

In order to better understand the role of IAP blockade in the regulation of immunity and its interaction with anti-PD-L1 therapies, in Part A of the study biopsies will be collected in Cycle 1 on Day1 and Day 7, prior to ASTX660 dosing. In Part A of this study only, an additional 2 biopsies will be collected on C2D1 and at treatment discontinuation. These two biopsies are optional.

The pre- and post-treatment biopsies obtained during this period will investigate changes in the tumour microenvironment induced by IAP blockade including looking at T cell infiltration, MDSC infiltration, Treg levels and PD-L1 expression in tumour/infiltrate as well as exploratory analyses of methods of cell death. In addition, paired tumour biopsies will be used to assess changes in MHC Class I expression, immune profile changes including NF-κB and STING pathway analysis as well as immunogenic cell death. Cytokine profiling will be performed in tumour lysates. Furthermore, we will do baseline WES to look at the tumour neoantigen load and in certain patients of interest we will perform paired TCR repertoire analysis.

In the expansion Part B phase, pre- and post-treatment biopsies will be obtained to investigate changes in the tumour microenvironment induced by the combination of IAP blockade with Pembrolizumab therapy including looking at T cell infiltration, MDSC infiltration and Treg levels in tumour/infiltrate. Additionally, paired tumour biopsies will be assessed for changes in immune profiles, including NF-κB and STING pathway analysis as well as immunogenic cell death and further exploratory analyses. Paired TCR repertoire analysis will also be undertaken.

For IO-naïve patients In Part B of the study, biopsies will be taken once the patients have progressed on single agent Pembrolizumab.

Tumour biopsies will be analysed at the laboratories of the Institute of Cancer Research. Please refer to the Study Laboratory Manual for handling, storage and shipment of samples.

8.2.3 Collection of blood samples for pharmacodynamic analysis

In Part A of this study, blood samples will be collected for the following assays:

- FACS analysis on paired blood samples to examine the effect of IAP inhibition on immune cell populations.
- Immune cytokine profiling on paired plasma samples utilising a validated MSD assay to examine the effect of single agent IAP inhibitor and the combination of IAP inhibitor with pembrolizumab.
- Paired TCR repertoire analysis.
- PBMC analysis in baseline samples.

In Part B of the study paired TCR repertoire analysis and transcriptomic analysis will be undertaken.

The schedule of blood sample collection for pharmacodynamics assays is outlined in section 8.2.4. Sample analysis will take place once funding has been secured.

8.2.4 Schedule of research sample collection

Part A:

Timepoints	Tissue		Blood based biomarkers					PK samples
	Archival	Fresh	FACS (EDTA tube)	Cytokine profiling (SST tube)	TCR repertoire analysis (PAXgene tube)	PBMCs I-exploratory (Sodium citrate tube)	PBMCs II-cIAP1 analysis (Sodium citrate tube)	EDTA tube
Screening (within 4 weeks prior to first dose)	X					X	X	
C1D1 (pre-dose)		X	X	X	X			
C1D7 (pre-dose)		X	X	X			X	X***
C1D7 (1h post-dose +/- 30min)								X
C1D7 (4h post-dose +/- 30min)								X
C1D8 (pre-dose)						X		
C2D1 (pre-dose)		X*	X	X			X	
C2D7 (pre-dose)								X***
C2D7 (2h post-dose +/- 30min)								X
C2D7 (6h post-dose +/- 30min)								X
C2D8 (pre-dose)					X	X**		
C3D1 (pre-dose)			X	X			X^	
Treatment discontinuation		X*	X	X				

*C2D1 and treatment discontinuation biopsies are optional

**optional

*** The pre-dose PK samples should not be taken earlier than 30min prior to ASTX660 administration

^ PBMC samples II to be taken on C3D1 but not on any subsequent cycles. FACS and Cytokine samples to be taken on D1 of every subsequent cycle.

Parts B1, B2 and B3 (IO pre-treated patients), B4 and B5:

**Timepoints**	**Tissue**		**Blood based biomarkers**	
	**Archival**	**Fresh**	**PBMCs I-exploratory (Sodium citrate tube)**	**PBMCs II-cIAP1 analysis (Sodium citrate tube)**
Screening (within 4 weeks prior to first dose)	X			X
C1D1 (pre-dose)		X	X	
C1D7 (pre-dose)				X
C1D8 (pre-dose)			X	
C2D1 (pre-dose)				X
C2D8 (pre-dose)		X	X	
C3D1 (pre-dose)				X

Parts B2 and B3 (IO naïve patients):

**Timepoints**	**Tissue**		**Blood based biomarkers**	
	**Archival**	**Fresh**	**PBMCs I-exploratory (Sodium citrate tube)**	**PBMCs II-cIAP1 analysis (Sodium citrate tube)**
Screening (within 4 weeks prior to first dose)	X			X
C1D1* (pre-dose)		X	X	
C1D7 (pre-dose)				X
C1D8 (pre-dose)			X	
C2D1 (pre-dose)				X
C2D8 (pre-dose)		X	X	
C3D1 (pre-dose)				X

* For IO naïve patients, C1D1 is the first day of ASTX660 dose

9 ASSESSMENT OF SAFETY

9.1 Adverse event definitions

9.1.1 Adverse event

For eligible patients, SAE and adverse event (AE) collection and monitoring commences from the time the patient gives their written consent to participate in the trial and continues for 28 days after the last administration of ASTX660 or 90 days after the last administration of Pembrolizumab.

Follow-up of AEs with a causality of possible, probable or highly probable will continue until the events resolve, stabilise or the patient starts another anti-cancer therapy.

An AE is any untoward, undesired or unplanned occurrence in a patient administered an IMP. An AE can be a sign, symptom, disease, and/or laboratory or physiological observation that may or may not be related to the IMP.

An abnormal laboratory parameter should be recorded as an AE if it is clinically significant or considered related to IMP.

An abnormal laboratory parameter is considered clinically significant if it meets the following criteria:

- is symptomatic;
- is treated with a con med;
- is treated with a medical procedure;
- requires an additional diagnostic test;
- results in a change in IMP dosing;
- results in the patient's withdrawal;
- meets the definition of DLT.

Abnormal lab parameters that are deemed not clinically significant and not related to IMP do not need to be recorded as AE's.

An AE includes but is not limited to those in the following list.

- A clinically significant worsening of a pre-existing condition. This includes conditions that may resolve completely and then become abnormal again.
- AEs occurring from an overdose of an IMP, whether accidental or intentional.

Other reportable events that must be treated as AEs are listed below.

- Pregnancy exposure to the IMP. Any pregnancy occurring in a patient or a patient's partner during treatment with an IMP or occurring within six months of the last IMP administration, must be reported to the DDU Pharmacovigilance Officer in the same timelines as an SAE. These should be reported even if the patient is withdrawn from the trial.
- Any AE associated with an overdose or incorrect administration of IMP. An overdose or incorrect administration of IMP is not an AE unless it results in untoward medical effects.
- Any AE that could be related to the protocol procedures, and which could modify the conduct of the trial.

Further information can be found in Appendix 6.

9.1.2 Serious adverse events (SAEs)

An SAE is any AE, regardless of dose, causality or expectedness, that:

- results in death;
- is life-threatening;
- requires in-patient hospitalisation or prolongs existing in-patient hospitalisation (some hospitalisations are exempt from SAE reporting – see Section 9.2.1);
- results in persistent or significant incapacity or disability;
- is a congenital anomaly or birth defect;
- is any other medically important event.*

*A medically important event is defined as any event that may jeopardise the patient or may require intervention to prevent one of the outcomes above. It should be noted that medically important events may be identified before or at any point in the study.

Adverse events of special interest are required to be reported by the investigator to the Sponsor immediately (i.e., no more than 24 hours after learning of the event; see Section 9.2 for reporting instructions).

Events of special interest for this study include:

- An overdose of pembrolizumab that is not associated with clinical symptoms or abnormal laboratory results. For purposes of this study, an overdose of pembrolizumab will be defined as any dose of 1,000 mg or greater (≥5 times the indicated dose). No specific information is available on the treatment of overdose of pembrolizumab. In the event of overdose, the participant should be observed closely for signs of toxicity. Appropriate supportive treatment should be provided if clinically indicated. If an adverse event(s) is associated with ("results from") the overdose of a Merck product, the adverse event(s) is reported as a serious adverse event, even if no other seriousness criteria are met.
- An elevated AST or ALT lab value that is possibly related to pembrolizumab and is greater than or equal to 3 x the upper limit of normal and an elevated total bilirubin lab value that is possibly related to pembrolizumab and is greater than or equal to 2 x the upper limit of normal and, at the same time, an alkaline phosphatase lab value that is possibly related to pembrolizumab and is less than 2 x the upper limit of normal, as determined by way of protocol-specified laboratory testing or unscheduled laboratory testing. These criteria are based upon available regulatory guidance documents. The purpose of the criteria is to specify a threshold of abnormal hepatic tests that may require an additional evaluation for an underling aetiology.

COVID-19 guidelines: For patients who have confirmed or who are suspected of having coronavirus infection, this should be reported on the SAE form as a medically important event.

9.1.3 Determining adverse event causality

The relationship of an AE to the IMP is determined as follows.

**Highly probable**<br>• Starts within a time related to the IMP administration and<br>• No obvious alternative medical explanation.
**Probable**<br>• Starts within a time related to the IMP administration and<br>• Cannot be reasonably explained by known characteristics of the patient’s clinical state.
**Possible**<br>• Starts within a time related to the IMP administration and<br>• A causal relationship between the IMP and the AE is at least a reasonable possibility.
**Unlikely**<br>• The time association or the patient’s clinical state is such that the trial drug is not likely to have had an association with the observed effect.
**Not related**<br>• The AE is definitely not associated with the IMP administered.

Note: Drug-related refers to events assessed as possible, probable or highly probable.

The Investigator must endeavour to obtain sufficient information to determine the causality of the AE (i.e. IMP, other illness, progressive malignancy etc) and must provide his/her opinion of the causal relationship between each AE and IMP. This may require instituting supplementary investigations of significant AEs based on their clinical judgement of the likely causative factors and/or include seeking a further opinion from a specialist in the field of the AE.

9.1.4 Expectedness

Assessment of expectedness will be made by the Chief Investigator (or delegate) against the RSI Sections of the current version of the ASTX660 Investigator Brochure and the Investigator Brochure for Pembrolizumab.

9.1.5 Suspected, unexpected, serious, adverse reactions (SUSARs)

A SUSAR is a suspected, unexpected, serious adverse reaction. All AEs and SAEs will be assessed for seriousness, causality and expectedness. The DDU Pharmacovigilance Officer will expedite all SUSARs to the relevant Competent Authority/Authorities and the relevant Ethics Committee(s) within the timelines specified in legislation (SI 2004/1031 as amended) i.e. 7 days for fatal/life threatening SUSARs or 15 days for non-fatal/non-life threatening SUSARs.

9.2 Expedited reporting of serious adverse events to the Pharmacovigilance Officer, DDU

All SAEs, regardless of causality, must be reported to the DDU Pharmacovigilance Officer in an expedited manner.

SAEs should be documented on an SAE report form, using the completion guidelines provided.

The SAE report form must be <u>emailed</u> to the DDU Pharmacovigilance Officer within 24 hours of site staff becoming aware of the SAE.

Email address: ddu-sae@icr.ac.uk

Each episode of an SAE must be recorded on a separate SAE report form. The NCI CTCAE Version 5.0 must be used to grade each SAE, and the worst grade recorded. If new or amended information on a previously reported SAE becomes available, the Investigator should report this to the DDU PV Officer on a new SAE report form.

If the SAE has not been reported within the specified timeframes, a reason for lateness must be added when sending the SAE report form to the DDU.

Should the Investigator become aware of any drug-related SAEs after the patient goes 'off study', these must also be reported to the DDU PV Officer within the specified timelines above.

9.2.1 Events exempt from being reported as SAEs

Events specified in this section do not require reporting as SAEs in this trial, unless hospitalisation is prolonged for any reason and then an SAE form must be completed. The events must still be recorded in the appropriate section of the case report form (CRF).

Screening period –

Part A and Part B (IO pre-treated patients):

SAEs occurring in the period prior to administration of ASTX660 or Pembrolizumab from the time of informed consent to the day prior to cycle 0 day 1 of ASTX660 administration that are not related to the design or conduct of the trial are exempt from reporting.

Part B (IO naïve patients):

SAEs occurring in the period prior to administration of ASTX660 or Pembrolizumab from the time of informed consent to the day prior to the first Pembrolizumab administration that are not related to the design or conduct of the trial are exempt from reporting

Elective admissions – Elective admissions to hospital for procedures which are planned and documented in the medical records are not SAEs, and do not require SAE reporting. Hospitalisation for administration of the IMP according to the trial protocol is also exempt from being reported as an SAE.

Death due to disease progression – cases of death due to disease progression do not require reporting unless considered related to IMP.

9.3 Recording of adverse events and serious adverse events in eCRFs

All AEs, including SAEs, must be recorded in the eCRF for eligible patients. All concomitant medications, including herbal medications and supplements must be recorded. Any therapy used to treat the event must be recorded. The eCRF will be reconciled with the safety database during and at the end of the trial. Therefore, the sites should ensure the data entered on the SAE report form and the data entered into the eCRF are consistent. The Safety Review Committee and the Investigator(s) will regularly review the safety data from both the safety and the clinical database.

9.3.1 Recording of anaemia and hypokalaemia

The NCI CTCAE Version 5.0 must be used to grade all AEs except Grade 2 or Grade 3 anaemia and Grade 1 or Grade 2 hypokalaemia.

Grade 2 or Grade 3 anaemia should be recorded by the lab value alone, regardless of any transfusion given. If a transfusion is given prophylactically or as a supportive care for a Grade 2 anaemia, this will be recorded in the patients notes and in the eCRF concomitant medication page; i.e. transfusion (prophylaxis).

Grade 1 or Grade 2 for hypokalaemia with any intervention will remain as Grade 1, where intervention is long-term supplements or oral medication. Exception to this rule is if intravenous intervention is required or if patient is symptomatic and deemed Grade 2.

The CTCAE to be used for all other grades of anaemia and hypokalaemia.

9.4 Follow-up of adverse events

Follow-up will continue until all the necessary safety data for the event has been gathered and until the drug-related AE or SAE has either resolved, returned to baseline or stabilised. The DDU Pharmacovigilance Officer will make requests for further information to the trial site at regular intervals. Requested follow-up information should be reported to the DDU Pharmacovigilance Officer in a timely manner and as soon as possible after receipt of the follow-up request. For fatal or life-threatening cases, follow-up information should be reported as soon as possible.

9.5 Urgent safety measures

The Sponsor or Investigator may take appropriate urgent safety measures (USMs) in order to protect the patient of a clinical trial against any immediate hazard to their health or safety. This includes procedures taken to protect patients from pandemics or infections that pose serious risk to human health.

USMs may be taken without prior authorisation from the competent authority.

The Medicines and Healthcare products Regulations Agency (MHRA) and the Main Research Ethics Committee (REC) must be notified within three days of such measures being taken.

Should the site initiate a USM, the Investigator must inform the Sponsor immediately either by:

- telephone 020 8722 4303 and then by email within 3 days;

  or

- email: asteroid@icr.ac.uk.

The notification must include:

- the date of the USM;
- who took the decision; and
- why action was taken.

The Sponsor will then notify the MHRA and the Main REC within three days of USM initiation.

9.6 Pregnancy

The Investigator must make every effort to try and ensure that a clinical trial patient or a partner of a clinical trial patient does not become pregnant during the trial or for six months afterwards. This should be done as part of the consent process by explaining clearly to the patient the potential dangers of becoming pregnant and also providing each patient with information about appropriate medically approved contraception. Two forms of medically approved contraception should be used, such as:

- oral contraceptives <u>and condom;</u>
- intra-uterine device (IUD) <u>and condom;</u>
- diaphragms with spermicidal gel <u>and condom.</u>

Contraceptives should be used form the time the patient joins the trial, throughout the trial and for six months after completing the trial.

It should be explained to the patient that if his partner is pregnant or breast-feeding when he enters the trial, the patient should use barrier method contraception (condom plus spermicidal gel) to prevent the unborn baby or the baby being exposed to ASTX660 or Pembrolizumab.

However, if a patient or a partner of a patient does become pregnant, the reporting procedures below must be followed.

Any pregnancy occurring in a patient or a patient’s partner during treatment with an IMP or occurring within six months of last IMP administration must be reported to the DDU Pharmacovigilance Officer within 24 hours of the site staff becoming aware of it using a Pregnancy Notification Form. It is the Investigator’s responsibility to obtain consent for follow-up from the patient or patient’s partner. The DDU Pharmacovigilance Officer will follow-up all pregnancies for the pregnancy outcome via the Investigator and document on the pregnancy form.

The Investigator must ensure that all patients are aware at the start of a clinical trial of the importance of reporting all pregnancies (in themselves and their partners) that occur whilst being treated with the IMP and occurring up to six months after the last IMP administration. The Investigator should offer counselling to the patient and/or the partner, and discuss the risks of continuing with the pregnancy and the possible effects on the foetus. Monitoring of the patient and the baby should continue until the conclusion of the pregnancy, if the patient or patient’s partner has consented to this.

9.7 SAE reporting to the Pharmaceutical Collaborators

Reporting will be per the collaborative agreements and our SOPs. Further information and details can be found in Appendix 6.

10 ASSESSMENT OF EFFICACY

Disease must be measured according to the immune-modified RECIST criteria (version1.1) given in Appendix 3 or RANO and iRANO given in Appendices 7 and 8.

10.1 Measurement of disease

Disease must be measured according to the immune-modified RECIST criteria given in Appendix 3. Glioblastoma patients will be assessed according to RANO and iRANO criteria as per Appendices 7 and 8. Patients with mCRPC patients who have RECIST measurable disease will be assessed according to the immune-modified RECIST criteria, but those with metastatic bone only metastastic disease will be assessed with PCWG 3 criteria.

10.2 Timing and type of tumour assessments

A thorough clinical and radiological evaluation of malignancy, as judged appropriate by the Investigator, and in line with the protocol, must be performed before starting the investigational medicinal product (IMP). The same methods that detect evaluable lesions at baseline must be used to follow these lesions throughout the trial. To ensure compatibility, the radiological assessments used to assess response must be performed using identical techniques. Imaging based evaluation is preferred to evaluation by clinical examination when both methods have been used to assess the anti-tumour effect of a treatment.

All radiological assessments must be performed within four weeks before starting treatment. The interval between the last anti-cancer therapy and these measurements must be at least four weeks (28 days). All clinical measurements to assess response must be done within **one** week before the patient starting treatment.

All complete (CR) and partial responses (PR) must be confirmed by two consecutive observations not less than four weeks apart.

Disease response in ovarian cancer patients may be determined by CA125 levels according to GCIG Criteria. CA125 levels will be measured and documented prior to initiation of study drug. Response and progression will be evaluated based on repeat tumour marker measurements performed every other cycle of therapy (approximately every eight weeks).

Copies of the scans must be available for external independent review if requested by the Drug Development Unit.

10.2.1 Immune-modified RECIST

Increasing clinical experience indicates that traditional response criteria (e.g., RECIST v1.1 and World Health Organization criteria) may not adequately assess the activity of immunotherapeutic agents because initial radiologic evidence of disease progression does not necessarily reflect therapeutic failure. Patients can experience a response in the presence of new lesions or after an increase in tumour burden. Thus, this study will employ immune-modified RECIST (imRECIST) for tumour assessments of soft tissue lesions to account for the possible appearance of new lesions and allow radiologic progression to be confirmed at a subsequent assessment at least 4 weeks after the 1st radiological progression is documented (see Appendix 3).

It is recommended that radiologic progression in soft tissue lesions be confirmed at a subsequent tumour assessment to take into account the potential for pseudoprogression (caused by immune cell infiltration). Given the proposed immunomodulatory mechanism of action of pembrolizumab and the possibility of observing delayed responses, use of immune-modified RECIST will allow for the capture of a greater proportion of potential responses and allow patients to derive maximum clinical benefit.

10.2.2 RANO / iRANO Criteria

For patients with recurrent or progressive GBM or high-grade glioma, evaluation of disease progression and response will be assessed by contrast-enhanced MRI, based on the RANO criteria (Appendix 7).

Among patients who demonstrate imaging findings meeting RANO criteria for progressive disease (PD) within **six** months of initiating immunotherapy including the development of new lesions, confirmation of radiographic progression on follow-up imaging per immunotherapy response assessment for neuro-oncology (iRANO) is recommended provided that the patient is not significantly worse clinically. (Appendix 8). Clinical progression of the tumour disease will also be considered as progressive disease (Okada et al, 2015).

Copies of the scans must be available for external independent review if requested by the Drug Development Unit.Baseline evaluations

These must include radiological measurements and as indicated chest, abdominal and pelvic computerised tomography (CT) scan, magnetic resonance imaging (MRI) and/or clinical measurements as appropriate. All areas of disease present must be documented (even if specific lesions are not going to be followed for response) and the measurements of all measurable lesions must be recorded clearly on the scan reports. Any non-measurable lesions must be stated as being present.

10.2.3 Evaluations during and at 'off-study'

Tumour assessments must be repeated every 3 cycles (9 weeks) or more frequently, when clinically indicated. All lesions measured at baseline must be measured at every subsequent disease assessment, and recorded clearly on the scan reports. All non-measurable lesions noted at baseline must be noted on the scan report as present or absent.

In studies of immunotherapeutic agents, complete response (CR), PR, and stable disease have each been shown to occur after radiographic evidence of an apparent increase in tumour burden. This initial increase in tumour burden caused by immune cell infiltration in the setting of a T-cell response has been termed pseudoprogression (Hales et al. 2010). In this study, selected patients may be considered for continued treatment after RECIST 1.1 progression, provided the benefit-risk ratio is judged to be favourable by the investigator, and the patient provides written confirmation of consent to continue treatment, and a repeat imaging for immune-modified RECIST assessment is planned (at least four weeks from 1st RECIST PD scan). All patients, who are removed from the trial for reasons other than progressive disease, must be re-evaluated at the time of treatment discontinuation, unless a tumour assessment was performed within the previous four weeks.

It is the responsibility of the Principal Investigator to ensure that the radiologists are aware of the requirement to follow-up and measure every target lesion mentioned at baseline and comment on the non-target lesions in accordance with imRECIST criteria.

10.3 Tumour response

All patients who meet the eligibility criteria, receive at least two cycles of combination treatment and have a baseline assessment of disease will be evaluable for response by RECIST criteria. To be assigned a status of complete response (CR) or partial response (PR), changes in tumour measurements must be confirmed by repeat measurements performed no less than four weeks after the response criteria are met. To be assigned a status of stable disease (SD), follow-up measurements must have met the SD criteria at least once and at least six weeks after the initial dose of the investigational medicinal product (IMP) is given.

Should rapid tumour progression occur before the completion of Cycle 1 the patient will be classified as having early progression (EP).

Tumour response should be classified as “not evaluable” (NE), only when it is not possible to classify it under another response category, for example, when baseline and/or follow-up assessment is not performed or not performed appropriately.

Expert reviewers appointed by DDU may undertake an independent review of all the Investigator’s assessed objective responses (CR and PR). The expert reviewers will include at least one specialist who is not an Investigator in the trial. In case of disagreement between the Investigator’s and the expert reviewers’ assessment, discussion will take place between the two parties in order to reach a consensus. However, if it is not forthcoming, the assessment of the expert reviewers will be retained in the clinical study report. The eCRF will reflect the Investigator’s opinion.

10.3.1 Recording of response in the eCRF

The applicable overall response category for each visit that includes disease assessment must be recorded in the eCRF, even though the criteria for determination of CR or PR by the protocol must be confirmed after two consecutive observations, no less than four weeks apart.

11 DISCONTINUATION OF TREATMENT/ WITHDRAWAL FROM TRIAL

The Investigator must make every reasonable effort to keep each patient on trial for the whole duration of the trial (i.e. until 28 days after last combination therapy administration). However, if the Investigator removes a patient from the trial or if the patient declines further participation, final 'off-study' assessments should be performed before any therapeutic intervention. All the results of the evaluations and observations, together with a description of the reasons for withdrawal from the trial, must be recorded in the medical records and in the eCRF.

Patients who are removed from the trial due to adverse events (clinical or laboratory) will be treated and followed according to accepted medical practice. All pertinent information concerning the outcome of such treatment must be recorded in the eCRF and on the serious adverse event (SAE) report form where necessary. Patients who have experienced a DLT must be discussed with Chief Investigator and/or the SRC as to the risk-benefits of continuing on the trial. All patients will be followed up for overall survival unless they withdraw consent.

The following are justifiable reasons for the Investigator to withdraw a patient from trial:

- AE/SAE;
- Unacceptable toxicity;
- Withdrawal of consent;
- Serious violation of the trial protocol (including persistent patient attendance failure and persistent non-compliance);
- Sponsor's decision to terminate the trial;
- Withdrawal by the Investigator for clinical reasons not related to the IMP;
- Evidence of disease progression;
- Pregnancy (for female patients during the study);
- Symptomatic deterioration.

12 DEFINING THE END OF TRIAL

The ‘end of trial’ is defined as the date when the last patient has completed the ‘off-study’ visit or the final follow-up visit (whichever is the latter).

It is the responsibility of the DDU to inform the Medicines and Healthcare products Regulations Agency (MHRA) and the Main Research Ethics Committee (REC) within 90 days of the ‘end of the trial’ that the trial has closed.

In cases of early termination of the trial (for example, due to toxicity) or a temporary halt by the DDU, the DDU will notify the MHRA and the Main REC within 15 days of the decision and a detailed, written explanation for the termination/halt will be given.

The entire trial will be stopped when:

- The drug is considered too toxic to continue treatment before the required number of patients being recruited.
- The stated number of patients to be recruited is reached.
- The stated objectives of the trial are achieved.

Regardless of the reason for termination, all data available for the patient at the time of discontinuation of follow-up must be recorded in the eCRF. All reasons for discontinuation of treatment must be documented.

In terminating the trial, DDU and the Investigators must ensure that adequate consideration is given to the protection of the patient’s interest.

13 DATA ANALYSIS AND STATISTICAL CONSIDERATIONS

The final analysis will be conducted after one of the following conditions is met.

- The trial is terminated early (for example, due to toxicity).
- All patients have had the opportunity to receive one cycle of treatment and have completed their 'off-study' visit (i.e. 28 days after the last dose of ASTX660 or 90 days after the last dose of pembrolizumab).

Once one of the conditions is met, a data cut-off date will be established. All patient visits occurring on or before this date will be analysed and summarised in the final clinical study report. Any data collected after this date will be summarised in a supplemental report.

13.1 Statistical considerations and analysis populations

13.1.1 Escalation phase

A dose-escalation design based on an efficient, modified Bayesian TITE-CRM will be used to identify the MTD. The full DLT assessment period is 6 weeks. The TITE-CRM will target the dose (from a selection of four possible doses) with estimated DLT rate closest to the target rate of 25%. The skeletons or prior DLT rates at each dose levels have been elicited using a combination of model-calibrated priors (23) and through discussion with clinicians (using an acceptable target DLT rates in the range between 19% and 31%). We have used the getprior function (in R package dfcrm) where the prior MTD guess is at Dose level 3. Starting dose is dose level 1, d(1).

An empiric dose-toxicity model $F(x,\beta) = x^{exp(\beta)}$, $0 < x < 1$ will be used, where the slope parameter β is assumed to follow a normal distribution with prior mean 0 and a pre-specified prior variance of 0.85.

The TITE-CRM design will incorporate practical considerations, including no skipping of untried doses in escalation, no escalation if the overall rate of toxicity seen at the current dose level is above the target rate and for stopping early if there is a high probability that the lowest dose is too toxic. Specifically the model will suggest stopping early due to excess toxicity if $P(\theta_L > 0.25 \mid data) > 0.88$, where theta is the probability of DLT at the lowest dose level. Assuming patients have completed their DLT follow-up period, this translates to stopping early when we see 2/2 DLT or 3/4 at the lowest dose (Yap et al 2017).

The operating characteristics of the proposed TITE-CRM design for up to 18 patients (Dose escalation phase) and 58 patients (total in trial) are provided separately in the statistical simulation plan.

13.1.2 Expansion phase

Using a Bayesian approach, we can quantify how confident we are that the clinical benefit of the novel combination therapy is greater than 5%. The underlying true clinical benefit rate is assessed using a beta-binomial conjugate analysis. A weakly informative prior of Be(0.2,0.8) with an equivalent sample size of 1 is used. This gives a clinical benefit rate of 20% with an equivalent sample size of 1. In other words, before the trial starts, we are adding in one equivalent patient data with a mean activity rate of 20%. The 3-outcome decision criteria is based on ProbA = P(true clinical benefit rate > 5% | data) with the threshold boundaries as follows:

NO GO	INCONCLUSIVE	GO
Strong signal that treatment is unlikely to be active $Prob_A \leq 0.12$	Unclear. Use other key outcomes for decision-making or recruit further patients $0.12 < Prob_A < 0.9$	Strong signal that treatment is active $Prob_A \geq 0.9$

The recommended decision that will be undertaken if we see n_R responders out of n patients can be tabulated in advance. Table 4 presents the posterior probabilities and decision that will be undertaken for all possible number of responders out of 10 and 15 patients. From Table 4, if we see 0 responders out of 10 patients, the posterior probability that the true clinical benefit rate is >5% is 0.11, the decision is NO GO. If we see at least 2 responders out of 10 patients, the decision is GO. Otherwise if it is 1/10 responders, it is INCONCLUSIVE.

Table 5 presents the performance of this 3-outcome Bayesian design using 10,000 simulations. If there is a strong signal of true activity of 40%, there is 96% chance of correctly indicating that the treatment is active and should proceed for further testing, with a very low chance of 1% of incorrectly indicating that it is a NO GO. In the case where the treatment is truly inactive (3%), we have a 73% and 23% chance of indicating it as a NO GO and INCONCLUSIVE respectively, and a low 4% chance of incorrectly indicating a GO. Further simulations and fine-tuning of the decision criteria will be undertaken to optimise the design further.

Table 4. Posterior Probabilities that the true clinical benefit rate is greater than 5% given the observed data ($Prob_A$) using a 3-outcome Bayesian decision framework, with the decision of either GO ($Prob_A \geq 0.9$), NO GO ($Prob_A \leq 0.12$) or INCONCLUSIVE ($0.12 < Prob_A < 0.9$).

Observed Number of Responses, n_R	Number of patients, n	P(true clinical benefit rate > 5% | data)	Decision
0	10	0.11	NO GO
1	10	0.69	INCONCLUSIVE
2	10	0.94	GO
3-10	10	>0.99	GO
0	15	0.07	NO GO
1	15	0.56	INCONCLUSIVE
2	15	0.87	INCONCLUSIVE
3	15	0.97	GO
4-15	15	>0.99	GO

Table 5. Operating characteristics of the 3-outcome Bayesian design for 10 patients, with 10,000 simulations, based on the decision criterion of $Prob_A$ = P(true clinical benefit rate > 5% | data), where GO: $Prob_A \geq 0.9$, NO GO: $Prob_A \leq 0.12$ and INCONCLUSIVE otherwise. The probabilities in red are the errors one would like to avoid.

True Clinical Benefit Rate	Probability of recommending NO GO	INCONCLUSIVE	GO
Strong negative signal 0.03	0.73	0.23	0.04
Moderate activity signal 0.2	0.1	0.27	0.63
Strong positive signal 0.3	0.03	0.12	0.85
Strong positive signal 0.4	0.01	0.04	0.95

We have chosen to adopt a Bayesian approach to decision-making as it provides greater flexibility for real-life evaluation due to the uncertainty regarding the eventual number in each expansion cohort. Each expansion cohort will be analysed and reported independently.

For each cohort, the posterior probabilities that the true clinical benefit rate is greater than 5% will be presented with the corresponding Bayesian decision outcome, as demonstrated in Table 4. Additionally, a sensitivity analysis assessing the shared response rate amongst all expansion cohorts will be conducted to understand the robustness of these findings. Full particulars of analysis methods for secondary and tertiary endpoints, including this sensitivity analysis, will be specified in the statistical analysis plan.

Further Safety (MTD) Evaluation

During the expansion phase where up to an additional 50 patients may be recruited in 5 specific cohorts, we will continue to update the TITE-CRM model regularly, taking into account all the accumulated DLT data gathered thus far. This will enable us to use the additional safety data to determine if the initial MTD (based on the first 18 DLT-evaluable patients) remains the same. This will provide a more refined and accurate estimate of the MTD than that obtainable from the initial patients in the dose-escalation phase. Several authors have advocated the use of additional data in dose expansion cohorts to increase the accuracy of MTD determination, and to allow for re-adjustment of the MTD if required [Iasonos & Quigley 2015, Boonstra et al 2015]. If the model recommends any adjustment of the recommended dose as the data accumulate, the Safety Review Committee will review the data, and subsequent patients may receive the updated dose. The statistical simulation plan encompasses simulations comparing TITE CRM's performance using data from 18 patients in the dose escalation phase against data from up to 68 patients from both dose escalation and expansion stages.

13.2 Presentation of data

Data will be presented in a descriptive fashion. Variables will be analysed to determine whether the criteria for the trial conduct are met. This will include a description of patients who did not meet all the eligibility criteria, an assessment of protocol violations, IMP accountability and other data that impact on the general conduct of the trial.

Baseline characteristics will be summarised for all enrolled patients. Patients who died or withdrew before treatment started or did not complete the required safety observations will be described and evaluated separately.

Treatment administration will be described for all cycles. Dose administration, dose modifications or delays and the duration of therapy will be described.

13.3 Safety

Safety data will be collected from the date of written consent. Safety variables will be summarised by descriptive statistics. Laboratory variables will be described using the National Cancer Institute (NCI) Common Terminology Criteria for Adverse Events (CTCAE) Version 5.0.

Adverse events (AEs) will be reported for each dose level and presented as tables of frequency of AEs by body system and by worse severity grade observed. Tables should indicate related and unrelated events. Laboratory data will be presented by dose level at each observation time. Values outside normal limits will be identified and summarised by frequency distribution.

13.4 Pharmacokinetics

In Part A of the study the plasma concentration/time data will be analysed using non-compartmental methods. The pharmacokinetic (PK) parameters to be determined for ASTX660 include the maximum observed plasma concentration (C_{max}), time to reach C_{max} (T_{max}), and the area under the plasma concentration time curve (AUC), and the terminal elimination half-life ($T_{1/2}$), mean residence time (MRT), total body clearance (CLT) and steady/state volume of distribution (V_{SS}).

13.5 Pharmacodynamics

Determination of changes in immune-cell population (using e.g FACS or mass cytometry) and host immune cell cytokine profile. Changes in the tumour micro-environment including changes in tumour-infiltrating lymphocytes, myeloid derived suppressor cells (MDSCs) and Tregs; as well as changes in PD-L1 expression. There is no formal statistical analysis.

13.6 Anti-tumour activity

Documenting anti-tumour activity is a primary objective in Part B of this trial and a secondary objective in Part A. Patients must receive at least two cycles of the combination trial medication to be evaluable for response. Objective responses, the best tumour response achieved by each patient while on trial and the time to progression will be presented in the data listings.

Time-to-event outcomes will be analysed through the production of Kaplan-Meier plots. Binary outcomes will be summarised through descriptive statistics; logistic modelling may be considered. Repeated measured data (including QoL) will be analysed through descriptive statistics and mixed effects modelling (accounting for within subject correlation) using parametric or more flexible models to assess for changes over time. Toxicity data (AE and SAE) will be summarised by dose and by number and proportions in each category as appropriate.

Further details will be provided in the statistical analysis plan.

14 ADMINISTRATION

This trial is conducted under a clinical trial authorisation (CTA) and approval from the Medicines and Healthcare products Regulations Agency (MHRA) and the relevant Research Ethics Committee(s) will be obtained before the start of this trial. This trial is sponsored by The Institute of Cancer Research (ICR). Applicable regulatory requirements are described in this section.

14.1 Protocol deviations and amendments

Eligibility waivers are strictly prohibited.

Amendments to the protocol may only be made with the approval of the DDU, CI and statistician. A protocol amendment will be subject to review by the Sponsor. Ethics Committee favourable opinion (and MHRA approval if appropriate) will be obtained by the DDU on behalf of the CI before the amendment can be implemented and incorporated into the protocol if necessary.

14.2 Completion of the case report form (CRF)

Electronic CRFs (eCRFs) will be used to collect the data. The Investigator is responsible for ensuring the accuracy, completeness, clarity and timeliness of the data reported in the eCRFs.

Only the Investigator and those personnel who have signed the Study Team Responsibilities Signature Log/Delegation Log provided by the DDU and have been authorised by the Investigator should enter or change data in the eCRFs. All protocol required investigations must be reported in the eCRF. The Investigators must retain all original reports, traces and images from these investigations for future reference.

Data will be entered directly into eCRFs by authorised site personnel.

Once data has been entered by the site personnel on the eCRF, the data will be reviewed and checked for error and inconsistencies by data management staff in the DDU.

Once the patient is ‘off study’ and the eCRF has been fully completed, the Investigator must provide a signature to authorise the complete subject casebook.

14.3 Trial performance and monitoring

14.3.1 Management of the study

The DDU Clinical Trials Manager will be responsible for the day to day coordination and management of the trial. The ICR Clinical Trials and Statistics Unit (ICR-CTSU) will act as custodian of the data on behalf of the sponsor. The DDU is responsible for all duties relating to pharmacovigilance in accordance with section 9.

Before the trial can be initiated, the prerequisites for conducting the trial must be clarified and the organisational preparations made with the trial centre. The DDU must be informed immediately of any change in the personnel involved in the conduct of the trial.

During the trial the DDU Clinical Trials Manager is responsible for monitoring data quality in accordance with DDU’s standard operating procedures (SOPs). Before the trial start, the Investigator will be advised of the anticipated frequency of the monitoring visits. The Investigator will receive reasonable notification before each monitoring visit.

It is the responsibility of the Clinical Trials Manager to:

- review trial records and compare them with source documents;
- check pharmacodynamic samples and storage;
- discuss the conduct of the trial and the emerging problems with the Investigator;
- check that the drug storage, dispensing and retrieval are reliable and appropriate; and
- verify that the available facilities remain acceptable.

All unused drug supplied must be properly destroyed at the Investigator site, upon confirmation by DDU, by an authorised person who will provide signed confirmation.

It is the responsibility of the DDU to inform the Main REC within 90 days of the 'end of the trial' (i.e. last patient study visit) that the trial has closed.

14.3.2 Source document verification

All data collected in the eCRF must be verifiable by the source data. Therefore it is the Investigator's responsibility to ensure that both he/she and his/her study team records all relevant data in the medical records. The Investigator must allow the CTM direct access to relevant source documentation for verification of data entered into the eCRF, taking into account data protection regulations. Entries in the eCRF will be compared with patients' medical records and the verification will be documented on the source data verification (SDV) form and the monitoring report.

The patients' medical records, and other relevant data, may also be reviewed by appropriate qualified personnel independent from the DDU appointed to audit the trial, and by regulatory authorities. Details will remain confidential and patients' names will not be recorded outside the hospital.

14.4 Clinical study report

All clinical data will be presented at the end of the trial on final data listings. The CI, DDU and ICR-CTSU will prepare a clinical study report based on the final data listings. The report will be submitted to the Investigator(s) for review and confirmation it accurately represents the data collected during the course of the trial. A summary of the final clinical report will be provided by the DDU to the MHRA and to the Research Ethics Committee.

14.5 Record retention

During the clinical trial and after trial closure the Investigator must maintain adequate and accurate records to enable both the conduct of a clinical trial and the quality of the data produced to be evaluated and verified. These essential documents (as detailed in Chapter V of Volume 10 (Clinical Trials) of The Rules Governing Medicinal Products in the European Union based upon Section 8 of the ICH GCP Guidelines), including source documents such as scans, trial related documents and copies of the eCRFs, associated audit trail and serious adverse event (SAE) report forms, shall show whether the Investigator has complied with the principles and guidelines of Good Clinical Practice (GCP).

All essential documents required to be held by the Investigator must be stored in such a way that ensures that they are readily available, upon request, to the Regulatory Agency or Sponsor, for the minimum period required by national legislation. Records must not be destroyed without prior written approval from the DDU.

The medical files of trial subjects shall be retained in accordance with national legislation and in accordance with the maximum period of time permitted by the hospital, institution or private practice.

14.6 Ethical considerations

Before starting the trial, the protocol, patient information sheet, consent form and all relevant study documentation must be approved by the ICR/RM joint Committee for Clinical Research, the appropriate Ethics Committee and the Health Research Authority.

It is the Chief/Principal Investigator's responsibility to update patients (or their authorised representatives, if applicable) whenever new information (in nature or severity) becomes available that might affect the patient's willingness to continue in the trial. The Chief/Principal Investigator must ensure this is documented in the patient's medical notes and the patient is re-consented.

The Sponsor and Chief/Principal Investigator must ensure that the trial is carried out in accordance with the GCP principles and requirements of the UK Clinical Trials regulations (SI 2004/1031 and SI 2006/1928 as amended).

14.7 Indemnity

This trial is being carried out under the auspices of The Institute of Cancer Research. The Institute of Cancer Research will provide patients with compensation for adverse side effects and has in place no-fault compensation insurance for any potential injury caused by participation in this clinical trial.

Indemnity for participating hospitals is provided by the usual NHS indemnity arrangements for clinical negligence.

14.8 Publication policy and press releases

The trial results will be submitted for publication in a relevant medical journal with authorship according to the criteria defined by the ICMJE (http://www.icmje.org). These state that: Authorship credit should be based 1) substantial contributions to conception and design, acquisition of data, or analysis and interpretation of data; 2) drafting the article or revising it critically for important intellectual content; and 3) final approval of the version to be published. Authors should meet conditions 1, 2, and 3.

Draft publications (manuscripts, abstracts, slides and posters) should be submitted to the DDU Clinical Trial Coordinator for circulation to the relevant parties and the ICR-CTSU to allow sufficient time for review prior to submission.

The DDU shall have for abstracts, slides and posters a twenty-one (21) day period and for manuscripts a thirty (30) day period to respond to the author with any revisions.

15 REFERENCES

1. Lopez J, Meier P. To fight or die - inhibitor of apoptosis proteins at the crossroad of innate immunity and death. Curr Opin Cell Biol. 2010;22(6):872-81.

2. Tenev T, Bianchi K, Darding M, Broemer M, Langlais C, Wallberg F, et al. The Ripoptosome, a signaling platform that assembles in response to genotoxic stress and loss of IAPs. Mol Cell. 2011;43(3):432-48.

3. Feoktistova M, Geserick P, Kellert B, Dimitrova DP, Langlais C, Hupe M, et al. cIAPs block Ripoptosome formation, a RIP1/caspase-8 containing intracellular cell death complex differentially regulated by cFLIP isoforms. Mol Cell. 2011;43(3):449-63.

4. Darding M, Meier P. IAPs: guardians of RIPK1. Cell Death Differ. 2012;19(1):58-66.

5. Yatim N, Cullen S, Albert ML. Dying cells actively regulate adaptive immune responses. Nat Rev Immunol. 2017;17(4):262-75.

6. Yatim N, Jusforgues-Saklani H, Orozco S, Schulz O, Barreira da Silva R, Reis e Sousa C, et al. RIPK1 and NF-kappaB signaling in dying cells determines cross-priming of CD8(+) T cells. Science. 2015;350(6258):328-34.

7. Dougan SK, Dougan M. Regulation of innate and adaptive antitumor immunity by IAP antagonists. Immunotherapy. 2018;10(9):787-96.

8. Muller-Sienerth N, Dietz L, Holtz P, Kapp M, Grigoleit GU, Schmuck C, et al. SMAC mimetic BV6 induces cell death in monocytes and maturation of monocyte-derived dendritic cells. PLoS One. 2011;6(6):e21556.

9. Chesi M, Mirza NN, Garbitt VM, Sharik ME, Dueck AC, Asmann YW, et al. IAP antagonists induce anti-tumor immunity in multiple myeloma. Nat Med. 2016;22(12):1411-20.

10. Nomi T, Sho M, Akahori T, Hamada K, Kubo A, Kanehiro H, et al. Clinical significance and therapeutic potential of the programmed death-1 ligand/programmed death-1 pathway in human pancreatic cancer. Clin Cancer Res. 2007;13(7):2151-7.

11. Cai G, Karni A, Oliveira EM, Weiner HL, Hafler DA, Freeman GJ. PD-1 ligands, negative regulators for activation of naive, memory, and recently activated human CD4+ T cells. Cell Immunol. 2004;230(2):89-98.

12. Blank C, Mackensen A. Contribution of the PD-L1/PD-1 pathway to T-cell exhaustion: an update on implications for chronic infections and tumor evasion. Cancer Immunol Immunother. 2007;56(5):739-45.

13. Iwai Y, Ishida M, Tanaka Y, Okazaki T, Honjo T, Minato N. Involvement of PD-L1 on tumor cells in the escape from host immune system and tumor immunotherapy by PD-L1 blockade. Proc Natl Acad Sci U S A. 2002;99(19):12293-7.

14. Tsushima F, Tanaka K, Otsuki N, Youngnak P, Iwai H, Omura K, et al. Predominant expression of B7-H1 and its immunoregulatory roles in oral squamous cell carcinoma. Oral Oncol. 2006;42(3):268-74.

15. Korman A, Chen B, Wang C, Wu L, Cardarelli P, Selby M. Activity of Anti-PD-1 in Murine Tumor Models: Role of “Host” PD-L1 and Synergistic Effect of Anti-PD-1 and Anti-CTLA-4 (48.37). The Journal of Immunology. 2007;178(1 Supplement):S82.

16. Gaughan EM, Petroni GR, Grosh WW, Slingluff CL. Salvage combination ipilimumab and nivolumab after failure of prior checkpoint inhibitor therapy in patients with advanced melanoma. Journal of Clinical Oncology. 2017;35(15_suppl):e21009-e.

17. Chung HC, Ros W, Delord J-P, Perets R, Italiano A, Shapira-Frommer R, et al. Efficacy and Safety of Pembrolizumab in Previously Treated Advanced Cervical Cancer: Results From the Phase II KEYNOTE-158 Study. Journal of Clinical Oncology. 2019;37(17):1470-8.

18. Cortés J, André F, Gonçalves A, Kümmel S, Martín M, Schmid P, et al. IMpassion132 Phase III trial: atezolizumab and chemotherapy in early relapsing metastatic triple-negative breast cancer. Future Oncology. 2019;15(17):1951-61.

19. Emens LA, Cruz C, Eder JP, Braiteh F, Chung C, Tolaney SM, et al. Long-term Clinical Outcomes and Biomarker Analyses of Atezolizumab Therapy for Patients With Metastatic Triple-Negative Breast Cancer: A Phase 1 Study. JAMA Oncology. 2019;5(1):74-82.

20. Blumenthal DT, Yalon M, Vainer GW, Lossos A, Yust S, Tzach L, et al. Pembrolizumab: first experience with recurrent primary central nervous system (CNS) tumors. J Neurooncol. 2016;129(3):453-60.

21. Sargent DJ, Chan V, Goldberg RM. A three-outcome design for phase II clinical trials. Control Clin Trials. 2001;22(2):117-25.

22. Yap C, Pettitt A, Billingham L. Screened selection design for randomised phase II oncology trials: an example in chronic lymphocytic leukaemia. BMC Med Res Methodol. 2013;13:87.

23. Lee SM, Cheung YK. Calibration of prior variance in the Bayesian continual reassessment method. Stat Med. 2011;30(17):2081-9.

24. Mita MM, LoRusso PM, Papadopoulos KP, Gordon MS, Mita AC, Ferraldeschi R, et al. A Phase I study of ASTX660, an antagosnist of Inhibitors of Apoptosis Proteins, in adults with advanced cancers or Lymphoma. Clinical Cancer Research. 2020; 26(12):2819-2826.

25. Boonstra PS, Shen J, Taylor JMG, Braun TM, Griffith KA, Daignault S, et al. A statistical evaluation of dose expansion cohorts in phase I clinical trials. J Natl Cancer Inst. 2015; 107(3).

26. Iasonos A, O'Quigley J. Adaptive dose-finding studies: a review of model-guided phase I clinical trials. J Clin Oncol. 2014; 32(23):2505-11

27. https://www.fda.gov/about-fda/oncology-center-excellence/project-optimus

Note for Guidance on Good Clinical Practice. ICH Topic E6. CSMP/ICH/135/95. EMEA, May 1996, updated September 1997 with post Step 4 errata included

DIRECTIVE 2001/20/EC OF THE EUROPEAN PARLIAMENT AND OF THE COUNCIL of 4 April 2001 on the approximation of the laws, regulations and administrative provisions of the Member States relating to the implementation of good clinical practice in the conduct of clinical trials on medicinal products for human use. *Official Journal of the European Communities L121/34-44*

Detailed guidance for the request for authorisation of a clinical trial on a medicinal product for human use to the competent authorities, notification of substantial amendments and declaration of the end of the trial. **October 2005***. ENTR/F2/BL D(2003) CT 1 (Revision 2)*

Detailed guidance on the application format and documentation to be submitted in an application for an Ethics Committee opinion on the clinical trial on medicinal products for human use. **February 2006*.*** *ENTR/CT 2 (Revision 1)*

Detailed guidance on the collection, verification and presentation of adverse reaction reports arising from clinical trials on medicinal products for human use. **April 2006.** *ENTR/CT 3 (Revision 2)*

The **Medicines** for Human Use (Clinical Trials) Regulations 2004 (Statutory Instrument 1031)

COMMISSION DIRECTIVE 2005/28/EC of 8 April 2005 laying down principles and detailed guidelines for good clinical practice as regards investigational medicinal products for human use, as well as the requirements for authorisation of the manufacturing or importation of such products. *Official Journal of the European Union L 91/13*

The Medicines for Human use (Clinical Trials) Amendment Regulations 2006 (Statutory Instrument 2006/1928).

16 APPENDICES

16.1 APPENDIX 1: WHO PERFORMANCE SCALE

Activity Performance Description	Score
Fully active, able to carry out all normal activity without restriction.	0
Restricted in physically strenuous activity but ambulatory and able to carry out work of a light or sedentary nature, for example, light housework, office work.	1
Ambulatory and capable of all self-care, but unable to carry out any work activities. Up and about more than 50% of waking hours.	2
Capable of only limited self-care. Confined to bed or chair more than 50% of waking hours.	3
Completely disabled. Cannot carry out any self-care. Totally confined to bed or chair.	4

16.2 APPENDIX 2: DECLARATION OF HELSINKI

Recommendations guiding physicians in biomedical research involving human subjects

Adopted by the 18th World Medical Assembly,
Helsinki, Finland, June 1964
amended by the 29th World Medical Assembly
Tokyo, Japan, October 1975,
and
the 35th World Medical Assembly,
Venice, Italy, October 1983
and
the 41st World Medical Assembly,
Hong-Kong, September 1989
and
the 48th General Assembly,
Somerset West, Republic of South Africa, October 1996

INTRODUCTION

It is the mission of the physician to safeguard the health of the people. His or her knowledge and conscience are dedicated to the fulfilment of this mission.

The Declaration of Geneva of the World Medical Association binds the physician with the words, "The health of my patient will be my first consideration", and the International Code of Medical Ethics declares that, "A physician shall act only in patient's interest when providing medical care which might have the effect of weakening the physical and mental condition of the patient".

The purpose of biomedical research involving human subjects must be to improve diagnostic, therapeutic and prophylactic procedures and the understanding of the aetiology and pathogenesis of disease.

In current medical practice most diagnostic, therapeutic or prophylactic procedures involve hazards. This applies especially to biomedical research.

Medical progress is based on research which ultimately must rest in part on experimentation involving human subjects.

In the field of biomedical research a fundamental distinction must be recognised between medical research in which the aim is essentially diagnostic or therapeutic for a patient, and medical research, the essential object of which is purely scientific and without implying direct diagnostic or therapeutic value to the person subjected to the research.

Special caution must be exercised in the conduct of research which may affect the environment, and the welfare of animals used for research must be respected.

Because it is essential that the results of laboratory experiments be applied to human beings to further scientific knowledge and to help suffering humanity, the World Medical Association has prepared the following recommendations as a guide to every physician in biomedical research involving human subjects. They should be kept under review in the future. It must be stressed that the standards as drafted are only a guide to physicians all over the world. Physicians are not relieved from criminal, civil and ethical responsibilities under the laws of their own countries.

I - BASIC PRINCIPLES

1. Biomedical research involving human subjects must conform to generally accepted scientific principles and should be based on adequately performed laboratory and animal experimentation and on a thorough knowledge of the scientific literature.

2. The design and performance of each experimental procedure involving human subjects should be clearly formulated in an experimental protocol which should be transmitted for consideration, comment and guidance to a specially appointed committee independent of the investigator and the sponsor provided that this independent committee is in conformity with the laws and regulations of the country in which the research experiment is performed.

3. Biomedical research involving human subjects should be conducted only by scientifically qualified persons and under the supervision of a clinically competent medical person. The responsibility for the human subject must always rest with a medically qualified person and never rest on the subject of the research, even though the subject has given his or her consent.

4. Biomedical research involving human subjects cannot legitimately be carried out unless the importance of the objective is in proportion to the inherent risk to the subject.

5. Every biomedical research project involving human subjects should be preceded by careful assessment of predictable risks in comparison with foreseeable benefits to the subject or to others. Concern for the interests of the subject must always prevail over the interests of science and society.

6. The right of the research subject to safeguard his or her integrity must always be respected. Every precaution should be taken to respect the privacy of the subject to minimise the impact of the study on the subject's physical and mental integrity and on the personality of the subject.

7. Physicians should abstain from engaging in research projects involving human subjects unless they are satisfied that the hazards involved are believed to be predictable. Physicians should cease any investigation if the hazards are found to outweigh the potential benefits.

8. In publication of the results of his or her research, the physician is obliged to preserve the accuracy of the results. Reports of experimentation not in accordance with the principles laid down in this Declaration should not be accepted for publication.

9. In any research on human beings, each potential subject must be adequately informed of the aims, methods, anticipated benefits and potential hazards of the study and the discomfort it may entail. He or she should be informed that he or she is at liberty to abstain from participation in the study and that he or she is free to withdraw his or her consent to participation at any time. The physician should then obtain the subject's freely given informed consent, preferably in writing.

10. When obtaining informed consent for the research project the physician should be particularly cautious if the subject is in a dependent relationship to him or her or may consent under duress. In that case the informed consent should be obtained by a physician who is not engaged in the investigation and who is completely independent of this official relationship.

11. In case of legal incompetence, informed consent should be obtained from the legal guardian in accordance with national legislation. Where physical or mental incapacity makes it impossible to obtain informed consent, or when the subject is a minor, permission from the responsible relative replaces that of the subject in accordance with national legislation.

12. The research protocol should always contain a statement of the ethical considerations involved and should indicate that the principles enunciated in the present Declaration are complied with.

II - MEDICAL RESEARCH COMBINED WITH PROFESSIONAL CARE
(Clinical research)

1. In the treatment of the sick person, the physician must be free to use a new diagnostic and therapeutic measure, if in his or her judgement it offers hope of saving life, re-establishing health or alleviating suffering.

2. The potential benefits, hazards and discomfort of a new method should be weighed against the advantages of the best current diagnostic and therapeutic methods.

3. In any medical study, every patient - including those of a control group, if any - should be assured of the best proven diagnostic and therapeutic method. This does not exclude the use of inert placebo in studies where no proven diagnostic or therapeutic method exists.

4. The refusal of the patient to participate in a study must never interfere with the physician-patient relationship.

5. If the physician considers it essential not to obtain informed consent, the specific reasons for this proposal should be stated in the experimental protocol for transmission to the independent Committee (1,2).

6. The physician can combine medical research with professional care, the objective being the acquisition of new medical knowledge, only to the extent that medical research is justified by its potential diagnostic or therapeutic value for the patient.

III - NON-THERAPEUTIC BIOMEDICAL RESEARCH INVOLVING HUMAN SUBJECTS
(Non-clinical biomedical research)

1. In the purely scientific application of medical research carried out on a human being, it is the duty of the physician to remain the protector of the life and health of that person on whom biomedical research is being carried out.

2. The subjects should be volunteers - either healthy persons or patients for whom the experimental design is not related to the patient’s illness.

3. The investigator or the investigating team should discontinue the research if in his/her or their judgement it may, if continued, be harmful to the individual.

4. In research on man, the interest of science and society should never take precedence over considerations related to the well-being of the subject.

16.3 APPENDIX 3: MEASUREMENT OF DISEASE

Immune-Modified Response Evaluation Criteria In Solid Tumours: IMRECIST

Conventional response criteria may not be adequate to characterize the anti-tumor activity of immunotherapeutic agents like pembrolizumab, which can produce delayed responses that may be preceded by initial apparent radiographic progression, including the appearance of new lesions. Therefore, immune-modified response criteria have been developed to incorporate new lesions into the assessment of total tumor burden and allow radiographic progression to be confirmed at a subsequent assessment.

Immune-modified Response Evaluation Criteria in Solid Tumors (RECIST), as described within this appendix, were adapted from RECIST, Version 1.1 (v1.1) (Eisenhauer et al. 2009), in the same manner that immune-related response criteria were adapted from WHO criteria (Wolchok et al. 2009) and RECIST v1.0 (Nishino et al. 2014).˙ When not otherwise specified, RECIST v1.1 conventions will apply.

Differences between immune-modified RECIST and RECIST v1.1 are summarized in Table 1.

Table 1 : Comparison of RECIST v1.1 and Immune-Modified RECIST

	RECIST v1.1	Immune-Modified RECIST
Measurable new lesions	Always represent progression	Incorporated into the total tumor burden [a] and followed
Non-measurable new	Always represent progression	Do not represent progression, but lesions preclude CR
Non-target lesions	Contribute to defining CR, PR, SD, and PD	Contribute to defining CR only
CR	Disappearance of all lesions	Disappearance of all lesions
PR	≥30% decrease in sum of diameters of target lesions, in the absence of CR, new lesions, and unequivocal progression in non-target lesions	≥30% decrease in tumor burden, [a] in the absence of CR
PD	≥20% increase in sum of diameters of target lesions, unequivocal progression in non-target lesions, and/or appearance of new lesions	≥20% increase in tumor burden [a]
SD	Neither sufficient shrinkage to qualify for CR or PR nor sufficient increase to qualify for PD	Neither sufficient shrinkage to qualify for CR or PR nor sufficient increase to qualify for PD

CR = complete response; PD = progressive disease; PR = partial response; RECIST = Response Evaluation Criteria in Solid Tumors; SD = stable disease.

a = Tumor burden is the sum of diameters of target lesions and measurable new lesions.

TUMOR MEASURABILITY

At baseline, tumor lesions/lymph nodes will be categorized as measurable or non-measurable as described below. All measurable and non-measurable lesions should be assessed at screening and at subsequent protocol-specified tumor assessment timepoints. Additional assessments may be performed as clinically indicated for suspicion of progression.

DEFINITION OF MEASURABLE LESIONS

Tumor Lesions

Tumor lesions must be accurately measured in at least one dimension (longest diameter in the plane of measurement is to be recorded) with a minimum size as follows:

- 10 mm by computed tomography (CT) or magnetic resonance imaging (MRI) scan (CT/MRI scan slice thickness/interval $\leq$5 mm)

10-mm caliper measurement by clinical examination (lesions that cannot be accurately measured with calipers should be recorded as non-measurable)

Malignant Lymph Nodes

To be considered pathologically enlarged and measurable, a lymph node must be

$\geq$15 mm in the short axis when assessed by CT scan (CT scan slice thickness recommended to be $\leq$5 mm). At baseline and follow-up, only the short axis will be measured and followed. Additional information on lymph node measurement is provided below (see "Identification of Target and Non-Target Lesions," "New Lesions," and "Calculation of Sum of Diameters").

DEFINITION OF NON-MEASURABLE LESIONS

Non-measurable tumor lesions encompass small lesions (longest diameter $<$10 mm or pathological lymph nodes with short axis $\geq$10 mm but $<$15 mm) as well as truly

non-measurable lesions. Lesions considered truly non-measurable include leptomeningeal disease, ascites, pleural or pericardial effusion, inflammatory breast disease, lymphangitic involvement of skin or lung, peritoneal spread, and abdominal mass/abdominal organomegaly identified by physical examination that is not measurable by reproducible imaging techniques.

SPECIAL CONSIDERATIONS REGARDING LESION MEASURABILITY

Bone lesions, cystic lesions, and lesions previously treated with local therapy require particular comment, as outlined below.

Bone Lesions:

- Technetium-99m bone scans, sodium fluoride positron emission tomography scans, and plain films are not considered adequate imaging techniques for measuring bone lesions. However, these techniques can be used to confirm the presence or disappearance of bone lesions.
- Lytic bone lesions or mixed lytic-blastic lesions with identifiable soft tissue components that can be evaluated by cross-sectional imaging techniques such as CT or MRI can be considered measurable lesions if the soft tissue component meets the definition of measurability described above.
- Blastic bone lesions are non-measurable.

- For mCRPC patients, bone scans will be used to document the progression of metastatic bone disease using the PCWG3 criteria

Cystic Lesions:

- Lesions that meet the criteria for radiographically defined simple cysts should not be considered malignant lesions (neither measurable nor non-measurable) since they are, by definition, simple cysts.
- Cystic lesions thought to represent cystic metastases can be considered measurable lesions if they meet the definition of measurability described above. However, if non-cystic lesions are present in the same patient, these are preferred for selection as target lesions.

Lesions with Prior Local Treatment:

- Tumor lesions situated in a previously irradiated area or in an area subjected to other loco-regional therapy are usually not considered measurable unless there has been demonstrated progression in the lesion.

METHODS FOR ASSESSING LESIONS

All measurements should be recorded in metric notation, using calipers if clinically assessed. All baseline evaluations should be performed as close as possible to the treatment start and never more than 4 weeks before the beginning of the treatment.

The same method of assessment and the same technique should be used to characterize each identified and reported lesion at baseline and during the study. Imaging-based evaluation should always be the preferred option.

CLINICAL LESIONS

Clinical lesions will only be considered measurable when they are superficial and ≥10 mm in diameter as assessed using calipers (e.g., skin nodules). For the case of skin lesions, documentation by color photography, including a ruler to estimate the size of the lesion, is suggested.

CHEST X-RAY

Chest CT is preferred over chest X-ray, particularly when progression is an important endpoint, since CT is more sensitive than X-ray, particularly in identifying new lesions.

CT AND MRI SCANS

CT is the best currently available and reproducible method to measure lesions selected for response assessment. In this guideline, the definition of measurability of lesions on CT scan is based on the assumption that CT slice thickness is ≤5 mm. When CT scans have slice thickness of >5 mm, the minimum size for a measurable lesion should be twice the slice thickness. MRI is also acceptable.

If prior to enrollment it is known that a patient is unable to undergo CT scans with intravenous (IV) contrast because of allergy or renal insufficiency, the decision as to whether a non-contrast CT or MRI (without IV contrast) will be used to evaluate the patient at baseline and during the study should be guided by the tumour type under investigation and the anatomic location of the disease. For patients

who develop contraindications to contrast after baseline contrast CT is done, the decision as to whether non-contrast CT or MRI (enhanced or non-enhanced) will be performed should also be based on the tumour type and the anatomic location of the disease, and should be optimized to allow for comparison with the prior studies if possible. Each case should be discussed with the radiologist to determine if substitution of these other approaches is possible and, if not, the patient should be considered not evaluable from that point forward. Care must be taken in measurement of target lesions and interpretation of

non-target disease or new lesions on a different modality, since the same lesion may appear to have a different size using a new modality.

ENDOSCOPY, LAPAROSCOPY, ULTRASOUND, TUMOR MARKERS, CYTOLOGY, HISTOLOGY

Endoscopy, laparoscopy, ultrasound, tumor markers, cytology, and histology cannot be utilized for objective tumor evaluation.

ASSESSMENT OF TUMOR BURDEN

To assess objective response or future progression, it is necessary to estimate the overall tumour burden at baseline and use this as a comparator for subsequent measurements.

IDENTIFICATION OF TARGET AND NON-TARGET LESIONS

When more than one measurable lesion is present at baseline, all lesions up to a maximum of five lesions total (and a maximum of two lesions per organ) representative of all involved organs should be identified as target lesions and will be recorded and measured at baseline. This means that, for instances in which patients have only one or two organ sites involved, a maximum of two lesions (one site) and four lesions (two sites), respectively, will be recorded. Other lesions (albeit measurable) in those organs will be considered non-target lesions.

Target lesions should be selected on the basis of their size (lesions with the longest diameter) and be representative of all involved organs, but in addition should lend themselves to reproducible repeated measurements. It may be the case that, on occasion, the largest lesion does not lend itself to reproducible measurement, in which circumstance the next largest lesion that can be measured reproducibly should be selected.

Lymph nodes merit special mention since they are normal anatomical structures that may be visible by imaging even if not involved by tumor. As noted above, pathological nodes that are defined as measurable and may be identified as target lesions must meet the criterion of a short axis of ≥15 mm by CT scan. Only the short axis of these nodes will contribute to the baseline sum. The short axis of the node is the diameter normally used by radiologists to judge if a node is involved by solid tumor. Lymph node size is normally reported as two dimensions in the plane in which the image is obtained (for CT, this is almost always the axial plane; for MRI, the plane of acquisition may be axial, sagittal, or coronal). The smaller of these measures is the short axis. For example, an abdominal node that is reported as being 20 mm × 30 mm has a short axis of 20 mm and qualifies as a malignant, measurable node. In this example, 20 mm should be recorded as the node measurement. All other pathological nodes (those with short axis ≥10 mm but <15 mm) should be considered non-target lesions. Nodes that have a short axis of

<10 mm are considered non-pathological and should not be recorded or followed.

All lesions (or sites of disease) not selected as target lesions (measurable or non-measurable), including pathological lymph nodes, should be identified as non-target lesions and should also be recorded at baseline. Measurements are not required. It is possible to record multiple non-target lesions involving the same organ as a single item on the electronic Case Report Form (eCRF) (e.g., "multiple enlarged pelvic lymph nodes" or "multiple liver metastases").

NEW LESIONS

New lesions identified after baseline will be evaluated for measurability with use of the same criteria applied to prospective target lesions at baseline per RECIST (e.g., non–lymph node lesions must be ≥10 mm on the longest diameter; new lymph nodes must be ≥15 mm on the short axis [see note below]). All new lesions (measurable or non-measurable) must be assessed and recorded at the time of identification and at all subsequent tumor assessment timepoints. Up to a maximum of five measurable new lesions total (and a maximum of two lesions per organ) can be included in the calculation of tumor burden that is performed as part of the tumor response evaluation. New lesion types that would not qualify as target lesions per RECIST cannot be included in the calculation of tumor burden and thus will not affect overall tumor response evaluation. New lesions that are not measurable at first appearance but meet measurability criteria at a subsequent timepoint can be included in the tumor response evaluation from that point on, if the maximum number of measurable new lesions has not been reached.

Note regarding new lymph node lesions: If at first appearance the short axis of a lymph node lesion is ≥15 mm, it will be considered a measurable new lesion. If at first appearance the short axis of a lymph node lesion is ≥10 mm and <15 mm, the lymph node will not be considered measurable but will still be considered a new lesion and should be identified as a non-measurable new lesion. If at first appearance the short axis of a lymph node is <10 mm, the lymph node should not be considered pathological and should not be considered a new lesion. A lymph node can subsequently become measurable, when the short axis is ≥15 mm.

CALCULATION OF SUM OF DIAMETERS

A sum of the diameters (longest diameter for non–lymph node lesions, short axis for lymph node lesions) will be calculated for all target lesions at baseline as a measure of tumor burden. At each subsequent tumor assessment, a sum of the diameters (longest diameter for non-lymph node lesions, short axis for lymph node lesions) will be calculated for all target lesions plus measurable new lesions (up to five new lesions, with a maximum of two new lesions per organ) that have emerged after baseline. Hence, each net percentage change in tumor burden per assessment accounts for the size and growth kinetics of both old lesions and new lesions as they appear.

Measuring Lymph Nodes

If at first appearance the short axis of a new lymph node lesion is ≥15 mm, it will be considered a measurable new lesion and may be included in the sum of the diameters. If the new lymph node lesion is included in the sum of diameters, it will continue to be measured and included in the sum of diameters at subsequent timepoints, even if the short axis decreases to <15 mm (or even <10 mm). However, if it subsequently decreases to <10 mm and all other lesions are no longer detectable or have also decreased to a short axis of <10 mm (if lymph nodes), a response assessment of complete response may be assigned.

Lymph nodes should always have the actual short axis measurement recorded (measured in the same anatomical plane as the baseline examination), even if the node regresses to $<$10 mm during the study. Thus, when lymph nodes are included in the sum of diameters, the sum may not be zero even if CR criteria are met, since a normal lymph node is defined as having a short axis of $<$10 mm.

Measuring Lesions That Become Too Small to Measure

During the study, all target lesions and up to five measurable new lesions (lymph node and non–lymph node) should have their actual measurements recorded at each subsequent evaluation, even when very small (e.g., 2 mm). However, sometimes lesions or lymph nodes become so faint on CT scan that the radiologist may not feel comfortable assigning an exact measurement and may report them as being too small to measure. When this occurs, it is important that a value be recorded on the eCRF, as follows:

- If it is the opinion of the radiologist that the lesion has likely disappeared, the measurement should be recorded as 0 mm.
- If the lesion is believed to be present and is faintly seen but too small to measure, a default value of 5 mm should be assigned and "too small to measure" should be ticked. (Note: It is less likely that this rule will be used for lymph nodes since they usually have a definable size when normal and are frequently surrounded by fat such as in the retroperitoneum; however, if a lymph node is believed to be present and is faintly seen but too small to measure, a default value of 5 mm should be assigned in this circumstance as well and "too small to measure" should also be ticked).

To reiterate, however, if the radiologist is able to provide an actual measurement, that should be recorded, even if it is $<$5 mm, and in that case "too small to measure" should not be ticked.

Measuring Lesions That Split or Coalesce on Treatment

When non–lymph node lesions fragment, the longest diameters of the fragmented portions should be added together to calculate the sum of diameters. Similarly, as lesions coalesce, a plane between them may be maintained that would aid in obtaining maximal diameter measurements of each individual lesion. If the lesions have truly coalesced such that they are no longer separable, the vector of the longest diameter in this instance should be the maximum longest diameter for the coalesced lesion.

EVALUATION OF NON-TARGET LESIONS AND NON-MEASURABLE NEW LESIONS

Measurements are not required for non-target lesions or non-measurable new lesions. Non-target lesions should be noted at baseline, and non-measurable new lesions should be noted at the time of identification. At subsequent evaluations, non-target lesions and non-measurable new lesions will be categorized as "present" or "absent."

After baseline, changes in non-target lesions or non-measurable new lesions (or measurable new lesions in excess of five total or two per organ) will contribute only in the assessment of CR (i.e., a CR is attained only with the complete disappearance of all tumor lesions, including non-target lesions and non-measurable new lesions) and will not be used to assess progressive disease.

RESPONSE CRITERIA

Definitions of the criteria used to determine objective tumor response are provided below:

- CR: Disappearance of all lesions

  Any pathological lymph nodes must have reduction in short axis to <10 mm.

- PR: At least a 30% decrease in the sum of diameters of all target lesions plus measurable new lesions (up to a maximum of five total or two per organ), taking as reference the baseline sum of diameters, in the absence of CR

- Progressive disease (PD): At least a 20% increase in the sum of diameters of all target lesions plus measurable new lesions (up to a maximum of five total or two per organ), taking as reference the smallest sum of diameters on study (including baseline)

  In addition to the relative increase of 20%, the sum of diameters must also demonstrate an absolute increase of ≥ 5 mm.

New lesions alone do not qualify as progressive disease. However, their contribution to total tumor burden is factored into the sum of the diameters, which is used to determine the overall immune-modified RECIST tumor response.

Stable disease (SD): Neither sufficient shrinkage to qualify for CR or PR nor sufficient increase to qualify for PD

CRITERIA FOR OVERALL RESPONSE AT A SINGLE TIMEPOINT

Table 2 provides a summary of the overall response status calculation at each response assessment timepoint for patients who have measurable disease at baseline.

Table 2 Criteria for Overall Response at a Single Timepoint: Patients with Target Lesions (with or without Non-Target Lesions)

Target Lesions and Measurable New Lesions [a]	Non-Target Lesions and Non-Measurable New Lesions [b]	Overall Response
CR	Absent	CR
CR	Present or not all evaluated	PR
PR	Any	PR
SD	Any	SD
Not all evaluated	Any	NE
PD	Any	PD

CR = complete response; NE = not evaluable; PD = progressive disease; PR = partial response; SD = stable disease.

[a] Up to a maximum of five measurable new lesions total (and a maximum of two lesions per organ) can be included in the calculation of tumor burden, in addition to the target lesions identified at baseline.

[b] Also includes measurable new lesions in excess of five total or two per organ.

MISSING ASSESSMENTS AND NOT-EVALUABLE DESIGNATION

When no imaging/measurement is done at all at a particular timepoint, the patient is not evaluable at that timepoint. If measurements are made on only a subset of target or measurable new lesions at a timepoint, usually the case is also considered not evaluable at that timepoint, unless a convincing argument can be made that the contribution of the individual missing lesions would not change the assigned timepoint response. This would be most likely to happen in the case of PD. For example, if

a patient had a baseline sum of 50 mm with three measured lesions and during the study only two lesions were assessed, but those gave a sum of 80 mm, the patient will have achieved PD status, regardless of the contribution of the missing lesion.

SPECIAL NOTES ON RESPONSE ASSESSMENT

Patients with a global deterioration in health status requiring discontinuation of treatment without objective evidence of disease progression at that time should be reported as "symptomatic deterioration." Every effort should be made to document objective progression even after discontinuation of treatment. Symptomatic deterioration is not a descriptor of an objective response; it is a reason for stopping study therapy. The objective response status of such patients is to be determined by evaluation of target and non-target lesions, as well as new lesions, as shown in Table 1.

REFERENCES

Eisenhauer EA, Therasse P, Bogaerts J, et al. New response evaluation criteria in solid tumors: revised RECIST guideline (version 1.1). Eur J Cancer 2009;45:228–47.

Wolchok JD, Hoos A, O'Day S, et al. Guidelines for the evaluation of immune therapy activity in solid tumors: immune-related response criteria. Clin Can Res 2009;15:7412-20.

Nishino M, Gargano M, Suda M, et al. Optimizing immune-related tumor response assessment: does reducing the number of lesions impact response assessment in melanoma patients treated with ipilimumab? J Immunother Can 2014;2:17.

16.4 APPENDIX 4: NEW YORK HEART ASSOCIATION (NYHA) SCALE

Class I – patients with cardiac disease but without resulting limitation of physical activity; ordinary physical activity does not cause undue dyspnoea (or fatigue, palpitation or anginal pain)

Class II – patients with cardiac disease resulting in slight limitation of physical activity; they are comfortable at rest; ordinary physical activity results in dyspnoea (or fatigue, palpitation or anginal pain)

Class III – patients with cardiac disease resulting in marked limitations of physical activity; they are comfortable at rest; less than ordinary physical activity causes dyspnoea (or fatigue, palpitation or anginal pain)

Class IV – patients with cardiac disease resulting in inability to carry out physical activity without discomfort; symptoms of dyspnoea (or of angina) may be present even at rest; if any physical activity is undertaken, discomfort is increased.

16.5 APPENDIX 5: CONTRACEPTIVE GUIDANCE AND PREGNANCY TESTING

Woman of Childbearing Potential (WOCBP)

A woman is considered fertile following menarche and until becoming post-menopausal unless permanently sterile (see below)

Women in the following categories are not considered WOCBP:

- Premenarchal
- Premenopausal female with 1 of the following:
  - Documented hysterectomy
  - Documented bilateral salpingectomy
  - Documented bilateral oophorectomy

  Note: Documentation can come from the site personnel’s review of the participant’s medical records, medical examination, or medical history interview.

- Postmenopausal female
  - A postmenopausal state is defined as no menses for 12 months without an alternative medical cause.
    - A high follicle stimulating hormone (FSH) level in the postmenopausal range may be used to confirm a postmenopausal state in women not using hormonal contraception or hormonal replacement therapy (HRT). However, in the absence of 12 months of amenorrhea, confirmation with two FSH measurements in the postmenopausal range is required.
  - Females on HRT and whose menopausal status is in doubt will be required to use one of the non-hormonal highly effective contraception methods if they wish to continue their HRT during the study. Otherwise, they must discontinue HRT to allow confirmation of postmenopausal status before study enrollment.

Contraception Requirements

Male Participants:

Male participants with female partners of childbearing potential are eligible to participate if they agree to one of the following during the protocol defined time frame:

- Be abstinent from penile-vaginal intercourse as their usual and preferred lifestyle (abstinent on a long term and persistent basis) and agree to remain abstinent
- Use a male condom plus partner use of a contraceptive method with a failure rate of <1% per year as described in Table 1 when having penile-vaginal intercourse with a woman of childbearing potential who is not currently pregnant.
  - Note: Men with a pregnant or breastfeeding partner must agree to remain abstinent from penile-vaginal intercourse or use a male condom during each episode of penile penetration.

Female Participants:

Female participants of childbearing potential are eligible to participate if they agree to use a highly effective method of contraception consistently and correctly as described in Table 1 during the protocol-defined time frame.

Table 1 Highly Effective Contraception Methods

**Highly Effective Contraceptive Methods That Are User Dependent [a]**<br>*Failure rate of <1% per year when used consistently and correctly.*
● Combined (estrogen- and progestogen- containing) hormonal contraception [b, c]<br>○ Oral<br>○ Intravaginal<br>○ Transdermal<br>○ Injectable
● Progestogen-only hormonal contraception [b, c]<br>○ Oral<br>○ Injectable
**Highly Effective Methods That Have Low User Dependency**<br>*Failure rate of <1% per year when used consistently and correctly.*
● Progestogen- only contraceptive implant [b, c]<br>● Intrauterine hormone-releasing system (IUS) [b]<br>● Intrauterine device (IUD)<br>● Bilateral tubal occlusion
● **Vasectomized partner**<br>A vasectomized partner is a highly effective contraception method provided that the partner is the sole male sexual partner of the WOCBP and the absence of sperm has been confirmed. If not, an additional highly effective method of contraception should be used.
● **Sexual abstinence**<br>Sexual abstinence is considered a highly effective method only if defined as refraining from heterosexual intercourse during the entire period of risk associated with the study treatment. The reliability of sexual abstinence needs to be evaluated in relation to the duration of the study and the preferred and usual lifestyle of the participant.)
Notes:<br>Use should be consistent with local regulations regarding the use of contraceptive methods for participants of clinical studies.<br><br>a) Typical use failure rates are lower than perfect-use failure rates (i.e. when used consistently and correctly).<br><br>b) If hormonal contraception efficacy is potentially decreased due to interaction with study treatment, condoms must be used in addition to the hormonal contraception during the treatment period and for at least [X days, corresponding to time needed to eliminate study treatment plus 30 days for study treatments with genotoxic potential] after the last dose of study treatment.<br>c) If locally required, in accordance with Clinical Trial Facilitation Group (CTFG) guidelines, acceptable hormonal contraceptives are limited to those which inhibit ovulation.

Pregnancy Testing

WOCBP should only be included after a negative highly sensitive urine or serum pregnancy test.

Following initiation of treatment, pregnancy testing will be performed whenever an expected menstrual cycle is missed or when pregnancy is otherwise suspected; at the time points specified in the Schedule of Events, and as required locally.

Pregnancy testing will be performed whenever an expected menstrual cycle is missed or when pregnancy is otherwise suspected.

16.6 APPENDIX 6: ADVERSE EVENTS: DEFINITIONS AND PROCEDURES FOR RECORDING, EVALUATING, FOLLOW UP AND REPORTING

16.6.1 Definition of AE

AE definition

- An AE is any untoward medical occurrence in a clinical study participant, temporally associated with the use of study intervention, whether or not considered related to the study intervention.
- NOTE: An AE can therefore be any unfavorable and unintended sign (including an abnormal laboratory finding), symptom, or disease (new or exacerbated) temporally associated with the use of a study intervention.
- NOTE: For purposes of AE definition, study intervention (also referred to as Merck product) includes any pharmaceutical product, biological product, vaccine, diagnostic agent, or protocol specified procedure whether investigational or marketed (including placebo, active comparator product, or run-in intervention), manufactured by, licensed by, provided by, or distributed by Merck for human use in this study.

Events meeting the AE definition

- Any abnormal laboratory test results (hematology, clinical chemistry, or urinalysis) or other safety assessments (eg, ECG, radiological scans, vital signs measurements), including those that worsen from baseline, considered clinically significant in the medical and scientific judgment of the investigator.
- Exacerbation of a chronic or intermittent pre-existing condition including either an increase in frequency and/or intensity of the condition.
- New conditions detected or diagnosed after study intervention administration even though it may have been present before the start of the study.
- Signs, symptoms, or the clinical sequelae of a suspected drug-drug interaction.
- Signs, symptoms, or the clinical sequelae of a suspected overdose of either study intervention or a concomitant medication.
- For all reports of overdose (whether accidental or intentional) with an associated AE, the AE term should reflect the clinical symptoms or abnormal test result. An overdose without any associated clinical symptoms or abnormal laboratory results is reported using the terminology “accidental or intentional overdose without adverse effect.”

Events NOT meeting the AE definition

- Medical or surgical procedure (eg, endoscopy, appendectomy): the condition that leads to the procedure is the AE.
- Situations in which an untoward medical occurrence did not occur (social and/or convenience admission to a hospital).

- Anticipated day-to-day fluctuations of pre-existing disease(s) or condition(s) present or detected at the start of the study that do not worsen.
- Surgery planned prior to informed consent to treat a pre-existing condition that has not worsened.

16.6.2 Definition of SAE

If an event is not an AE per definition above, then it cannot be an SAE even if serious conditions are met.

An SAE is defined as any untoward medical occurrence that, at any dose:

a. Results in death

b. Is life-threatening

- The term “life-threatening” in the definition of “serious” refers to an event in which the participant was at risk of death at the time of the event. It does not refer to an event, which hypothetically might have caused death, if it were more severe.

c. Requires inpatient hospitalization or prolongation of existing hospitalization

- Hospitalization is defined as an inpatient admission, regardless of length of stay, even if the hospitalization is a precautionary measure for continued observation. (Note: Hospitalization for an elective procedure to treat a pre-existing condition that has not worsened is not an SAE. A pre-existing condition is a clinical condition that is diagnosed prior to the use of a Merck product and is documented in the participant’s medical history.

d. Results in persistent or significant disability/incapacity

- The term disability means a substantial disruption of a person’s ability to conduct normal life functions.
- This definition is not intended to include experiences of relatively minor medical significance such as uncomplicated headache, nausea, vomiting, diarrhea, influenza, and accidental trauma (eg, sprained ankle) that may interfere with or prevent everyday life functions but do not constitute a substantial disruption.

e. Is a congenital anomaly/birth defect

- In offspring of participant taking the product regardless of time to diagnosis.

f. Other important medical events

- Medical or scientific judgment should be exercised in deciding whether SAE reporting is appropriate in other situations such as important medical events that may not be immediately life-threatening or result in death or hospitalization but may jeopardize the participant or may require medical or surgical intervention to prevent 1 of the other outcomes listed in the above definition. These events should usually be considered serious.

- Examples of such events include invasive or malignant cancers, intensive treatment in an emergency room or at home for allergic bronchospasm, blood dyscrasias or convulsions that do not result in hospitalization, or development of drug dependency or drug abuse.

16.6.3 Additional Events Reported in the Same Manner as SAE

In addition to the above criteria, AEs meeting either of the below criteria, although not serious per ICH definition, are reportable to Merck and Astex in the same time frame as SAEs to meet certain local requirements. Therefore, these events are considered serious by Merck and Astex for collection purposes.

- Is a new cancer (that is not a condition of the study)
- Is associated with an overdose of pembrolizumab

16.6.4 Recording AE and SAE

AE and SAE recording

- When an AE/SAE occurs, it is the responsibility of the investigator to review all documentation (eg, hospital progress notes, laboratory, and diagnostics reports) related to the event.
- The investigator will record all relevant AE/SAE information on the AE CRFs/worksheets at each examination.
- There may be instances when copies of medical records for certain cases are requested by the Merck. In this case, all participant identifiers, with the exception of the participant number, will be blinded on the copies of the medical records before submission to the Merck.
- The investigator will attempt to establish a diagnosis of the event based on signs, symptoms, and/or other clinical information. In such cases, the diagnosis (not the individual signs/symptoms) will be documented as the AE/SAE.

Assessment of intensity/toxicity

- An event is defined as “serious” when it meets at least 1 of the predefined outcomes as described in the definition of an SAE, not when it is rated as severe.

1. The investigator will make an assessment of intensity for each AE and SAE (and other reportable safety event) according to the NCI Common Terminology for Adverse Events (CTCAE), version 5. Any AE that changes CTCAE grade over the course of a given episode will have each change of grade recorded on the AE CRFs/worksheets.
    - Grade 1: Mild; asymptomatic or mild symptoms; clinical or diagnostic observations only; intervention not indicated.
    - Grade 2: Moderate; minimal, local or noninvasive intervention indicated; limiting age-appropriate instrumental activities of daily living (ADL).
    - Grade 3: Severe or medically significant but not immediately life-threatening; hospitalization or prolongation of hospitalization indicated; disabling; limiting self-care ADL.

- Grade 4: Life threatening consequences; urgent intervention indicated.
- Grade 5: Death related to AE.

Assessment of causality

1. Did Merck or Astex product cause the AE?

2. The determination of the likelihood that Merck or Astex product caused the AE will be provided by an investigator who is a qualified physician. The investigator's signed/dated initials on the source document or worksheet that supports the causality noted on the AE form, ensures that a medically qualified assessment of causality was done. This initialed document must be retained for the required regulatory time frame. The criteria below are intended as reference guidelines to assist the investigator in assessing the likelihood of a relationship between the test product and the AE based upon the available information.

3. The following components are to be used to assess the relationship between Merck's or Astex's product and the AE; the greater the correlation with the components and their respective elements (in number and/or intensity), the more likely Merck or Astex product caused the AE:

- **Exposure:** Is there evidence that the participant was actually exposed to Merck or Astex product such as: reliable history, acceptable compliance assessment (pill count, diary, etc.), expected pharmacologic effect, or measurement of drug/metabolite in bodily specimen?

- **Time Course:** Did the AE follow in a reasonable temporal sequence from administration of Merck product? Is the time of onset of the AE compatible with a drug-induced effect (applies to studies with investigational medicinal product)?

- **Likely Cause:** Is the AE not reasonably explained by another etiology such as underlying disease, other drug(s)/vaccine(s), or other host or environmental factors.

- **Dechallenge:** Was Merck or Astex product discontinued or dose/exposure/frequency reduced?

- If yes, did the AE resolve or improve?

- If yes, this is a positive dechallenge.

- If no, this is a negative dechallenge.

- (Note: This criterion is not applicable if: (1) the AE resulted in death or permanent disability; (2) the AE resolved/improved despite continuation of the Merck or Astex product; (3) the study is a single-dose drug study; or (4) Merck or Astex product(s) is/are only used 1 time.)

- **Rechallenge:** Was the participant re-exposed to Merck or Astex product in this study?

- If yes, did the AE recur or worsen?

- If yes, this is a positive rechallenge.

- If no, this is a negative rechallenge.

(Note: This criterion is not applicable if: (1) the initial AE resulted in death or permanent disability, or (2) the study is a single-dose drug study; or (3) Merck or Astex product(s) is/are used only 1 time.)

NOTE: IF A RECHALLENGE IS PLANNED FOR AN AE THAT WAS SERIOUS AND MAY HAVE BEEN CAUSED BY MERCK OR ASTEX PRODUCT, OR IF RE-EXPOSURE TO MERCK'S OR ASTEX's PRODUCT POSES ADDITIONAL POTENTIAL SIGNIFICANT RISK TO THE PARTICIPANT THEN THE RECHALLENGE MUST BE APPROVED IN ADVANCE BY THE SPONSOR AS PER DOSE MODIFICATION GUIDELINES IN THE PROTOCOL, AND IF REQUIRED, THE INIRB/IEC.

4. **Consistency with study intervention profile:** Is the clinical/pathological presentation of the AE consistent with previous knowledge regarding Merck or Astex product or drug class pharmacology or toxicology?

5. The assessment of relationship will be reported on the case report forms/worksheets by an investigator who is a qualified physician according to his/her best clinical judgment, including consideration of the above elements.

6. Use the following scale of criteria as guidance (not all criteria must be present to be indicative of Merck or Astex product relationship).

   - Yes, there is a reasonable possibility of Merck or Astex product relationship:
   - There is evidence of exposure to the Merck or Astex product. The temporal sequence of the AE onset relative to the administration of Merck or Astex product is reasonable. The AE is more likely explained by Merck or Astex product than by another cause.
   - No, there is not a reasonable possibility of Merck or Astex product relationship:
   - Participant did not receive the Merck or Astex product OR temporal sequence of the AE onset relative to administration of the Merck or Astex product is not reasonable OR the AE is more likely explained by another cause than the Merck or Astex product. (Also entered for a participant with overdose without an associated AE.)

7. For each AE/SAE, the investigator must document in the medical notes that he/she has reviewed the AE/SAE and has provided an assessment of causality.

8. There may be situations in which an SAE has occurred and the investigator has minimal information to include in the initial report to the Merck and Astex. However, it is very important that the investigator always make an assessment of causality for every event before the initial transmission of the SAE data to Merck and Astex.

9. The investigator may change his/her opinion of causality in light of follow-up information and send an SAE follow-up report with the updated causality assessment.

10. The causality assessment is 1 of the criteria used when determining regulatory reporting requirements.

11. For studies in which multiple agents are administered as part of a combination regimen, the investigator may attribute each AE causality to the combination regimen or to a single agent of the combination. In general, causality attribution should be assigned to the combination regimen (ie, to all agents in the regimen). However, causality attribution may be assigned to a single agent if in the investigator's opinion, there is sufficient data to support full attribution of the AE to the single agent.

Follow-up of AE and SAE

- The investigator is obligated to perform or arrange for the conduct of supplemental measurements and/or evaluations as medically indicated to elucidate the nature and/or causality of the AE or SAE as fully as possible. This may include additional laboratory tests or investigations, histopathological examinations, or consultation with other health care professionals.
- New or updated information will be recorded in the CRF.
- The investigator will submit any updated SAE data to Merck and Astex within 2 business days but no longer than 3 calendar days of receipt of the information.

16.6.5 Reporting of AEs, SAEs, and Other Reportable Safety Events to Merck and Astex

SAE reports and any other relevant safety information are to be forwarded to the Merck Global Safety email inbox pv.uk@merck.com and to Astex drug safety group at drugsafety@astx.com by the sponsor.

A copy of all 15 Day Reports and Annual Progress Reports is submitted as required by MHRA, European Union (EU), Pharmaceutical and Medical Devices agency (PMDA) or other local regulators. Investigators will cross reference this submission according to local regulations to the Merck Investigational Compound Number (IND, CSA, etc.) at the time of submission. Additionally, sponsor will submit a copy of these reports to Merck & Co., Inc. (Attn: Worldwide Product Safety; email pv.uk@merck.com) at the time of submission to MHRA.

All initial and follow-up AEs, SAEs, and other reportable safety events will be recorded and reported to Merck & Astex within the time frames as indicated in Table 1 below.

Table 1. Reporting Time Periods and Time Frames for Adverse Events and Other Reportable Safety Events

**Type of Event**	**Reporting Time Period:** **Consent to Randomization/ Allocation**	**Reporting Time Period:** **Randomization/ Allocation through Protocol-specified Follow-up Period**	**Reporting Time Period:** **After the Protocol-specified Follow-up Period**	**Time Frame to Report Event and Follow-up Information to Merck & Astex**
Serious Adverse Event (SAE) including Cancer and Overdose	Report if:<br>- due to protocol-specified intervention<br>- causes exclusion<br>- participant is receiving placebo	Report all	Report if:<br>- drug/vaccine related.<br>(Follow ongoing to outcome)	Within 2 business days but no longer than 3 calendar days of learning of

Type of Event	<u>Reporting Time Period:</u> Consent to Randomization/ Allocation	<u>Reporting Time Period:</u> Randomization/ Allocation through Protocol-specified Follow-up Period	<u>Reporting Time Period:</u> After the Protocol-specified Follow-up Period	Time Frame to Report Event and Follow-up Information to Merck & Astex
	run-in or other run-in treatment			event
Pregnancy/Lactation Exposure	Report if:<br>- due to intervention<br>- causes exclusion	Report all	Previously reported – Follow to completion/termination; report outcome	Within 2 business days but no longer than 3 calendar days of learning of event
Event of Clinical Interest (require regulatory reporting)	Report if:<br>- due to intervention<br>- causes exclusion	Report<br>- potential drug-induced liver injury (DILI)<br>- require regulatory reporting	Not required	Within 2 business days but no longer than 3 calendar days of learning of event

16.7 APPENDIX 7: RANO CRITRIA FOR GLIOBLASTOMAS AND HIGH GRADE GLIOMAS

Source: Wen PY, Macdonald DR, Reardon DA, Cloughesy TF, Sorensen AG, Galanis E, Degroot J, Wick W, Gilbert MR, Lassman AB, Tsien C, Mikkelsen T, Wong ET, Chamberlain MC, Stupp R, Lamborn KR, Vogelbaum MA, van den Bent MJ, Chang SM. Updated response assessment criteria for high-grade gliomas: response assessment in neuro-oncology working group. J Clin Oncol. 2010;28(11):1963-72. http://jco.ascopubs.org/content/28/11/1963.full

Criteria for Response Assessment Incorporating MRI and Clinical Factors

Response	Criteria
Complete response	Requires all of the following: complete disappearance of all enhancing measurable and nonmeasurable disease sustained for at least 4 weeks; no new lesions; stable or improved nonenhancing (T2/FLAIR) lesions; patients must be off corticosteroids (or on physiologic replacement doses only); and stable or improved clinically. Note: Patients with nonmeasurable disease only cannot have a complete response; the best response possible is stable disease.
Partial response	Requires all of the following: ≥ 50% decrease compared with baseline in the sum of products of perpendicular diameters of all measurable enhancing lesions sustained for at least 4 weeks; no progression of nonmeasurable disease; no new lesions; stable or improved nonenhancing (T2/FLAIR) lesions on same or lower dose of corticosteroids compared with baseline scan; the corticosteroid dose at the time of the scan evaluation should be no greater than the dose at time of baseline scan; and stable or improved clinically. Note: Patients with nonmeasurable disease only cannot have a partial response; the best response possible is stable disease.

Stable disease	Requires all of the following: does not qualify for complete response, partial response, or progression; stable nonenhancing (T2/FLAIR) lesions on same or lower dose of corticosteroids compared with baseline scan. In the event that the corticosteroid dose was increased for new symptoms and signs without confirmation of disease progression on neuroimaging, and subsequent follow-up imaging shows that this increase in corticosteroids was required because of disease progression, the last scan considered to show stable disease will be the scan obtained when the corticosteroid dose was equivalent to the baseline dose.
Progression	Defined by any of the following: ≥ 25% increase in sum of the products of perpendicular diameters of enhancing lesions compared with the smallest tumor measurement obtained either at baseline (if no decrease) or best response, on stable or increasing doses of corticosteroids*; significant increase in T2/FLAIR nonenhancing lesion on stable or increasing doses of corticosteroids compared with baseline scan or best response after initiation of therapy* not caused by comorbid events (eg, radiation therapy, demyelination, ischemic injury, infection, seizures, postoperative changes, or other treatment effects); any new lesion; clear clinical deterioration not attributable to other causes apart from the tumor (eg, seizures, medication adverse effects, complications of therapy, cerebrovascular events, infection, and so on) or changes in corticosteroid dose; failure to return for evaluation as a result of death or deteriorating condition; or clear progression of nonmeasurable disease.

NOTE. All measurable and nonmeasurable lesions must be assessed using the same techniques as at baseline.
MRI, magnetic resonance imaging; FLAIR, fluid-attenuated inversion recovery.
* Stable doses of corticosteroids include patients not on corticosteroids.

Summary of the Proposed RANO Response Criteria

Criterion	CR	PR	SD	PD
T1 gadolinium enhancing disease	None	≥ 50% ↓	< 50% ↓ but < 25% ↑	≥ 25% ↑*
T2/FLAIR	Stable or ↓	Stable or ↓	Stable or ↓	↑*
New lesion	None	None	None	Present*
Corticosteroids	None	Stable or ↓	Stable or ↓	NA†
Clinical status	Stable or ↑	Stable or ↑	Stable or ↑	↓*
Requirement for response	All	All	All	Any*

RANO, Response Assessment in Neuro-Oncology; CR, complete response; PR, partial response; SD, stable disease; PD, progressive disease; FLAIR, fluid-attenuated inversion recovery; NA, not applicable.
*Progression occurs when this criterion is present.
† Increase in corticosteroids alone will not be taken into account in determining progression in the absence of persistent clinical deterioration.

16.8 APPENDIX 8: iRANO CRITERIA

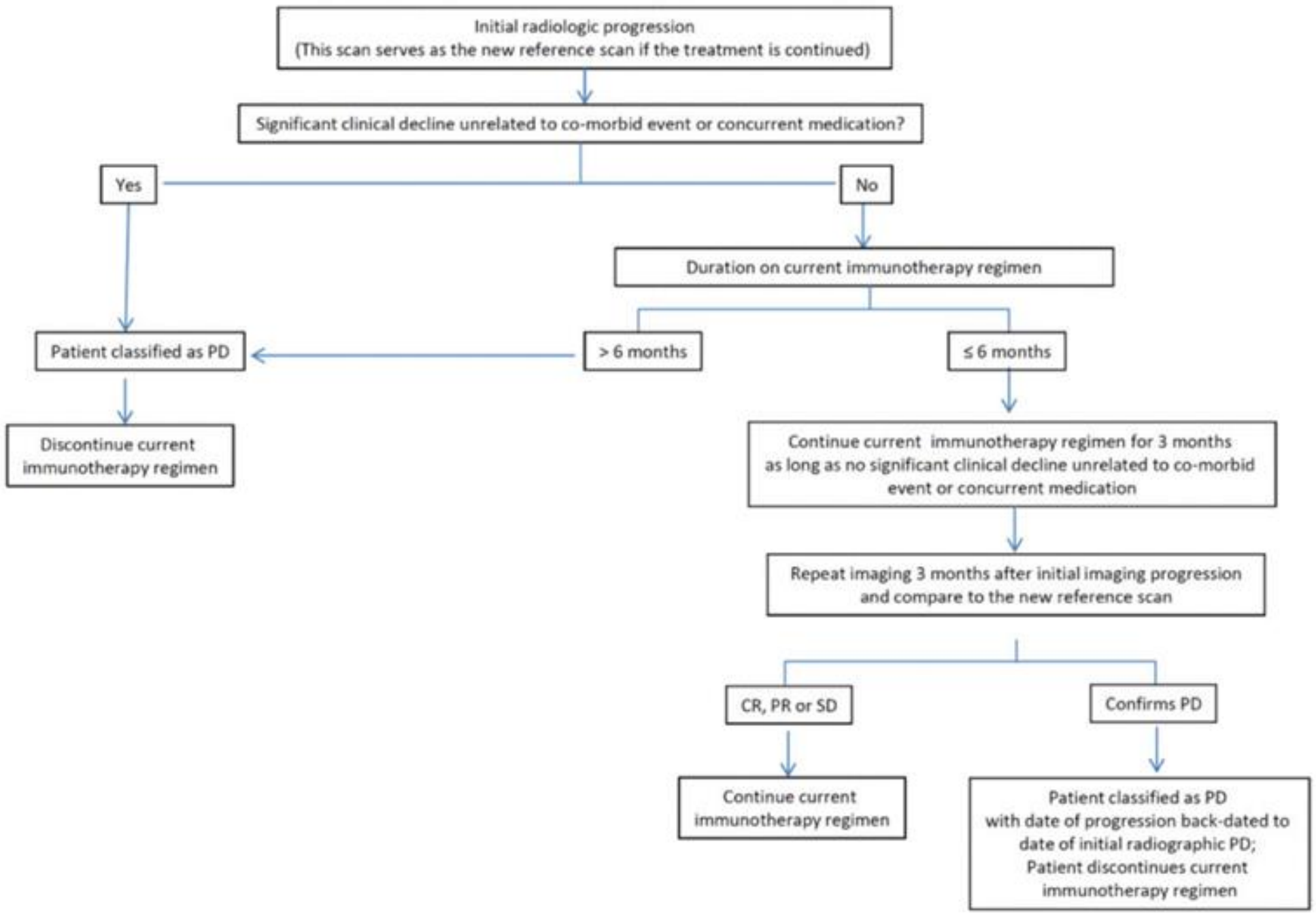

Source: Hideho Okada, MD, Michael Weller, MD, Raymond Huang, MD, Gaetano Finocchiaro, MD, Mark R. Gilbert, MD, Wolfgang Wick, MD, Benjamin M. Ellingson, PhD, Naoya Hashimoto, MD, Ian F. Pollack, MD, Alba A. Brandes, MD, Enrico Franceschi, MD, Christel Herold-Mende, PhD, Lakshmi Nayak, MD, Ashok Panigrahy, MD, Whitney B. Pope, MD, Robert Prins, PhD, John H. Sampson, MD, Patrick Y. Wen, MD, and David A. Reardon, MD. Immunotherapy Response Assessment in Neuro-Oncology (iRANO): A Report of the RANO Working Group. Lancet Oncol. 2015 November ; 16(15): e534–e542. doi:10.1016/S1470-2045(15)00088-1. Lancet Oncol. 2015 November ; 16(15): e534–e542. doi:10.1016/S1470-2045(15)00088-1.

ASTEROID: A Phase I trial of tolinapant (ASTX660) in combination with Pembrolizumab (MK-3475) in patients with solid tumours: utilizing triple IAP blockade as a strategy to maximize Immunogenic cell Death and the generation of an efficient adaptive immune response

Statistical Analysis Plan

clinical data – efficacy/safety analyses

Version 1.0

19-Dec-2024

This statistical analysis plan is based on protocol version 4.0 [22-Sep-2023]

IRAS Number: 280601

Sponsor Reference Number: CCR5199

EUDRACT number: 020-004648-28

All approved versions of the SAP will be stored in the Statistical Section of the Trial Master File.

This statistical analysis plan is a framework to guide statistical analysis and may be supplemented by additional and exploratory analyses, after discussion with the ICR-CTSU Scientific/Methodology Lead and the Chief Investigator. Trial statisticians can amend analysis methods as appropriate after discussion with the ICR-CTSU Scientific/Methodology Lead.

For the final version to be used for the principal analysis of the primary endpoint only:

This statistical analysis plan has been approved by the following personnel:

Senior Trial Statistician: Dr Xiaoran Lai

Signed: ______________________________ **Date:** 14 January 2025

ICR-CTSU Scientific/Methodology Lead: Prof Christina Yap

Signed: ______________________________ **Date:** 14/01/2025

Chief Investigator: Dr Juanita Lopez

Signed: ______________________________ **Date:** 13 January 2025

Table 1 - Document history

Version	Date	Author	Changes made* (*including justifications and timing of SAP revisions in relation to trial milestones)
0.1.0	2022-02-23	Ben Jenkins	Initial draft of the SAP
0.2.0	2023-11-17	Xiaoran Lai	Redo in R Markdown. Added statistical analysis plan for all endpoints.
0.3.0	2024-02-05	Xiaoran Lai	Tabulated trial objectives and endpoints. Major revision to the definition of analysis population previously not included in this SAP to align them with the protocol and all endpoint analyses.
0.4.0	2024-02-27	Xiaoran Lai	Incorporated comments from J Rekowski and C Yap.
0.5.0	2024-06-29	Xiaoran Lai	Incorporated comments from DDU and updated to protocol v4
1.0.0	2024-12-19	Xiaoran Lai	Updated wording on cover page

1 Introduction

1.1 Scope

This statistical analysis plan (SAP) details the principal statistical analyses of the ASTEROID trial conducted by the ICR-CTSU Early Phase and Adaptive Trials Team. The SAP will be used to guide statistical analysis and to minimise bias due to data-driven choices of analysis. Once approved, the SAP will be stored in the electronic and paper Trial Master File and can be viewed on request. Any deviations from the analysis plan will be documented in the Statistical Analysis Report. Analyses of pharmacodynamics and pharmacokinetics are outlined in the Laboratory Manual. This version of the SAP covers the statistical analyses for dose-recommendation, safety data and efficacy outcomes for the dose escalation and expansion cohorts.

1.2 Trial background and rationale

1.2.1 IAPs at the cross roads of cell-death and cancer immunity

Cellular inhibitor of apoptosis protein 1/2 (cIAP1/2) and X-linked inhibitor of apoptosis protein (XIAP) are key members of the inhibitor of apoptosis (IAP) protein family that function as important gatekeepers of cell death and pro-survival inflammatory and immunogenic signalling pathways [1]. Although best known for their ability to regulate caspases, IAPs also influence ubiquitin-dependent pathways regulating the 'Ripoptosome' – a receptor-interacting protein kinase 1 (RIPK1)-dependent cytosolic signalling platform responsible for the integration of cellular stress, innate immune signalling pathways as well as cell death [2, 3, 4, 5]. This leads to the orchestration of inflammatory signalling including NF-κB activation which are critical for eliciting efficient T cell cross-priming [6].

In response to TNFα, cIAPs ubiquitinate RIPK1, promoting the formation of complexes which activate survival signalling through the canonical NF-κB pathway. Simultaneously, formation of the death-inducing signalling complex (DISC), which drives apoptosis, is prevented. Removal of cIAP1/2 allows the DISC to form, leading overall to a switch in TNFα signalling from pro-survival to proapoptotic. Once loss of cIAP1/2 is combined with release of the XIAP-mediated block on caspases, which is essential for full activation of apoptosis, a sustained proapoptotic effect in the presence of TNFα is obtained. Antagonism and subsequent degradation of the cIAP1/2 also leads to the stabilization of NIK (NF-κB-inducing kinase), which activates the non-canonical NF-κB pathway, resulting in enhancement of T-cell co-stimulation [7].

1.2.2 Smac mimetics in combination with PDL-1 inhibitors

Small-molecule antagonists of the IAPs, known as Smac mimetic compounds, bind to IAPs, preventing their binding to caspases, but also inducing the auto-ubiquitination and subsequent proteasomal-mediated degradation of these IAPs. Smac-mediated antagonism of IAPs sensitizes cancer cells to death ligands originating from the immune system through the formation of the ripoptosome [2]. In parallel, Smac mimetic mediated depletion of IAPs in immune cells leads to the activation of the NF-κB pathway which is postulated to produce co-stimulatory signals that stimulate dendritic cell maturation [8] and efficient cross-priming of effector T cells [6] (Figure 1).

Preclinical models testing first generation Smac mimetics in combination with anti-PD-1 have shown significant efficacy with durable cures in syngeneic mouse models of cancer [9].

ASTX660 is a potent, non-peptidomimetic, antagonist of cIAP1, cIAP2 and XIAP. Combined antagonism of cIAP1/2 and XIAP may offer improved efficacy over first-generation Smac mimetics that are more cIAP1/2 selective. ASTX660 has been shown to have tumor growth inhibitory activity and potent proapoptotic activity in nonclinical models (see Section 4.1 of IB). ASTX660 is being developed in a first-in-human (FIH) phase I-II study for the treatment of patients with advanced solid tumors or lymphoma and for whom standard life-prolonging measures are not available (NCT02503423).

As such, it is postulated that ASTX660 may more efficiently push cancer cells into dying by necroptosis or inflammatory apoptosis; and equally more efficiently initiate an adaptive immune response that could be augmented by immune-checkpoint blockade.

ASTX660 has been combined in vivo with anti-PD-L1 in a murine syngeneic tumour model CT26. Significant tumour regression and enhanced survival was seen with the combination regardless of single agent ASTX660 or anti-PD-L1 activity (Astex, in house data). Combination trials of first generation cIAP1/2 selective antagonists together with immune checkpoint inhibitors are ongoing (NCT03270176, NCT02890069).

1.2.3 Rationale for the proposed trial

ASTX660 has been shown to have tumour growth inhibitory activity and potent proapoptotic activity in nonclinical models, with a more balanced inhibitory profile for both compared with other first-generation Smac mimetics that are more cIAP1/2 selective (Refer to IB Section 4.1) It is therefore expected to exhibit more efficient tumour cell death by necroptosis or inflammatory apoptosis; and equally more efficiently initiate an adaptive immune response that could be augmented by combining with anti-PD-1 agent pembrolizumab. This trial will investigate more in depth the mechanisms by which ASTX660 elicits its therapeutic effects both on tumour and on the host immune system. This will be critical to determine the best strategy to pursue in future later

stage tumour specific trials of IAP antagonists in combination with immunotherapy, and to ensure appropriate molecular stratification biomarkers for the greatest benefit to patients.

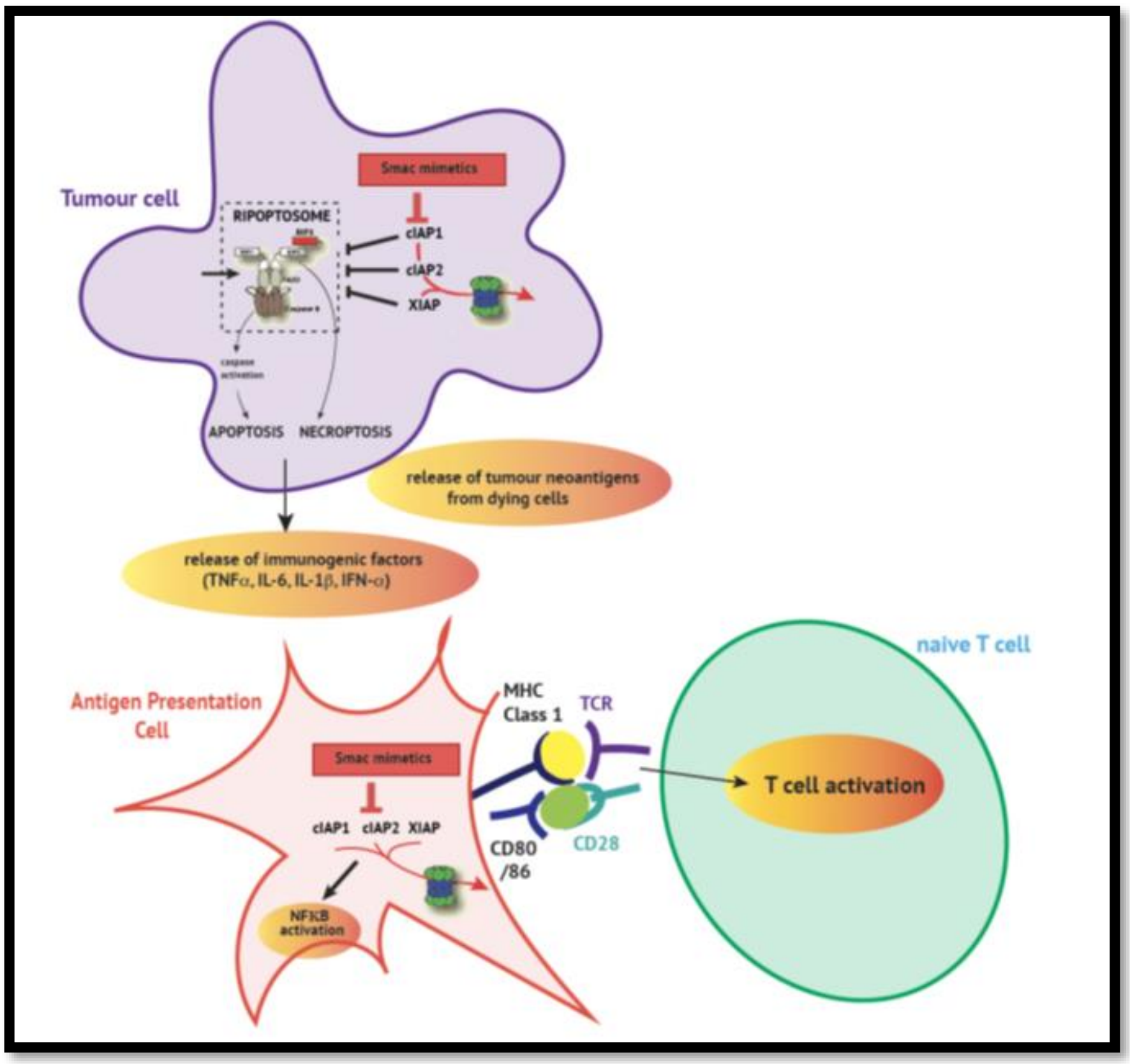

Figure 1 – (top) Redundancy of cIAP1/2 and XIAP in 'guarding' the ripoptosome. Inhibition of CIAP1/cIAP2 as well as XIAP by ASTX660 is hypothesized to increase tumour immunogenic cell death by more efficient activation of the ripoptosome. Together with the activation of NfKB, this promotes dendritic cell activation enhancing antigen presentation and the generation of an efficient immune response.

2 Trial Design and Methods

2.1 Trial design

This is prospective, open label, multi-centre phase I non-randomised TITE-CRM study, consisting of an initial dose escalation phase and followed by an expansion phase.

2.1.1 Part A - Dose escalation

The aim of the first part of this study is to establish the maximum tolerated dose (MTD) and recommended phase 2 dose (RP2D) of ASTX660 and pembrolizumab when given in combination in patients with advanced solid tumours.

In Part A, escalating doses of ASTX660 will be investigated in combination with a fixed dose of pembrolizumab (200mg). The starting dose of ASTX660 is 90mg orally once a day which is the dose shown to have pharmacodynamics effects in depleting IAPs in a phase I single agent study.

ASTX660 administration will follow a 7 day on / 14 day off pattern (ie dosing for 7 consecutive days leading up to administration of the immune check-point inhibitors). Therefore in Cycle 1, ASTX660 will be given alone from Day 1 to Day 7, followed by a 14 day break, then taken again in Cycle 2 . Each cycle will run for a period of 21 days, with ASTX660 taken always on Days 1 to 7 and pembrolizumab given always on Day 8. Paired tumour biopsies and blood samples will be collected for analysis of pharmacodynamic biomarkers. Up to 4 dose levels of ASTX660 will be explored (Table 2).

Table 2 – Schema of dose escalation

Dose level	ASTX660 (mg)	pembrolizumab (mg)
1	90	200
2	120	200
3	150	200
4	180	200

2.1.2 Part B - Dose expansion

Patients will be accrued to the expansion cohorts in Part B in order to further assess the safety and tolerability of the combination and to obtain a preliminary signal of efficacy. Patients in Part B will be treated at the RP2D as determined by the SRC after the initial dose escalation (Part A) has been completed. Safety, tolerability and occurrence of DLT will continually be monitored and the RP2D will be adjusted if necessary. The expansion cohorts have statistically justified sample sizes and predefined criteria to inform a three-outcome decision of go, no-go or inconclusive to guide further testing of the combinational therapy.

The treatment schedule will be the same as that for Part A, i.e. each cycle will be 21 days-long. pembrolizumab will always be administered on day 8 of every cycle. ASTX660 will be taken daily on days 1-7.

Part B will consist of 5 cohorts; Cohort B1, Cohort B2, Cohort B3, Cohort B4, and Cohort B5

- **Cohort B1**: Patients with immune-checkpoint refractory tumours (n = 10)
- **Cohort B2**: Patients with cervical cancer (n = 10)
  Patients with PD-L1 positive tumours who have progressed on prior Immuno-Oncology (IO) treatments will go straight onto combination therapy. Patients with PD-L1 negative tumours,

or those who are IO-naïve, will have a run-in with pembrolizumab and switch to combination at progression.

- **Cohort B3**: Patients with triple negative breast cancer (n=10)
- Patients who have progressed on prior IO treatments will go straight onto combination therapy. Patients who are IO-naïve will have a run-in with pembrolizumab and switch to combination at progression.
- **Cohort B4:** Patients with relapsed Glioblastoma (n=10)
- **Cohort B5:** Patients with ER positive HER2 negative breast cancer (n=10) who have progressed on CDK4/6 inhibitor therapy

2.2 Trial objectives and endpoints

2.2.1 Primary objectives and endpoints

Table 3 - Overview of primary objectives and endpoints

Objectives	Endpoints	Part
To propose a recommended dose for Phase II (RP2D) evaluation by establishing the maximum tolerated dose (MTD) of ASTX660 in combination with Pembrolizumab.	To propose a recommended dose for Phase II (RP2D) evaluation by establishing the maximum tolerated dose (MTD) of ASTX660 in combination with Pembrolizumab.	A
To assess the safety and tolerability of ASTX660 and Pembrolizumab when given in combination.	Determining the frequency, severity and causality of each adverse event to the combination of ASTX660 and Pembrolizumab according to the National Cancer Institute (NCI) Common Terminology Criteria for Adverse Events (CTCAE) Version 5.0.	A and B
To evaluate the preliminary anti-tumour activity of ASTX660 in combination with Pembrolizumab.	Disease response by imRECIST criteria, clinical benefit rate, best changes in tumour size, progression-free survival and duration of response.	B

2.2.2 Secondary objectives and endpoints

Table 4 - Overview of secondary objectives and endpoints

Objectives	Endpoints	Part
To evaluate the preliminary anti-tumour activity of ASTX660 in combination with Pembrolizumab.	Disease response by imRECIST criteria, clinical benefit rate, best changes in tumour size, progression-free survival and duration of response.	A
To characterise the pharmacokinetics (PK) of ASTX660 in the escalation phase.	Determination of the plasma levels of ASTX660 using validated assays.	A

2.2.3 Tertiary objectives and endpoints

Table 5 - Overview of tertiary objectives and endpoints

Objectives	Endpoints	Part
To characterise the pharmacodynamics (PD) profile of single agent ASTX660 and the combination of ASTX660 and pembrolizumab on the host immune response, during the escalation phase, using paired blood and plasma samples.	Determination of changes in immune cell population (using FACS) and host immune cell cytokine profile with single agent ASTX660 and the combination of ASTX660 and Pembrolizumab.	A
To characterise the pharmacodynamics profile of single agent ASTX660 and the combination of ASTX660 and Pembrolizumab in pre- and post-treatment tumour biopsies.	Determination of changes in the tumour microenvironment including changes in tumour infiltrating lymphocytes (TILs), myeloid derived suppressor cells (MDSCs) and other immune cells.	A and B
To assess putative predictive biomarkers of response and resistance to the doublet combination.	Determination of changes in exploratory biomarkers including but not limited to immune transcriptome profile changes, PD-L1 expression and tumour lysate cytokine profiling in pre- and post-treatment tumour biopsies.	A and B
To evaluate the effect of ASTX660 on the tumour microenvironment including the evaluation of inflammatory cytokines downstream of NF-kappaB in paired tumour biopsies.	Determination of changes in exploratory biomarkers including but not limited to immune transcriptome profile changes, PD-L1 expression and tumour lysate cytokine profiling in pre- and post-treatment tumour biopsies.	A and B
To evaluate changes in patient reported outcomes from baseline using the EORTC QLQ-C30.	HRQoL will be assessed using the global score of the EORTC.	A and B
To estimate the overall survival (OS) in patients receiving the combination of pembrolizumab and ASTX660.	OS will be measured from the date of addition of ASTX660 to pembrolizumab to the date of death.	A and B

2.3 Sample size and power

Up to 68 evaluable patients will be enrolled onto the study. For part A, a target sample size of 18 evaluable patients will be recruited to Part A to enable adequate collection of paired evaluable tumour and blood samples for pharmacodynamic analyses, and this target sample size is anticipated to be sufficient to determine the MTD as per the trial design. for Part B: Dose Expansion, which may recruit a further 50 patients in 5 separate cohorts (10 patients per cohort). The operating characteristics of the trial design can be found in the statistical simulation plan.

2.4 Randomisation/recruitment procedures

Patients will be enrolled following completion of an eligibility checklist. No randomisation will take place.

2.5 Dose escalation scheme

2.5.1 Intra-patient dose escalation

Intra-patient dose escalation for Part A is not allowed. The SRC may recommend intra-patient dose escalations in Part B, but not exceeding the MTD.

2.5.2 Inter-patient dose escalation

The dose escalation will utilize a flexible 2 stage Time to event Continual Reassessment Method (TITE-CRM) including time-to-toxicity analysis to account for dose-limiting toxicities that may arise outside the DLT period. The aim of the escalation phase will be to obtain the MTD of ASTX660 in combination with a fixed dose of pembrolizumab associated with a dose limiting toxicity (DLT) set at a target of 25%.

The TITE-CRM design uses a weighted measure based on DLT follow-up period, and has the attractive feature of not requiring (or minimising) enrolment suspension while patients are still being observed for DLT [10]. It is particularly useful to account for potential late onset toxicities where the DLT may occur beyond the typical 21-28 days cycle period. This design not only allows for a full DLT assessment period (like the CRM) but it also allows the flexibility of dose decisions once patients have been followed-up for a minimum assessment period. In the TITE-CRM paradigm, patients who have started treatment but have not experienced DLT will be included in the probability calculation with a weigh equal to the proportion of the full DLT assessment period. This feature is applicable for this trial, where we would like to capture DLT occurrence up to 6 weeks from start of treatment. For instance, a patient who has not experienced DLT at 4 weeks will be included in the model as having a weight of 4/6 of no DLT. Patients who experience DLT or completed the 6-week DLT assessment period without DLT will be assigned full weight. A further advantage of this design is that it will be able to include partial information on patients who could not be formally evaluated for DLTs due to withdrawal, treatment discontinuation or death that is unrelated to treatment within the DLT assessment period.

In the first stage of the TITE-CRM design, patients will be recruited in cohorts of two with a starting dose at Dose level 1. If no DLT occurs, escalation to the next higher dose may take place once each patient has at least 3 weeks of DLT assessment period. If we observe no DLT for the first 8 patients (at Dose levels 1, 2, 3 and 4), subsequent patients will be recruited continuously at

the highest dose (Dose level 4). Once the first DLT occurs, we will proceed to the second stage, where the TITE-CRM model will be used to recommend the best dose for subsequent patients with all the information accumulated so far. A minimum DLT follow-up of 2 weeks will be imposed between patients. Intra-patient dose escalation is not allowed.

Once the MTD has been established, patients will be recruited to the expansion cohort, Part B. Safety will continually be monitored and the RP2D may be adjusted if necessary.

2.6 Dose expansion scheme

A Bayesian decision framework with a three-outcome design (No Go, Inconclusive and Go) based on the clinical benefit rate (see definition in section 9.2.3) will be used. Based on previous trials using single agent of IO (including pembrolizumab), the clinical benefit rate is expected to be <5% for the patient population to be recruited into this trial (cohorts B1-B5).

The decision criteria will be based on the posterior probability that the clinical benefit rate in the combination therapy is greater than 5%. The underlying true clinical benefit rate is assessed using a beta-binomial conjugate analysis. A weakly informative prior of Be(0.2,0.8) with an equivalent sample size of 1 is used. This gives a clinical benefit rate of 20% with an equivalent sample size of 1. In other words, before the trial starts, we are adding in one equivalent patient data with a mean activity rate of 20%. The 3-outcome decision criteria is based on

$$\text{ProbA} = P(\text{true clinical benefit rate} > 5\% \mid \text{data})$$

with the threshold boundaries as follows:

- GO: A high probability (≥90%) that the clinical benefit rate is greater than 5% will indicate that there is a clear signal that the treatment is active, or
- NO-GO: A low probability (≤12%) will provide a clear signal that the treatment is not active, otherwise
- Inconclusive: There is no clear evidence (12% < Prob < 90%) to conclude if it is active or not active

This is analogous to the three-outcome design in the frequentist settings for Phase II single arm clinical trials proposed by Hong and Wang [11], and randomised Phase II screened selection design by Yap, Pettitt, and Billingham [12]. We will however explore this in a Bayesian framework here, which is particularly useful as the sample size in each expansion cohort is small and is not fixed (ranging from 10 to 15 patients).

The exact number is dependent on the number of evaluable patients dosed at the RP2D in the dose-escalation phase. Those patients will contribute to the activity evaluation in the expansion

phase. Simulation results in Table 6 indicate a favourable performance of this design even with a small sample size of n = 10.

The recommended decision that will be recommended if we see n_R responders out of n patients can be tabulated in advance. Table 6 presents the posterior probabilities and decision that will be undertaken for all possible number of responders out of 10 and 15 patients, respectively. From Table 6, if we see 0 responders out of 10 patients, the posterior probability that the true clinical benefit rate is >5% is 0.11, the decision is NO GO. If we see at least 2 responders out of 10 patients, the decision is GO. Otherwise if it is 1/10 responders, it is INCONCLUSIVE.

Table 6 - Posterior probabilities that the true clinical benefit rate is greater than 5% given the observed data (ProbA) using a 3-outcome Bayesian decision framework, with the decision of either GO (ProbA = 0.9), NO GO (ProbA = 0.12) or INCONCLUSIVE (0.12 < ProbA < 0.9).

Number of patients, N	Observed Number of Responses, n_R	P(true clinical benefit rate > 5% | data)	Decision
10	0	0.11	No Go
10	1	0.69	Inconclusive
10	2	0.94	Go
10	3-10	> 0.99	Go
15	0	0.07	No Go
15	1	0.56	Inconclusive
15	2	0.87	Inconclusive
15	3	0.97	Go
15	4-15	> 0.99	Go

Table 7 - Operating characteristics of the 3-outcome Bayesian design for 10 patients, with 10,000 simulations, based on the decision criterion of Prob A = P(true clinical benefit rate > 5% | data), where GO: ProbA = 0.9, NO GO: ProbA = 0.12 and INCONCLUSIVE otherwise.

	Probability of recommending		
**Characteristic**	**Go**	**Inconclusive**	**No Go**
True clinical benefit rate			
0.03	0.04	0.23	0.74
0.2	0.63	0.27	0.1
0.3	0.85	0.12	0.03
0.4	0.95	0.04	0.01

We have chosen to adopt a Bayesian approach to decision-making as it provides greater flexibility for real-life evaluation due to the uncertainty regarding the eventual number in each expansion cohort. Each expansion cohort will be analysed and reported independently.

For each cohort, the posterior probabilities that the true clinical benefit rate is greater than 5% will be presented with the corresponding Bayesian decision outcome, as demonstrated in Table 7. Posterior clinical benefit rates with credible intervals will also be reported.

2.6.1 Further Safety (MTD) Evaluation

During the expansion phase where up to an additional 50 patients may be recruited in 5 specific cohorts, we will continue to update the TITE-CRM model after every subsequent 5-10 patients enrolled during the expansion phase, taking into account all the accumulated DLT data gathered thus far. This will enable us to use the additional safety data to determine if the initial MTD (based on the first 18 DLT-evaluable patients) remains the same. If the model recommends any adjustment of the recommended dose as the data accumulate, the Safety Review Committee will review the data, and subsequent patients may receive the updated dose. Simulations comparing the performance of the TITE CRM with 18 patients' vs 68 patients' data are included in the statistical simulation plan.

2.7 Dose modifications, delays and reductions

Details and reasons for permitted dose modifications, delays and reductions are described in Section 5.6 of the protocol.

2.8 Definition of dose limiting toxicity (DLT)

Details on the definition of DLT are described in Section 3.3 of the protocol.

2.9 Definition of maximum tolerated dose (MTD), biologically active dose (BAD) and recommended phase 2 dose (RP2D)

The MTD is defined as the dose of ASTX660 in combination with a fixed dose of pembrolizumab with estimated DLT rate close to a target DLT rate of 25%, as recommended by the TITE-CRM model.

The BAD is not defined in the protocol and will be determined by the totality of the data including pharmacokinetic, pharmacodynamic and efficacy data seen.

The RP2D will be either the MTD or a dose below as recommended by the SRC taking into account the toxicity profile seen as well as the emerging pharamacokinetic, pharamacodynamic and efficacy data.

3 Eligibility criteria

Inclusion and exclusion criteria for the trial are described in detail in Sections 4.1.1 and 4.1.2 of the protocol, respectively.

4 Data monitoring committee and interim analyses

The Safety Review Committee (SRC) and Investigator(s) will regularly review the safety data. Monthly SRC meetings will be planned, where the trial statistician will be expected to produce a comprehensive dose recommendation report on all of the DLT data accumulated thus far. If no further DLTs have occurred since the previous update, the meeting will go ahead as per the monthly schedule. If no further DLTs have occurred and the dose recommendation is to remain at the current dose, this may be communicated to the SRC team via email in which the decision may be to cancel the meeting, and continue recruiting patients at the current dose level. If a patient experiences a DLT, this will trigger an earlier SRC meeting, and the model will be updated to allow for potential dose de-escalation. An SRC meeting may be cancelled if, since the previous model update, no DLTs have occurred and there has been no further patient follow-up within the 6-week DLT assessment period (Figure 2).

The SRC will consist of:

- Chief Investigator (CI) (or delegated DDU consultant)
- Principal Investigators (PI) from each participating site (or delegated clinician)
- DDU Clinical Trials Manager (CTM) managing the study
- Trial Statistician(s)
- Observer from the Pharmaceutical Company providing the IMP (optional)
- DDU Pharmacovigilance Officer
- External, senior ECMC clinical who is independent of the study team

Further internal or external experts may be consulted by the SRC as necessary.

The SRC will review and assess all DLT and available safety data, the TITE-CRM's recommended dose, together with available pharmacokinetic and pharmacodynamic data to make a decision on the appropriate dose for the next cohort of patients. Any dose interruptions and reductions will be taken into account. The decision may be to:

- Proceed with dose escalation
- Expand the cohort to further explore that dose level (to a maximum of 6 evaluable patients)
- De-escalate the dose: to a previous lower dose level (to a maximum of 6 evaluable patients)

- Explore different dosing schedules
- Stop the dose escalation phase of the study
- De-escalate the dose during the dose expansion

The decisions and decision-making of the SRC on the next dose level will be documented and provided to the investigators prior to dosing any new patients.

The dose recommendation report produced for each TITE-CRM model update will primarily consist of the following items:

- A table consisting of one row per patient recruited so far, with summaries of their data utilised by the TITE CRM model. This includes, DLT vs. no DLT, status of DLT (suspected vs confirmed), length of DLT follow up period observed and corresponding weight parameter, and evaluability data.
- Updated dose toxicity curve illustrating the current posterior probability of DLT at each dose level.
- If deemed valuable/relevant, a dose transition pathway for the TITE-CRM will be included, illustrating the length of further follow-up time with no DLT that would be required in order to recommend to escalate the dose.

Consideration will be given to DLTs that are only suspected at the time of the model update. The model may be updated twice, once considering the event to be a confirmed DLT, and again without this being a DLT (and subsequently the patient is weighted for the length of follow up observed without DLT). If two patients' have suspected DLTs, this would result in four different permutations of the model update. If more than two patients have suspected DLTs, only the two extreme cases will be considered of all DLTs vs. no DLTs, as the number of permutations becomes too large. This is however highly unlikely to occur, and it is expected that only one patient may have a suspected DLT at a time.

During the expansion phase, the SRC will review accumulated data and the model updates from the TITE-CRM (which will be conducted after 5-10 and every subsequent 10 patients enrolled).

A schema illustrating the expected trial flow for model updates and SRC meetings in both stage one and stage two of the escalation phase is provided in Figure 2.

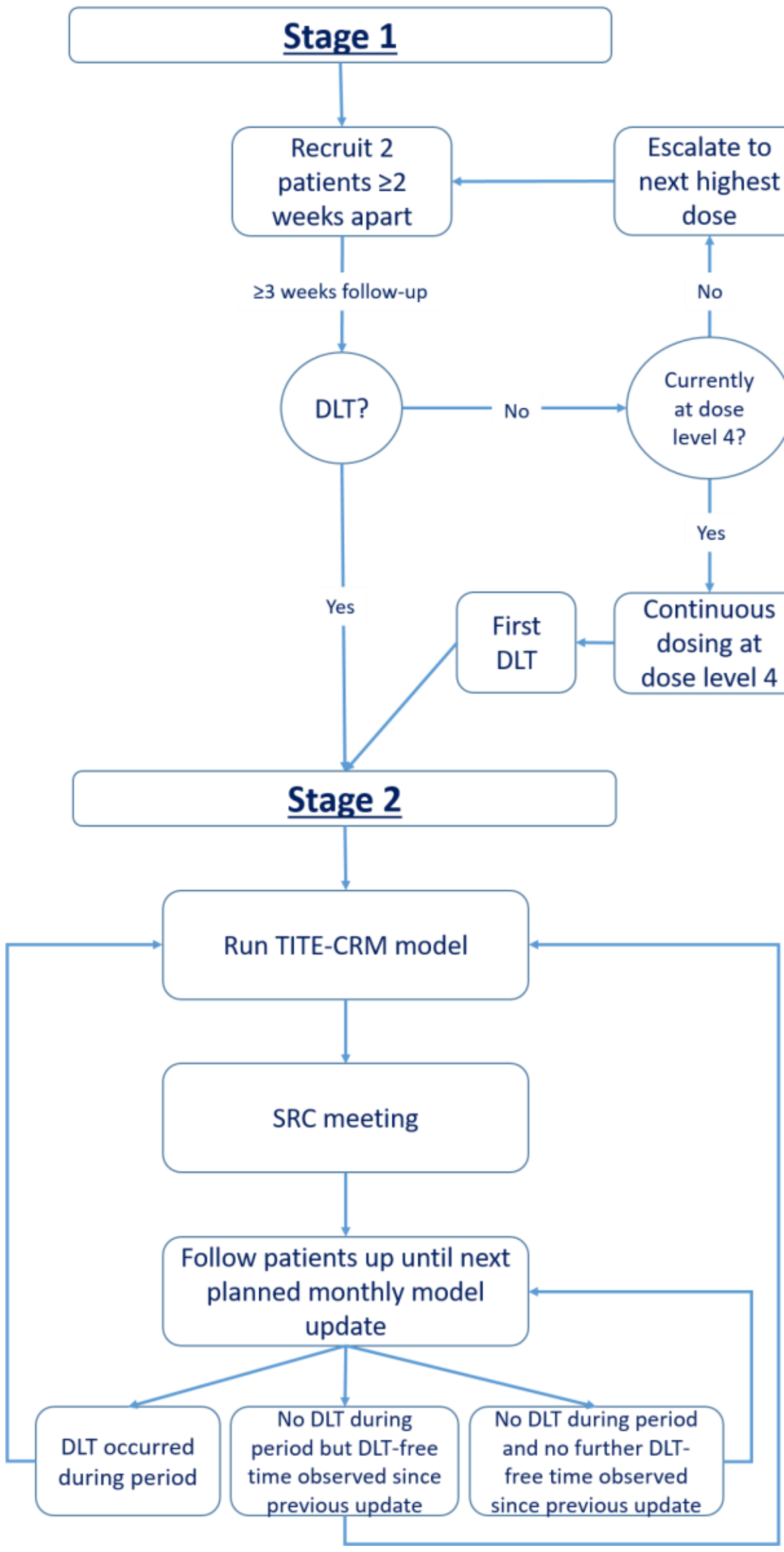

Figure 2 - ASTEROID TITE CRM schema for model updates and SRC meetings

5 Data completeness/consistency

5.1 Missing data

It is expected that the majority of missing data will be missing at random and therefore it is not expected that imputation methods will be used for individual patients or variables.

6 Patient disposition and follow-up

6.1 Patient flow through trial/recruitment

Patient flow through the trial will be described including the number of patients entered into and evaluable in each cohort and the number of patients who discontinued along with the reasons for discontinuation.

6.2 Treatment compliance

Treatment administration will be described for all cycles. Dose administration, dose modifications or delays and the duration of therapy will be described. In the dose escalation cohorts, listings will be provided for each dose with a line per-patient, per- cycle indicating doses received and any changes from originally prescribed dose/schedule. For each expansion cohort, data will be summarised by cycle indicating the number of patients at each cycle with changes to the originally prescribed dose. Reasons for treatment discontinuations will be tabulated for each of the cohorts.

6.3 Follow-up

Median follow-up will be calculated using the reverse Kaplan-Meier (KM) approach - censoring deceased patients at their time of death.

7 Analysis populations

7.1 Safety population

This population includes any enrolled patient who fulfilled the eligibility criteria and received at least one dose of study medication at any dose level.

7.2 Dose recommendation decision and MTD determination population

Patients who have completed the DLT period or at least 2 weeks of DLT follow up and are still being observed for DLT. Patients who are still being monitored for DLT or who have come off within the DLT period for any reason other than toxicity will be included in the probability calculation with a weight equal to the proportion of the full DLT assessment period using the TITE-CRM design.

Patients who have experienced a DLT within the DLT period or have received at least 75% of the specified dose of ASTX660 (i.e. 5 doses of ASTX660) and 1 dose of Pembrolizumab during the first cycle will be evaluable for determination of MTD (initially at the end of Part A as well as the end of Part B).

7.3 Response-evaluable population (REP)

All patients who meet the eligibility criteria, receive at least two cycles of combination treatment and have a baseline assessment of disease will be evaluable for response by imRECIST.

To be assigned a status of complete response (CR) or partial response (PR), changes in tumour measurements must be confirmed by repeat measurements performed no less than four weeks after the response criteria are met; To be assigned a status of stable disease (SD), follow-up measurements must have met the SD criteria at least once and at least six weeks after the initial dose of the investigational medicinal product (IMP) ASTX660 or Pembrolizumab is given.

For patients in cohorts B2 and B3 (IO naïve), the scan prior to the start of combination treatment will be used as the baseline scan.

8 Timing of the analysis

8.1 Principal analysis of the primary endpoint

The final analyses will be carried out only at the end of the dose expansion phase, and if one of the following conditions is met: - The trial is terminated early (for example, due to toxicity), or - All patients have had the opportunity to receive one cycle of treatment and have completed their 'off-study' visit (i.e. 28 days after the last dose of ASTX660 or 90 days after the last dose of pembrolizumab).

Once one of the conditions is met, a data cut-off date will be established. All patient visits occurring on or before this date will be analysed and summarised in the final clinical study report. Any data collected after this date will be summarised in a supplemental report.

8.2 Subgroup analyses

No subgroup analyses are planned for this trial.

Any unplanned subgroup analyses not mentioned in this analysis plan will be treated as exploratory and hypothesis generating.

9 Analysis methods

9.1 Baseline characteristics

Baseline characteristics will be summarised for the Safety Population. For categorical variables, absolute numbers (with percentages) will be provided. For continuous variables, means (with standard deviations) or medians (with range) will be presented. Data will be presented separately for the escalation and expansion phases (by dose received) and overall. Patients who died or withdrew before treatment started will be described separately.

9.2 Primary endpoints

9.2.1 MTD & R2PD

The primary endpoint analysis will make use of Bayesian methods for estimating the probability of a DLT at each of the doses under investigation. The MTD, which is the dose level with estimated DLT rate closest to the target toxicity level, will be reported with its associated DLT rate and 90% credible interval, using all DLT evaluable patients in Phase I. At the end of Phase I, the RP2D will

be determined based on a thorough review of all patient data (both safety, PD and anti- tumour activity) in consultation with the SRC, which will be the MTD or a lower tolerable dose.

The dose escalation process to determine the RP2D will be illustrated in a flow diagram.

9.2.2 Safety and tolerability

Safety data will be collected from the date of written consent. The safety population will be used in the reporting of adverse events (AEs). AEs will be reported for each dose level and presented as tables of frequency of AEs by body system and by worst severity grade observed during the trial. AEs related to either study drug in the combination (possibly, probably or highly probably related) will be tabulated separately. Non-clinically significant laboratory values that meet National Cancer Institute (NCI) Common Terminology Criteria for Adverse Events (CTCAE) version 5.0 will be summarised by descriptive statistics. Clinically significant laboratory values are routinely reconciled with the AEs, and will therefore be captured in AE tables. Data will be presented separately for escalation vs. expansion, by dose received for those in escalation, and by each of the five expansion cohorts.

9.2.3 Anti-tumour activity

The analyses in this subsection will be performed for both the safety population and the response evaluable population if not indicated otherwise. Patients in Part A who are dosed at or above biologically active dose and satisfy the eligibility criteria for any of the expansion cohorts can contribute to the efficacy evaluation in that expansion cohort.

- **Clinical benefit rate (CBR)**

CBR defined as the proportion of patients with CR or PR assessed by imRECIST, or SD of 6 months or longer. The posterior probability of clinical benefit rate greater than 5% will be calculated in addition to the posterior CBR with credible interval. The decision of whether the treatment is active will be made based on the 3-outcome decision criteria outlined in section 2.6.

- **Objective response rate (ORR)**

ORR is defined as the proportion of patients with Complete Response (CR) or confirmed Partial Response (PR), as assessed by investigator, throughout the duration of the trial.

- **Progression-free survival (PFS)**

PFS will be calculated as time from start of the combination until date of confirmed progressive disease (PD) (radiographic or clinical progression) or death due to any cause, whichever occurs first.

The KM estimates for the PFS probability at 6, 12, and 24 months will be reported alongside their 90% CI. Patients who are lost to follow-up will be censored at the last scheduled disease

assessment on study, i.e., their last known date to be alive and progression-free; patients who start a new treatment will be censored at the new treatment start date.

- **Duration of response**

It is defined as the time from the date of first confirmed observation of CR or PR to PD or death in patients who had CR, PR. The Kaplan-Meier method will be used to estimate survival probabilities and visualise duration of response. Median duration of response will be reported alongside its 90% CI. Patients not reaching disease progression at the time of analysis will be censored at the last date known to be progression-free.

- **Best change in tumour size**

Best change in tumour size is defined as the maximum reduction from baseline or the minimum increase from baseline in the absence of a reduction in the sum of the longest diameters (or the short axis measurements for lymph nodes) of the target lesions. The best absolute change in target lesion tumour size from baseline, and best percentage change in target lesion tumour size from baseline will be summarized using descriptive statistics. The best change in tumour size will include all assessments prior to progression.

9.3 Secondary endpoints

Secondary endpoints relate to pharmacokinetic/pharmacodynamics data and are not covered by this analysis plan.

The analyses for disease response by imRECIST criteria, clinical benefit rate, best changes in tumour size, progression-free survival and duration of response for Part A will utilise the same methodology as applied in Part B for the analysis of the primary endpoint.

9.4 Tertiary endpoints

Tertiary endpoints that relate to translational/transcriptomic data are not covered by this statistical analysis plan.

9.4.1 Health-related QoL (HRQOL)

HRQoL will be assessed using the global score of the EORTC QLQ-C30. QLQ-C30 questionnaires will be administered at three points during the trial: baseline, during cycle 4 and at the end of study. Repeated measured data will be analysed through descriptive statistics and mixed effects modelling (accounting for within subject correlation) using parametric or more flexible models to assess for changes over time.

9.4.2 Overall survival (OS)

OS will be measured from the date of administration of the first dose of ASTX660 to death. The KM method will be used to estimate survival probabilities and visualise OS. Median OS will be reported alongside its 90% CI. The KM estimate for the OS probability at 12 and 24 months will be reported alongside its 90% CI. Patients who are lost to follow-up will be censored at the time of last contact; patients who start a new treatment will not be censored.

10 Statistical software and analysis program locations

10.1 Statisticial software

Analysis will be conducted using R version 4.2 (or subsequent versions).
The TiTE-CRM model will be analysed using functions from the R packages dfcrm by K. Cheung [13] and dtpcrm by Yap et al. [13]. In particular, the dose for the next cohort of patients according to the TiTE-CRM model will be calculated using the applied_titecrm function.

10.2 Analysis program locations

All programs will be stored in the analyses folder for DDU-ASTEROID on the ICR-CTSU server. Only the DDU ASTEROID trial statistician(s), ICR-CTSU IT staff, Director and Deputy Directors of ICR-CTSU will be able to see the analysis folder. Programmes will be stored under the type of analysis e.g. Study report. All official analysis reports that are to be circulated externally of ICR-CTSU will be password protected. Hard copies of reports will be stored securely in the statistical section of the DDU-ASTEROID trial master file held in a locked fire proof cupboard with restricted access.

11 References

[1] M. Chesi, N. N. Mirza, V. M. Garbitt, M. E. Sharik, A. C. Dueck, Y. W. Asmann, I. Akhmetzyanova, H. E. Kosiorek, A. Calcinotto, D. L. Riggs and others, “IAP antagonists induce anti-tumor immunity in multiple myeloma,” *Nature medicine,* vol. 22, p. 1411–1420, 2016.

[2] K. Cheung, “dfcrm: Dose-Finding by the Continual Reassessment Method,” 2019.

[3] Y. K. Cheung and R. Chappell, “Sequential designs for phase I clinical trials with late-onset toxicities,” *Biometrics,* vol. 56, p. 1177–1182, 2000.

[4] M. Darding and P. Meier, “IAPs: guardians of RIPK1,” *Cell Death & Differentiation,* vol. 19, p. 58–66, 2012.

[5] N. Yatim, H. Jusforgues-Saklani, S. Orozco, O. Schulz, R. Barreira da Silva, C. Reis e Sousa, D. R. Green, A. Oberst and M. L. Albert, “RIPK1 and NF-κB signaling in dying cells determines cross-priming of CD8+ T cells,” *Science,* vol. 350, p. 328–334, 2015.

[6] N. Yatim, S. Cullen and M. L. Albert, “Dying cells actively regulate adaptive immune responses,” *Nature Reviews Immunology,* vol. 17, p. 262–275, 2017.

[7] C. Yap, A. Pettitt and L. Billingham, “Screened selection design for randomised phase II oncology trials: an example in chronic lymphocytic leukaemia,” *BMC Medical Research Methodology,* vol. 13, p. 1–9, 2013.

[8] C. Yap, D. Slade, K. Brock and Y. Pan, “dtpcrm: Dose Transition Pathways for Continual Reassessment Method,” 2019.

[9] T. Tenev, K. Bianchi, M. Darding, M. Broemer, C. Langlais, F. Wallberg, A. Zachariou, J. Lopez, M. MacFarlane, K. Cain and others, “The Ripoptosome, a signaling platform that assembles in response to genotoxic stress and loss of IAPs,” *Molecular cell,* vol. 43, p. 432–448, 2011.

[10] N. Müller-Sienerth, L. Dietz, P. Holtz, M. Kapp, G. U. Grigoleit, C. Schmuck, H. Wajant and D. Siegmund, “SMAC mimetic BV6 induces cell death in monocytes and maturation of monocyte-derived dendritic cells,” *PLoS One,* vol. 6, p. e21556, 2011.

[11] J. Lopez and P. Meier, “To fight or die—inhibitor of apoptosis proteins at the crossroad of innate immunity and death,” *Current opinion in cell biology,* vol. 22, p. 872–881, 2010.

[12] S. Hong and Y. Wang, “A three-outcome design for randomized comparative phase II clinical trials,” *Statistics in medicine,* vol. 26, p. 3525–3534, 2007.

[13] M. Feoktistova, P. Geserick, B. Kellert, D. P. Dimitrova, C. Langlais, M. Hupe, K. Cain, M. MacFarlane, G. Häcker and M. Leverkus, “cIAPs block Ripoptosome formation, a RIP1/caspase-8 containing intracellular cell death complex differentially regulated by cFLIP isoforms,” *Molecular cell,* vol. 43, p. 449–463, 2011.

[14] S. K. Dougan and M. Dougan, “Regulation of innate and adaptive antitumor immunity by IAP antagonists,” *Immunotherapy,* vol. 10, p. 787–796, 2018.

Supplementary Note 3

CONSORT-DEFINE downloadable checklist

Recommended checklist items to consider in an early phase dose-finding clinical trial report from CONSORT 2010 and CONSORT-DEFINE checklists^

Yap C, Solovyeva O, de Bono J, et al. Enhancing reporting quality and impact of early phase dose-finding clinical trials: CONSORT Dose-finding Extension (CONSORT-DEFINE) guidance. *BMJ* 2023;383:e076387. doi:10.1136/bmj-2023-076387

Category and section	Standard CONSORT 2010 checklist item		CONSORT-DEFINE checklist item for EPDF Trials		Addressed on Page No¤
	Item No	CONSORT 2010	Item No	CONSORT-DEFINE	
**Title and abstract**					
	1a	Identification as a randomised trial in the title	1a†	Identification as an early phase dose-finding (eg, first-in-human, dose escalation or de-escalation, phase 1, phase 1/2, expansion, dose titration) and, if applicable, randomised trial in the title or abstract	Manuscript: Title and abstract
	1b	Structured summary of trial design, methods, results, and conclusions (for specific guidance, see CONSORT for abstracts)	1b	Structured summary of trial design, methods, results, and conclusions (for specific guidance, see CONSORT-DEFINE for abstracts)	Manuscript: Abstract covers important aspects but is limited by word limit
**Introduction**					
Background and objectives	2a	Scientific background and explanation of rationale	2a.1†	Description of research question(s) and justification for undertaking the trial, including summary of relevant clinical studies (published and unpublished) examining benefits and harms for each intervention	Manuscript: Introduction
			2a.2*	Summary of key findings from relevant non-clinical or preclinical research	Manuscript: Introduction
			2a.3*	Summary of findings from previously generated preclinical and translational studies to support any planned biomarker substudies (where applicable)	Manuscript: Introduction

Category and section	Standard CONSORT 2010 checklist item		CONSORT-DEFINE checklist item for EPDF Trials		Addressed on Page No¤
	Item No	CONSORT 2010	Item No	CONSORT-DEFINE	
	2b	Specific objectives or hypotheses	2b†	Specific objectives (eg, relating to safety, activity, pharmacokinetics, pharmacodynamics, recommended dose(s))	Manuscript: Introduction and Methods
**Methods**					
Trial design	3a	Description of trial design (such as parallel, factorial) including allocation ratio	3a.1†	Description of trial design elements, such as dose escalation or de-escalation strategy, number of treatment groups, allocation ratio if relevant, and details of any prespecified trial adaptations	Manuscript: Methods
			3a.2*	Trial design schema to show the flow of major transition points (eg, dose escalation to dose expansion, phase 1 to phase 2, single ascending dose to multiple ascending dose)	Manuscript: Methods; Main Fig 1a-b
			3a.3*	Statistical methods or rationale underpinning the trial design	Manuscript: Methods section
			3a.4*	Starting dose(s) with rationale	Manuscript: Methods section; Protocol v4.0: page 36
			3a.5*	Range of planned dose levels with rationale	Manuscript: Methods section; Protocol v4.0: page 36
			3a.6*	Presentation of planned dose levels (eg, as a diagram, table, or infographic), where applicable	Manuscript: Fig. 1a-b; Protocol v4.0: Table 1.
			3a.7*	Skipping of dose level(s), if applicable	N/A

Category and section	Standard CONSORT 2010 checklist item		CONSORT-DEFINE checklist item for EPDF Trials		Addressed on Page No¤
	Item No	CONSORT 2010	Item No	CONSORT-DEFINE	
			3a.8*	Planned cohort size(s) (eg, fixed, flexible, adaptive)	Manuscript: Methods section
			3a.9*	Dose allocation method within a dose level (including sequence and interval between dosing of participants, eg, sentinel or staggered dosing)	Manuscript: Methods section
			3a.10*	Dose expansion cohort(s), if applicable, with rationale	Protocol: Section 5.5
			3a.11*	Criteria for progression to the next part of the trial (eg, phase 1 to phase 2, single ascending dose to multiple ascending dose), where applicable	Manuscript: Methods section; Protocol v4.0: Sections 5.3, 5.4, 5.5
	3b	Important changes to methods after trial commencement (such as eligibility criteria), with reasons	3b†	Important changes to the design or methods after trial commencement (eg, insertion of unplanned additional doses) outside the scope of the prespecified adaptive design features, with reasons	Manuscript: Results section explaining protocol amendment for management of asymptomatic lipase elevations; Protocol v4.0: Section 5.7.2
Participants	4a	Eligibility criteria for participants	4a		Manuscript: Methods and Supplementary Methods sections; Protocol v4.0 Section 4.1
	4b	Settings and locations where the data were collected	4b		Manuscript: Methods section

Category and section	Standard CONSORT 2010 checklist item		CONSORT-DEFINE checklist item for EPDF Trials		Addressed on Page No¤
	Item No	CONSORT 2010	Item No	CONSORT-DEFINE	
Interventions	5	The interventions for each group with sufficient details to allow replication, including how and when they were actually administered	5a†	Interventions for each dose level (within each group) with sufficient details to allow replication, including administration route and schedule showing how and when they were actually administered	Manuscript: Methods section
			5b*	Criteria for dose discontinuation, dose modifications, and dosing delays of allocated interventions for a given trial participant (eg, dose change in response to harms, participant request, or improving or worsening disease)	Manuscript: Methods section; Protocol v4.0: Section 5.6
Outcomes	6a	Completely defined prespecified primary and secondary outcome measures, including how and when they were assessed	6a†	Primary and secondary outcomes, including the specific measurement variable, analysis metric, method of aggregation, and time point for each outcome. Explanation of the clinical relevance of chosen outcomes is strongly recommended. Any other outcomes used to inform prespecified adaptations should be described with the rationale	Manuscript: Methods section Protocol v4.0: Section 3.1
	6b	Any changes to trial outcomes after the trial commenced, with reasons	6b†	Any unplanned changes to trial outcomes after the trial commenced, with reasons	N/A

Category and section	Standard CONSORT 2010 checklist item		CONSORT-DEFINE checklist item for EPDF Trials		Addressed on Page No[¤]
	Item No	CONSORT 2010	Item No	CONSORT-DEFINE	
Sample size	7a	How sample size was determined	7a†	Estimated number of participants (minimum, maximum, or expected range) needed to address trial objectives and how it was determined, including clinical and statistical assumptions supporting any sample size and operating characteristics	Manuscript: Methods section Protocol v4.0: Section 3.2
	7b	When applicable, explanation of any interim analyses and stopping guidelines	7b†	Prespecified interim decision making criteria or rules that guided the trial adaptation process (eg, dosing decision to (de-)escalate); prespecified and actual timing and frequency of interim data reviews and the information to inform trial adaptations	Manuscript: Methods section; Protocol v4.0: Section 5.3-5.4
Randomization (if applicable)					
Sequence generation	8a	Method used to generate the random allocation sequence	8a		Not planned
	8b	Type of randomisation; details of any restriction (such as blocking and block size)	8b†	Type of randomisation; details of any restrictions (such as blocking and block size); any prespecified adaptive assignment rules or algorithm leading to adjustments in the allocation ratio, including timing and frequency of updates; any changes to the allocation rule following trial adaptation decisions	Not planned

Category and section	Standard CONSORT 2010 checklist item		CONSORT-DEFINE checklist item for EPDF Trials		Addressed on Page No¤
	Item No	CONSORT 2010	Item No	CONSORT-DEFINE	
Allocation concealment mechanism	9	Mechanism used to implement the random allocation sequence (such as sequentially numbered containers), describing any steps taken to conceal the sequence until interventions were assigned	9		Not planned
Implementation	10	Who generated the random allocation sequence, who enrolled participants, and who assigned participants to interventions	10		Not planned
Blinding	11a	If done, who was blinded after assignment to interventions (eg, participants, care providers, and how	11a		Not planned
	11b	If relevant, description of the similarity of interventions	11b		Not planned
Statistical methods	12a	Statistical methods used to compare groups for primary and secondary outcomes	12a.1†	Statistical methods for primary and secondary outcomes and any other outcomes used to make prespecified adaptations	Manuscript: Methods section; Statistical Analysis Plan v1.0
			12a.2*	For the implemented adaptive design features, statistical methods used for estimation (eg, safety, dose(s), treatment effects) and to make inferences	Manuscript: Methods section; Statistical Analysis Plan v1.0
	12b	Methods for additional analyses, such as subgroup analyses and adjusted analyses	12b†	Statistical methods for additional analyses (eg, subgroup and adjusted analyses, pharmacokinetics or pharmacodynamics, biomarker correlative analyses)	Manuscript: Methods section; Statistical Analysis Plan v1.0

Category and section	Standard CONSORT 2010 checklist item		CONSORT-DEFINE checklist item for EPDF Trials		Addressed on Page No¤
	Item No	CONSORT 2010	Item No	CONSORT-DEFINE	
			12c*	Analysis population(s) (eg, evaluable population for dose-finding, safety population)	Manuscript: Methods section; Protocol v4.0: Section 3.5
			12d*	Strategies for handling intercurrent events occurring after treatment initiation (eg, how dosing adjustments were handled) that can affect either the interpretation or the existence of the measurements associated with the clinical question of interest, and any methods to handle missing data	Manuscript: Methods section; Statistical Analysis Plan v1.0
**Results**					
Participant flow (a diagram is strongly recommended)	13a	For each group, the numbers of participants who were randomly assigned, received intended treatment, and were analysed for the primary outcome	13a†	For each group, the number of participants who were assigned to each dose level at each interim analysis (eg, for dosing decisions), received intended treatment, and were analysed for the primary outcome and, if applicable, any other outcomes used to inform prespecified adaptations	Fig 1b
	13b	For each group, losses and exclusions after randomisation, together with reasons	13b†	For each group, losses and exclusions after allocation to each dose level, together with reasons	Fig 1b
Recruitment	14a§	Dates defining the periods of recruitment and follow-up	14a§		Manuscript: Results Section
	14b§	Why the trial ended or was stopped	14b§		Manuscript: Results Section

Category and section	Standard CONSORT 2010 checklist item		CONSORT-DEFINE checklist item for EPDF Trials		Addressed on Page No¤
	Item No	CONSORT 2010	Item No	CONSORT-DEFINE	
			14c*	Trial adaptation decisions made (including on what basis they were made, and when) in light of the prespecified decision making criteria and observed accrued data	Manuscript: Results Section
Baseline data	15	A table showing baseline demographic and clinical characteristics for each group	15†	Baseline demographic and clinical characteristics across each dose level within each group, where appropriate	Manuscript: Results Section; Tables 1-2
Numbers analysed	16	For each group, number of participants (denominator) included in each analysis and whether the analysis was by original assigned groups	16†	For each group, the number of participants (denominator) included in each analysis across each dose level, and whether the analysis was by original assigned interventions	Manuscript: Results Section; Fig 1b
Outcomes and estimation	17a	For each primary and secondary outcome, results for each group, and the estimated effect size and its precision (such as 95% confidence interval)	17a†	For each primary and secondary outcome, results for each dose level within each group, and the estimated effect size and its precision, if applicable	Manuscript: Results Section
	17b§	For binary outcomes, presentation of both absolute and relative effect sizes is recommended	17b§		N/A
			17c*	Report interim results used to inform interim decision making such as dose escalation, de-escalation, or staying at the same dose	Manuscript: Results Section; Fig. 1b
Ancillary analyses	18	Results of any other analyses performed, including subgroup analyses and adjusted analyses, distinguishing prespecified from exploratory	18		Manuscript: Results Section

Category and section	Standard CONSORT 2010 checklist item		CONSORT-DEFINE checklist item for EPDF Trials		Addressed on Page No¤
	Item No	CONSORT 2010	Item No	CONSORT-DEFINE	
Harms	19	All important harms or unintended effects in each group (for specific guidance, see CONSORT for harms (1))	19†	All important harms (eg, adverse events or effects, toxicities) reported by dose level in each group (for specific guidance, see CONSORT for harms (2))	Manuscript: Results Section
**Discussion**					
Limitations	20	Trial limitations, addressing sources of potential bias, imprecision, and, if relevant, multiplicity of analyses	20		Manuscript: Discussion section
Generalizability	21	Generalisability (external validity, applicability) of the trial findings	21		Manuscript: Discussion section
Interpretation	22	Interpretation consistent with results, balancing benefits and harms, and considering other relevant evidence	22		Manuscript: Discussion section
**Other information**					
Registration	23	Registration number and name of trial registry	23		Manuscript: Abstract and Methods sections; Protocol v4.0
Protocol	24	Where the full trial protocol can be accessed, if available	24		Manuscript: Methods section
Funding	25	Sources of funding and other support (such as supply of drugs), role of funders	25		Manuscript: Acknowledgements section

Category and section	Standard CONSORT 2010 checklist item		CONSORT-DEFINE checklist item for EPDF Trials		Addressed on Page No¤
	Item No	CONSORT 2010	Item No	CONSORT-DEFINE	
Data monitoring			26a*	Composition of any decision making or safety review committee or group; summary of its role and reporting structure; statement of whether it is independent from the sponsor and competing interests; and reference to where further details can be found (such as in a charter or protocol)	Protocol: Section 5.3.
			26b*	Description of who had access to interim results and made the interim and final decision to terminate the trial (or part(s) of the trial, eg, end of dose escalation), and measures to safeguard the confidentiality of interim information	Protocol: Sections 5.3 and 14
Dissemination			27*	Specify, if applicable, whether and when results (such as safety and/or activity) were reported externally (eg, through scientific presentations, journal publication, or the trial website) while the trial (or part(s) of the trial) was still ongoing	Interim results were previously reported as an oral presentation at the American Association of Cancer Research 2024 Annual Meeting with an abstract published in the conference proceedings. https://doi.org/10.1158/1538-7445.AM2024-CT035

CONSORT=CONonsolidated Standards Of Reporting Trials; DEFINE=Dose-finding Extension; EPDF=early phase dose-finding.

^ This checklist should be used in conjunction with the CONSORT explanation and elaboration document (3) for important clarifications on the checklist items. Empty items in the CONSORT-DEFINE column indicate no modification from the standard CONSORT item. CONSORT extensions for non-pharmacological treatments and outcomes might

also be relevant (4). Note that the term “dose” in the checklist can be considered synonymous and used interchangeably with dosage, or dosing regimen (dose or schedule), or a unit dose.
¤ If the information is not provided in the primary paper, give details of where this information is available. This may include locations such as a published protocol or other published papers (provide citation details) or a website (provide the URL).
* New items that should only be applied in reference to CONSORT-DEFINE.
† Modified items that require reference to both CONSORT and CONSORT-DEFINE.
§ Item wording remains unchanged in reference to CONSORT, but additional CONSORT-DEFINE explanatory text has been provided to clarify additional considerations for early phase dose-finding trials (web appendix 3).
