# Supplementary material for: IAP antagonist tolinapant (ASTX660) in combination with pembrolizumab in advanced solid tumors: a phase I trial
Source: Nat Commun. 2026 Sep 30;17:11255. doi: 10.1038/s41467-026-77552-7 (PMC13720124; doi:10.1038/s41467-026-77552-7)
Supplement: Supplementary file 2 [file 41467_2026_77552_MOESM2_ESM.pdf]

# Description of Additional Supplementary Files

**Supplementary Data 1.** Summary of paired biopsies for pharmacodynamic analyses.

**Supplementary Data 2.** Differential Expression and GSEA Results (Bulk RNAseq)

a. Differential gene expression (Z-scores) of patient samples at baseline (C1D1)
b. Differential gene expression (Z-scores) of patient samples following ASTX660 (C1D7)
c. GSEA (MSigDB Hallmark): Responders vs Non-responders, Pre-treatment (C1D1)
d. GSEA (MSigDB Hallmark): Responders vs Non-responders, Post-treatment (C1D7)
e. GSEA (MSigDB Hallmark): C1D7 vs C1D1, Responders
f. GSEA (MSigDB Hallmark): C1D7 vs C1D1, Non-responders

**Supplementary Data 3.** List of 15 plasma proteins significantly upregulated in non-responders compared to responders in ASTEROID (ASTX660+Pembrolizumab) showing overlap with ASTX660-01 monotherapy trial
