# Supplementary material for: IAP antagonist tolinapant (ASTX660) in combination with pembrolizumab in advanced solid tumors: a phase I trial
Source: Nat Commun. 2026 Sep 30;17:11255. doi: 10.1038/s41467-026-77552-7 (PMC13720124; doi:10.1038/s41467-026-77552-7)
Supplement: Supplementary file 3 [file 41467_2026_77552_MOESM6_ESM.pdf]

nature portfolio

Corresponding author(s): Pascal Meier

Last updated by author(s): Mar 6, 2026

Reporting Summary

Nature Portfolio wishes to improve the reproducibility of the work that we publish. This form provides structure for consistency and transparency in reporting. For further information on Nature Portfolio policies, see our Editorial Policies and the Editorial Policy Checklist.

Statistics

For all statistical analyses, confirm that the following items are present in the figure legend, table legend, main text, or Methods section.

n/a	Confirmed	
☐	☒	The exact sample size (*n*) for each experimental group/condition, given as a discrete number and unit of measurement
☐	☒	A statement on whether measurements were taken from distinct samples or whether the same sample was measured repeatedly
☐	☒	The statistical test(s) used AND whether they are one- or two-sided<br>*Only common tests should be described solely by name; describe more complex techniques in the Methods section.*
☐	☒	A description of all covariates tested
☐	☒	A description of any assumptions or corrections, such as tests of normality and adjustment for multiple comparisons
☐	☒	A full description of the statistical parameters including central tendency (e.g. means) or other basic estimates (e.g. regression coefficient) AND variation (e.g. standard deviation) or associated estimates of uncertainty (e.g. confidence intervals)
☐	☒	For null hypothesis testing, the test statistic (e.g. *F*, *t*, *r*) with confidence intervals, effect sizes, degrees of freedom and *P* value noted<br>*Give P values as exact values whenever suitable.*
☐	☒	For Bayesian analysis, information on the choice of priors and Markov chain Monte Carlo settings
☐	☒	For hierarchical and complex designs, identification of the appropriate level for tests and full reporting of outcomes
☐	☒	Estimates of effect sizes (e.g. Cohen's *d*, Pearson's *r*), indicating how they were calculated

Our web collection on statistics for biologists contains articles on many of the points above.

Software and code

Policy information about availability of computer code

Data collection

Clinical data: Data was entered into MACRO EDC system (Version 4.11.0.459) then extracted using the in-built MACRO export function into a Microsoft Access file.

Cell death marker IHC: Slides were scanned at 20x using a Nano Zoomer S60 Hamamatsu Scanner.

PD-L1 IHC: Slides were scanned at high resolution (200×) using the VS200 digital slide scanner (Olympus, Tokyo, Japan).

Multiplex immunofluorescence: Slides were scanned by the Perkin Elmer Vectra Polaris scanner (Akoya Bioscience) using PhenoImager HT 2.0 software.

Proximity ligation assay: All images were acquired using an Olympus VS200 scanner controlled by the Olympus SlideView software. The camera used was a Hamamatsu Orca FUSION Camera and samples were illuminated using a XCITE Novem light source. The objective used was a UPLXAPO 20x / 0,80. Each sample was imaged in four cycles, each time with DAPI, FITC, CY3 and CY5 channels. The first three cycles correspond to the three Detection mixes; the fourth cycle images autofluorescence. Following image acquisition, the four separate layers were merged into a single layered image using the ‘Combine Cannels of Multi-Layer Image’ function in Olympus VS200 Desktop software. Images were visualized on QuPath with the following adjusted channel (min, max) settings: DAPI (0, 29190), RIPK1:cC8 (0.163, 3716), RIPK1:RIPK3 (11.1, 2600); gamma = 1 for all channels.

Data analysis

Dose-finding decisions were based on the time-to-event continual reassessment method (TITE-CRM), implemented using the dfcrm (version 0.2) and dtpcrm (version 0.1) packages in R (version 4.4.1, R Foundation for Statistical Computing). Survival analyses were performed using

the survival package (version 3.8) with Kaplan-Meier curves generated via ggsurv. Descriptive summaries were produced using gtsummary (version 2.2.0) and additional figures were created using ggplot2 (version 3.5.2). No novel computational code or algorithms were developed for this study. Analysis scripts are available from the corresponding author upon request. Details of the parameters for the TITE-CRM model are included in the study protocol.

Pharmacokinetics parameters were estimated with standard noncompartmental methods using Phoenix WinNonlin 6.4 (Pharsight Corporation). Statistical analyses were performed using R version 3.5.1.

Immunohistochemistry analysis of cell death markers was performed using QuPath (version 0.5.0).
Multiplex immunofluorescence digital image analysis was performed in HALO Image Analysis Platform version 3.6.4134 (Indica Labs) using the Highplex FL v4.2.14 module for cell segmentation, mark up, phenotyping and quantification.

Plasma Protein statistical analysis was performed using R version 4.3.3. Type III Sum of Squares ANOVA analysis was performed using R package afex (version 1.3_1), lme4 (version 1.1_35), lmerTest (version 3.1_3). The pre-processing was performed in python (3.12.8) using pandas (2.2.3). Volcano plots were produced using R package ggplot2 (3.5.1).

RNAseq analysis was performed using R version 4.4.2.

TCRseq analysis was performed using the Immunarch R package (Nazarov V, T. V., Fiadziushchanka S, Rumynskiy E, Popov A, Balashov I, Samokhina M. immunarch: Bioinformatics Analysis of T-Cell and B-Cell Immune Repertoires. (2023).)

All workflows for Whole Exome Sequencing analysis were executed on the ALMA HPC cluster under SLURM scheduling using containerized tools via Singularity. Key tools included nf-core/Sarek, nf-core/hlatyping, GATK, Strelka2, Manta, CNVkit, VEP (v110/v114), BCFtools (v1.16), pVACtools, SOPRANO, and Java JDK 15. Data processing and figure generation were performed in R (v4.3.1). All scripts, parameter files, and configuration settings are available upon reasonable request.

Statistical analyses of cIAP1 levels, multiplex immunofluorescence, and flow cytometry were performed using GraphPad Prism 10.

For manuscripts utilizing custom algorithms or software that are central to the research but not yet described in published literature, software must be made available to editors and reviewers. We strongly encourage code deposition in a community repository (e.g. GitHub). See the Nature Portfolio guidelines for submitting code & software for further information.

Data

Policy information about availability of data

All manuscripts must include a data availability statement. This statement should provide the following information, where applicable:

- Accession codes, unique identifiers, or web links for publicly available datasets
- A description of any restrictions on data availability
- For clinical datasets or third party data, please ensure that the statement adheres to our policy

The full study protocol and statistical analysis plan are provided in the Supplementary Material. De-identified sequencing data has been deposited at the European Genome-phenome Archive (EGA), which is hosted by the EBI and the CRG, under accession number EGAC50000001005. Further information about EGA can be found at https://ega-archive.org and "The European Genome-phenome Archive of human data consented for biomedical research". Requests for the clinical and translational data from patient samples must provide clinically relevant rationale in adherence with the intent of the study and patients' consent. These will be reviewed by the corresponding authors and the ICR to determine whether the request is subject to any intellectual property, ethical and/or confidentiality considerations. A prompt response will be provided to such requests. Patient identifiers or information that may reveal the patient's identity will not be shared. Any data or material that can be shared will be made available through a material transfer agreement with the ICR.

Research involving human participants, their data, or biological material

Policy information about studies with human participants or human data. See also policy information about sex, gender (identity/presentation), and sexual orientation and race, ethnicity and racism.

Reporting on sex and gender	Biological sex is reported in our study. Gender-based analyses was not performed. Written informed consent was obtained from patients for sharing of individual-level data.
Reporting on race, ethnicity, or other socially relevant groupings	Self-reported ethnicity is reported in our study.
Population characteristics	Full patient characteristics and dosing are shown in Table 2. Median age was 55 years [interquartile range (IQR) 45-73]. 78.3% of patients were female, consistent with the tumor types of enrolled patients which were predominantly breast and cervical cancers. 15 (65.2%) self-reported White British ethnicity. The median number of prior lines of systemic treatment for metastatic disease was 5 (IQR 2-6). Four (17.4%) had prior immune checkpoint inhibitor treatment (Table 1). Patients were enrolled with the following tumor types: breast [ER+HER2- (n=12), triple-negative breast cancer (TNBC, 1), ER-HER2+ (1)], cervical (3), cholangiocarcinoma (1), head and neck squamous cell carcinoma (HNSCC, 2), hepatocellular carcinoma (HCC, 1), melanoma (1), and pancreatic (1).
Recruitment	Referrals to our recruiting centres were from across the UK encompassing a range of socioeconomic areas. While not fully representative of global cancer demographics, the enrolled cohort reflects populations most frequently referred to tertiary cancer centers where early phase trials are conducted.
Ethics oversight	The protocol, patient information sheet, consent form and all relevant study documentation were approved by the Institute

Ethics oversight	of Cancer Research (ICR)/Royal Marsden Hospital (RMH) joint Committee for Clinical Research, the local institutional Research Ethics Committee, the Health Research Authority and Medicines and Healthcare products Regulatory Agency. The study was carried out in compliance with The International Conference on Harmonization Good Clinical Practice Guidelines and requirements of the UK Clinical Trials regulations (SI 2004/1031 and SI 2006/1928 as amended). Written informed consent was obtained from all participants. No participant compensation was provided. The study was sponsored and monitored by the ICR.

Note that full information on the approval of the study protocol must also be provided in the manuscript.

Field-specific reporting

Please select the one below that is the best fit for your research. If you are not sure, read the appropriate sections before making your selection.

☒ Life sciences ☐ Behavioural & social sciences ☐ Ecological, evolutionary & environmental sciences

For a reference copy of the document with all sections, see nature.com/documents/nr-reporting-summary-flat.pdf

Life sciences study design

All studies must disclose on these points even when the disclosure is negative.

Sample size	A dose-escalation design based on an efficient, modified Bayesian TITE-CRM was used to identify the MTD. The full DLT assessment period OF 6 weeks. The TITE-CRM will target the dose (from a selection of four possible doses) with estimated DLT rate closest to the target rate of 25%. The skeletons or prior DLT rates at each dose levels have been elicited using a combination of model-calibrated priors (23) and through discussion with clinicians (using an acceptable target DLT rates in the range between 19% and 31%). We have used the getprior function (in R package dfcrm) where the prior MTD guess is at Dose level 3. Starting dose is dose level 1, d(1).<br>An empiric dose-toxicity model will be used, where the slope parameter is assumed to follow a normal distribution with prior mean 0 and a pre-specified prior variance of 0.85.<br>The TITE-CRM design will incorporate practical considerations, including no skipping of untried doses in escalation, no escalation if the overall rate of toxicity seen at the current dose level is above the target rate and for stopping early if there is a high probability that the lowest dose is too toxic. Specifically the model will suggest stopping early due to excess toxicity if $P(\theta_L > 0.25 \mid data) > 0.88$, where theta is the probability of DLT at the lowest dose level. Assuming patients have completed their DLT follow-up period, this translates to stopping early when we see 2/2 DLT or 3/4 at the lowest dose (Yap et al 2017).<br>A target sample size of 18 evaluable patients was recruited to Part A to enable adequate collection of paired evaluable tumour and blood samples for pharmacodynamic analyses, and this target sample size was anticipated to be sufficient to determine the MTD as per the TITE-CRM design.
Data exclusions	No patients were excluded from safety evaluation (total n=23); 2 patients were excluded from MTD/RP2D evaluation due to incorrect dosing and withdrawal of consent. 3 patients were excluded from response evaluation due to rapid disease progression (within 6 weeks) since there was not enough time for combination drug exposure to confidently assess treatment response. (refer to Fig 1b Consort diagram).
Replication	Not relevant for phase 1 trial
Randomization	Not relevant for phase 1 trial
Blinding	Not relevant for phase 1 trial

Reporting for specific materials, systems and methods

We require information from authors about some types of materials, experimental systems and methods used in many studies. Here, indicate whether each material, system or method listed is relevant to your study. If you are not sure if a list item applies to your research, read the appropriate section before selecting a response.

Materials & experimental systems

n/a	Involved in the study	
☐	☒	Antibodies
☐	☒	Eukaryotic cell lines
☒	☐	Palaeontology and archaeology
☒	☐	Animals and other organisms
☐	☒	Clinical data
☒	☐	Dual use research of concern
☒	☐	Plants

Methods

n/a	Involved in the study	
☒	☐	ChIP-seq
☐	☒	Flow cytometry
☒	☐	MRI-based neuroimaging

Antibodies

Antibodies used	IHC:<br>RIPK1 (E8S7U) CST 73271

RIPK3 (E7A7F) CST 10188
MLKL (E7V4W) CST 26539
Cleaved caspase 3 (Asp175 - 5A1E) CST 9664
Cleaved caspase 8 (D374) (18C8) CST 9496
Ki67 (SP6) AB16667
cIAP1 (EPR4673) AB108361
PD-L1 (E1L3N) CST 13684

mIF Panel 1:
CD4 (EPR6855) AB133616
CD8 (C8/144B) M7103
FoxP3 (236A/E7) AB20034
Granzyme B (EPR8260) AB134933
PD1 (EPR4877(2)) AB137132
PanCK (AE1/AE3) M3515

mIF Panel 2:
CD11c (EP1347Y) AB52632
CD141 (EPR4051) AB109189
CD123 (BR4MS) CST 23797S
CD28 (D274E) CST 38774
CD86 (E2G8P) CST 91882
PanCK (AE1/AE3) Dako M3515

mIF Panel 3:
CD68 (PG-M1) Dako M0814
CD206 (E2L9N) CST 91992
CCR2 (SN707) ThermoFisher MA5-41175
CD31 (JC70A) Dako M0823
CD20 (L26) AB9475
PanCK (AE1/AE3) Dako M3515

In situ Proximity Ligation Assay:
Naveni Plex Omni conjugates targeting CD3ε (CD3ε.F1.10152), Zap70 (ZAP70.F2.10292), PD1 (PD1.D1.10157), PD-L2 (PD-L2.D2.10158), Lck (LCK.B1.10271), Zap70 (ZAP70.B2.10306), cleaved caspase-8 (cleaved Caspase-8.C1:10372), RIPK1 (RIP.C2:10373), PD1 (PD1.A1.10034), SHP2 (SHP2.A2.10304), P-Tyrosine (P-Tyrosine.G1.10291), STAT3 (STAT3.G2.10355), PD1 (PD1.H1.10030), PD-L1 (PD-L1.H2.10033), RIPK3 (RIPK3.I1:10376), RIPK1 (RIP.I2:10377)

Validation

Primary antibodies were validated using the following positive and negative controls:
RIPK1 (E8S7U)- Pos control: Normal human colon; Human colorectal cancer; Neg control: HT29 cells treated with RIPK1 PROTAC degrader
RIPK3 (E7A7F)- Pos control: HT29 cells; Human normal colon; Neg control: HT29 RIPK3 knockout cells
MLKL (E7V4W) -Pos control: HT29 cells; Human colorectal cancer; Neg control: NCI-H522 cells
Cleaved caspase 3 (Asp175 - 5A1E)-Pos control: Human tonsil; Human colorectal cancer; Neg control: Human tonsil (no primary antibody control)
Cleaved caspase 8 (D374) (18C8)- Pos control: Human tonsil; Human colorectal cancer; Neg control: Human tonsil (no primary antibody control)
Ki67 (SP6)-Pos control: Human tonsil; Human lymph node; Neg control: Human tonsil (no primary antibody control)
mIF Panels 1 and 3- Pos control: Human tonsil
mIF Panel2- Pos control: Human tonsil and appendix
cIAP1 (EPR4673) -Pos control:Human appendix; MDA-MB-231 cells; Neg control: MDA-MB-231 cells treated with SM164
PD-L1 (E1L3N) - Pos control: Human Placenta; LNCaP cells; Neg control: Human Ovary; LNCaP cells treated with siRNA against PD-L1

Proximity Ligation Assay antibody conjugates: For evaluation of cell death protein interactions, fixed cell blocks of HT29 cells treated for 4 hours with DMSO or T/A/E [combination human TNF (100 ng/mL), ASTX660 (1 μM) and Emricasan (5 μM)] were used as negative and positive controls, respectively.

Eukaryotic cell lines

Policy information about cell lines and Sex and Gender in Research

Cell line source(s)

HT29 - human colon adenocarcinoma cell line isolated from a primary tumor of a 44 years old Caucasian female

Authentication

cell line purchased from ATCC

Mycoplasma contamination

Tested negative for Mycoplasma contamination

Commonly misidentified lines
(See ICLAC register)

Name any commonly misidentified cell lines used in the study and provide a rationale for their use.

Clinical data

Policy information about clinical studies
All manuscripts should comply with the ICMJE guidelines for publication of clinical research and a completed CONSORT checklist must be included with all submissions.

Clinical trial registration	NCT05082259
Study protocol	Full study protocol is submitted with the manuscript
Data collection	Data shown are from Part A (dose escalation), which enrolled 23 patients from March 2022 to November 2023, as of cut-off date October 24, 2025. The trial was conducted across the Drug Development Unit at the Royal Marsden and Cambridge University Hospitals NHS Foundations Trusts, UK.
Outcomes	The primary objectives of Part A were to propose a recommended dose for Phase II (RP2D) evaluation by establishing the maximum tolerated dose (MTD) of ASTX660 in combination with pembrolizumab and to assess the safety and tolerability of the combination. The secondary objectives were to evaluate the preliminary anti-tumor activity of the combination and characterize the pharmacokinetics (PK) of ASTX660. Tertiary objectives were to characterize the pharmacodynamics (PD) profile of single agent ASTX660 and the combination with pembrolizumab on the host immune response using blood and tumor samples; to assess putative predictive biomarkers of response and resistance to the doublet combination; and to evaluate the effect of ASTX660 on the tumor microenvironment including evaluation of inflammatory cytokines downstream of NF-kB.

Plants

Seed stocks	no plants involved
Novel plant genotypes	no plants involved
Authentication	no plants involved

Flow Cytometry

Plots

Confirm that:

☒ The axis labels state the marker and fluorochrome used (e.g. CD4-FITC).

☒ The axis scales are clearly visible. Include numbers along axes only for bottom left plot of group (a 'group' is an analysis of identical markers).

☒ All plots are contour plots with outliers or pseudocolor plots.

☒ A numerical value for number of cells or percentage (with statistics) is provided.

Methodology

Sample preparation	Whole blood was collected by venepuncture into EDTA blood collection tubes, pre-dose at the following timepoints: C1D1, C1D7, CXD1 (day 1 of each subsequent cycle) and at end of trial. Samples underwent flow cytometry to identify DC and T cell subpopulations using the following commercially available kits:<br>1. T cell panel of 10 markers (DURAclone IM, Beckman Coulter): CD45RA, CD197 (CCR7), CD28, CD279 (PD1), CD27, CD4, CD8, CD3, CD57, CD45<br>2. DC panel of 12 markers (DURAclone IM, Beckman Coulter):<br>o DC population markers: CD1c, CD11c, CD16, CD45, CD123, C-type lectin domain containing 9A (CLEC9A), human leukocyte antigen-DR isotype (HLA-DR)<br>o Lineage exclusion markers: CD3, CD14, CD19, CD20, CD56<br>Samples were incubated in the IM T Cell and DC Subsets tubes provided with the kits to stain the different cell populations. Erythrocytes were removed by lysis and leukocytes are acquired on the Beckman Coulter DX Flex according to standard procedures.
Instrument	Beckman Coulter DX Flex
Software	Beckman Coulter Kaluza C software
Cell population abundance	See Figshare Dataset. https://doi.org/10.6084/m9.figshare.31266469

Gating strategy | See Supplementary Methods for figure of gating strategies.

☒ Tick this box to confirm that a figure exemplifying the gating strategy is provided in the Supplementary Information.
