# Supplementary material for: IAP antagonist tolinapant (ASTX660) in combination with pembrolizumab in advanced solid tumors: a phase I trial
Source: Nat Commun. 2026 Sep 30;17:11255. doi: 10.1038/s41467-026-77552-7 (PMC13720124; doi:10.1038/s41467-026-77552-7)
Supplement: Supplementary file 4 [file 41467_2026_77552_MOESM7_ESM.pdf]

nature portfolio

Peer Review File

IAP antagonist tolinapant (ASTX660) in combination with pembrolizumab in advanced solid tumors: a phase I trial

Corresponding Author: Professor Pascal Meier

This file contains all reviewer reports in order by version, followed by all author rebuttals in order by version.

Parts of this Peer Review File have been redacted as indicated to maintain the confidentiality of unpublished data.

Version 0:

Reviewer comments:

Reviewer #1

(Remarks to the Author)
This ASTEROID Phase I Trial Part A had enrolled 23 patients (with 18 response-evaluable patients) as a dose-escalation, proof-of-concept study of ASTX600. It is based on a flexible 2-stage time to event Continual Reassessment Method (TITE-CRM) design. This manuscript reported the results from the Part A od the trial. The expansion study (Part B) started 2024 Feb, and likely is still on going.

The study had been successfully conducted followed the protocol and the statistical analysis and results were clearly presented according to the SAP, mainly descriptively, in a proper order.

The conclusions of the MTD and RP2D are reasonable based on the data.

A minor deviation of the statistical report, in SAP 9.2.1, it is stated that "the MTD, which is the dose level with estimated DLT rate closest to the target toxicity level, will be reported with its associated DLT rate and 90% credible interval, using all DLT evaluable patients in Phase I." However, the 90% CI was not found in the report.

Reviewer #2

(Remarks to the Author)
This is an excellent and detailed summary of clinical outcomes and correlatives of an investigator initiated combination trial evaluating ASTX660 + pembrolizumab. The results are promising, and the biomarker analysis robust. The authors provide plausible interpretation of the data and do not make claims that are overstated, although I have concerns about the response designation for some of the responding patients, which can be clarified. Please address the below comments/critiques which I hope will refine and improve the manuscript, and make it easier for the reader to correctly understand the clinical responses.

1) 77 Do all effective cancer therapies require mobilization and activation of the immune system?
2) 153 Could you provide rationale for lead-in of ATX preceding pembro?
3) 158- was 90% CI pre-specified for reporting in this trial? If not then convention is 95%. Also later you use IQR for median age. Would favor more consistent data reporting.
4) 165 text implies that 21 patients were assessed at the MTD. Consider editing to say "21 were evaluable for determining the MTD" or something like this.
5) 166: patients with rapid progression prior to 6 weeks would not be evaluable for response. Was this explicitly called out in the protocol? Generally, it is preferred to report response by IIT so as not to inflate the degree of clinical benefit observed by removing rapid progressing patients from the denominator.
6) 209 if available it would be good to know the specifics of steroid pulse +/- taper for the rash
7) 270 again it seems a bit biased to exclude rapid progressors in an overall response rate, maybe provide ORR for ITT and for protocol-specified evaluable population, if they differ.
8) 270 imRECIST is cited here but in methods line 851 the iRECIST criteria are cited. iRECIST requires confirmation of response and uses specific nomenclature for unconfirmed responses, imRECIST may also require this. Please clarify the protocol-designed response criteria and incorporate proper nomenclature when describing the case reports of response

9) 297- this patient 101-004 unclear if they meet criteria for response or even stable disease if they have symptomatic progression at week 11. Later in the discussion line 483 it is mentioned the possibility that they came off due to symptomatic pseudoprogression. Please double check that this patient meets protocol-specified response criteria for response.
0) 277- if only one ER+ breast cancer responder, you can argue that gene changes are associated with response, but would not be sufficient to argue that this gene signature would be conserved across other patients.
1) 288 this is a remarkable response and it would be important to know if there were other clinical biomarkers suggesting this was an immune-sensitive ER+ breast cancer, i.e. was ER low, was PR 0 (suggesting more basal phenotype), H&E TILs, MSI, TMB. Pembro monotherapy responses are not unheard of for ER+ disease especially ER-low.
2) 297 interesting why this patient did not receive conventional anti-endocrine + CDK4/6i combination, could you clarify this? What was ER expression? Was BRCA or TMB tested? Also would you say she had clinical benefit if she had progression at the week 12 restaging?
3) 412 would be interesting to see if the PBMC increased clonal diversity associates with extent of prior cytotoxic therapy. In breast cancer at least we see a dropoff of ICI benefit after sequential chemotherapy.
4) 494 even though the tumor was ER+ it had features not typical for the luminal type ER+ breast cancer, including high PD-L1 and TILs. Could you comment on the possibility that the tumor responded to pembro rather than the ASTX660 or the combo. In the vignette 288, it is mentioned there was marked inflammation and necosis following ASTX alone. Do you have photographs to support these findings?
5) 852 tumor burden by protocol is to be assessed every 3 cycles (9 weeks), so would presume imaging available at weeks 9 and 18. Please clarify why scans were obtained at week 11 for patient 101-004, and week 14 for patient 101-021. Could you clarify.
6) Table 2: Also would be convenient to see in this table which were the patients with clinical benefit. In column 4, would specify whether BRCA was wild type versus not tested. Would add the ER percentage if available, as this informs our understanding of biology in a heterogeneous disease type. Also why not provide the PAM50 imputations on this table?
7) Figure 2i could it be informative to see pre/post changes in RIPK, etc. also in a bar/line plot similar to 2e/g?
8) The association of pneumonitis and response is interesting and does not statistically seem likely to be due to chance. Could you further explore with your biomarkers what the potential mechanistic link could be, between the response and the pneumonitis? Or provide some plausible explanations
9) Supp Table 2: why do we not have PAM50 for the 010 responding patient

Reviewer #3

(Remarks to the Author)
The manuscript described the results from the Phase I ASTEROID trial on the combination of the inhibitor of apoptosis tolinapant with pembrolizumab for patients with advanced pretreated solid tumors. The study is presented in three parts, safety, activity and a descriptive/exploratory correlative analysis by integrating transcriptomic data, plasma proteomics, cellular infiltrates, and genomic features. The following review is from a clinical/translational point of view.

1. The study used a more modern TITE-CRM design. Its potential weaknesses should be discussed in more detail, especially the assumption of constant hazard of toxicity over time which may not hold in immunotherapy.
2. What happened with the five non-response evaluable patients? The clinical trajectory should be presented in the supplement. Non-completion of two cycles might be due to a number of reasons including rapid PD, which skews the overall results
3. PAM50 subtyping for the breast tumors was performed according to the cohort-dependent Parker method. Why was this preferred for this very small cohort over a more robust single-sample assignment such as AIMS?
4. Corticosteroid use during plasma and tissue sampling should be documented and discussed as a potential confounder
5. Adverse event profile seems to overlap to that due to ICI. A comparative discussion with tolinapant monotherapy studies is warranted, especially if AE such as asymptomatic lipase increase (which is not clinically relevant for ICI and should not be treated or even followed according to ESMO guidelines) may have clinical implications, including treatment with corticosteroids which may impair ICI efficacy (PMID: 39110922).
6. Line 277: very strong wording considering the depth of available data (or lack thereof)
7. TNF signaling has been previously recognized as a potential predictor of response to ICI (for example, PMID 40098565), while a T-cell IFN gene expression signature was predictive for pembrolizumab monotherapy in KN-119 (ESMO Breast 2023). The results here are largely consistent with previous findings, which leads to the question why these markers are thought to be predictive for IAP inhibition specifically and not pembrolizumab. Same applies for TCR repertoire expansion and TAM/Treg infiltration.
8. The wording when attempting to present mechanistic explanations based on a very small sample size without external validation is very strong and should be considerably toned down. For example, the entire TME remodeling argument of the authors is based largely on patient 101-010. In addition, these results should be presented in the context of existing multi-omics studies during ICI.
9. While I agree that the paragraph starting at line 488 describes a very interesting finding, this is really based on a single patient. In general, a paragraph listing study limitations, its descriptive/exploratory nature and lack of validation of correlative analyses is missing.
0. Why were E1L3N clone and H-score chosen instead of 22C3 and CPS which is well validated for pembrolizumab in several indications?

Version 1:

Reviewer comments:

Reviewer #2

(Remarks to the Author)
Thank you for the hard work in addressing reviewer feedback, the manuscript is excellent and all concerns have been adequately addressed from my perspective.

Reviewer #3

(Remarks to the Author)
The authors have adequately responded to all my comments

Reviewer #4

(Remarks to the Author)
This review is to comment on the authors' responses to reviewer 1's comments to the original submission of this manuscript.

1. The statistical reviewer's main comment was that the MTD and RP2D conclusions looked reasonable, and that the trial appeared to follow the protocol and SAP. The only specific issue was that the SAP called for reporting the estimated DLT rate at the MTD together with a 90% credible interval, but that interval was missing in the original manuscript. The authors addressed this by adding the 90% credible interval to the Results section and updating Supplementary Figure 1. (Supplementary Figure 1a is the plot with CIs of Prob(DLT) added and Figure 1b shows a table with CIs.)

2. Another important point was about the response denominator. Since patients with rapid progression before 6 weeks were excluded from the response-evaluable population, the reviewer asked whether this could inflate the ORR and suggested reporting both the protocol-specified evaluable analysis and an ITT analysis. The authors clarified that the response-evaluable definition was pre-specified and required eligibility, baseline tumor assessment, and at least two cycles of treatment. They also added a sensitivity analysis counting the three rapid progressors as progressive disease, giving an ORR of 6/21, or 28.6%, in the ITT population, compared with 6/18, or 33.3%, in the response-evaluable population. (This can be seen in lines 681-683 in the revised manuscript).

We thus believe that the authors satisfactorily addressed the comments from reviewer #1. We have no further comments / questions.

Reviewer #5

(Remarks to the Author)
I co-reviewed this manuscript with one of the reviewers who provided the listed reports. This is part of the Nature Communications initiative to facilitate training in peer review and to provide appropriate recognition for Early Career Researchers who co-review manuscripts.

Open Access This Peer Review File is licensed under a Creative Commons Attribution 4.0 International License, which permits use, sharing, adaptation, distribution and reproduction in any medium or format, as long as you give appropriate credit to the original author(s) and the source, provide a link to the Creative Commons license, and indicate if changes were made.
In cases where reviewers are anonymous, credit should be given to 'Anonymous Referee' and the source.
The images or other third party material in this Peer Review File are included in the article’s Creative Commons license, unless indicated otherwise in a credit line to the material. If material is not included in the article’s Creative Commons license and your intended use is not permitted by statutory regulation or exceeds the permitted use, you will need to obtain permission directly from the copyright holder.
To view a copy of this license, visit https://creativecommons.org/licenses/by/4.0/

NCOMMS-26-018776-T

We would like to thank you and the referees for the constructive and helpful comments on our manuscript. We were happy to see that the reviewers thought that our study was well conducted and reports an excellent and detailed summary of clinical outcomes.

We have followed the reviewers' suggestions and have expanded our manuscript to address their comments. This has further improved our ms.

Specifically, we have:

- **Clarified the statistical reporting**, including addition of the 90% credible interval for the MTD/DLT estimate and improved consistency in reporting clinical variables.
- **Clarified response-evaluable and ITT populations**, including a sensitivity analysis in which rapid progressors were conservatively counted as progressive disease, showing an ORR of 6/21 (28.6%) in the ITT population versus 6/18 (33.3%) in the protocol-defined evaluable population.
- **Revised the response assessment terminology and figure legends**, including clarification that imRECIST was used and that Figures 1b, 3a and 3b now distinguish protocol-specified evaluable patients from the broader ITT population.
- **Expanded and refined the patient vignettes**, particularly for patients 101-004, 101-010 and 101-021, adding clinical context, biomarker information, scan timing, treatment interruptions and interpretation of response.
- **Added further clinical and molecular annotation to Table 2**, including best response, BRCA status, TMB where available, PAM50 classification, and additional ER/PR information in Supplementary Table 2.
- **Added new supplementary analyses and figures**, including pre-/post-treatment bar/line plots for RIPK pathway components and related markers to complement the heatmap in Figure 2i.
- **Documented corticosteroid exposure more explicitly**, including a new Supplementary Table 3 detailing corticosteroid use for treatment-related adverse events and discussing corticosteroids as a potential confounder for biomarker interpretation.
- **Expanded the safety discussion**, including comparison with tolinapant monotherapy studies and a more nuanced discussion of lipase elevation, rash, pneumonitis and immune-related adverse events.
- **Clarified the interpretation of pneumonitis**, presenting its association with response as hypothesis-generating and discussing plausible biological explanations without overstatement.
- **Adjusted mechanistic claims**, especially where observations were driven by small numbers or individual patients, and reframed the correlative analyses as descriptive and hypothesis-generating.
- **Expanded the Discussion to place our findings in the context of prior multi-omics ICI studies**, while clarifying that the early immune changes observed after ASTX660 lead-in suggest immune priming before pembrolizumab exposure rather than biomarkers uniquely attributable to IAP inhibition alone.
- **Added a dedicated limitations discussion**, emphasising the small and heterogeneous Phase I all-comers cohort, the exploratory nature of the correlative analyses, and the need for validation in larger, prospectively designed studies.

Please find below a point-by-point response to the reviewers' comments, with the reviewers' comments in blue boxes and our response in 'plain text'.

REVIEWERS' COMMENTS:

Reviewer #1 (Remarks to the Author)

This ASTEROID Phase I Trial Part A had enrolled 23 patients (with 18 response-evaluable patients) as a dose-escalation, proof-of-concept study of ASTX600. It is based on a flexible 2-stage time to event Continual Reassessment Method (TITE-CRM) design. This manuscript reported the results from the Part A od the trial. The expansion study (Part B) started 2024 Feb, and likely is still on going.

The study had been successfully conducted followed the protocol and the statistical analysis and results were clearly presented according to the SAP, mainly descriptively, in a proper order.

The conclusions of the MTD and RP2D are reasonable based on the data.

A minor deviation of the statistical report, in SAP 9.2.1, it is stated that “the MTD, which is the dose level with estimated DLT rate closest to the target toxicity level, will be reported with its associated DLT rate and 90% credible interval, using all DLT evaluable patients in Phase I.” However, the 90% CI was not found in the report.

We thank the reviewer for spotting this oversight. We have now included 90 % Credible Interval (CrI) in the Results section and updated Supplementary Figure 1.

Reviewer #2 (Remarks to the Author):

This is an excellent and detailed summary of clinical outcomes and correlatives of an investigator initiated combination trial evaluating ASTX660 + pembrolizumab. The results are promising, and the biomarker analysis robust. The authors provide plausible interpretation of the data and do not make claims that are overstated, although I have concerns about the response designation for some of the responding patients, which can be clarified. Please address the below comments/critiques which I hope will refine and improve the manuscript, and make it easier for the reader to correctly understand the clinical responses.

1) 77 Do all effective cancer therapies require mobilization and activation of the immune system?

We thank the reviewer for highlighting that our original wording was too broad. We agree that effective cancer therapies can act through multiple mechanisms and do not universally depend on mobilisation or activation of the immune system. We have therefore revised this statement to better reflect the fact that immune engagement is not an obligatory feature of all successful cancer treatments, but is particularly important for therapies designed to promote durable, systemic anti-tumour responses.

2) 153 Could you provide rationale for lead-in of ATX preceding pembro?

We thank the reviewer for this question. The rationale for the 7-day lead-in with ASTX660 prior to pembrolizumab was to allow ASTX660 to first condition the tumour and immune microenvironment. Through antagonism of cIAP1/2 and XIAP, ASTX660 was expected to promote immunogenic tumour cell death, enhance antigen release, and induce inflammatory signalling, including NF-κB-dependent pathways, that support dendritic-cell activation and T-cell cross-priming. We therefore hypothesised that ASTX660 lead-in could establish a more immunogenic tumour state, which would then be more amenable to augmentation by PD-1 blockade.

A second key reason for this design was mechanistic. The lead-in window enabled collection of paired blood and tumour samples before and after ASTX660 monotherapy, allowing us to define the pharmacodynamic and biological effects of IAP inhibition alone, before pembrolizumab was introduced. This provided an opportunity to assess target modulation, cell-death pathway engagement, and changes in tumour immunogenicity in a way that would not have been possible if both agents had been initiated simultaneously.

3) 158- was 90% CI pre-specified for reporting in this trial? If not then convention is 95%. Also later you use IQR for median age. Would favor more consistent data reporting.

Thank you for your response. 90% confidence interval was pre-specified for the reporting of overall survival (OS), median OS, and OS at 12 and 24 months. We have now included median follow-up with 95% confidence interval (95% CI: [6.0, NE] months) for completeness.

We have also corrected the reporting of age and number of previous systemic anti-cancer treatment to range (Table 1), as specified in the statistical analysis plan.

4) 165 text implies that 21 patients were assessed at the MTD. Consider editing to say "21 were evaluable for determining the MTD" or something like this.

We thank the reviewer for this helpful suggestion. We agree that the original wording could have implied that all 21 patients were assessed at the MTD. We have therefore amended the text to clarify that **21 patients were evaluable for determination of the MTD**.

5) 166: patients with rapid progression prior to 6 weeks would not be evaluable for response. Was this explicitly called out in the protocol? Generally, it is preferred to report response by IIT so as not to inflate the degree of clinical benefit observed by removing rapid progressing patients from the denominator.

Per the **pre-specified protocol**, patients are considered **evaluable for response by imRECIST only** if they (i) meet eligibility criteria, (ii) have a baseline tumour assessment, and (iii) receive **at least two cycles (≥6 weeks)** of combination therapy. This definition is commonly used in early-phase studies to ensure **adequate drug exposure** for a meaningful radiographic response assessment and to avoid classifying patients who discontinue very early (often due to rapid clinical decline) as non-responders when 'on-treatment assessment' is not feasible.

This conservative definition is particularly appropriate in a heterogeneous study population, where patients may be at different stages and trajectories of disease. In contrast, later-phase trials, which typically enrol more homogeneous populations (e.g., with respect to disease characteristics, stage, and prior lines of therapy), more often rely on intention-to-treat analyses to minimize potential bias.

To address the reviewer's concern regarding potential inflation of the degree of clinical benefit, we have performed a sensitivity analysis in which the three patients in question were conservatively counted as having **progressive disease**. Under this assumption, the objective response rate (ORR) in the intention-to-treat (ITT) population is **6/21 (28.6%),** compared with **6/18 (33.3%)** in the protocol-specified evaluable population. This represents only a modest difference and does not meaningfully affect the overall interpretation of the study.

→ We have amended the legends for Figures 1b (consort diagram), 3a and 3b (waterfall and swimmer plots) to make these distinctions explicit.

6) 209 if available it would be good to know the specifics of steroid pulse +/- taper for the rash

We have added a **new Supplementary Table 3** that lists the specifics of corticosteroid use for all TRAEs and referenced this in the Results section.

7) 270 again it seems a bit biased to exclude rapid progressors in an overall response rate, maybe provide ORR for ITT and for protocol-specified evaluable population, if they differ.

Please see response to point (5)

8) 270 imRECIST is cited here but in methods line 851 the iRECIST criteria are cited. iRECIST requires confirmation of response and uses specific nomenclature for unconfirmed responses, imRECIST may also require this. Please clarify the protocol-designed response criteria and incorporate proper nomenclature when describing the case reports of response

We thank the reviewer for spotting the inconsistency in the Methods citation. We confirm that the protocol-designed criteria was **imRECIST**. The correct citation has been updated (PMID: 29341833).

9) 297- this patient 101-004 unclear if they meet criteria for response or even stable disease if they have symptomatic progression at week 11. Later in the discussion line 483 it is mentioned the possibility that they came off due to symptomatic pseudoprogression. Please double check that this patient meets protocol-specified response criteria for response.

Following two cycles (5 weeks), scans demonstrated a partial response (PR) of -63%. Treatment was interrupted at Cycle 3 day 8 due to asymptomatic G3 lipase elevation, which improved without requiring steroids. During this period of interruption, the patient developed back pain. As a result, staging scans were brought forward (week 11) to investigate the cause of pain and assess overall disease status prior to recommencing Cycle 4. Although imaging continued to show target lesions meeting imRECIST criteria for PR, non-measurable epidural disease was identified at the site corresponding to the patient's pain. At that time, it was unclear whether this represented true disease progression, tumour flare or pseudoprogression. The Safety Review Committee (SRC) determined that the patient met protocol-specified response criteria with respect to improvement in target lesions. However, considering the clinical scenario, including the need for radiotherapy to the symptomatic site and the prolonged treatment interruption, the decision was made to discontinue the patient from the trial.

We have amended the patient vignette to include these details.

10) 277- if only one ER+ breast cancer responder, you can argue that gene changes are associated with response, but would not be sufficient to argue that this gene signature would be conserved across other patients.

We acknowledge that the gene changes were observed in only one patient, and is insufficient to generalise across other ER+ breast cancer patients. We have thus toned down the wording accordingly:

We have also added a paragraph to the 'Discussion' section acknowledging the limitations of single patient observations.

11) 288 this is a remarkable response and it would be important to know if there were other clinical biomarkers suggesting this was an immune-sensitive ER+ breast cancer, i.e. was ER low, was PR 0 (suggesting more basal phenotype), H&E TILs, MSI, TMB. Pembro monotherapy responses are not unheard of for ER+ disease especially ER-low.

This patient's historical records indicate that her tumour was ER-positive at the time of metastatic disease diagnosis; however, ER/PR expression scores were not reported. Trial biopsies contained insufficient tumour content to repeat hormone receptor IHC staining or perform sequencing analyses, and therefore MSI, TMB, and PAM50 status could not be assessed to evaluate for an immune-sensitive or more basal-like phenotype. MMR proficiency was confirmed by IHC. Nevertheless, we identified biomarkers suggestive of increased sensitivity to pembrolizumab monotherapy, including high tumour-infiltrating lymphocyte (TIL) content and PD-L1 positivity.

→ We have added the MMR, TILs and PD-L1 information to Table 2 (patient disease characteristics) and the patient's case vignette and included in the discussion their potential sensitivity to pembrolizumab.

12) 297 interesting why this patient did not receive conventional anti-endocrine + CDK4/6i combination, could you clarify this? What was ER expression? Was BRCA or TMB tested? Also would you say she had clinical benefit if she had progression at the week 12 restaging?

The patient (101-004) had high ER expression (7/8) and was BRCA wild type. TMB was unknown at the time of treatment, however since then we have tested their tumour, which show high TMB (24 mut/mB). We have added this information to Table 2 and patient's case vignette.

It is not entirely clear from the available records why treatment with endocrine therapy plus a CDK4/6 inhibitor was not selected. However, the patient's clinical history indicates poor tolerance of prior endocrine therapies, including letrozole and fulvestrant, which likely contributed to this decision. Furthermore, CDK4/6 inhibitors were not approved for single-agent use in the UK, thereby restricting their clinical applicability in this setting.

On the question regarding clinical benefit, please see our response to point 9 above for further details.

13) 412 would be interesting to see if the PBMC increased clonal diversity associates with extent of prior cytotoxic therapy. In breast cancer at least we see a dropoff of ICI benefit after sequential chemotherapy.

In the plots below, there appears to be a trend towards reduced TCR clonal diversity in PBMCs with increasing numbers of prior lines of chemotherapy. This observation is consistent with the notion that sequential chemotherapy may negatively impact immune repertoire diversity, and thereby limit responsiveness to ICI. However, without baseline pre-chemotherapy measurements, it remains unclear whether the reduced diversity is caused by prior cytotoxic exposure or reflects underlying inter-patient variability. Prospective studies incorporating longitudinal sampling from the pre-treatment setting would be needed to better define this relationship. Due to the lack of pre-chemotherapy data, we decided to provide this information only in our 'response letter'.

Unpublished Data Figure Removed

14) 494 even though the tumor was ER+ it had features not typical for the luminal type ER+ breast cancer, including high PD-L1 and TILs. Could you comment on the possibility that the tumor responded to pembro rather than the ASTX660 or the combo. In the vignette 288, it is mentioned there was marked inflammation and necosis following ASTX alone. Do you have photographs to support these findings?

We thank the reviewer for this thoughtful comment. We agree that, despite the tumour being ER-positive, its high PD-L1 expression and prominent TIL infiltrate are not typical of a conventional luminal ER+ breast cancer and raise the possibility that pembrolizumab contributed to the observed response. Owing to the single-arm design of the study, we cannot formally distinguish whether the clinical benefit in this patient was driven by ASTX660, pembrolizumab, or their combination, and we have therefore been careful not to overstate causality.

Nevertheless, several observations support the view that ASTX660 made an important biological contribution and may have enhanced responsiveness to PD-1 blockade. Most notably, during the 7-day ASTX660 lead-in period, before pembrolizumab was introduced, there was clear clinical evidence of tumour inflammation and necrosis. In parallel, paired tumour analyses after ASTX660 monotherapy showed remodelling of the tumour microenvironment towards a more inflamed, immune-active state (Figure 3c), consistent with the hypothesis that ASTX660 increased tumour immunogenicity and thereby primed the lesion for subsequent checkpoint inhibition. At the same time, the baseline presence of high TILs and PD-L1 expression suggests that this tumour may already have possessed features associated with sensitivity to pembrolizumab. Taken together, we believe the most appropriate interpretation is that the observed response likely reflects a favourable immune contexture that was further augmented by ASTX660, rather than being attributable with confidence to either agent alone. We have incorporated these points into the Discussion.

Regarding the reviewer's request for supporting documentation, representative clinical photographs demonstrating inflammation and necrosis in patient 101-010 following ASTX660 lead-in are provided in Figure 3d, panel i.

15) 852 tumor burden by protocol is to be assessed every 3 cycles (9 weeks), so would presume imaging available at weeks 9 and 18. Please clarify why scans were obtained at week 11 for patient 101-004, and week 14 for patient 101-021. Could you clarify.

We thank the reviewer for highlighting this point and apologise for the lack of clarity. The statement in the Methods section that tumour assessments were performed every 3 cycles was an oversimplified summary of the protocol. To clarify, according to Section 7.6 of the protocol, the first radiological tumour assessment was scheduled **after Cycle 2** (approximately week 6), with subsequent assessments then performed **every 3 cycles / 9 weeks** thereafter (for example around weeks 15, 21, and 27, etc). In addition, the protocol allowed scan timing to be modified (Section 7.2 and 10.2.3) at investigator discretion where clinically indicated.

For **patient 101-004**, the first assessment was performed after Cycle 2 (week 5), and the next protocol-planned scan would have been after Cycle 5 (approximately week 14–15). However, Cycle 4 was delayed because of lipase elevation. During this treatment interruption, the patient developed back pain, and imaging was therefore **brought forward to week 11** to investigate the cause of symptoms and reassess disease status before deciding whether treatment could safely resume.

Patient 101-021 had scheduled scan at **week 14 prior to Cycle 5, as per protocol**.

We have revised the Methods section to clarify the tumour assessment schedule and have also amended the relevant patient narrative to make the sequence of events clearer.

16) Table 2: Also would be convenient to see in this table which were the patients with clinical benefit. In column 4, would specify whether BRCA was wild type versus not tested. Would add the ER percentage if available, as this informs our understanding of biology in a heterogeneous disease type. Also why not provide the PAM50 imputations on this table?

We thank the reviewer for these helpful suggestions. We have revised **Table 2** to make the clinical and molecular annotation more informative. Specifically, the table now includes 'best response', allowing patients with clinical benefit to be identified directly, as well as '**PAM50 classification', 'BRCA status'** (distinguishing wild type from not tested), and '**TMB'** where available. In addition, '**ER and PR scores'** have been added to **Supplementary Table 2** alongside the PAM50 data, to provide a more complete view of tumour biology in this heterogeneous cohort. We believe these additions substantially improve the interpretability of the table.

17) Figure 2i could it be informative to see pre/post changes in RIPK, etc. also in a bar/line plot similar to 2e/g?

We thank the reviewer for this helpful suggestion. To facilitate interpretation of the changes shown in the heatmap in Figure 2i, we have now added corresponding pre-/post-treatment bar/line plots to the **Supplementary Figures**, in a format analogous to Figure 2e,g. These plots provide a clearer visualisation of the direction and magnitude of change for RIPK pathway components and related markers across samples.

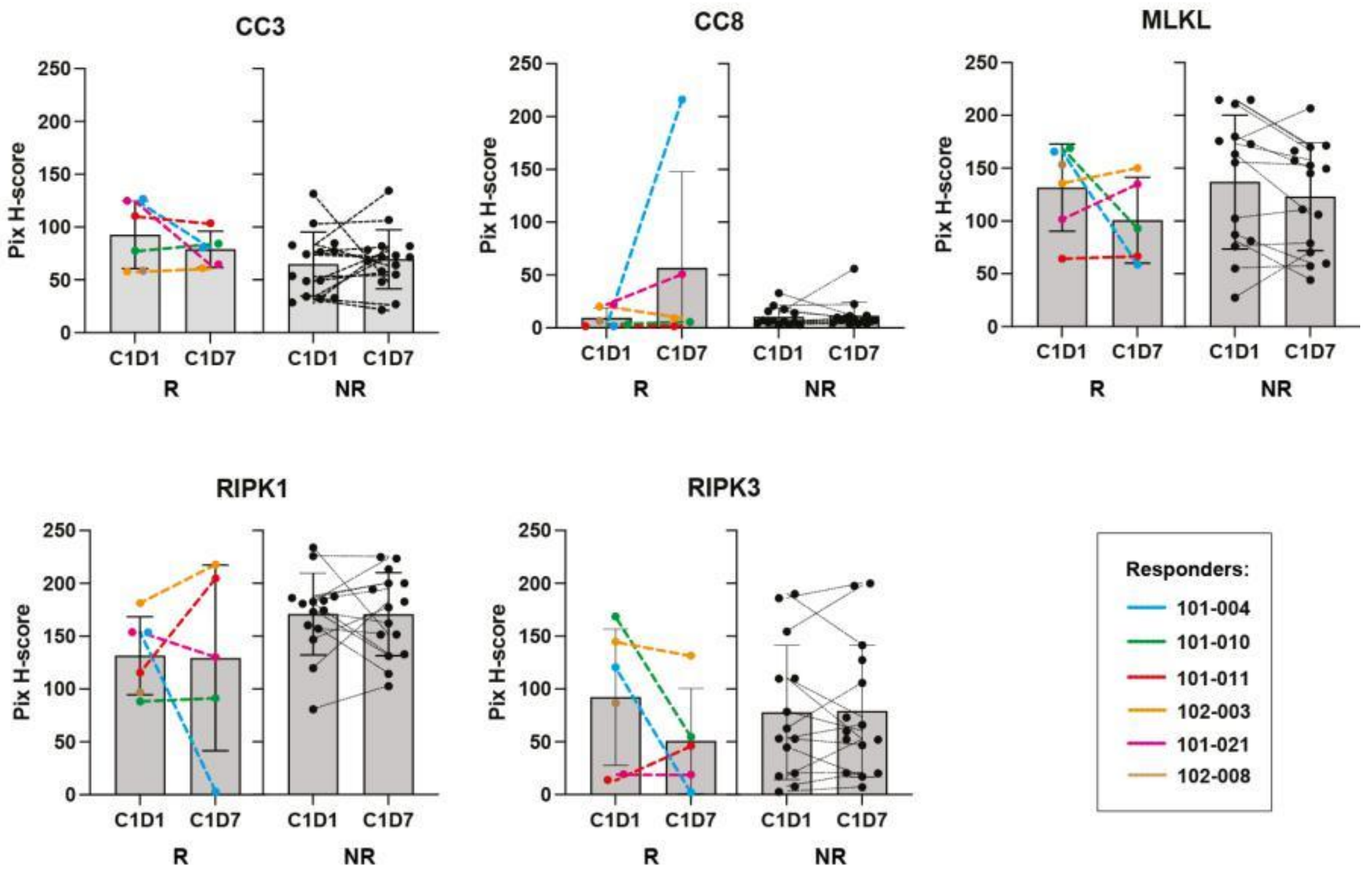

18) The association of pneumonitis and response is interesting and does not statistically seem likely to be due to chance. Could you further explore with your biomarkers what the potential mechanistic link could be, between the response and the pneumonitis? Or provide some plausible explanations

We thank the reviewer for this comment. We agree that the association between pneumonitis and clinical response is intriguing and may not be entirely attributable to chance, although the cohort size is too small to draw firm conclusions.

While our study was not powered to mechanistically dissect this relationship, several plausible explanations can be considered:

First, pneumonitis may represent an on-target manifestation of heightened systemic immune activation in patients mounting the most robust anti-tumour immune responses. In this context,

ASTX660 may enhance tumour immunogenicity and inflammatory signalling, while pembrolizumab may further amplify activated T-cell responses, together increasing both anti-tumour activity and the risk of immune-mediated tissue inflammation.

Second, although patients were screened and monitored for concomitant inflammatory and infectious conditions, it is still possible that patients who developed pneumonitis had an underlying pre-existing inflammatory condition, predisposing both to response and to immune-related toxicity. This would be consistent with the broader concept that efficacy and immune-related adverse events can, in some cases, arise from overlapping biology. Notably, three of these cases occurred in patients with predisposing risk factors, such as extensive lung metastases (patient 101-011), prior radiotherapy to the chest (patient 102-003), and radiotherapy to T12 metastasis (patient 101-021). However, areas of pneumonitis do not radiologically overlap with sites of metastases or prior treatment fields.

Third, given the mechanism of IAP antagonism, a contributory role for enhanced non-canonical NF-κB-driven inflammatory signalling may also be plausible. Such effects could lower the threshold for inflammatory damage in normal tissues such as lung.

Although our biomarker analyses support treatment-associated immune activation, they do not allow us to define a specific mechanistic link between pneumonitis and response in this study. We have therefore tempered our interpretation in the manuscript and state that this observation should be regarded as hypothesis-generating. Larger studies with longitudinal immune profiling, including analyses of circulating cytokines, T-cell activation states, and tissue-specific immune signatures, will be needed to clarify whether pneumonitis and response are biologically linked in this setting.

We have added these points to our Discussion.

19) Supp Table 2: why do we not have PAM50 for the 010 responding patient

Unfortunately, the biopsy material from patient 101-010 contained insufficient tumour content to support RNA-seq analysis and therefore PAM50 classification could not be performed. We have now made this limitation explicit in the Results section.

Reviewer #3 (Remarks to the Author)

The manuscript described the results from the Phase I ASTEROID trial on the combination of the inhibitor of apoptosis tolinapant with pembrolizumab for patients with advanced pretreated solid tumors. The study is presented in three parts, safety, activity and a descriptive/exploratory correlative analysis by integrating transcriptomic data, plasma proteomics, cellular infiltrates, and genomic features. The following review is from a clinical/translational point of view.

1. The study used a more modern TITE-CRM design. Its potential weaknesses should be discussed in more detail, especially the assumption of constant hazard of toxicity over time which may not hold in immunotherapy.

We thank the reviewer for raising this important point.

First, the linear weight is the default and most widely adopted choice in practice. In a recent methodological review of 61 published TITE-CRM trials, Yin et al. (2024) reported that 54.1% used the uniform weight function, while only 14.8% used alternative weights; the remainder did not report the weight function at all. The uniform weight is the prevailing convention when the time-to-toxicity profile of the investigational agent is not known a priori, which is our situation. Yin et al. further note that, owing to the typical sparsity of DLTs in early-phase trials, it is generally not feasible to empirically verify the weight assumption even retrospectively.

Second, the choice of weight function has little influence on the final MTD declaration. As Cheung and Chappell (2000) explicitly demonstrate, the final estimation of the dose-toxicity parameter is based on the marginal (unweighted) model using completed binary DLT outcomes, not on the weighted model; the failure-time distribution therefore affects the final recommendation only indirectly, through its effect on in-trial allocation. Their simulation study confirms this empirically: the proportion of correct MTD recommendations is essentially identical across uniform, log-logistic, and Weibull failure-time distributions within each toxicity scenario (Cheung and Chappell, 2000, Table 2).

Third, our design further mitigates any other concern about weight misspecification through its two-stage structure. Stage 1 is a rule-based run-in: cohorts of two patients escalate from the lowest dose level only when no DLT is observed, and the TITE-CRM model is triggered only once the first DLT occurs. This follows the recommendation by Cheung and Chappell (2000) to guard against aggressive escalation before toxicities have been observed.

2. What happened with the five non-response evaluable patients? The clinical trajectory should be presented in the supplement. Non-completion of two cycles might be due to a number of reasons including rapid PD, which skews the overall results.

We thank the reviewer for raising this point, which was also raised by Reviewer 2. The five non-response-evaluable patients are shown in the CONSORT diagram in Figure 1b. The reasons for non-evaluability were: incorrect dosing (n=1), withdrawal of consent (n=1), and rapid progressive disease before six weeks of treatment (n=3).

Per the **pre-specified protocol**, patients are considered **evaluable for response by imRECIST** only if they (i) meet eligibility criteria, (ii) have a baseline tumour assessment, and (iii) receive **at least two cycles (≥6 weeks)** of combination therapy. This definition is commonly used in early-phase studies to ensure **adequate drug exposure** for a meaningful radiographic response assessment and to avoid classifying patients who discontinue very early (often due to rapid clinical decline) as non-responders when on-treatment assessment is not feasible.

This conservative definition is particularly appropriate in a heterogeneous study population, where patients may be at different stages and trajectories of disease. In contrast, later-phase trials, which typically enrol more homogeneous populations (e.g., with respect to disease characteristics, stage, and prior lines of therapy), more often rely on intention-to-treat analyses to minimize potential bias.

To address the reviewer's concern regarding potential inflation of the degree of clinical benefit, we have performed a sensitivity analysis in which the three patients in question were conservatively counted as having **progressive disease**. Under this assumption, the objective response rate (ORR) in the intention-to-treat (ITT) population is **6/21 (28.6%),** compared with **6/18 (33.3%)** in the protocol-specified evaluable population. This represents only a modest difference and does not meaningfully affect the overall interpretation of the study.

We have amended the legends for Figures 1b (consort diagram), 3a and 3b (waterfall and swimmer plots) to make these distinctions explicit.

3. PAM50 subtyping for the breast tumors was performed according to the cohort-dependent Parker method. Why was this preferred for this very small cohort over a more robust single-sample assignment such as AIMS?

Both AIMS and PAM50 were performed for our sample cohort. While we agree with the reviewer that AIMS, in principle, is more appropriate for single sample calling, AIMS calls for this cohort were not concordant with the gold standard histology calls (see table below). For example, 5 samples that were negative for HER2 on ISH were designated as *Her2* subtype according to AIMS.

We therefore relied on PAM50, which showed better overall concordance with the histological classification.

Unpublished Data Removed

4. Corticosteroid use during plasma and tissue sampling should be documented and discussed as a potential confounder.

We thank the reviewer for highlighting this important potential confounder. We have now documented corticosteroid exposure during the plasma and tissue sampling window and clarified this point in the manuscript. Specifically, no patients received concomitant corticosteroids during the key pharmacodynamic sampling period (**C1D1 to C1D7**), with the exception of one patient who was receiving long-term **physiological replacement-dose hydrocortisone** for adrenal insufficiency. We therefore believe that the pharmacodynamic analyses are unlikely to have been materially confounded by corticosteroid use during this interval.

To address this more explicitly, we have added a discussion of the potential impact of corticosteroids on biomarker interpretation and included a new **Supplementary Table 3** summarising corticosteroid use for treatment-related adverse events.

5. Adverse event profile seems to overlap to that due to ICI. A comparative discussion with tolinapant monotherapy studies is warranted, especially if AE such as asymptomatic lipase increase (which is not clinically relevant for ICI and should not be treated or even followed according to ESMO guidelines) may have clinical implications, including treatment with corticosteroids which may impair ICI efficacy (PMID: 39110922).

We thank the reviewer for this comment. We agree that the adverse-event profile observed with ASTX660 plus pembrolizumab overlaps in part with that expected for immune checkpoint inhibition alone, and that comparison with tolinapant monotherapy is therefore informative. We have now expanded the Discussion accordingly. In brief, the combination showed a broadly comparable rate of lipase elevation to that reported with tolinapant monotherapy, whereas rash and pneumonitis appeared less frequent in the monotherapy setting despite a more intensive dosing schedule. Taken together, these observations are consistent with the possibility that the addition of pembrolizumab increases the incidence of immune-mediated toxicities relative to ASTX660 alone.

We also acknowledge the reviewer's concern regarding the management of asymptomatic lipase elevation and the potential implications of corticosteroid use for ICI efficacy. In our dataset, among 10 cases of lipase increase, only one patient received corticosteroids, specifically oral prednisolone 10 mg daily for 7 days. The remaining cases resolved with dose interruption, dose adjustment, or observation alone. Given the low dose and short duration of steroid exposure in this case, we consider it unlikely that pembrolizumab efficacy was meaningfully compromised. More broadly, although asymptomatic lipase elevations may not always require active treatment, management must also take into account the need to avoid prolonged treatment interruption in patients deriving potential benefit.

Corticosteroids given for other treatment-related adverse events were also limited in most cases and generally remained below 1 mg/kg prednisone equivalent, with the exception of one patient who received high-dose methylprednisolone for pneumonitis. Notably, this patient still experienced durable clinical benefit, suggesting that clinically indicated corticosteroid use does not necessarily abrogate anti-tumour activity in this setting.

We have incorporated these points into the Discussion and added a new **Supplementary Table 3**, summarising corticosteroid use for treatment-related adverse events.

6. Line 277: very strong wording considering the depth of available data (or lack thereof)

We agree and have adjusted the original wording. We have also added a discussion on the limitations of the study to the 'Discussion' section.

7. TNF signaling has been previously recognized as a potential predictor of response to ICI (for example, PMID 40098565), while a T-cell IFN gene expression signature was predictive for pembrolizumab monotherapy in KN-119 (ESMO Breast 2023). The results here are largely consistent with previous findings, which leads to the question why these markers are thought to be predictive for IAP inhibition specifically and not pembrolizumab. Same applies for TCR repertoire expansion and TAM/Treg infiltration.

We agree with the reviewer that TNF signalling and IFN-related gene expression have previously been associated with response to immune checkpoint inhibition, and we have now clarified this point in the revised manuscript.

The key distinction in our study is the **timing of biomarker assessment**. Paired tumour and blood samples were collected **before treatment and after the 7-day ASTX660 lead-in, but before the first dose of pembrolizumab**. Thus, the early pharmacodynamic changes we report cannot be attributed to pembrolizumab exposure. Rather, they show that IAP inhibition alone is sufficient to remodel the tumour immune microenvironment, inducing a TNF- and IFN-enriched state together with evidence of enhanced T-cell activation.

We therefore do not intend to imply that TNF signalling, IFN signatures, TCR repertoire expansion, or TAM/Treg infiltration are uniquely predictive of IAP inhibition as a monotherapy. Instead, we interpret these features as **markers of an ASTX660-induced immune-primed state** that may render tumours more susceptible to subsequent PD-1 blockade. In this model, IAP inhibition acts upstream to lower the threshold for inflammatory tumour cell death and immune activation, thereby generating a tumour microenvironment in which pembrolizumab is more likely to be effective.

Accordingly, we have revised the text to avoid over-attributing these biomarkers specifically to IAP inhibition alone. We now describe them as candidate biomarkers of response to the **ASTX660-pembrolizumab combination**, reflecting treatment-induced immune priming during the ASTX660 lead-in phase and subsequent sensitivity to immune checkpoint blockade.

→ We have added this to the 'Discussion' section.

8. The wording when attempting to present mechanistic explanations based on a very small sample size without external validation is very strong and should be considerably toned down. For example, the entire TME remodeling argument of the authors is based largely on patient 101-010. In addition, these results should be presented in the context of existing multi-omics studies during ICI.

We agree with the reviewer that our sample size is limited and have toned down mechanistic interpretations accordingly. We now emphasize that observations such as TME remodelling are hypothesis-generating, particularly as they are largely driven by a single patient.

We have also revised the manuscript to place our findings in the context of prior multi-omics studies of ICI across tumour types, which have consistently linked IFN signature, TCR repertoire dynamics, tumour myeloid/lymphoid composition, and mutational burden with treatment response. In this context, our results are concordant with established immune correlates of ICI response, but extend these observations by suggesting that IAP inhibition may induce such immune features **prior** to pembrolizumab exposure.

→ We have addressed these points in the 'Discussion' section.

9. While I agree that the paragraph starting at line 488 describes a very interesting finding, this is really based on a single patient. In general, a paragraph listing study limitations, its descriptive/exploratory nature and lack of validation of correlative analyses is missing.

We thank the reviewer for this important comment and agree that the findings described are based on a limited sample size, with some observations driven by a single patient. We have revised the text to tone down the interpretation of these results and clarify their exploratory nature. This highlights the small, heterogeneous cohort typical of a phase I all-comers study, the descriptive and hypothesis-generating nature of the correlative analyses and that these findings require confirmation in larger, prospectively designed studies.

à We have addressed these limitations in the fully revised 'Discussion' section.

0. Why were E1L3N clone and H-score chosen instead of 22C3 and CPS which is well validated for pembrolizumab in several indications?

High concordance between the PD-L1 clones E1L3N and 22C3 has been demonstrated in several studies (PMID: 28278348, 39351784, 39282585, 36352254). The PD-L1 E1L3N clone was selected because it had been validated and optimised by the ICR Cancer Biomarkers Laboratory, and was readily available, cost-effective, and compatible with the staining platforms in use at the time (PMID: 30179225).

Regarding PD-L1 scoring, both TPS and CPS provide a percentage-based assessment of PD-L1-positive cells, but do not capture staining intensity or its distribution. While these scores are appropriate for clinical treatment decision-making in defined tumour types, they provide a less complete description of PD-L1 expression for exploratory biomarker analyses. We therefore used the H-score, which provides a semi-quantitative measure of tumour-cell PD-L1 expression by incorporating staining intensity. This enabled comparison across samples and was aligned with the exploratory aims of this phase I all-comers study, in which CPS is not uniformly validated or applicable across all tumour types included.

We have now cited the reference supporting E1L3N antibody validation in the Materials and Methods.

NCOMMS-26-018776-T

We would like to thank you and the referees for reviewing our revised manuscript. We are pleased to know that all reviewers are satisfied with the changes made and that all concerns have been addressed.

None of the reviewers' comments required a further response.

REVIEWERS' COMMENTS:

Reviewer #2 (Remarks to the Author):
Thank you for the hard work in addressing reviewer feedback, the manuscript is excellent and all concerns have been adequately addressed from my perspective.

Reviewer #3 (Remarks to the Author):
The authors have adequately responded to all my comments

Reviewer #4 (Remarks to the Author):
This review is to comment on the authors' responses to reviewer 1's comments to the original submission of this manuscript.

1. The statistical reviewer's main comment was that the MTD and RP2D conclusions looked reasonable, and that the trial appeared to follow the protocol and SAP. The only specific issue was that the SAP called for reporting the estimated DLT rate at the MTD together with a 90% credible interval, but that interval was missing in the original manuscript. The authors addressed this by adding the 90% credible interval to the Results section and updating Supplementary Figure 1. (Supplementary Figure 1a is the plot with CIs of Prob(DLT) added and Figure 1b shows a table with CIs.)

2. Another important point was about the response denominator. Since patients with rapid progression before 6 weeks were excluded from the response-evaluable population, the reviewer asked whether this could inflate the ORR and suggested reporting both the protocol-specified evaluable analysis and an ITT analysis. The authors clarified that the response-evaluable definition was pre-specified and required eligibility, baseline tumor assessment, and at least two cycles of treatment. They also added a sensitivity analysis counting the three rapid progressors as progressive disease, giving an ORR of 6/21, or 28.6%, in the ITT population, compared with 6/18, or 33.3%, in the response-evaluable population. (This can be seen in lines 681-683 in the revised manuscript).

We thus believe that the authors satisfactorily addressed the comments from reviewer #1. We have no further comments / questions.

Reviewer #5 (Remarks to the Author): I co-reviewed this manuscript with one of the reviewers who provided the listed reports. This is part of the Nature Communications initiative to facilitate training in peer review and to provide appropriate recognition for Early Career Researchers who co-review manuscripts.
